# Physiological and health-related effects of 5G-relevant radiofrequency fields: A systematic review of experimental studies in humans and mammals

**DOI:** 10.1371/journal.pone.0361104

**Published:** 2026-11-04

**Authors:** Lambert Bodewein, Dagmar Dechent, Tanja Emonds, David Graefrath, Anna Sophia Keller, Julia Krabbe, Lavender Morgan, Philine Johanna Scheelhaase, Gernot Schmid, Pia Schneeweiss, Sarah Driessen

**Affiliations:** 1 Research Center for Bioelectromagnetic Interaction (femu)-Institute for Occupational, Social and Environmental Medicine, Medical Faculty, RWTH Aachen University, Aachen, Germany; 2 Institute for Occupational, Social and Environmental Medicine, Medical Faculty, RWTH Aachen University, Aachen, Germany; 3 Electromagnetic Compatibility & Optics, Seibersdorf Laboratories, Seibersdorf, Austria; National Research Council: Consiglio Nazionale delle Ricerche, ITALY

## Abstract

The introduction of the latest mobile communications standard of the fifth generation (5G) is accompanied by controversial discussions about potential health risks due to radiofrequency electromagnetic field exposure. Therefore, we conducted a systematic review of experimental and epidemiological studies investigating the physiological and health-related effects of 5G-relevant radiofrequency electromagnetic fields in humans and mammals. The review protocol has been registered in PROSPERO (CRD42022374434) and the review conforms to the PRISMA guidelines. The risk of bias and the quality of included studies were assessed using the recommended approach by the Office of Health Assessment and Translation of the National Toxicology Program. A total of 100 experimental animal and seven human controlled studies were included, analyzed, and evaluated. Narrative summaries were performed for a total of 23 topics (e.g., effects on cognition, male fertility, and thermoregulation). However, investigated species and outcomes in studies were very heterogeneous. Additionally, most studies had substantial methodological weaknesses. Only 11 studies showed a low risk of bias. In summary, for most topics, the current scientific data were inadequate to draw any conclusions about the physiological and health-related effects of radiofrequency electromagnetic fields in the investigated frequency range of 3–30 GHz. However, moderate evidence below the limit values was found for cytokine secretion in mice and low evidence was found for structural changes in the hippocampus of rats above the limit values, when taking the exposure limits presently recommended by the International Commission on Non-Ionizing Radiation Protection as the basis. Therefore, we strongly recommend high-quality systematic research. Particular attention should be paid to reasonable hypotheses, experimental studies on effects on the skin, eyes, and temperature increase, exposures to different frequencies and field strengths, as well as epidemiological studies with separate consideration of 5G (if possible).

## Introduction

### Background

Exposure to radiofrequency electromagnetic fields (RF EMF) has grown steadily over the past decades [1–6]. This has led to concerns about possible adverse effects on human health.

To date, the only scientifically accepted biological effect of RF EMF at frequencies above 10 MHz is an increase in temperature caused by the absorption of electromagnetic energy by body tissues. This mechanism of action (thermal effects) is the basis for current limits for exposure to RF EMF recommended by the International Commission on Non-Ionizing Radiation Protection (ICNIRP) and Institute of Electrical and Electronics Engineers (IEEE) [7,8]. Below the limit values, health-related thermal effects are not expected. However, there is scientific and public debate whether there may also be non-thermal effects below the exposure limits, especially in the case of long-term exposures [9–16].

Recently, discussions about potential health risks below the limit values have been rekindled by the introduction of the latest mobile communications standard of the fifth generation (5G; 694 MHz–28 GHz; see Description of exposure). The dispute appears to be similar in nature and scale to the protests against mobile network expansion in the 1990s and 2000s [17,18]. Additionally, health concerns are often mixed with unsystematically compiled information about other suspected dangers from “electrosmog” up to conspiracy theories, e.g., a link between the introduction of 5G and the COVID-19 pandemic [19].

For frequencies in the range of existing mobile communications applications (< 3 GHz, like Universal Mobile Telecommunications System (UMTS) and Long Term Evolution (LTE)), many studies are available to evaluate possible health effects. For example, in the international topic-specific literature database EMF-Portal [20] there are over 1800 experimental studies listed with exposures to mobile communications [21]. Although 5G also uses frequency bands below 3 GHz, the vast majority of 5G applications and services are presently using the frequency range 3.4–3.8 GHz, particularly in urban environments. However, future 5G applications are going to use higher frequencies up to the millimeter-wave range (> 30 GHz). Mobile millimeter-wave applications in 5G public networks are presently commercialized in a few countries only (e.g., USA, Japan, Australia), mainly using the 26–28 GHz frequency range. In Europe this is still not the case and 5G data communication in the 26–28 GHz band is presently taking place on a relatively small scale for fixed wireless access (as alternative to glass-fiber internet access) and in private networks, typically set up in fully automated smart factories. For frequencies ≥ 3 GHz, there are significantly fewer studies available on physiological and health-related effects than for the mobile communications frequencies used in the past, e.g., in EMF-Portal, only about 385 experimental studies on millimeter waves (30 GHz–300 GHz) [22] can be found. In addition, the higher the frequency, the more RF EMF energy is absorbed by the body surface resulting in potentially different sites of effects [7].

### Description of outcome

Exposure to RF EMF is going to affect large parts of the population in the future due to the widespread use of mobile communications and 5G. Therefore, the earliest possible detection of any adverse effects of RF EMF below the limit values is of high interest, even without scientific acceptance of any non-thermal mechanism of action. Hence, the outcome will not be restricted in advance, and all reported effects, whether potentially adverse, beneficial (therapeutic), or of unclear health relevance, will be included in the review. We define an adverse effect as any undesired harmful effect resulting from exposure. In contrast, beneficial effects are, e.g., desirable therapeutic or positive effects that improve a person’s health following exposure to RF EMF. Additionally, there may be effects that are unclear in terms of their relevance to health, e.g., changes in brain activity that have no further health implications. We define results as “no effect” if there are no differences between the exposed and control groups. The endpoints include health-related (e.g., ocular damage, cancer), physiological (e.g., brain activity), behavioral (e.g., motor activity, anxiety), and therapeutic outcomes in humans and mammals.

### Description of exposure

5G operates at several different frequency bands. In 2019, the European Union harmonized three 5G “pioneer bands”: 694–790 MHz, 3.4–3.8 GHz (5G frequency range 1), and 24.25–27.5 GHz (5G frequency range 2). Globally, 3.3–3.8 GHz has become the most used 5G frequency band [23]. However, further frequencies in the range 3–30 GHz are also used, e.g., 4.9 GHz in China [24]. The main sources of 5G exposure are mobile phones, mobile phone base stations of 5G networks, and wearables. Additionally, some types of wireless local area network (WLAN), radar applications (e.g., military radar) [25], and some medical applications [26] use frequencies in this range. The large variety of applications will lead to manifold exposure scenarios in practice. While exposure from base stations and access points usually leads to (widely) uniform far-field exposure, close near-field exposure conditions of different body parts are to be expected when using mobile phones and wearables, depending on which devices and how they are used. Particularly in case of millimeter-wave applications, it must be noted that absorption takes place almost entirely inside the skin and that inner organs remain largely unexposed due to the decreasing penetration depth with increasing frequency.

Exposure to RF EMF induces body/tissue internal electric fields and the energy is absorbed in tissues. This can lead to an increase in tissue temperature and, depending on the level and duration of exposure, cause thermal biological effects. To protect the public from these effects, the most important parameters to limit exposure are the internal electric field strength and the rate of absorption of RF energy (specific absorption rate, SAR). The SAR is directly related to the internal electric field strength via the electric conductivity and the mass density of the tissue. In the 10 MHz–6 GHz range, SAR is the most important exposure parameter. The SAR can be determined numerically and experimentally; however, this requires sophisticated and elaborate methods.

At higher frequencies above 6 GHz, absorption is confined to the surface of the exposed tissue, and the absorbed power density (APD) can be used as an appropriate measure of exposure.

Alternatively, the more easily measurable incident electric and magnetic field strength or the incident power density (PD) at the site of the exposed body can be used as measurable exposure parameters. However, it is important to note that these body/tissue external exposure metrics can in general only be seen as rough approximations for the biologically relevant body/tissue internal exposure metrics. This is due to the coupling of the external fields into the body, which follows highly complex physical phenomena and therefore depends on several factors, e.g., frequency, size, orientation and posture of the exposed body, field polarization, and dielectric properties of body tissues. Hence, it is not possible to provide a generalized conversion table between the body/tissue external and body/tissue internal exposure metrics. The ICNIRP sets limits for both body/tissue internal and body/tissue external quantities, referring to the body/tissue external limits as “reference levels” and the body/tissue internal limits as “basic restrictions” [7].

For frequencies in the range of existing mobile communications applications (< 3 GHz), many studies and assessments by international expert groups are available to evaluate possible health effects (see Background). Therefore, this review focuses on the newly introduced frequency range 3–30 GHz.

### Previous reviews on the topic

In the past five years, several authors and organizations have conducted comprehensive reviews on the potential health risks of 5G-related exposures. Systematic searches (S1 File) using EMF-Portal [20] and PubMed [27] returned twelve reviews on the topic (S2 File). There are several contradictory findings in these previous reviews. The quality of most of them is questionable as none of these reviews were performed systematically based on international systematic review guidelines. Thus, important methodological steps were missing (S2 File, column “Limitations”), e.g., almost all of them didn’t provide search strings. In addition, the quality of the studies included in the previous reviews varied and could be one reason for the inconsistency in findings. To the best of our knowledge, a systematic search, analysis, and evaluation of the current state of scientific knowledge on the health-related effects of 5G-relevant RF EMF exposure is therefore still missing.

### Objectives

This paper describes a systematic review which provides a comprehensive analysis of the following PECOS (**P**opulation, **E**xposure, **C**omparator, **O**utcome, **S**tudy design) question [28] carried out according to the most rigorous, internationally acknowledged, and transparent methodology:

To assess the physiological, health-related, behavioral, and therapeutic effects (O) of exposure to 5G-relevant RF EMF (3–30 GHz) (E) in humans and mammals (P) compared to no or lower exposure level (C) in experimental *in vivo* and epidemiological studies (S).

The specific objectives are to (i) identify the relevant animal and human (experimental and epidemiological) *in vivo* literature, (ii) assess the risk of bias for individual studies, (iii) synthesize the evidence on the exposure-outcome relationship, and (iv) rate the confidence in the body of evidence.

## Methods

This systematic review conforms to the Preferred Reporting Items for Systematic Reviews and Meta-Analyses (PRISMA) guidelines [29]. The PRISMA checklist is provided in S1 Checklist**.** At the beginning of the project, a review protocol was created according to PRISMA-P (Preferred Reporting Items for Systematic Reviews and Meta-Analyses Protocols) guidelines [30,31] and registered in the international database for systematic reviews PROSPERO [32]. Amendments in the review compared to the protocol are documented along with the rationale in the section Amendments compared to protocol [30]. Methodological limitations in individual studies were assessed using the Office of Health Assessment and Translation (OHAT) Risk-of-Bias Rating Tool for Human and Animal Studies [28,33]. Study selection, data extraction and study appraisal were performed in duplicate, independently by two authors. The applied methods are based on our previous systematic reviews [34–38], the protocol for a systematic review by Romeo et al. [39], and the systematic review protocols commissioned by the World Health Organization (WHO) [40–43]. Records and extraction data were managed using the Covidence web application for systematic reviews [44]. Literature references were managed using Mendeley Reference Manager [45].

### Eligibility criteria

According to our PROSPERO protocol, studies were included if they complied to a set of pre-defined inclusion criteria for each element of our PECOS question (Table 1). In case a study reported both outcomes for conditions valid to our criteria and outcomes for conditions invalid to our criteria (e.g., when both frequencies in and outside the relevant spectrum were examined), the study was included in the review, but only outcomes for conditions valid to our criteria were extracted and evaluated.

**Table 1:** PECOS Statement.

PECOS	Inclusion criteria	Exclusion criteria
**Population**	• Members of the general population and occupationally active individuals, without restriction on sex, age, or other individual characteristics • Mammals without restriction on species, sex, age, or other individual characteristics	• *In vitro* studies (studies on cells, isolated organs and tissues) • *In silico* studies
**Exposure**	• 5G-relevant RF EMF (3–30 GHz) at any exposure level supported by measured or calculated metrics as specified in the PROSPERO protocol [32] • Exposure surrogates for duration of use, such as cumulative hours of use of RF emitting devices or cumulative lifetime exposure calculated for the individual based on measured RF EMF at work or at home and duration of exposure	• Static or extremely low-frequency magnetic and/or electric fields • RF EMF < 3 GHz or > 30 GHz • RF EMF with the sole intent of heating tissues significantly above physiological relevant levels (> 42 °C) and damaging or destroying tissues • Contact current • Co-exposures • Unspecified exposure metrics
**Comparator**	• Non-exposed control condition (i.e., no exposure beyond background exposure level) or an exposure condition at a lower level	• No control group or exposure condition of control group unclear
**Outcome**	• Any health-related (e.g., ocular damage, cancer), physiological (e.g., brain activity), behavioral (e.g., motor activity, anxiety), or therapeutic endpoints	• None
**Study design**	• Experimental animal studies (EA) • Human controlled trials (single-blind or double-blind) • Epidemiological cohort and case-control studies	• Reviews and meta-analyses • Statements, opinion papers, editorials • Research reports • Conference abstracts • Dosimetric studies • Case studies • Cross-sectional studies • Ecological studies • Studies on electromagnetic interference involving implants • Studies without peer review

### Information sources and search strategy

Our primary information sources were PubMed (U.S. National Library of Medicine, National Institutes of Health) [27], Web of Science (Clarivate) [46] and EMF-Portal [20], the latter being a dedicated database of the scientific literature on biological and health-related effects of exposure to electromagnetic fields. The identification of studies to be included in the EMF-Portal is based on systematic search strategies in major literature databases, including PubMed, Web of Science Core Collection and IEEE Xplore Digital Library. Scientific journals not listed in these databases as well as reference lists of review articles are additionally screened to identify further relevant publications. Thus, the EMF-Portal comprises EMF-related articles from major international literature databases and other sources.

The search strategy design was based on (i) our long-term experience with literature searches as providers of our database EMF-Portal, (ii) our previous systematic reviews [34–38] as well as (iii) the systematic review protocols commissioned by the WHO [40–43].

The complete search strings in the different electronic databases are provided in S3 File.

For the search in the EMF-Portal, the following items were selected: “Experimental studies”, “Epidemiological studies” and “Medical applications” as topics and “Radiofrequency (≥ 10 MHz)” and “mobile communications” as frequency ranges. No further search terms were utilized, i.e., we searched for all articles for which the aforementioned items were true.

The searches utilized in PubMed and Web of Science were based on a search in title and abstract using a list of search terms referring to the exposure (e.g., “millimeter wave” or “5G”) in combination with relevant species (e.g., “human” or “hamster”) while excluding articles featuring key words without relevance for our review (e.g., “nomophobia”). The terms were adjusted to achieve a balance of precision and comprehensiveness in results. We reduced the number of key words via a manual trial-and-error process by removing single keywords and checking whether the search would lead to fewer matches or not. All search terms resulting in no changes of our search were removed.

To calibrate our search strategies, we performed a bibliographic search without time restrictions. We then compared the search outputs to the content of a library of 41 “seed studies”. The seed studies were identified as relevant for our topic and extracted from the comprehensive review of Simkó et al. [47]. Our search retrieved 100% of the validation set. The search was supplemented by backward citation searches of included publications and relevant reviews. We ran our first search at the start date of the systematic review process (November 2022) and re-ran searches prior to the final analysis (August 2023).

### Study selection

All bibliographic records were imported into Covidence web application for systematic reviews and the appropriate functions were used to remove duplicates. Screening for eligibility of all potentially relevant articles was conducted in two stages. First, titles and abstracts of the identified articles were screened independently by two authors (LB, PJS, DD, SD, TE) and records that did not fulfill the inclusion criteria (see Eligibility criteria) were excluded. In the second stage, the full texts were retrieved for those publications that meet the inclusion criteria, and the articles were independently reviewed by two authors (LB, PJS, DD, SD, TE). The authors jointly made a final decision about the inclusion or exclusion of the reviewed articles and possible disagreement was resolved by discussion with a further reviewer. We documented the selection process in a study flow diagram according to the PRISMA reporting guidelines. A separate table including articles excluded at the stage of full-text screening, with the reason for exclusion for each article (in case of multiple exclusion reasons, the most distinct reason is stated) is provided in S4 File.

### Data extraction

Two authors (LB, PJS, DD, SD, TE) independently extracted details regarding the design, methods, and analysis of results of each study. Extracted data included bibliographic data, fundings, declaration of competing interest, study design, examined species and the number and size of groups, exposure parameters (e.g., frequency, field strength, exposure duration, and signal pattern), study focus (endpoints) and methods, results, and additional remarks. In addition, for epidemiological cohort, case-control, and cross-sectional studies, the study population investigated, and confounders were recorded. See S5 File for a comprehensive list of parameters that were extracted from each study. The most important study parameters of all extracted studies can be found in S1 Tables and S2 Table.

### Study appraisal

The internal validity of the included studies (i.e., the extent to which individual studies minimized biases in study design, methods, and analysis of results) and the quality of included studies was assessed using a modified version of the recommended approach by the Office of Health Assessment and Translation of the National Toxicology Program (NTP) [28,33]. The OHAT Risk-of-Bias Rating Tool for Human and Animal Studies was developed for studies with a focus on environmental health and toxicology. It consists of a set of questions and provides detailed instructions on how to evaluate the credibility of the results, that is, the risk of an over- or underestimation of the exposure effects.

For our review, a few aspects of the OHAT protocol were modified. Assessment criteria for crossover studies were included for question 1 and 2 according to Ding et al. [48] as OHAT does not specify the handling of crossover studies. Moreover, the rating for question 3 was redefined. In the original OHAT protocol, this criterion only applies to animal studies. However, in human studies with exposures to EMF, systematic differences between the experimental conditions during exposure versus sham exposure could substantially bias the outcomes as well. Therefore, this criterion is crucial for a well-controlled design in human studies. For more details on the assessed risk-of-bias domains and questions according to OHAT see S6 File. All modifications are specified in detail below each question.

The OHAT criteria were independently assessed by at least two authors (LB, PJS, DD, SD, TE) for all included studies according to the following ratings: “++” definitely low risk of bias, “+” probably low risk of bias, “-” probably high risk of bias, or “--” definitely high risk of bias. Disagreements in the assessment were discussed between the authors and resolved by consensus.

Finally, the OHAT approach was utilized to place individual studies into quality categories. This approach outlines a 3-tier system to rate study quality (1^st^ tier: high confidence in the reported results, 2^nd^ tier: moderate confidence in the reported results, or 3^rd^ tier: low confidence in the reported results). The placement of a study into a quality tier was based on the risk-of-bias ratings. Three critical risk-of-bias criteria, called “key criteria”, were given the most weight in determining the study quality. In experimental animal studies and human controlled trials, these key criteria were questions 3, 6, and 7. The ratings for the remaining criteria were given less weight in determining study quality. See S7 File for further details regarding study quality ratings and examples.

### Evidence synthesis and determination of confidence

Only 1^st^ and 2^nd^ tier studies were included in the narrative summaries and evidence synthesis. Heterogeneity within the included studies determined the appropriate type of analysis. Meta-analyses were not performed because studies were too heterogeneous regarding the investigated parameters and exposure characteristics to form sensible subgroups. For all subsets of studies, narrative syntheses of the available evidence were performed, as suggested by NTP [28]. The findings of the included studies were translated into categories of “effect found”/”no effect found” and presented together with the study quality according to OHAT criteria.

The confidence in the body of evidence was assessed according to the approach described in the OHAT guidelines, which is based on the Grading of Recommendations, Assessment, Development and Evaluation (GRADE) approach. Studies were first divided into topics according to the parameters examined and each topic was given an initial confidence rating depending on the study design of the included studies. This initial rating can be downgraded for factors that decrease confidence in the results (e.g., risk of bias and unexplained inconsistency), and it can be upgraded for factors that increase confidence in the results (e.g., large magnitude of effect, dose response, and consistency across study designs/populations). The evidence appraisal was conducted at endpoint level, i.e., separately for each topic. Four descriptors were used to indicate the level of confidence in the body of evidence: “high”, “moderate”, “low”, and “very low” confidence.

- High Confidence: The true effect is highly likely to be reflected in the apparent relationship.
- Moderate Confidence: The true effect may be reflected in the apparent relationship.
- Low Confidence: The true effect may be different from the apparent relationship.
- Very Low Confidence: The true effect is highly likely to be different from the apparent relationship.

Finally, according to OHAT, the confidence in the body of evidence was translated into five descriptors of the evidence for health effects using the confidence ratings and direction of the effect (“health effect” or “no health effect”): “high”, “moderate”, “low”, “evidence of no health effect”, and “inadequate evidence”. “High,” “moderate,” and “low” level of evidence directly translate from the ratings of confidence in the evidence (see above) in case the exposure was associated with a health effect. If only a “very low” or “no confidence” in the body of evidence is identified, the level of evidence was characterized as “inadequate evidence of health effect”. According to OHAT, the descriptor “evidence of no health effect” is used to indicate confidence that the exposure is not associated with a health effect. Because of the inherent difficulty in proving a negative effect, the conclusion “evidence of no health effect” was only reached when there was high confidence in the body of evidence.

We did not perform an evidence synthesis if a) only single studies were available for a specific topic, b) ≤ 3 studies were available and in addition, risk of bias was identified in these studies (i.e., 2^nd^ tier studies), or c) the studies lacked a sufficient homogeneity in terms of species, exposure and/or outcomes and, therefore, a meaningful comparison was not possible. If any of these conditions were met, we deemed it impossible to perform a meaningful evidence synthesis.

### Amendments compared to protocol

The frequency range was extended from 3–4 GHz and 20–30 GHz to 3–30 GHz. This option had already been considered in the protocol and was incorporated after it turned out that only 45 studies could have been included in the review with the more restricted frequency range. Moreover, cross-sectional studies were excluded due to the lack of temporality and the consequential difficulty in proving causal effects, i.e., the simultaneous assessment of exposure and outcome makes a causal effect difficult to establish. Further, any reported effects may be due to factors other than exposure to RF EMF, and cross-sectional studies do not control for potential confounding factors. The general limitations of cross-sectional studies are also reflected by OHAT classifying the certainty of evidence as “low” for cross-sectional studies in the initial confidence rating based on study design [28]. In addition, studies without exposure assessment of the individual (e.g., ecological studies) were also excluded from the review, as individual exposure in such studies is very uncertain.

### Classification of exposure in relation to limit values

To classify the results of the included studies with regard to the existing limit values, we categorized the exposure-outcome pairs of the included studies according to the limits recommended by the ICNIRP [7] (S9 File). However, this classification is subject to considerable uncertainties, because the ICNIRP limits are based on conditions in the human body and are not readily transferable to other species. Nevertheless, we aimed to distinguish studies with effects below the limits from studies with effects above the exposure limits and thus provide added value for the discussion on potential non-thermal effects. Thermal effects of RF EMF are generally accepted, well-documented, and can be harmful at certain levels of intensity. In cases where our systematic review includes studies in which exposure exceeds the limits, it is important to indicate, despite all the uncertainties, which studies this applies to. This will help to understand that such exposures are unlikely in everyday life. Otherwise, potential harmful effects of studies with exposures above the limits could be misinterpreted. If there were reasons for caution concerning the exposure categorization in individual studies, we commented on this appropriately in S9 File.

## Results

### Study selection

The systematic search in PubMed, Web of Science and EMF-Portal (S3 File) returned a total of 21,060 potentially relevant articles (Fig 1). After removal of 4,722 duplicates, 16,338 articles were screened in title and abstract, whereof 15,322 studies were excluded because they did not match the eligibility criteria (e.g., no experimental or epidemiological study). The full texts of the remaining 1,012 articles were obtained to check for their eligibility for inclusion in the current analysis. Of these, 909 articles were excluded for the following reasons: the exposure was not relevant (e.g., frequency outside the target range) or unclear (n = 760), the type of study was not relevant (e.g., review) or the study was not EMF/health-related (n = 91), there was no control group or the control conditions were unclear (n = 17), the article was not peer-reviewed (n = 13), it was a study investigating non-mammals or an *in vitro* study (n = 12), the article was not in English or German (n = 10), or other reasons (e.g., article not available) (n = 6). Some of these articles were excluded because they met more than one exclusion criterion. For reasons of clarity, only the most noteworthy reason for exclusion was documented in Fig 1. A list of all excluded articles, including bibliographic data and the reasons for their exclusion, is provided in S4 File. Due to additional non-systematic screening in the EMF-Portal after August 2023 (through October 2023), we identified four additional relevant articles [49–52]. Finally, 107 studies fulfilled the eligibility criteria and were included in this systematic review.

**Fig 1:**
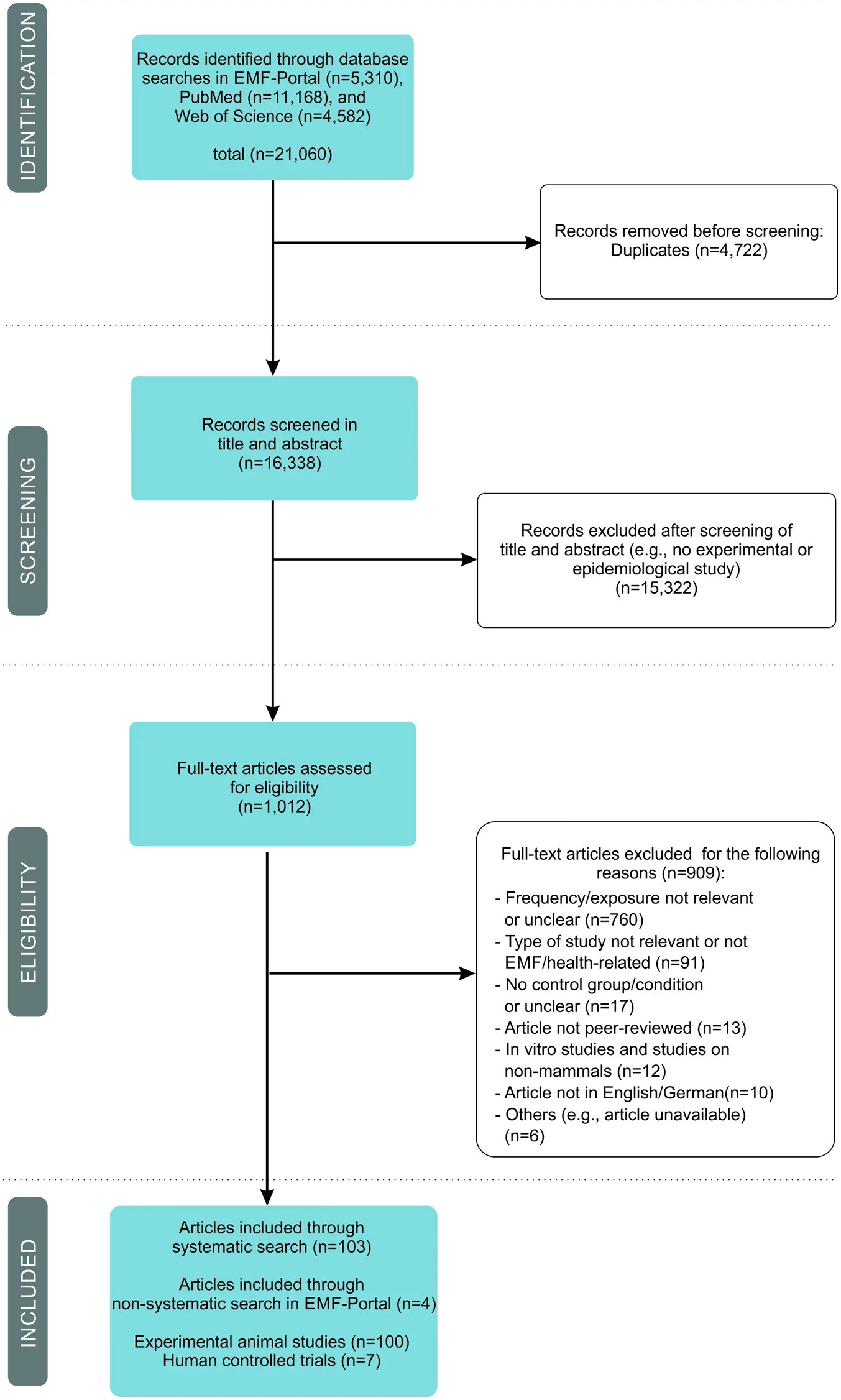
Flow diagram of literature search, eligibility and inclusion process according to PRISMA statement.

### Study appraisal

Key parameters of all studies that met the inclusion criteria were extracted based on a predefined extraction sheet (S5 File). Using the risk-of-bias tool recommended by OHAT [28,33] the internal validity and the quality of all these studies were assessed. Of the 107 included experimental studies, 11 studies (10%) were assigned to the 1^st^ tier, 39 studies (37%) to the 2^nd^ tier, and 57 studies (53%) to the 3^rd^ tier. Relevant domains and questions for risk-of-bias assessment are presented in S6 File, explanations and examples for assignment of studies to tiers are provided in S7 File, and risk-of-bias assessments of 1^st^ and 2^nd^ tier studies are shown in Fig 2 and of 3^rd^ tier studies in S8 File.

**Fig 2:**
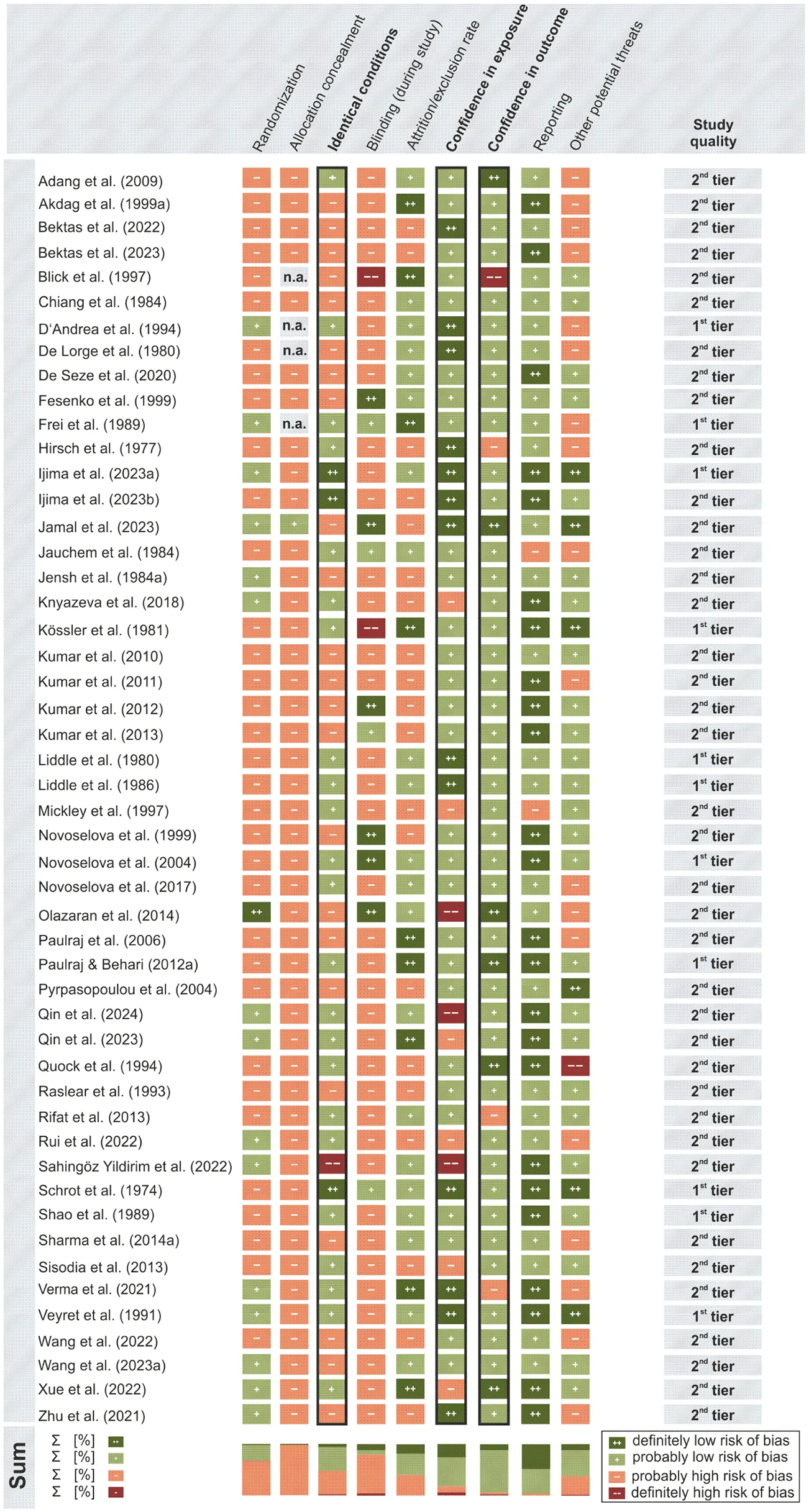
Risk-of-bias ratings for 1^st^ and 2^nd^ tier studies (n = 50). Criteria ratings served as the basis for the assignment of individual studies to the quality categories. Black frames indicate key risk-of-bias criteria.

In 1^st^ and 2^nd^ tier studies, methodological flaws were identified particularly in the criteria *Allocation concealment* and *Blinding of research personnel/subjects*. A total of 98% and 83%, respectively, of the studies were rated with a probably high risk of bias here, as no blinding measures were mentioned for these points. Furthermore, weaknesses were found in the criteria *Randomization of exposure level*, *Attrition/exclusion rate*, and *Other potential threats*. Among the three key criteria, a probably or definitely high risk of bias was most common in *Identical experimental conditions in all groups*, as in many cases the sham exposure condition was not or not sufficiently described, and thus identical experimental conditions could not be confirmed for all groups. In contrast, *Confidence in the exposure*, *Confidence in the outcome assessment*, and *Reporting of all measured outcomes* were largely rated with a low risk of bias, i.e., at least 82% of the 1^st^ and 2^nd^ tier studies had a probably or definitely low risk of bias in these criteria.

Most of the criteria of the 3^rd^ tier studies exhibited a probably or definitely high risk of bias.

### Summary of results and evidence synthesis

A total of 107 experimental studies were included in the review, but no epidemiological studies were identified that met the inclusion criteria. Of the included studies, 100 were experimental animal studies and seven were human controlled trials. The studies were divided into topics based on the focus of their research (e.g., effects on cognition, behavior, or male fertility). However, as the studies were often heterogeneous in terms of species, exposure and investigated parameters, this classification is only a rough approximation. Additionally, the evidence of the few human controlled trials (n = 7) was derived together with the evidence of experimental animal studies due to the similar study design and same initial confidence rating (high confidence). Only 1^st^ and 2^nd^ tier studies were included in the narrative summaries and evidence synthesis. The extracted data from 1^st^ and 2^nd^ tier studies are reported in Tables A to G in S1 Tables. Extracted data from all 3^rd^ tier studies are reported in S2 Table. A tabular overview of the evidence synthesis is provided in S10 File.

Most studies included in our synthesis focused on the effects on the central nervous system, cognition, and behavior (n = 31), and the immune system (n = 17). Further topics were effects on male reproduction, teratogenicity, skin, thermoregulation, eyes, and blood parameters. The seven human controlled trials investigated the central nervous system, cognition, and behavior (n = 4), thermoregulation (n = 1), and systemic effects/multiple parameters (n = 2). The most investigated frequencies were in a narrow range around 3–4 GHz and 10 GHz; frequencies above 20 GHz were examined in only five studies. Fig 3 provides an overview of all studies in the respective topics including information on study quality and the direction of the result (effect/no effect).

**Fig 3:**
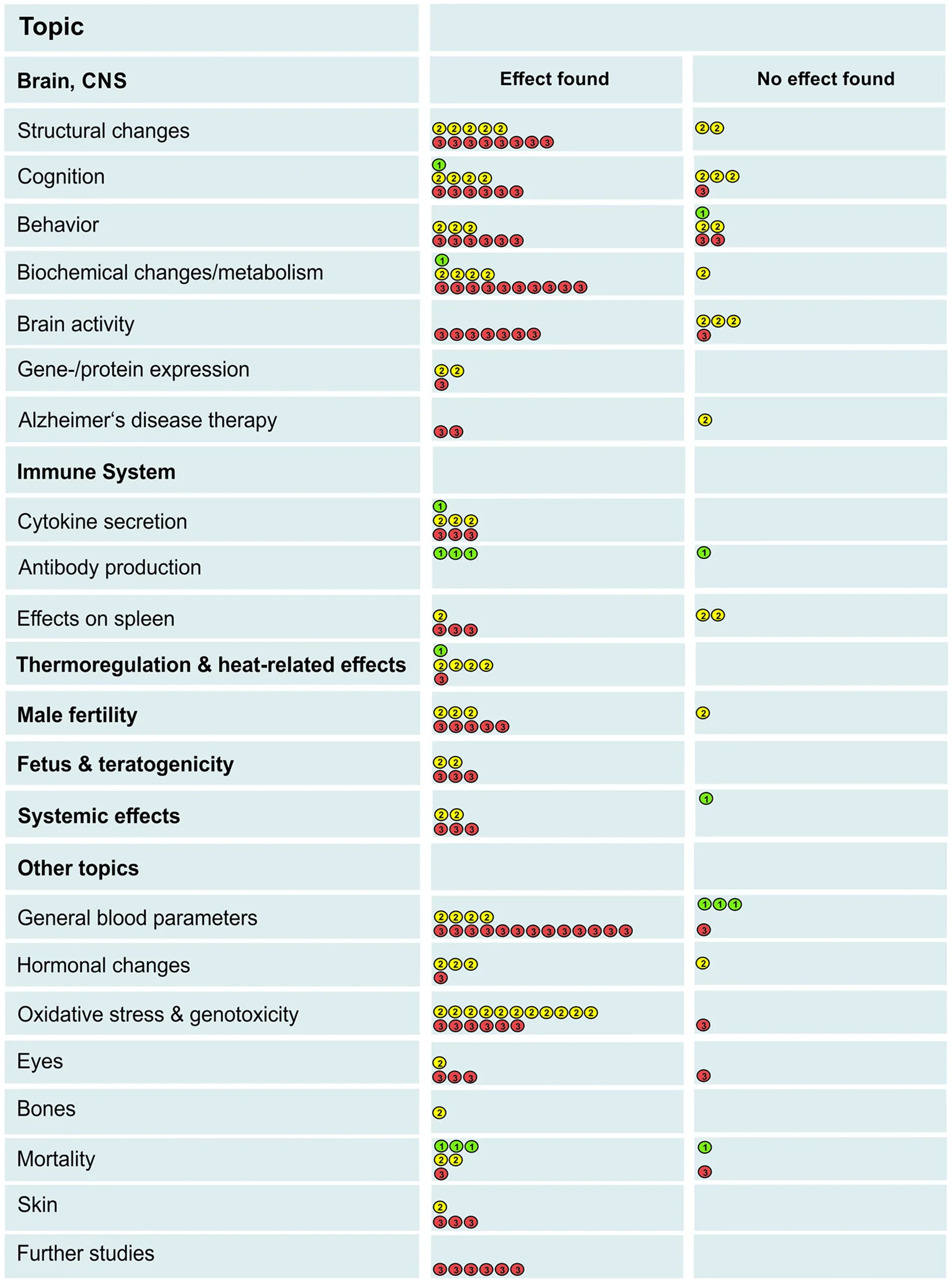
Overview of study quality and effects. Each study is represented by a numbered and colored circle indicating the OHAT study quality (1/green = 1^st^ tier, 2/yellow = 2^nd^ tier, 3/red = 3^rd^ tier). Studies which investigated more than one endpoint are listed separately in each column.

#### Central nervous system, cognition, and behavior.

##### Effects on cognition.

There were eight studies on cognitive functions (Table A in S1 Tables) and four of them revealed adverse effects due to exposure in monkeys, rats, and mice [53–56], i.e., the animals showed deteriorations in different cognitive tasks. In contrast, Rui et al. [57] and Wang et al. [52,58] found no effects on the spatial memory of rats after exposures and De Seze et al. [59] (see Systemic effects) observed an improvement in sensorimotor functions in rats after chronic exposure to pulsed fields.

In total, three studies found effects [54,56,59] and three studies found no effects above the limit values, two studies found effects below the limit values [53,55]. The parameters of the studies were highly heterogeneous except for effects on learning and spatial memory tested in the Morris water maze, which was investigated in four studies [52,56–58] so an evidence synthesis was only performed for this parameter indicating a direction towards no effect of exposure. The initial high confidence was downgraded two times due to unexplained inconsistency of the results and risk of bias (all 2^nd^ tier) resulting in low confidence in the body of evidence. Thus, the evidence is inadequate to draw a conclusion about any effects on learning and spatial memory.

##### Structural changes in the brain.

Seven studies investigated structural changes in the brain (Table A in S1 Tables), five of which found histological changes in the hippocampus of rats [52,56,60] or amygdala of mice [24,61]. In contrast, in the studies by Rui et al. [57] and Wang et al. [58], no structural changes in the brain of rats were found due to microwave exposure.

The seven included studies had a high initial confidence. Except for one study [60], all studies investigated exposures above the limit values. Although most of the investigated parameters were highly heterogenous in the seven studies, histological changes in the hippocampus were studied in six studies. Thereof, three studies found histological changes [52,56,60], but three [24,57,61] found no effects on hippocampus structure. The initial confidence was downgraded due to risk of bias (all 2^nd^ tier) and inconsistent results, which lead to a low evidence for an effect on hippocampal structure following exposure (above the limit values).

##### Effects on behavior.

Six studies investigated behavioral parameters in rodents (Table A in S1 Tables). De Lorge and Ezell [62] observed a consistent deterioration of vigilance after pulsed exposure and suspected thermal effects. Qin et al. [24] found a depressive effect in mice following 4.9 GHz (5G) exposure, however, this could not be confirmed by Qin et al. [61]. None of these studies found any effects on other behavioral parameters, such as spontaneous activity or exploratory behavior. De Seze et al. [59] observed that rats avoided an area with high-intensity RF EMF exposure. Kössler et al. [63] and Quock et al. [64] found no effects on different behavioral endpoints in mice after microwave exposure.

The studies were hardly comparable because of substantial differences in the endpoints. Therefore, we did not perform an evidence synthesis.

##### Biochemical changes/metabolism.

The six studies summarized in this category investigated changes in different biochemical parameters, such as oxidative stress or effects on energy metabolism (Table A in S1 Tables). Bektas et al. [60] found oxidative stress and changes in the brain energy balance in healthy and diabetic rats, but the authors were unable to draw a clear conclusion regarding the interaction with diabetes. Sharma et al. [53] also found an increase in oxidative stress and changes in some antioxidant enzyme activities in the brains of mice. Huai et al. [65] investigated the levels of two mitochondrial enzymes in the brains of mice and found reduced enzyme levels after pulsed 3 GHz exposure, which was interpreted as an effect on the energy metabolism of the brain. Mickley et al. [66] aimed to compare different heating methods (microwaves and hot air) on glucose metabolism in the brains of rats. The authors found a comparable increase in metabolism with both methods, concluding that microwaves affect brain metabolism through thermal effects. Paulraj and Behari [67] observed changes in calcium efflux and enzyme activities in the developing rat brain after pulsed 9.9 GHz exposure and concluded that this could affect brain development and function. In contrast, Zhu et al. [56] measured no changes in enzyme activities in the brain of rats after 4.3 GHz exposure.

The studies were hardly comparable because of substantial differences in the endpoints and risk of bias was common in all studies (five studies 2^nd^ tier, one study 1^st^ tier). Therefore, the conduct of an evidence synthesis was not reasonable.

##### Effects on brain activity.

Effects on brain activity, e.g., detected by electroencephalography (EEG), were investigated in three studies (Table A in S1 Tables) and none of them found any effects of exposure above or below the limit values [50,52,56]. Due to the low number of studies and the risk of bias (all 2^nd^ tier) identified, we did not perform an evidence synthesis.

##### Gene and protein expression in the brain.

In their study on newborn rats, Şahingöz Yildirim et al. [68] found changes in the expression of genes related to brain development after 10.7 GHz exposure. Wang et al. [52] observed changes in exosomal protein expression related to synaptic transmission function after 4.3 GHz exposure of rats as well as damage in the hippocampus. They concluded that both effects were related (see Table A in S1 Tables).

The two studies differed substantially in terms of the parameters investigated, and in addition, risk of bias was identified (both 2^nd^ tier). Thus, we deemed it not reasonable to perform an evidence synthesis.

##### Alzheimer’s disease therapy.

Olazaran et al. [69] investigated the effects of the so-called REAC (Radio-Electric Asymmetric Conveyer) device, which is intended to be used for the treatment of Alzheimer’s disease. No clear effect of a single 10.5 GHz pulse on the motor functions of patients with Alzheimer’s disease was found (Table A in S1 Tables). We did not perform an evidence synthesis for this single study (2^nd^ tier).

##### Genotoxicity in the brain.

Paulraj and Behari [70] found an increase in DNA single-strand breaks in the developing brain of rats after 16.5 GHz exposure (Table A in S1 Tables). Genotoxicity was also found in other studies unrelated to the central nervous system (see Other topics). We did not perform evidence synthesis for this single study.

#### Immune system.

##### Effects on cytokines.

Four studies from the same laboratory [71–74] with a similar exposure investigated the effect of microwaves on the secretion of various cytokines, in particular tumor necrosis factor (TNF), in mice (Table B in S1 Tables). In all four studies, an increase in cytokine production was described, indicating stimulation of the immune system. All studies found effects at exposures below the limit values. The initial high confidence was downgraded due to risk of bias (three of the studies 2^nd^ tier). Thus, a moderate confidence level and therefore moderate evidence for an effect on the secretion of various cytokines in mice was derived. However, this should be confirmed by an independent study by a different working group.

##### Antibody production.

Liddle et al. [75,76], Shao and Chiang [77], and Veyret et al. [78] investigated the effects of exposure on the antibody response in mice (Table B in S1 Tables). In all four studies, the antibody response was stimulated either by antigen administration or by bacterial infection. Veyret et al. [78] found that the antibody response could be enhanced or attenuated in mice when the pulse component of the microwave exposure was impressed with amplitude modulation. The relevance of these results for future therapeutic applications is to be tested in suitable biological models. Shao and Chiang [77] found an increased haemagglutinin titer and thus an enhanced immune response after pulsed exposure to 3 GHz. The authors concluded that short-term stimulation of the immune system is possible. Liddle et al. [75,76] investigated the effects of microwave exposure on bacterially stressed mice, particularly on antibody production and a potential activation of the immune system. In their first study [75], the animals survived for a longer period after exposure (PD 10 mW/cm²) and an increase in circulating antibodies was thought to be the cause. However, in their later study [76] with a ten times lower exposure intensity (PD 1 mW/cm²), the effect could not be confirmed. The authors therefore concluded that the threshold value for stimulation of the immune response is between 1 and 10 mW/cm².

Three of the four studies differed substantially in terms of exposure and antibodies tested, which made a meaningful evidence synthesis infeasible.

##### Effects on the spleen.

The three studies (Table B in S1 Tables) in this topic focused on general effects on the spleen [79] or T-cell proliferation in the spleen [71,72]. Rifat et al. [79] found various histopathological as well as biochemical changes in the spleen of mice after microwave exposure and an amelioration after administration of a plant extract. Studies by Fesenko et al. [71] and Novoselova et al. [72] found no significant effects on splenic T-cell proliferation. We deemed it inappropriate to perform an evidence synthesis due to the small number of studies and the presence of risk of bias (all 2^nd^ tier). Additionally, the studies differed substantially.

#### Thermoregulation and heat-related effects.

A total of five studies focused on thermoregulation and heat-related effects (Table C in S1 Tables), with Ijima et al. investigating thermoregulation [49,51], while Frei et al. [80] and Jauchem et al. [81] studied the changes in heart rate due to a temperature increase. Blick et al. [82] determined detection thresholds of thermal sensation.

Two recent studies by the same group of authors [49,51] analyzed the associations between the increase in skin and rectal temperature, thermoregulatory effects, and the exposure to RF EMF in the quasi-millimeter wave range (26.5 GHz and 28 GHz) in rats. Ijima et al. [51] found that dorsal skin temperature increased by approximately 11.3 °C at 500 W/m², although rectal temperature only increased by 0.6 °C, indicating highly localized effects. A significant correlation was found between the absorbed power density and the increase in skin temperature. From further calculations, the authors concluded that the threshold value from the ICNIRP guidelines [7] would be valid under the present experimental conditions (i.e., a temperature increase of less than 5 °C (4.32 °C) in the skin exposed to 200 W/m² APD). In their second study, concomitantly investigating skin blood flow [49], the authors also showed an intensity-dependent temperature increase in different parts of the body. In addition, the ratio of skin blood flow was altered by the increase in rectal temperature, resulting in an increase in tail skin temperature. Therefore, the authors concluded that whole-body exposure to quasi-millimeter waves drives thermoregulation to transport the heat generated by the exposure to the body surface.

Both studies found effects above the limit values and thermal effects are in line with known mechanisms of action. However, whether the results reflect the real extent of the thermal effect needs to be confirmed by an independent study. We did not perform evidence synthesis for these two studies due to the low number of studies and the presence of risk of bias in one of them.

The two studies by Frei et al. [80] and Jauchem et al. [81] focused on cardiovascular changes in rats following a 1 °C increase in body temperature induced by microwave exposure. Jauchem et al. [81] found that the changes in heart rate during 5.6 GHz exposure were related to the average power density and thus to the rate of temperature change and not simply to the absolute temperature change. In addition, the same group of authors showed in their later study [80] that the time required to achieve a 1 °C increase in colonic temperature varied inversely with the power density (at 30 mW/cm² approximately three times longer than at 60 mW/cm²). Moreover, they found that at both exposure levels, the heart rate increased significantly during exposure and returned to baseline when exposure was discontinued. Thus, in both studies, cardiovascular effects were observed in association with changes in body temperature of 1 °C.

Both studies found effects above the limit values and thermal effects are in line with known mechanisms of action (see above). We did not perform evidence synthesis for these two studies due to the low number of studies and the presence of risk of bias in one of them.

Blick et al. [82] investigated the warmth sensation in the skin of human volunteers in a frequency range from 2.45 GHz to 94 GHz and found an increasing sensitivity with increasing frequencies, determining detection thresholds around 19.5 mW/cm² for 7.5 and 10 GHz.

Besides the studies mentioned, other groups of authors not explicitly investigating heat-related effects hypothesized that the effects revealed in their studies were probably related to temperature increases induced by exposure as well [66,83,84].

#### Effects on male fertility.

Four studies investigated the effects of microwave exposure on male fertility (Table D in S1 Tables). Three studies by Kumar et al. investigated the effects of 10 GHz exposure on male fertility in a group of six rats. It is not clear whether the same six rats were used for all three studies. In their first study, Kumar et al. [85] found a reduced histone kinase activity, increased levels of reactive oxygen species (ROS), and apoptosis in sperms after exposure, which the authors interpreted as an effect on spermatogenesis and the cell cycle in sperms. In subsequent studies, the authors also observed an effect on male fertility, which could have been caused by oxidative stress [86] or DNA damage [87]. In contrast, Xue et al. [88] found no effect on sperm count or sperm abnormalities after exposure to 5.8 GHz, nor on histological testicular structure or sex hormones.

The studies differed substantially in terms of the endpoints investigated and methods, and risk of bias (all 2^nd^ tier) and imprecision were also identified. Thus, we deemed it inappropriate to perform evidence synthesis.

#### Effects on fetus and teratogenicity.

Two studies [89,90] investigated the effects of *in utero* exposure on fetal development in rats (Table E in S1 Tables). Jensh [89] observed a decreased fetal growth and thus concluded a slight teratogenic effect of 6 GHz exposure. Pyrpasopoulou et al. [90] investigated the expression of bone morphogenetic proteins (BMP) in the kidneys, playing an important role as growth factors for the development of the locomotor system. After a 9.4 GHz exposure, they found an altered BMP expression and localization in the newborns, which could be attributed to the effect of the exposure on gene expression in the early gestational phase.

The two studies differed substantially in terms of the endpoints investigated, and risk of bias was identified (both 2^nd^ tier). Thus, evidence synthesis was not performed.

#### Systemic effects.

There were three studies that used a comprehensive approach to investigate the effects of microwaves on the general health of rodents (Table F in S1 Tables). The only 1^st^ tier study found no effects on behavior, blood parameters, and enzyme activities [63]. In contrast, Adang et al. [91] reported changes in blood parameters and a reduced survival rate after long-term exposure to 9.7 GHz microwaves. However, the study has been criticized by other authors for several shortcomings and the conclusions drawn by the authors may not be supported by the data [92]. The aim of the study by de Seze et al. [59] was to investigate the effects of high-power microwaves used to disrupt the electronics of military and civilian vehicles. They found changes in cognitive and sensorimotor functions in rats, as well as an increase in cancer prevalence, and a decrease in survival time.

As the studies in this paragraph investigated multiple parameters, we did not perform evidence synthesis. However, the parameters examined in these studies were considered in the appropriate sections mentioned above.

#### Other topics.

##### General blood parameters.

Sisodia et al. [93] revealed several changes in hematological, biochemical, and histopathological blood parameters in mice exposed to 10 GHz microwaves (Table G in S1 Tables). Changes in blood parameters were also observed in other studies, primarily as an additional measurement to assess the effects of exposure on different organ systems. Kumar et al. [86] and Adang et al. [91] observed changes in various blood parameters in rats following exposure. In Jensh [94] the maternal monocyte count was lower in exposed pregnant animals. The remaining studies [63,75,76] did not find significant effects on the hematological parameters considered. The studies included in this topic are very heterogeneous and hardly comparable. Therefore, we did not perform an evidence synthesis.

##### Hormonal changes.

Two studies investigated hormonal changes (Table G in S1 Tables). Akdag et al. [83] found that the levels of various hormones (e.g., follicle-stimulating hormone, estradiol) were affected in rats after exposure, probably due to thermal stress. Knyazeva et al. [95] found changes in serum corticosterone level after repeated exposure to microwave pulses depending on the frequency of the repetition rate (decrease or increase). However, the relevance of the exposure was not elucidated.

Hormonal changes were also investigated in two studies from other topics [61,88]. One of these studies showed a decrease in serotonin levels in mice after exposure [61]. As the four studies in this topic examined different organ systems and hormones, the conduct of an evidence synthesis was not reasonable.

##### Oxidative stress and genotoxic effects.

Kumar et al. (2^nd^ tier) [96] investigated the oxidative imbalance due to microwave exposure and identified increased serum ROS levels and genotoxicity (micronucleus formation) in red blood cells of rats (Table G in S1 Tables).

Oxidative stress parameters were also analyzed in eight studies on other topics. In most of these studies, oxidative stress was investigated in addition to other investigations and was not usually the main focus. Changes in oxidative stress parameters were identified in all eight studies. However, the studies differed substantially in their study focus investigating, e.g., effects on the brain [53], the spleen [79], the blood [93], the male reproductive system [85,86], and the skin [97]). Additionally, Bektas et al. [60] found changes in oxidative stress parameters in the brains of healthy and diabetic rats, while Bektas et al. [98] observed such changes in their muscles.

The studies were very heterogeneous and hardly comparable. Therefore, we did not perform evidence synthesis.

In addition to Kumar et al. [96] finding genotoxicity in red blood cells, genotoxic effects were also observed in the brain [70] (see Central nervous system, cognition, and behavior) as well as in the blood and spermatozoa of rats [87] (see Effects on male fertility) following exposure. The studies differed substantially in terms of the investigated organ system, and risk of bias was also identified. Thus, evidence synthesis was not performed.

##### Effects on eyes.

Hirsch et al. [84] found that exposure to 3 GHz and PD ≥ 300 mW/cm² (above limit values) damaged the iris of rabbits and produced cataracts, and that cataract may be caused due to increased temperature levels (Table G in S1 Tables). We did not perform evidence synthesis for this single study. However, eyes are considered temperature-sensitive organs and damage to the eyes at field strengths above the limit values is generally plausible.

##### Effect on bones.

Bektas et al. [98] showed that exposure to 3.5 GHz below the limit values had adverse effects on bone biomechanics and could induce muscle oxidative stress parameters in healthy and diabetic rats (Table G in S1 Tables). However, the background of this study or possible mechanisms of action were not further elucidated. We did not perform evidence synthesis for this single study.

##### Mortality.

In the study by Schrot and Hawkins [99], rats were killed by exposure to 3 GHz of varying field strengths and exposure durations and the LD_50_ was determined (Table G in S1 Tables). Although the article should have been excluded due to the exclusion criteria of the review, we nevertheless included the article because the LD_50_ is an internationally recognized parameter in toxicology and could be of general interest for the present systematic review. However, the article was not considered for evidence synthesis. The authors found that the mean lethal power density varied between 2.36 W/cm² and 0.42 W/cm² for exposure durations between 30 s and 240 s.

Liddle et al. [75,76] and Novoselova et al. [74] investigated whether EMF exposure could have a “positive”, i.e., life-prolonging effect in experimentally diseased animals. Liddle et al. investigated whether microwave exposure could influence bacterially stressed mice and found in their first study [75] that the animals survived for a longer period of time, probably due to stimulation of the circulating antibody response. However, in their later study [76] with a ten times lower exposure strength, the effect could not be confirmed. Novoselova et al. [74] found in animals with experimental tumors that exposure increased survival time and decreased tumor growth rate, probably mediated by TNF production. The studies by Liddle et al. [75,76] differed substantially from the study by Novoselova et al. [74] in terms of the investigated mouse model. Thus, we deemed it inappropriate to combine the three studies in a meaningful evidence synthesis and could not draw a conclusion on whether there may be positive effects of microwave exposure in experimentally diseased animals.

De Seze et al. [59] and Adang et al. [91] investigated systemic effects in rats, and both found, among other things, reduced survival after the respective exposures (see Systemic effects). Because only two studies were available for this topic and, in addition, risk of bias was identified (both 2^nd^ tier), we deemed it inappropriate to perform a meaningful evidence synthesis.

##### Effects on the skin.

Verma et al. [97] investigated the effects of 10 GHz exposure (above the limit values) on various parameters in the skin of rats as the first entry point for environmental noxious agent into the body (Table G in S1 Tables). They found significant changes in molecular markers in the skin of rats associated with the adaptive stress response (oxidative stress, inflammatory responses, metabolic changes) after 10 mW/cm² exposure, but no changes after 5 mW/cm² exposure. We did not perform evidence synthesis for this single study. Nevertheless, damage to the skin at field strengths above the limit values is generally plausible.

## Discussion

### Summary of evidence

In this systematic review, we summarized the current knowledge on the effects of exposure to 3–30 GHz EMFs. For this purpose, we evaluated the outcomes of 107 experimental human and animal studies and critically appraised the internal validity of individual studies. In summary, for most topics, the current scientific data were inadequate to draw any conclusions about the physiological and health-related effects of RF EMF in the investigated frequency range (3–30 GHz). However, moderate evidence below the limits values was found for cytokine secretion in mice. Low evidence was found for structural changes in the hippocampus of rats above the limit values. There was a large heterogeneity regarding the applied frequencies, field strengths, exposure durations, the examined endpoints, and exposed species.

Furthermore, we identified methodical flaws in most studies, which lowered the credibility in the reported results. An additional analysis revealed that high quality studies tended to report less effects, while moderate and low-quality studies were more likely to report biological effects of exposure (Fig 3). Of all 1^st^ tier studies, 59% reported an effect of exposure. In contrast, 76% of all 2^nd^ tier studies and 90% of all 3^rd^ tier studies reported biological effects of exposure. In the latter studies, it is possible that observed effects were not caused by EMF exposure per se but by factors that were not sufficiently controlled by an appropriate experimental design.

### Review limitations

A limitation of the present systematic review is that the search terms used in identifying relevant journal articles may not have been found in the title, abstract, or keywords of certain articles. Moreover, we only considered peer-reviewed articles written in English or German. Therefore, potentially relevant data from articles published in other languages or data from gray literature (data that are not published in scientific journals) were not included.

Many studies (n = 909, S4 File), which had initially been classified as potentially relevant during the screening step, were excluded from the review in the eligibility step. The main reason for this was that the frequency of the RF EMF or the type of exposure did not meet the inclusion criteria (n = 761). This means that a study was excluded, if, e.g., it examined a frequency spectrum that was partly outside the target spectrum of this review (3–30 GHz) and if there was no separate evaluation for the target frequency range. For example, Perov et al. [100] investigated 5G exposure of rats at simultaneous frequencies of 3.6 GHz, 28 GHz, and 37 GHz. They found changes in the levels of stress hormones and concluded a stress-inducing effect of multifrequency exposure. However, we excluded this study because a frequency outside 3–30 GHz was also investigated. Some epidemiological studies also fell under this exclusion criterion, as they did not carry out a separate evaluation for 5G, but only evaluated the effects of mobile communications in general [101,102]. Overall, we did not identify any epidemiological studies that fulfilled our eligibility criteria. However, this was to be expected for the following reasons: 1) 5G technology has only recently been introduced; 2) epidemiological studies, especially prospective cohort studies in the field of mobile communications, are usually conducted over a long period of time due to the long latency period of, e.g., brain tumors (the main area of interest) and other health effects; and 3) there are almost no new (or ongoing) projects investigating the health effects of mobile phone exposure in epidemiological studies. Finally, all studies that deliberately investigated co-exposures to RF EMF and non-EMF exposures were excluded, as synergistic effects cannot be excluded in these cases [103]. All these studies were not included in the review due to the exclusion criteria specified in the study protocol but could potentially provide additional information on the effect of 5G-relevant exposure. The same applies to animal studies with non-mammals and *in vitro* studies.

As the OHAT Risk-of-Bias tool was used to assess the risk of bias in each study as a whole, in some cases, the true risk of bias may not be presented for each parameter evaluated. This could apply, for example, to the criterion “Confidence in the outcome assessment” if the evaluation was blinded for one part of the results but not for another. In such a case, a conservative approach was taken, and the criterion as a whole was assessed as “not blinded”. This may have led to an over- or underestimation of the risk of bias in single studies.

Only five of the 50 studies included in the evidence synthesis explicitly stated that they were investigating 5G exposure [24,50,60,61,98]. Although the RF signals used in these studies were at frequencies used in 5G networks, they did not represent typical 5G modulation patterns but were either continuous wave or (GSM-like) pulse modulated. The remaining studies either used therapeutic applications, high-power microwaves, radar exposures or investigated the effects of microwave exposure without focusing on any specific application. Therefore, it is important to note that the exposure scenarios of the included studies might only have limited significance for real 5G exposures due to the different modulations and field strengths investigated. According to Kostoff et al. [103], the biological effects of 5G-relevant exposures may depend not only on the carrier frequency, but also on various other factors, such as signal modulation and technical features (e.g., beamforming or massive Multiple Input Multiple Output (MIMO)). Additionally, it must be kept in mind that our systematic review is focused on a narrow frequency range of the currently most common 5G bands and allows no conclusion on future 5G applications in the millimeter frequency range. Further, the numerous exposure scenarios that a person may encounter in everyday life are not all covered by the included studies. These scenarios may consist of a combination of exposure frequency and type (partial body vs. whole body), resulting in different target organs potentially being impacted. In most studies, whole-body exposure was investigated, whereas only few studies considered localized head exposure.

Finally, recent publications issued after the end of the search (August 2023) were not considered for inclusion in the review (e.g., [104–107]; see Newly published articles since the last systematic search).

### Newly published articles since the last systematic search

Our last systematic search was conducted at the end of August 2023 and four additional articles were identified through supplementary unsystematic searches conducted till the end of October 2023 (see Study selection). An additional systematic search in the EMF-Portal (S11 File) was conducted on 18 March 2026 to identify newly published relevant studies due to the long interval between the extraction and appraisal period of already included studies (October 2024), manuscript submission (April 2025), and revision (March 2026). The search string used in the EMF-Portal is provided in S11 File. Screening for eligibility of relevant articles was conducted in two stages (titles/abstracts and full text) by two authors (ASK, LM). As expected, numerous new studies have been published in the meantime because of research initiatives following the introduction of 5G technology.

Overall, 28 additional relevant articles were identified (S12 File). Five studies investigated effects on humans, while the remaining 23 examined effects on animals. Compared with the first systematic search, which included 7 human studies and 100 animal studies (see Summary of results and evidence synthesis), this indicates a relative increase in the proportion of human studies in more recent research. Most studies (n = 19) investigated EMF exposure at frequencies ≤ 6 GHz. Five studies examined frequencies between 24–28 GHz [108–112] and one study covered both frequency ranges [113]. Additionally, three studies examined frequencies between 9.3 and 10 GHz [114–116].

17 of the 28 relevant articles, including all human studies, explicitly stated that they investigated 5G exposure. However, not all of them used a 5G modulation scheme for the applied RF signal, e.g., three studies conducted by the same research group investigated GSM-modulated 3.5 GHz exposure, which is not representative of 5G exposure [117–119]. The remaining studies either applied therapeutic approaches [114,120], investigated high-power microwave exposure [115,121], examined exposure to a MIMO router [122], or addressed EMF exposure without reference to 5G or any other specific application [116,123,124].

Three of the five studies investigating 5G exposure to humans were conducted by the same group. No significant effects of 5G exposure were observed on electrical brain activity measured by EEG [110] or on salivary stress biomarkers [111]. In a third study, effects on the autonomic nervous system were observed, including small changes in skin temperature and electrodermal activity; however, these changes remained within normal physiological ranges [105]. The two remaining human studies also focused on brain-related outcomes. One study reported changes in sleep EEG patterns in individuals with a specific genotype [125] and another study found no effects on cortical excitability [126].

Five of the 12 animal studies with explicit 5G exposure investigated effects on the brain (e.g., gene and protein expression) and behavior [127–131]. Two studies examined effects on the male reproductive system [113,132] and two additional studies assessed effects on the offspring of previously exposed animals [133,134].

The remaining three animal studies investigated thresholds for eye damage in rabbits [108], effects on skin in rats [109] and stress biomarkers in rats [112].

The newly identified studies were not extracted and appraised in detail and were not included in our evidence synthesis. Therefore, it is not possible to determine whether these additional studies confirm or alter the overall evidence synthesis. A detailed appraisal and synthesis may be performed in a future update.

### Research needs and ongoing projects

The present systematic review identified clear weaknesses in the study quality (risk of bias). To achieve higher validity and accurate reporting of results, we suggest that the bias criteria defined by OHAT should be considered when planning future studies [28,33]. Vijayalaxmi [135] has also provided a detailed guide and checklists for well-controlled study design and publication standards in EMF research. To ensure an adequate exposure characterization and dosimetry, we recommend following the guidelines by Kuster and Schönborn [136], Valberg [137], and Makinistian et al. [138] and considering the aspects and pitfalls discussed.

Moreover, particular attention should be paid to experimental studies on the effects on the skin and eyes, on realistic 5G exposure scenarios considering different field strengths, as well as epidemiological studies with separate consideration of 5G (if possible) in future studies. However, conducting future case-control studies may be difficult as most people already use mobile phones today, which reduces the number of suitable control subjects without exposure. Any potential health-related effects could be elucidated by future cohort studies with improved exposure assessment, i.e., with objective prospective exposure data. Cross-sectional studies with subjective exposure data should be avoided as confidence in the evidence provided is low.

In response to the generally recognized need for research, four major EU projects on 5G have been initiated in the past (ETAIN [139], GOLiAT [140], NextGEM [141], SEAWave [142]), which work together in the CLUE-H network [143]. In these projects, among other objectives, the effects on the skin and improved exposure assessment are to be investigated. Depending on the outcome of these projects further research needs may be derived.

## Conclusion

A total of 100 experimental animal studies and seven human controlled studies were systematically researched, analyzed and evaluated in this review. No epidemiological studies could be included. Most studies investigated exposures ≤ 10 GHz (n = 95). Seven studies investigated the frequency range 11–20 GHz and five studies investigated exposures > 20 GHz. A total of 57 studies had a high risk of bias (3^rd^ tier) and were therefore not included in the narrative or evidence synthesis. A total of 39 studies had several methodological weaknesses that limited the internal validity of the results (2^nd^ tier). Only eleven studies showed a low risk of bias (1^st^ tier). Overall, most studies found an effect of RF EMF exposure. However, a tendency was observed that studies with a high risk of bias were more likely to find an effect than studies with a low risk of bias. In summary, for most topics, the current scientific data were inadequate to draw any conclusions about the physiological and health-related effects of RF EMF in the investigated frequency range (3–30 GHz). However, moderate evidence below the limits values was found for cytokine secretion in mice. Low evidence was found for structural changes in the hippocampus of rats above the limit values. Although thermal effects are the only biological effect of RF EMF at frequencies above 10 MHz that is scientifically accepted, they were hardly investigated systematically in the studies included in this review. Nevertheless, many of the study authors suspect that thermal effects are the main effect in their studies. We recommend further high-quality research. Particular attention should be paid to experimental studies on effects on the skin and eyes, realistic 5G exposures considering different field strengths, as well as epidemiological studies with separate consideration of 5G (if possible).

## Supporting information

S1 File Search Strings. Links to search strings for review search in EMF-Portal and PubMed.
(PDF)

S2 File Extraction Table. Extraction table of previous reviews about 5G exposure-relevant effects.
(PDF)

S3 File Search Strings. Links to search strings in EMF-Portal, PubMed, and Web of Science.
(PDF)

S4 File Excluded Articles. Excluded articles with reasons for their exclusion after title/abstract screening.
(PDF)

S5 File Extraction Parameters. Extracted study parameters.
(PDF)

S6 File Risk of Bias. Risk-of-Bias domains and questions according to OHAT.
(PDF)

S7 File Example of Study Appraisal. Placement of studies into one of three quality categories (1^st^, 2^nd^, 3^rd^ tier).
(PDF)

S8 File Risk-of-Bias Figure. Risk-of-bias ratings for 3^rd^ tier studies.
(PDF)

S9 File Exposure. Comparison of exposure metrics with ICNIRP limits (1^st^ and 2^nd^ tier studies).
(PDF)

S10 File Evidence Synthesis. Assessment of confidence in the body of evidence synthesis on topic level.
(PDF)

S11 File Search String. Links to search string for search in EMF-Portal for newly published articles.
(PDF)

S12 File Reference List. Reference list of newly identified articles.
(PDF)

S13 File Reference List. References of Supplement information (S2, S8, S9, S15, S1 Tables, S2 Table).
(PDF)

S1 Checklist PRISMA checklist for systematic reviews. (PDF)

S1 Tables Extracted study data (1^st^ and 2^nd^ tier studies). (PDF)

S2 Table Extracted study data (3^rd^ tier studies). (PDF)

## References

[1] Calin MD, Ursachi C, Helerea E. Electromagnetic environment characteristics in an urban area. In: 2013 4th International Symposium on Electrical and Electronics Engineering (ISEEE), 2013. 1–6. 10.1109/iseee.2013.6674381

[2] Gajšek P, Ravazzani P, Wiart J, Grellier J, Samaras T, Thuróczy G. Electromagnetic field exposure assessment in Europe radiofrequency fields (10 MHz-6 GHz). J Expo Sci Environ Epidemiol. 2015;25(1):37–44. doi: 10.1038/jes.2013.40 23942394

[3] Kljajic D, Djuric N. Comparative analysis of EMF monitoring campaigns in the campus area of the University of Novi Sad. Environ Sci Pollut Res Int. 2020;27(13):14735–50. doi: 10.1007/s11356-020-08008-8 32052328

[4] Kurnaz C, Mutlu M. Comprehensive radiofrequency electromagnetic field measurements and assessments: A city center example. Environ Monit Assess. 2020;192(6):334. doi: 10.1007/s10661-020-08312-3 32382839

[5] SCENIHR (Scientific Committee on Emerging and Newly Identified Health. Potential health effects of exposure to electromagnetic fields (EMF). European Commission, Luxembourg. [Publications Office]; 2015.

[6] Urbinello D, Joseph W, Verloock L, Martens L, Röösli M. Temporal trends of radio-frequency electromagnetic field (RF-EMF) exposure in everyday environments across European cities. Environ Res. 2014;134:134–42. doi: 10.1016/j.envres.2014.07.003 25127524

[7] International Commission on Non-Ionizing Radiation (ICNIRP). Guidelines for limiting exposure to electromagnetic fields (100 kHz to 300 GHz). Health Phys. 2020;118:483–524. doi: 10.1097/HP.0000000000001210 32167495

[8] IEEE. IEEE Standard for Safety Levels with Respect to Human Exposure to Electric, Magnetic, and Electromagnetic Fields, 0 Hz to 300 GHz. IEEE Std C951-2019 (Revision of IEEE Std C951-2005/ Incorporates IEEE Std C951-2019/Cor 1-2019). IEEE. 2019. doi: 10.1109/IEEESTD.2019.8859679

[9] Belpomme D, Hardell L, Belyaev I, Burgio E, Carpenter DO. Thermal and non-thermal health effects of low intensity non-ionizing radiation: An international perspective. Environ Pollut. 2018;242(Pt A):643–58. doi: 10.1016/j.envpol.2018.07.019 30025338

[10] Belyaev IY. Dependence of non-thermal biological effects of microwaves on physical and biological variables: implications for reproducibility and safety standards. Non-thermal effects and mechanisms of interaction between electromagnetic fields and living matter. 1st ed. 2010. 187–217.

[11] Bortkiewicz A, Gadzicka E, Szymczak W. Mobile phone use and risk for intracranial tumors and salivary gland tumors - A meta-analysis. Int J Occup Med Environ Health. 2017;30(1):27–43. doi: 10.13075/ijomeh.1896.00802 28220905

[12] Hardell L, Carlberg M, Söderqvist F, Mild KH. Case-control study of the association between malignant brain tumours diagnosed between 2007 and 2009 and mobile and cordless phone use. Int J Oncol. 2013;43(6):1833–45. doi: 10.3892/ijo.2013.2111 24064953 PMC3834325

[13] Jazyah YH. Thermal and nonthermal effects of 5 G radio-waves on human’s tissue. ScientificWorldJournal. 2024;2024:3801604. doi: 10.1155/2024/3801604 39105157 PMC11300075

[14] Hu C, Zuo H, Li Y. Effects of radiofrequency electromagnetic radiation on neurotransmitters in the brain. Front Public Health. 2021;9:691880. doi: 10.3389/fpubh.2021.691880 34485223 PMC8415840

[15] Kundi M, Mild K, Hardell L, Mattsson M-O. Mobile telephones and cancer--A review of epidemiological evidence. J Toxicol Environ Health B Crit Rev. 2004;7(5):351–84. doi: 10.1080/10937400490486258 15371240

[16] Sciorio R, Tramontano L, Esteves SC. Effects of mobile phone radiofrequency radiation on sperm quality. Zygote. 2022;30(2):159–68. doi: 10.1017/S096719942100037X 34384508

[17] Nyberg NR, McCredden JE, Weller SG, Hardell L. The European Union prioritises economics over health in the rollout of radiofrequency technologies. Rev Environ Health. 2022;39(1):47–64. doi: 10.1515/reveh-2022-0106 36129168

[18] Rowley J, Mazar H. Misunderstandings about radiofrequency electromagnetic field exposure and 5G misinformation. In: 2021 IEEE International Conference on Microwaves, Antennas, Communications and Electronic Systems (COMCAS), 2021. 219–21. 10.1109/comcas52219.2021.9629063

[19] Frith J, Campbell S, Komen L. Looking back to look forward: 5G/COVID-19 conspiracies and the long history of infrastructural fears. Mob Media Commun. 2023;11(2):174–92. doi: 10.1177/20501579221133950 38603387 PMC9623406

[20] EMF-Portal. https://www.emf-portal.org/en. Accessed 2026 April 16.

[21] EMF-Portal | Study overviews, Experimental studies on mobile communications. [cited 16 Apr 2026]. https://www.emf-portal.org/en/article/overview/mobile-communications-med-bio

[22] EMF-Portal | Study overviews, Studies on millimeter waves. [cited 16 Apr 2026]. https://www.emf-portal.org/en/article/overview/millimeter-waves/medical-biological

[23] European 5G Observatory. 5G Observatory report 2025. 2025. https://digital-strategy.ec.europa.eu/en/policies/5g-observatory-2025

[24] Qin T-Z, Wang X, Du J-Z, Lin J-J, Xue Y-Z, Guo L, et al. Effects of radiofrequency field from 5G communications on the spatial memory and emotionality in mice. Int J Environ Health Res. 2024;34(1):316–27. doi: 10.1080/09603123.2022.2149708 36413628

[25] Gallucci S, Fiocchi S, Bonato M, Chiaramello E, Tognola G, Parazzini M. Exposure Assessment to Radiofrequency Electromagnetic Fields in Occupational Military Scenarios: A Review. Int J Environ Res Public Health. 2022;19(2):920. doi: 10.3390/ijerph19020920 35055741 PMC8776107

[26] Gupta AK, Venkataraman M, Joshi LT, Cooper EA. Potential use of microwave technology in dermatology. J Dermatolog Treat. 2022;33(7):2899–910. doi: 10.1080/09546634.2022.2089333 35699665

[27] PubMed. https://pubmed.ncbi.nlm.nih.gov/. Accessed 2026 April 16.

[28] NTP (National Toxicology Program) U.S. Department of Health and Human Services. Handbook for Conducting a Literature-Based Health Assessment Using OHAT Approach for Systematic Review and Evidence. 2019. https://ntp.niehs.nih.gov/go/ohathandbook

[29] Page MJ, McKenzie JE, Bossuyt PM, Boutron I, Hoffmann TC, Mulrow CD, et al. The PRISMA 2020 statement: An updated guideline for reporting systematic reviews. BMJ. 2021;372:n71. doi: 10.1136/bmj.n71 33782057 PMC8005924

[30] Shamseer L, Moher D, Clarke M, Ghersi D, Liberati A, Petticrew M, et al. Preferred reporting items for systematic review and meta-analysis protocols (PRISMA-P) 2015: Elaboration and explanation. BMJ. 2015;349(jan02 1):g7647–g7647. doi: 10.1136/bmj.g7647 25555855

[31] Moher D, Shamseer L, Clarke M, Ghersi D, Liberati A, Petticrew M, et al. Preferred reporting items for systematic review and meta-analysis protocols (PRISMA-P) 2015 statement. Syst Rev. 2015;4(1):1. doi: 10.1186/2046-4053-4-1 25554246 PMC4320440

[32] Bodewein L, Dechent D, Emonds T, Graefrath D, Krabbe J, Scheelhaase PJ, et al. Physiological and health-related effects of 5G-relevant radiofrequency fields (3–30 GHz): a systematic review of experimental and epidemiological studies in humans and non-human mammals. PROSPERO. 2022. https://www.crd.york.ac.uk/PROSPERO/view/CRD42022374434 10.1371/journal.pone.0361104 PMC13734471 42981375

[33] NTP (National Toxicology Program) U.S. Department of Health and Human Services. OHAT Risk of Bias Rating Tool for Human and Animal Studies. 2015. https://ntp.niehs.nih.gov/go/riskbias

[34] Bodewein L, Dechent D, Graefrath D, Kraus T, Krause T, Driessen S. Systematic review of the physiological and health-related effects of radiofrequency electromagnetic field exposure from wireless communication devices on children and adolescents in experimental and epidemiological human studies. PLoS One. 2022;17(6):e0268641. doi: 10.1371/journal.pone.0268641 35648738 PMC9159629

[35] Driessen S, Bodewein L, Dechent D, Graefrath D, Schmiedchen K, Stunder D, et al. Biological and health-related effects of weak static magnetic fields (≤ 1 mT) in humans and vertebrates: A systematic review. PLoS One. 2020;15(6):e0230038. doi: 10.1371/journal.pone.0230038 32516313 PMC7282627

[36] Petri A-K, Schmiedchen K, Stunder D, Dechent D, Kraus T, Bailey WH, et al. Biological effects of exposure to static electric fields in humans and vertebrates: A systematic review. Environ Health. 2017;16(1):41. doi: 10.1186/s12940-017-0248-y 28416002 PMC5393013

[37] Schmiedchen K, Petri A-K, Driessen S, Bailey WH. Systematic review of biological effects of exposure to static electric fields. Part II: Invertebrates and plants. Environ Res. 2018;160:60–76. doi: 10.1016/j.envres.2017.09.013 28963966

[38] Bodewein L, Schmiedchen K, Dechent D, Stunder D, Graefrath D, Winter L, et al. Systematic review on the biological effects of electric, magnetic and electromagnetic fields in the intermediate frequency range (300 Hz to 1 MHz). Environ Res. 2019;171:247–59. doi: 10.1016/j.envres.2019.01.015 30690271

[39] Romeo S, Zeni O, Sannino A, Lagorio S, Biffoni M, Scarfì MR. Genotoxicity of radiofrequency electromagnetic fields: Protocol for a systematic review of in vitro studies. Environ Int. 2021;148:106386. doi: 10.1016/j.envint.2021.106386 33486297

[40] Mevissen M, Ward JM, Kopp-Schneider A, McNamee JP, Wood AW, Rivero TM, et al. Effects of radiofrequency electromagnetic fields (RF EMF) on cancer in laboratory animal studies. Environ Int. 2022;161:107106. doi: 10.1016/j.envint.2022.107106 35091376

[41] Pacchierotti F, Ardoino L, Benassi B, Consales C, Cordelli E, Eleuteri P, et al. Effects of Radiofrequency Electromagnetic Field (RF-EMF) exposure on male fertility and pregnancy and birth outcomes: Protocols for a systematic review of experimental studies in non-human mammals and in human sperm exposed in vitro. Environ Int. 2021;157:106806. doi: 10.1016/j.envint.2021.106806 34454359 PMC8484860

[42] Pophof B, Burns J, Danker-Hopfe H, Dorn H, Egblomassé-Roidl C, Eggert T, et al. The effect of exposure to radiofrequency electromagnetic fields on cognitive performance in human experimental studies: A protocol for a systematic review. Environ Int. 2021;157:106783. doi: 10.1016/j.envint.2021.106783 34333292 PMC8485020

[43] Röösli M, Dongus S, Jalilian H, Feychting M, Eyers J, Esu E, et al. The effects of radiofrequency electromagnetic fields exposure on tinnitus, migraine and non-specific symptoms in the general and working population: A protocol for a systematic review on human observational studies. Environ Int. 2021;157:106852. doi: 10.1016/j.envint.2021.106852 34500362 PMC8484767

[44] Covidence - Better systematic review management. [cited 16 Apr 2026]. https://www.covidence.org/

[45] Mendeley. [cited 16 Apr 2026]. https://www.mendeley.com/

[46] Smart Search - Web of Science Core Collection. [cited 16 Apr 2026]. https://www.webofscience.com/wos/woscc/smart-search

[47] Simkó M, Mattsson M-O. 5G Wireless Communication and Health Effects-A Pragmatic Review Based on Available Studies Regarding 6 to 100 GHz. Int J Environ Res Public Health. 2019;16(18):3406. doi: 10.3390/ijerph16183406 31540320 PMC6765906

[48] Ding H, Hu GL, Zheng XY, Chen Q, Threapleton DE, Zhou ZH. The method quality of cross-over studies involved in Cochrane Systematic Reviews. PLoS One. 2015;10(4):e0120519. doi: 10.1371/journal.pone.0120519 25867772 PMC4395015

[49] Ijima E, Kodera S, Hirata A, Hikage T, Matsumoto A, Ishitake T, et al. Excessive whole-body exposure to 28 GHz quasi-millimeter wave induces thermoregulation accompanied by a change in skin blood flow proportion in rats. Front Public Health. 2023;11:1225896. doi: 10.3389/fpubh.2023.1225896 37732093 PMC10507335

[50] Jamal L, Yahia-Cherif L, Hugueville L, Mazet P, Lévêque P, Selmaoui B. Assessment of Electrical Brain Activity of Healthy Volunteers Exposed to 3.5 GHz of 5G Signals within Environmental Levels: A Controlled-Randomised Study. Int J Environ Res Public Health. 2023;20(18):6793. doi: 10.3390/ijerph20186793 37754652 PMC10530694

[51] Ijima E, Li K, Hikage T, Nagai A, Murakami Y, Arima T, et al. Intensity-dependent temperature rise induced by local exposure to 26.5 GHz quasi-millimeter-wave in rat. In Vivo. 2023;37(5):2092–9. doi: 10.21873/invivo.13306 37652500 PMC10500540

[52] Wang H, Liu Y, Sun Y, Zhao L, Dong J, Xu X, et al. Changes in rat spatial learning and memory as well as serum exosome proteins after simultaneous exposure to 1.5 GHz and 4.3 GHz microwaves. Ecotoxicol Environ Saf. 2022;243: 113983. doi: 10.1016/j.ecoenv.2022.113983 35985199

[53] Sharma A, Bhatnagar D, Sisodia R. 10 Ghz microwaves induced biochemical, learning and memory alterations in swiss albino mice brain. IJAR. 2011;4(5):1–5. doi: 10.15373/2249555x/may2014/215

[54] D’Andrea JA, Thomas A, Hatcher DJ. Rhesus monkey behavior during exposure to high-peak-power 5.62-GHz microwave pulses. Bioelectromagnetics. 1994;15(2):163–76. doi: 10.1002/bem.2250150207 8024607

[55] Raslear TG, Akyel Y, Bates F, Belt M, Lu ST. Temporal bisection in rats: The effects of high-peak-power pulsed microwave irradiation. Bioelectromagnetics. 1993;14(5):459–78. doi: 10.1002/bem.2250140507 8285916

[56] Zhu R, Wang H, Xu X, Zhao L, Zhang J, Dong J, et al. Effects of 1.5 and 4.3 GHz microwave radiation on cognitive function and hippocampal tissue structure in Wistar rats. Sci Rep. 2021;11:10061. doi: 10.1038/s41598-021-89348-4 PMC8115682 33980913

[57] Rui G, Liu L-Y, Guo L, Xue Y-Z, Lai P-P, Gao P, et al. Effects of 5.8 GHz microwave on hippocampal synaptic plasticity of rats. Int J Environ Health Res. 2022;32(10):2247–59. doi: 10.1080/09603123.2021.1952165 34293966

[58] Wang H, Liu Y, Sun Y, Dong J, Xu X, Zhao X, et al. Changes in cognitive function, synaptic structure and protein expression after long-term exposure to 2.856 and 9.375 GHz microwaves. Cell Commun Signal. 2023;21:34. doi: 10.1186/s12964-022-01011-1 PMC9926547 36782203

[59] de Seze R, Poutriquet C, Gamez C, Maillot-Maréchal E, Robidel F, Lecomte A, et al. Repeated exposure to nanosecond high power pulsed microwaves increases cancer incidence in rat. PLoS One. 2020;15(4):e0226858. doi: 10.1371/journal.pone.0226858 32267859 PMC7141660

[60] Bektas H, Algul S, Altindag F, Yegin K, Akdag MZ, Dasdag S. Effects of 3.5 GHz radiofrequency radiation on ghrelin, nesfatin-1, and irisin level in diabetic and healthy brains. J Chem Neuroanat. 2022;126:102168. doi: 10.1016/j.jchemneu.2022.102168 36220504

[61] Qin T, Liu L, Wang X, Guo L, Lin J, Du J, et al. Combined effects of EMP and RF field on emotional behavior in mice. Front Public Health. 2023;11:1087161. doi: 10.3389/fpubh.2023.1087161 37006533 PMC10061096

[62] de Lorge JO, Ezell CS. Observing-responses of rats exposed to 1.28- and 5.62-GHz microwaves. Bioelectromagnetics. 1980;1(2):183–98. doi: 10.1002/bem.2250010208 7284023

[63] Kössler F, Lange F, Kupfer J, Rothe R. Effect of low-intensity microwave radiation on body weight, spontaneous activity and blood values in mice in a long-term experiment. Z Gesamte Hyg. 1981;27(1):44–7. 7269675

[64] Quock RM, Klauenberg BJ, Hurt WD, Merritt JH. Influence of microwave exposure on chlordiazepoxide effects in the mouse staircase test. Pharmacol Biochem Behav. 1994;47(4):845–9. doi: 10.1016/0091-3057(94)90286-0 8029254

[65] Huai C, Gengdong Y, Shuiyun Z. Effects of microwave exposure at various power densities on mitochondrial marker enzymes in mouse brains. Journal of Bioelectricity. 1984;3(3):361–6. doi: 10.3109/15368378409027701

[66] Mickley GA, Cobb BL, Farrell ST. Brain hyperthermia alters local cerebral glucose utilization: A comparison of hyperthermic agents. Int J Hyperthermia. 1997;13(1):99–114. doi: 10.3109/02656739709056434 9024931

[67] Paulraj R, Behari J. Biochemical changes in rat brain exposed to low intensity 9.9 GHz microwave radiation. Cell Biochem Biophys. 2012;63(1):97–102. doi: 10.1007/s12013-012-9344-3 22426826

[68] Şahi̇ngöz Yildirim AG, Karaca E, Gözen O, Durmaz B, Yildiz T, Yildirim N, et al. The effect of radiofrequency waves on pregnant mice in association with genes involved in neuronal migration. J Clin Obstet Gynecol. 2022;32(4):111–9. doi: 10.5336/jcog.2021-84287

[69] Olazarán J, González B, Osa-Ruiz E, Felipe-Ruiz S, Boyano I, Fontani V, et al. Motor effects of radio electric asymmetric conveyer in Alzheimer’s disease: Results from a cross-over trial. J Alzheimers Dis. 2014;42(1):325–32. doi: 10.3233/JAD-140417 24898637

[70] Paulraj R, Behari J. Single strand DNA breaks in rat brain cells exposed to microwave radiation. Mutat Res. 2006;596(1–2):76–80. doi: 10.1016/j.mrfmmm.2005.12.006 16458332

[71] Fesenko EE, Makar VR, Novoselova EG, Sadovnikov VB. Microwaves and cellular immunity. I. Effect of whole body microwave irradiation on tumor necrosis factor production in mouse cells. Bioelectrochem Bioenerg. 1999;49(1):29–35. doi: 10.1016/s0302-4598(99)00058-6 10619445

[72] Novoselova EG, Fesenko EE, Makar VR, Sadovnikov VB. Microwaves and cellular immunity. Bioelectrochemistry and Bioenergetics. 1999;49(1):37–41. doi: 10.1016/s0302-4598(99)00059-8 10619446

[73] Novoselova EG, Glushkova OV, Khrenov MO, Novoselova TV, Lunin SM, Fesenko EE. Extremely low-level microwaves attenuate immune imbalance induced by inhalation exposure to low-level toluene in mice. Int J Radiat Biol. 2017;93(5):535–43. doi: 10.1080/09553002.2017.1270473 28067111

[74] Novoselova EG, Ogay VB, Sorokina OV, Glushkova OV, Sinotova OA, Fesenko EE. The Production of Tumor Necrosis Factor in Cells of Tumor-Bearing Mice after Total-Body Microwave Irradiation and Antioxidant Diet. Electromagn Biol Med. 2004;23:167–80. doi: 10.1081/LEBM-200042320

[75] Liddle CG, Putnam JP, Ail JS, Lewis JY, Bell B, West MW, et al. Alteration of circulating antibody response of mice exposed to 9-GHz pulsed microwaves. Bioelectromagnetics. 1980;1(4):397–404. doi: 10.1002/bem.2250010406 7284036

[76] Liddle CG, Putnam JP, Lewter OH, West M, Morrow G. Circulating antibody response of mice exposed to 9-GHz pulsed microwave radiation. Bioelectromagnetics. 1986;7(1):91–4. doi: 10.1002/bem.2250070110 3730005

[77] Shao BJ, Chiang H. The effects of microwaves on the immune system in mice. Journal of Bioelectricity. 1989;8:1–10.

[78] Veyret B, Bouthet C, Deschaux P, de Seze R, Geffard M, Joussot-Dubien J, et al. Antibody responses of mice exposed to low-power microwaves under combined, pulse-and-amplitude modulation. Bioelectromagnetics. 1991;12(1):47–56. doi: 10.1002/bem.2250120107 2012621

[79] Rifat F, Sharma A, Srivastava P, Patni S, Sisodia R. Protective effects of prunus avium extract on 10 Ghz induced damages in the spleen of swiss albino mice. IJAR. 2011;3(8):680–4. doi: 10.15373/2249555x/aug2013/219

[80] Frei MR, Jauchem JR, Heinmets F. Thermoregulatory responses of rats exposed to 9.3-GHz radiofrequency radiation. Radiat Environ Biophys. 1989;28(1):67–77. doi: 10.1007/BF01209724 2919227

[81] Jauchem JR, Frei MR, Heinmets F. Heart rate changes due to 5.6-GHz radiofrequency radiation: Relation to average power density. Proc Soc Exp Biol Med. 1984;177(3):383–7. doi: 10.3181/00379727-177-41960 6514713

[82] Blick DW, Adair ER, Hurt WD, Sherry CJ, Walters TJ, Merritt JH. Thresholds of microwave-evoked warmth sensations in human skin. Bioelectromagnetics. 1997;18(6):403–9. doi: 10.1002/(sici)1521-186x(1997)18:6<403::aid-bem1>3.0.co;2-6 9261536

[83] Akdag MZ, Sert C, Kaya H, Dasdag S, Celik MS. Effects of whole-body chronic microwave exposure on some hormones of variously treated rats. Biochem Arch. 1999;15:345–50.

[84] Hirsch SE, Appleton B, Fine BS, Brown PV. Effects of repeated microwave irradiations to the albino rabbit eye. Invest Ophthalmol Vis Sci. 1977;16(4):315–9. 844990

[85] Kumar S, Kesari KK, Behari J. Influence of microwave exposure on fertility of male rats. Fertil Steril. 2011;95(4):1500–2. doi: 10.1016/j.fertnstert.2010.04.078 20723534

[86] Kumar S, Behari J, Sisodia R. Impact of microwave at X-band in the aetiology of male infertility. Electromagn Biol Med. 2012;31(3):223–32. doi: 10.3109/15368378.2012.700293 22897403

[87] Kumar S, Behari J, Sisodia R. Influence of electromagnetic fields on reproductive system of male rats. Int J Radiat Biol. 2013;89(3):147–54. doi: 10.3109/09553002.2013.741282 23078358

[88] Xue Y, Guo L, Lin J, Lai P, Rui G, Liu L. Effects of 5.8 GHz microwaves on testicular structure and function in rats. Biomed Res Int. 2022;2022:301–14. doi: 10.1155/2022/5182172 PMC9192205 35707372

[89] Jensh RP. Studies of the teratogenic potential of exposure of rats to 6000 MHz microwave radiation. II. Postnatal psychophysiologic evaluations. Radiat Res. 1984;97:282–301. 6695050

[90] Pyrpasopoulou A, Kotoula V, Cheva A, Hytiroglou P, Nikolakaki E, Magras IN, et al. Bone morphogenetic protein expression in newborn rat kidneys after prenatal exposure to radiofrequency radiation. Bioelectromagnetics. 2004;25(3):216–27. doi: 10.1002/bem.10185 15042631

[91] Adang D, Remacle C, Vorst AV. Results of a long-term low-level microwave exposure of rats. IEEE Trans Microwave Theory Techn. 2009;57(10):2488–97. doi: 10.1109/tmtt.2009.2029667

[92] van Rongen E, van Rhoon GC, Aleman A, Kelfkens G, Kromhout H, van Leeuwen FE, et al. Comments on "results of a long-term low-level microwave exposure of rats. IEEE Trans Microwave Theory Techn. 2011;59(7):1893–4. doi: 10.1109/tmtt.2011.2131678

[93] Sisodia R, Rifat F, Sharma A, Srivastava P, Sharma K. Effects of 10-GHz microwaves on hematological parameters in Swiss albino mice and their modulation by Prunus avium. J Environ Pathol Toxicol Oncol. 2013;32(3):205–17. doi: 10.1615/jenvironpatholtoxicoloncol.2013007891 24266407

[94] Jensh RP. Studies of the teratogenic potential of exposure of rats to 6000 MHz microwave radiation. I. Morphologic analysis at term. Radiat Res. 1984;97:272–81. 6695049

[95] Knyazeva IR, Medvedev MA, Kutenkov OP, Vasilev AV, Gorokhovsky AA, Rostov VV. The Features of the Stress-Reaction to Repetitive Nanosecond Microwave Pulses. In: 2018 20th International Symposium on High-Current Electronics (ISHCE), 2018.94–7. 10.1109/ishce.2018.8521190

[96] Kumar S, Kesari KK, Behari J. Evaluation of genotoxic effects in male Wistar rats following microwave exposure. Indian J Exp Biol. 2010;48(6):586–92. 20882761

[97] Verma S, Keshri GK, Karmakar S, Mani KV, Chauhan S, Yadav A. Effects of microwave 10 GHz radiation exposure in the skin of rats: An insight on molecular responses. Radiat Res. 2021;196:404–16. doi: 10.1667/RADE-20-00155.1 34407201

[98] Bektas H, Dasdag S, Nalbant A, Akdag MB, Demir C, Kavak S. 3.5 GHz radiofrequency radiation may affect biomechanics of bone and muscle of diabetics. Biotechnology & Biotechnological Equipment. 2023;37(1):329–38. doi: 10.1080/13102818.2023.2199096

[99] Schrot J, Hawkins TD. Lethal effects of 3000 MHz radiation on the rat. Radiat Res. 1974;59(2):504–12. doi: 10.2307/3573999 4417617

[100] Perov SY, Rubtsova NB, Belaya OV. Status of the neuroendocrine system in animals chronically exposed to electromagnetic fields of 5G mobile network base stations. Bull Exp Biol Med. 2022;174(2):277–9. doi: 10.1007/s10517-023-05689-2 36598666

[101] Bayatli E, Tetik B, Büyüktepe M, Tokdemir N, Güner YE, Güney Y. Epidemiological Markers in Adult Glial Tumors: A Single-Center Experience and The Utility of the Classification System. Turk Onkoloji Dergisi. 2022;37:105–14. doi: 10.5505/TJO.2021.3441

[102] Zhang Y, Zhang Y, Ye Z, Yang S, Liu M, Wu Q, et al. Mobile phone use, genetic susceptibility and new-onset chronic kidney diseases. Int J Public Health. 2023;68:1605358. doi: 10.3389/ijph.2023.1605358 36874223 PMC9977800

[103] Kostoff RN, Heroux P, Aschner M, Tsatsakis A. Adverse health effects of 5G mobile networking technology under real-life conditions. Toxicol Lett. 2020;323:35–40. doi: 10.1016/j.toxlet.2020.01.020 31991167

[104] Butković I, Vince S, Lojkić M, Folnožić I, Tur SM, Vilić M, et al. Effects of 5G radiofrequency electromagnetic radiation on indicators of vitality and DNA integrity of in vitro exposed boar semen. Theriogenology. 2024;230:243–9. doi: 10.1016/j.theriogenology.2024.09.025 39342826

[105] Jamal L, Michelant L, Delanaud S, Hugueville L, Mazet P, Lévêque P, et al. Autonomous nervous system responses to environmental-level exposure to 5G’s first deployed band (3.5 GHz) in healthy human volunteers. Exp Physiol. 2024;109:2122–33. doi: 10.1113/EP092083 PMC11607616 39404491

[106] Yuasa A, Uehara S, Ushizawa K, Kodera S, Arai N, Hirata A, et al. The thermal sensation threshold and its reliability induced by the exposure to 28 GHz millimeter-wave. Front Neurosci. 2024;18:1331416. doi: 10.3389/fnins.2024.1331416 38476868 PMC10927852

[107] Zhou GQ, Wang X, Gao P, Qin TZ, Guo L, Zhang ZW, et al. Intestinal microbiota via NLRP3 inflammasome dependent neuronal pyroptosis mediates anxiety-like behaviour in mice exposed to 3.5 GHz radiofrequency radiation. Science of The Total Environment. 2024;927:172391. doi: 10.1016/J.SCITOTENV.2024.172391 38608899

[108] Kojima M, Tasaki T, Kamijo T, Hada A, Suzuki Y, Kik A, et al. Investigation of the Ocular Response and Corneal Damage Threshold of Exposure to 28 GHz Quasi-millimeter Wave Exposure. Health Phys. 2025;128(6):487–96. doi: 10.1097/HP.0000000000001951 39787318

[109] Ijima E, Nagai A, Li K, Hikage T, Kamizawa N, Hidaka E. Front Public Health. 2025;13:1580155. doi: 10.3389/fpubh.2025.1580155 PMC12206703 40589806

[110] Michelant L, Baz T, Carrie A, Hugueville L, Lévêque P, Selmaoui B. Millimeter-wave high frequency 5G (26 GHz) electromagnetic fields do not modulate human brain electrical activity. Environ Res. 2026;289:123349. doi: 10.1016/J.ENVRES.2025.123349 41265672

[111] Michelant L, Hugueville L, Lévêque P, Selmaoui B. No measurable impact of acute 26 GHz 5G exposure on salivary stress markers in healthy adults. Environ Res. 2026;290:123439. doi: 10.1016/J.ENVRES.2025.123439 41317834

[112] Matsumoto A, Endo I, Ijima E, Hirata A, Kodera S, Ichiba M, et al. Single exposure to near-threshold 5G millimeter wave modifies restraint stress responses in rats. Environ Health Prev Med. 2025;30:33. doi: 10.1265/ehpm.24-00321 40335291 PMC12062831

[113] Syed Taha SMA, Jaffar FHF, Hairulazam A, Vijay S, Jamaludin N, Zulkifli AF, et al. Prolonged 3.5 GHz and 24 GHz RF-EMF exposure alters testicular immune balance, apoptotic gene expression, and sperm function in rats. Biomedicines. 2025;13:2471. doi: 10.3390/BIOMEDICINES13102471/S1 PMC12562081 41153756

[114] Samoylova AV, Gostyukhina AA, Bolshakov MA, Yartsev VV, Evseeva SS, Doroshenko OS, et al. Combined effects of bone marrow cells and pulsed microwaves on thermally damaged skin of laboratory rats. Bull Exp Biol Med. 2024;178(1):91–5. doi: 10.1007/s10517-024-06288-5 39585592

[115] Wang X, Zhao X, Xu J, Li M, Sun B, Gao A. The impact of 9.375 GHz microwave radiation on the emotional and cognitive abilities of mice. Int J Mol Sci. 2025;26:2871. doi: 10.3390/IJMS26072871 PMC11988873 40243492

[116] Sun L, Wang X, Ren K, Yao C, Wang H, Xu X. Compound exposure of 2.8 GHz and 9.3 GHz microwave causes learning and memory impairment in rats. Heliyon. 2025;11:e41626. doi: 10.1016/J.HELIYON.2025.E41626 PMC11760294 39866417

[117] Bektas H, Yildirim S, Cakir S, Dogu S, Altindag F. Ameliorative Role of Coenzyme Q10 in RF Radiation-Associated Testicular and Oxidative Impairments in a 3.5-GHz Exposure Model. Bioelectromagnetics. 2026;47(1):e70043. doi: 10.1002/bem.70043 41578890

[118] Gelenli Dolanbay E, Mert T, Caliskan Bender G, Bektas H, Uslu U, Fernandez-Rodriguez CE. Male Reproductive and Cellular Damage After Prenatal 3.5 GHz Radiation Exposure: One-Year Postnatal Effects. Ann N Y Acad Sci. 2025;1554:140–52. doi: 10.1111/nyas.70116 41128732

[119] Geleni Dolanbay E, Mert T, Kurtoglu RN, Uslu U, Dasdag S. Prenatal Exposure to 3.5 GHz Radiofrequency Radiation and Long-Term Skin Histomorphometry: An 18-Month Experimental Rat Study. Medeni Med J. 2025;40(4):209–16. doi: 10.4274/MMJ.galenos.2025.48323 41479147 PMC12758509

[120] Liu S, Cheng Y, Zhang X, Shen Y, Wu S, Chen Y, et al. Assessing effects of microwave electromagnetic field in APP/PS1 mice by in vivo GABA-weighted imaging via variable delay multipulse-chemical exchange saturation transfer MRI. Neurochem Int. 2026;194:106131. doi: 10.1016/j.neuint.2026.106131 41713500

[121] Li Y, Yao B, Men J, Pang Y, Gao J, Bai Y, et al. Oxidative stress and energy metabolism in male reproductive damage from single and combined high-power microwave exposure at 1.5 and 4.3GHz. Reprod Toxicol. 2025;132:108759. doi: 10.1016/j.reprotox.2024.108759 39617305

[122] Obajuluwa AO, Lech JC, Amina D, Onwuka CC, Bolarinwa RA, Obajuluwa TM, et al. Single and combined neurotoxic, cytotoxic and genotoxic effects of 5GHz MIMO waves and computed tomography irradiation in male Wistar rats. Egyptian Journal of Basic and Applied Sciences. 2023;10(1):835–45. doi: 10.1080/2314808x.2023.2282243

[123] Emre M, Karamazi Y, Emre T, Varan NE, Toyran T, Yücebilgiç G. Effect of high-frequency radiofrequency (6 GHz) electromagnetic radiation on oxidative stress and kidney morphology. Toxicol Ind Health. 2026;42:124–36. doi: 10.1177/07482337261422845 41701901

[124] Karamazı Y, Emre M, Uçar S, Aksoy G, Emre T, Tokuş M. Effect of 6 GHz radiofrequency electromagnetic field on the development of fetal bones. Electromagn Biol Med. 2025;44(1):17–25. doi: 10.1080/15368378.2024.2438608 39692219

[125] Sousouri G, Eicher C, D’Angelo RM, Billecocq M, Fussinger T, Studler M, et al. 5G radio-frequency-electromagnetic-field effects on the human sleep electroencephalogram: A randomized controlled study in CACNA1C genotyped volunteers. Neuroimage. 2025;317:121340. doi: 10.1016/j.neuroimage.2025.121340 40541756

[126] Torkan A, Zoghi M, Foroughimehr N, Jaberzadeh S. The effect of 5G mobile phone electromagnetic exposure on corticospinal and intracortical excitability in healthy adults: A randomized controlled pilot study. Brain Sci. 2025;15(11):1134. doi: 10.3390/brainsci15111134 41300142 PMC12649992

[127] Nikitina VN, Kalinina NI, Dubrovskaya EN, Plekhanov VP, Kovshov AA. Experimental study of animal behavior under the influence of the electromagnetic field of the 5G mobile communication standard. Biol Bull Russ Acad Sci. 2024;51(11):3473–80. doi: 10.1134/s1062359024701875

[128] Wang X, Zhou G, Lin J, Zhang Z, Qin T, Guo L. Effects of 4.9 GHz radiofrequency field exposure on brain metabolomic and proteomic characterization in mice. Biology. 2024;13:806. doi: 10.3390/BIOLOGY13100806/S1 PMC11505847 39452115

[129] Seewooruttun C, Bouguila B, Corona A, Delanaud S, Bodin R, Bach V. 5G radiofrequency exposure reduces PRDM16 and C/EBP β mRNA Expression, Two Key Biomarkers for Brown Adipogenesis. Int J Mol Sci. 2025;26:2792. doi: 10.3390/IJMS26062792/S1 PMC11942954 40141434

[130] Lameth J, Royer J, Martin A, Marie C, Arnaud-Cormos D, Lévêque P, et al. Repeated Head Exposures to a 5G-3.5 GHz Signal Do Not Alter Behavior but Modify Intracortical Gene Expression in Adult Male Mice. Int J Mol Sci. 2025;26(6):2459. doi: 10.3390/ijms26062459 40141104 PMC11941837

[131] Zhou G, Guo L, Qin T, Zhang Z, Gao P, Wang X, et al. Single and combined microwave induced anxiety-like behavior via NLRP3 dependent neuronal pyroptosis in hippocampus and amygdala in mice. Ecotoxicol Environ Saf. 2026;310:119818. doi: 10.1016/j.ecoenv.2026.119818 41643305

[132] Yilmaz H, Tümkaya L, Mercantepe T, Yılmaz A, Gül F, Suzan ZT. Effects of 5G Mobile Phone Network Electromagnetic Field Exposure on Testicular Endoplasmic Reticulum Stress and the Protective Role of Coenzyme Q10. Arch Med Res. 2025;56(4):103157. doi: 10.1016/j.arcmed.2024.103157 39765017

[133] Zhaowen Z, Ling G, Guiqiang Z, Jiajin L, Tongzhou Q, Jiangyi L, et al. Effects of paternal 5G RFR exposure on health of male offspring mice. Reprod Toxicol. 2026;140:109139. doi: 10.1016/j.reprotox.2025.109139 41391574

[134] Bodin R, Seewooruttun C, Corona A, Delanaud S, Pelletier A, Villégier A-S. Sex-dependent impact of perinatal 5G electromagnetic field exposure in the adolescent rat behavior. Environ Sci Pollut Res Int. 2023;30(53):113704–17. doi: 10.1007/s11356-023-30256-7 37851267

[135] Vijayalaxmi. Biological and health effects of radiofrequency fields: Good study design and quality publications. Mutat Res Genet Toxicol Environ Mutagen. 2016;810:6–12. doi: 10.1016/j.mrgentox.2016.09.007 27776692

[136] Kuster N, Schönborn F. Recommended minimal requirements and development guidelines for exposure setups of bio-experiments addressing the health risk concern of wireless communications. Bioelectromagnetics. 2000;21(7):508–14. doi: 10.1002/1521-186x(200010)21:7<508::aid-bem4>3.0.co;2-f 11015115

[137] Valberg PA. Designing EMF experiments: What is required to characterize “exposure”?. Bioelectromagnetics. 1995;16(6):396-401 discussion 402-6. doi: 10.1002/bem.2250160608 8789071

[138] Makinistian L, Muehsam DJ, Bersani F, Belyaev I. Some recommendations for experimental work in magnetobiology, revisited. Bioelectromagnetics. 2018;39(7):556–64. doi: 10.1002/bem.22144 30303544

[139] ETAIN project. [cited 16 Apr 2026]. https://www.etainproject.eu/

[140] GOLIAT. [cited 16 Apr 2026]. https://projectgoliat.eu/

[141] NextGEM. [cited 16 Apr 2026]. https://www.nextgem.eu/

[142] Seawave project. [cited 16 Apr 2026]. https://seawave-project.eu/

[143] European Research Cluster on EMF and Health (CLUE-H). [cited 16 Apr 2026]. https://www.emf-health-cluster.eu/

