# Supplementary material for: Physiological and health-related effects of 5G-relevant radiofrequency fields: A systematic review of experimental studies in humans and mammals
Source: PLoS One. 2026 Nov 4;21(11):e0361104. doi: 10.1371/journal.pone.0361104 (PMC13734471; doi:10.1371/journal.pone.0361104)
Supplement: Supplementary file 1 [file pone.0361104.s001.pdf]

S1 Search Strings. Links to search strings for review search in EMF-Portal and PubMed.

1. EMF-Portal

The database was searched using the following search string:

TA=5G AND (TA=review OR TA=meta-analysis)

Topics: all

Frequency ranges: all

Link: https://www.emf-portal.org/de/article/search/v2/results?query=ta%3D5G+AND+%28ta%3Dreview+OR+ta%3Dmeta-analysis%29

2. PubMed

The database was searched using the following search string:

(5G[Title/Abstract]) AND ((review[Title/Abstract]) OR (meta-analysis[Title/Abstract]))

Link: https://pubmed.ncbi.nlm.nih.gov/?term=%285G%5BTitle%2FAbstract%5D%29+AND+%28%28review%5BTitle%2FAbstract%5D%29+OR+%28meta-analysis%5BTitle%2FAbstract%5D%29%29
