# Supplementary material for: Physiological and health-related effects of 5G-relevant radiofrequency fields: A systematic review of experimental studies in humans and mammals
Source: PLoS One. 2026 Nov 4;21(11):e0361104. doi: 10.1371/journal.pone.0361104 (PMC13734471; doi:10.1371/journal.pone.0361104)
Supplement: Supplementary file 2 [file pone.0361104.s002.pdf]

S2 Extraction Table. Extraction table of previous reviews about 5G exposure-relevant effects.

Abbreviations: EEG = electroencephalography, FR1/FR2 = frequency range 1/2, RF EMF = radiofrequency electromagnetic field

Reference	Type of publication	Databases searched in and search dates	Design/type and number of studies included in systematic review	Exposure (frequency range across all included studies)	Outcome considered	Methodology provided (systematic search, PECO, data extraction, study appraisal, evidence synthesis, meta-analysis)	Conclusion regarding 5G frequencies	Limitations
ANSES (2022)<br><br>*English summary of a more detailed report in French*	Opinion report, published by ANSES<br><br>review	Different data sources (depending on frequency range; Scopus, Pubmed and some key publications): 2012–2020	Not elucidated in detail in the English summary (animal and *in vitro* studies are at least mentioned)	700 MHz–2100 MHz, 900 MHz–2.5 GHz, 3–4 GHz, 18–100 GHz	Health effects, biological effects	Comprehensive search	3.5 GHz: only 5 studies identified; based on data of exposures from 900 MHz–2.5 GHz exposure to 3.5 GHz does not constitute a new health risk<br><br>26 GHz:<br>no conclusion possible	Search strings and inclusion/exclusion criteria not provided, data extraction and study appraisal not shown (*at least not in the English summary*)
Chiaraviglio et al. (2021)	Peer-reviewed journal article<br><br>Review based on communica-tions engineering perspective (focus not only on health)	No data provided	Not elucidated in detail<br><br>Cancer: some animal studies and epidemiological studies	RF EMF	Cancer, skin effects, ocular effects, glucose metabolism, male fertility, electrosensitivity, COVID-19	Not elucidated in detail, narrative synthesis	Health concerns attributed to 5G are not supported by scientific evidence from communications engineering	Only cancer studies evaluated in detail

Di Ciaula (2018)	Peer-reviewed journal article<br><br>Narrative review	Pubmed: 2011–2018	Epidemiological studies, experimental animal studies, *in vitro* studies<br><br>Numbers not exactly provided	Millimeter waves used in 5G Communica-tion systems (not exactly specified)	Cancer, reproductive effects, metabolic effects, neurologic effects, microbiological effects	Systematic search, narrative synthesis	No clear conclusion based on the included studies provided (Authors: "available findings seem sufficient to demonstrate the existence of biomedical effects")	Search strings not provided, PECO not provided, data extraction not in detail, no study appraisal, no evidence synthesis
European Parliament: Directorate-General for Parliamentary Research Services (2021)	Study report, published by European Parliament<br><br>Review (effects on inverte-brates and plants and fungi), meta-review (effects on vertebrates; <6 GHz)	Web of Science: > 1945	*In vivo* studies *in vitro* studies, meta-analysis, reviews, dosimetry studies, studies on dielectric heating<br><br>Numbers provided according to frequency range and taxonomy group: n= 23–109	450 MHz–6 GHz, 6 GHz–300 GHz	Effects on vertebrates (cells) (except humans), invertebrates and plants/fungi<br><br>Any effects, e.g. reproductive effects, morphogenesis, carcinogenicity, hyperthermia, dielectric heating, development, population diversity, behavioural effects, magnetic sense, neural effects, genotoxicity, gene/protein expression, cardiovascular effects, auditory effects, cerebral effects, physiological effects	Systematic search, data extraction, narrative synthesis	No effect, apart from dielectric heating, in all six categories (3 taxonomy groups, 2 frequency ranges)	Search strings not provided, no study appraisal, no evidence synthesis
European Parliament: Directorate-General for Parliamentary Research Services (2021)	Study report, published by European Parliament<br><br>narrative review,	Pubmed: from inception to 2020<br><br>EMF-Portal: from inception to 2021	<u>Cancer</u>: 20/1 epidemiological studies (FR1/FR2); 7/0 experimental animal studies (FR1/FR2)	2G–5G frequencies (FR1: 450 MHZ–6 GHz and FR2: 24–100 GHz)	Carcinogenic effects, reproductive and developmental effects	Systematic search, PECO, data extraction, evidence synthesis<br><br>FR1: narrative review	<u>Cancer</u>:<br><u>FR1</u>: EMF are probably carcinogenic for humans; <u>FR2</u>: no adequate studies available<br><br><u>Reproductive and developmental effects</u>:	Study appraisal not based on specific transparent tool (only presented in short narrative manner or as comment), search String for EMF-Portal not provided;

	scoping review		<u>Reproductive and developmental effects</u>: 26/2 epidemiological studies (FR1/FR2); 39/0 experimental animal studies (FR1/FR2)			FR2: scoping review	<u>FR1</u>: clear effects on male and possibly on female fertility; possible adverse effects on development of embryos and newborns; <u>FR2</u>: no adequate studies available	not clear if report was peer-reviewed
Health Council of the Netherlands (2020)	Background document and advisory report, published by Health Council of the Netherlands<br><br>Literature survey	Pubmed, EMF-Portal OR literature search based on other document (e.g. draft WHO review and reports of Swedish Radiation Safety Authority), depending on frequency range and endpoint: 2015–2020, from inception to 2020 or not exactly provided	Epidemiological studies, experimental animal studies, experimental human studies, *in vitro* studies<br><br>Numbers provided according to frequency range, study types and endpoint (0–64)	700 MHz–2200 MHz, 2200 MHz–5 GHz, 20 GHz–40 GHz	<u>Diseases and conditions</u>: studies on cancer, symptoms, hearing, eyes, cardiovascular system, autonomic nervous system, neurodegenerative diseases, male fertility, pregnancy and birth defects<br><br><u>Biological processes</u>: studies on behaviour, cognition, sleep, signal conduction in the brain, electrical activity in the brain, blood-brain barrier, gene expression in the brain, immune system, blood, hormones, oxidative stress	Systematic search for certain frequency ranges and endpoints, quality criteria for inclusion of papers, customized quality assessment but not provided in detail	<u>Diseases and conditions</u>: occurrence of cancer, reduced male fertility, poorer pregnancy outcomes and congenital defects classified as “possible” (but neither proven nor probable)<br><br><u>Biological processes:</u> relations are classified as “possible” for these processes<br><br>Committee is unable to make any statement about the actual health risks of future exposures to 5G frequencies	Systematic search not performed for all frequency ranges and endpoints, data extraction and risk of bias not provided in detail, no evidence synthesis
Hinrikus et al. (2022)	Peer-reviewed journal article	EMF-Portal: 2007–2015	73 experimental human studies	Search for 0.01 GHz–300 GHz, evaluation:	Effects on brain (EEG, cognition, behavior, brain metabolism)	Systematic search, narrative synthesis	No data for 5G; no consistent relationship between effects and parameters of exposure by different generations (2G,	Data extraction not shown in detail; no study appraisal (some quality criteria

	Review			450 MHz–2500 MHz			3G, and 4G); impact of 5G FR1 should have no principal differences compared to the previous generations	seemed to be used for exclusion only); studies analyzed as function of frequency and signal structure and not according to outcome
Karipidis et al., (2021)	Peer-reviewed journal article<br><br>"State-of-the science review"	Pubmed, EMF-Portal, Google Scholar, Embase and WoS, major reviews and reference lists of included studies: from inception to 2019	107 experimental (1 human, 15 animals, 91 *in vitro*) studies<br><br>31 epidemiological studies	> 6 GHz–300 GHz (below occupational limit values)	Various effects including genotoxicity, cell proliferation, gene expression, cell signaling, membrane function and other effects<br><br>Epidemiological studies: cancer, effects on reproduction and diseases	Comprehensive search, data extraction, narrative synthesis	No confirmed evidence that low-level RF EMF above 6 GHz are hazardous to human health	Search strings not provided, no PECO, study appraisal not based on specific transparent tool (only presented in short narrative manner or as comment), no transparent evidence synthesis
Russell (2018)	Peer-reviewed journal article<br><br>Review	No details provided	No details provided	Relevant electro-magnetic frequencies of 2G, 3G, 4G and 5G including millimeter wave exposure	Health effects and biological effects (electrosensitivity, oxidative stress, skin effects, ocular effects, immune system, bacterial antibiotic resistance, gene expression)	No details provided, narrative synthesis	RF EMF appears to act as a toxin to biological systems; a moratorium on the deployment of 5G is warranted	No details on methodology
Simkó and Mattsson (2019)	Peer-reviewed journal article<br><br>"pragmatic review"	Pubmed, EMF-Portal, other relevant grey literature: from inception to 2018	*In vivo* experimental studies, *in vitro* studies<br><br>altogether 94 studies	6 GHz–100 GHz	Effects grouped into physiological, neurological, histological changes, gene or protein expression, cytotoxic effects, genotoxic changes, temperature-related reactions	Data extraction, study appraisal based on customized pre-defined criteria	No consistent relationship between power density, exposure duration, or frequency, and exposure effects; no adequate and sufficient information for a meaningful safety assessment, or for the	No systematic search (or at least not transparently provided), search strings not provided, PECO not provided, study appraisal based on customized pre-

							question about non-thermal effects	defined criteria, studies analyzed as function of frequency and not systematically according to outcome; no transparent evidence synthesis
Sofri et al. (2022)	Peer-reviewed journal article<br><br>Systematic review	Not provided	33 experimental human studies	RF exposure related to base station and mobile phones	Cognitive performance, well-being, physiological parameters (heart rate, blood pressure, body temperature), EEG	Comprehensive search, data extraction, narrative synthesis	No studies identified with 5G exposures (700 MHz, 3.5 GHz, 28 GHz)	Search strings not provided, no PECO, no study appraisal, no evidence synthesis
Wood et al. (2021)	Peer-reviewed journal article<br><br>Meta-analysis (quantitative analysis of papers included in Karipidis et al. 2021, see above)	Pubmed, EMF-Portal, Google Scholar, Embase and Web of Science, major reviews and reference lists of included studies: from inception to 2019	107 experimental (1 human, 15 animals, 91 *in vitro*) studies<br><br>31 epidemiological studies	> 6 GHz–300 GHz (below occupational limit values (100 W/m²))	Papers were grouped into bacteria and yeast (24); (non-bacterial) cells in culture (35); artificial cell suspensions (7); neural activity (10); *in vivo* (8) and miscellaneous (4)	Comprehensive search, meta-analysis, narrative synthesis	No association between low-level microwaves and biological effects	Search strings not provided, no clear PECO, study appraisal based on customized pre-defined criteria, no transparent evidence synthesis; studies analyzed as function of frequency, quality score, power density and SAR and not according to outcome
