# Supplementary material for: Physiological and health-related effects of 5G-relevant radiofrequency fields: A systematic review of experimental studies in humans and mammals
Source: PLoS One. 2026 Nov 4;21(11):e0361104. doi: 10.1371/journal.pone.0361104 (PMC13734471; doi:10.1371/journal.pone.0361104)
Supplement: Supplementary file 3 [file pone.0361104.s003.pdf]

S3 Search Strings. Links to search strings in EMF-Portal, PubMed, and Web of Science.

1. EMF-Portal

The database was searched using the following criteria:

Topics: experimental studies, epidemiological studies, medical applications

Frequency ranges: radiofrequency (≥ 10 MHz), mobile communications

Time span: complete time span

Link: https://www.emf-portal.org/en/article/search/v2/results?query=%28topic%3Dexperimental+OR+topic%3Depidemiological+OR+topic%3Dmedical_application%29+AND+%28frequencyRange%3Dradio_frequency+OR+frequencyRange%3Dmobile_communications%29

2. PubMed

Terms were identified for each of the three components of the query Exposure, Species and Restrictions and were combined to interrogate the database:

Exposure	(("radiofrequenc*"[tiab] OR "radio-frequenc*"[tiab] OR "radio frequenc*"[tiab] OR "radiowave*"[tiab] OR "radio-wave*"[tiab] OR "microwave*"[tiab] OR "GHz"[tiab] OR "MHz"[tiab] OR "radar"[tiab] OR "super high frequency"[tiab] OR "centimeter wave*"[tiab] OR "centimetre wave*"[tiab] OR "millimeter wave*"[tiab] OR "millimetre wave*"[tiab] OR "5G"[tiab] OR "5th generation"[tiab]) AND ("field"[tiab] OR "expos*"[tiab] OR "radiat*"[tiab] OR "irradiat*"[tiab]))
Species	AND ("mammal"[tiab] OR "mice"[tiab] OR "mouse"[tiab] OR "rat"[tiab] OR "rats"[tiab] OR "hamster*"[tiab] OR "gerbil*"[tiab] OR "rabbit*"[tiab] OR "dog"[tiab] OR "dogs"[tiab] OR "monkey*"[tiab] OR "animal*"[tiab] OR "murin*"[tiab] OR "muridae"[tiab] OR "rodent*"[tiab] OR "cat"[tiab] OR "cats"[tiab] OR "sheep"[tiab] OR "goat*"[tiab] OR "cow"[tiab] OR "cows"[tiab] OR "wistar"[tiab] OR "balb"[tiab] OR "C57"[tiab] OR "C57bl"[tiab] OR "long-evans"[tiab] OR "primate*"[tiab] OR "cattle"[tiab] OR "bovine"[tiab] OR "pig"[tiab] OR "pigs"[tiab] OR "swine"[tiab] OR "Sprague-Dawley"[tiab] OR "human"[tiab] OR "humans"[tiab] OR "child*"[tiab] OR "adolescent*"[tiab] OR "man"[tiab] OR "men"[tiab] OR "woman"[tiab] OR "women"[tiab] OR "teen*"[tiab] OR "adult*"[tiab] OR "elderly"[tiab] OR "individual*"[tiab] OR "patient*"[tiab] OR "participant*"[tiab] OR "student*"[tiab] OR "subject*"[tiab] OR "volunteer*"[tiab])
Restrictions	NOT ("ablation"[tiab] OR "chemotherap*"[tiab] OR "app"[tiab] OR "apps"[tiab] OR "mobile application*"[tiab] OR "telemedicine"[tiab] OR "adherence"[tiab] OR "nomophobia"[tiab] OR "cyberbull*"[tiab] OR "problematic smartphone use"[tiab] OR "Genitourinary Syndrome of Menopause"[tiab] OR "problematic cell phone use"[tiab] OR "problematic smart phone use"[tiab] OR "phubbing"[tiab] OR "addict*"[tiab] OR "covid"[tiab] OR nanopart*[tiab] OR review[pt] OR systematic review[pt])

Applied filter: Language only English and German

3. Web of Science

Terms were identified for each of the three components of the query Exposure, Species and Restrictions and were combined to interrogate the database:

Exposure	TS=((radiofrequenc* OR radio-frequenc* OR "radio frequenc*" OR radiowave* OR radio-wave* OR microwave* OR "GHz" OR "MHz" OR "radar" OR "super high frequency" OR "centimet* wave" OR "millimet* wave" OR "5G" OR "5th generation") NEAR/2 ("field" OR "expos*" OR "radiat*" OR "irradiat*"))
Species	AND TS=("mammal" OR "mice" OR "mouse" OR "rat" OR "rats" OR "hamster?" OR "gerbil?" OR "rabbit?" OR "dog?" OR "monkey?" OR "animal*" OR "murin*" OR "muridae" OR "rodent*" OR "cat?" OR "sheep" OR "goat?" OR "cow?" OR "wistar" OR "balb" OR "C57" OR "C57bl" OR "long-evans" OR "primate?" OR "cattle" OR "bovine" OR "pig?" OR "swine" OR "Sprague-Dawley" OR "human?" OR "child*" OR "adolescent?" OR "man" OR "men" OR "wom?n" OR "teen*" OR "adult*" OR "elderly" OR "individual?" OR "patient?" OR "participant?" OR "student?" OR "subject?" OR "volunteer?")
Restrictions	NOT TS=("ablation" OR "chemotherap*" OR "app?" OR "mobile application*" OR "telemedicine" OR "adherence" OR "nomophobia" OR "cyberbull*" OR "problematic smartphone use" OR "Genitourinary Syndrome of Menopause" OR "problematic cell phone use" OR "problematic smart phone use" OR "phubbing" OR "addict*" OR "covid" OR nanopart*)
