# Supplementary material for: Physiological and health-related effects of 5G-relevant radiofrequency fields: A systematic review of experimental studies in humans and mammals
Source: PLoS One. 2026 Nov 4;21(11):e0361104. doi: 10.1371/journal.pone.0361104 (PMC13734471; doi:10.1371/journal.pone.0361104)
Supplement: Supplementary file 4 [file pone.0361104.s004.pdf]

S4 Excluded Articles. Excluded articles with reasons for their exclusion after title/abstract screening.

**Reference**	**Exclusion reason**
Abad, M., et al. (2016). "Association between electromagnetic field exposure and abortion in pregnant women living in Tehran." International Journal of Reproductive Biomedicine 14(5): 347-354.	Frequency not relevant, electric current application, co-exposure or exposure condition unclear
Abbasi, M., et al. (2017). "Multiple sclerosis and environmental risk factors: a case-control study in Iran." Neurol Sci 38(11): 1941-1951.	Frequency not relevant, electric current application, co-exposure or exposure condition unclear
Abdollahi, M. B., et al. (2021). "Comparison of mice' sperm parameters exposed to some hazardous physical agents." Environmental Analysis, Health and Toxicology 36(3): e2021013-2021010.	Frequency not relevant, electric current application, co-exposure or exposure condition unclear
Abdulkadyrov, K. M., et al. (1992). "Use Of Laser-Radiation and Microwave Emission In Treatment Of Patients With Malignant Diseases Of Hematopoietic System." Eksperimentalnaya Onkologiya 14(4): 67-70.	Article not in English/German
Abramova, T. V., et al. (2007). "Responses of hypothalamic orexin-containing neurons to cyclophosphamide, EHF-irradiation of the skin, and their combination in rats." Pathophysiology 14(2): 79-85.	Frequency not relevant, electric current application, co-exposure or exposure condition unclear
Adang, D. and A. Vander Vorst (2006). “First results of a long-term epidemiological study on low-level microwave exposure of rats.” IEEE MTT-S International Microwave Symposium Digest, San Francisco, CA, USA. IEEE, Piscataway Township, 2006: 1742-1745	Type of study not relevant or study not EMF/health-related
Adang, D., C. Remacle and A. Vander Vorst (2005). Long-term and low-thermal biological effects of microwaves. WIT Transactions on Biomedicine and Health 9: 397-404.	Type of study not relevant or study not EMF/health-related
Afolabi, O. B., et al. (2019). "Exposure to a 2.5 ghz Non-ionizing Electromagnetic Field Alters Hematological Profiles, Biochemical Parameters, and Induces Oxidative Stress in Male Albino Rats." Biomedical and Environmental Sciences 32(11): 860-863.	Frequency not relevant, electric current application, co-exposure or exposure condition unclear

Ahamed, V. I., N. G. Karthick and P. K. Joseph (2008). "Effect of mobile phone radiation on heart rate variability." Computers in Biology and Medicine 38(6): 709-712.	Frequency not relevant, electric current application, co-exposure or exposure condition unclear
Ait-Aissa, S., et al. (2010). "In situ detection of gliosis and apoptosis in the brains of young rats exposed in utero to a Wi-Fi signal." Comptes Rendus Physique 11(9-10): 592-601.	Frequency not relevant, electric current application, co-exposure or exposure condition unclear
Akbari, H. A. And A. A. Gaeini (2021). "Moderate exercise training as an effective strategy to reduce the harmful effects of cell phone radiation on Wistar rat's semen quality." International Journal of Radiation Research 19(2): 317-323.	Frequency not relevant, electric current application, co-exposure or exposure condition unclear
Akbari, H. A., A. A. Gaeini and S. Choobineh (2020). "Moderate exercise training reduced the harmful effects of electromagnetic radiation emitted from a cell phone on hematological parameters in male Wistar rats." Sport Sciences for Health 16(2): 267-272.	Frequency not relevant, electric current application, co-exposure or exposure condition unclear
Akbas, M., et al. (2011). "Efficacy of pulsed radiofrequency treatment on the saphenous nerve in patients with chronic knee pain." Journal of Back and Musculoskeletal Rehabilitation 24(2): 77-82.	Frequency not relevant, electric current application, co-exposure or exposure condition unclear
Akkam, Y., et al. (2020). "Correlation of Blood Oxidative Stress Parameters to Indoor Radiofrequency Radiation: A Cross Sectional Study in Jordan." International Journal of Environmental Research and Public Health 17(13): E4673.	Frequency not relevant, electric current application, co-exposure or exposure condition unclear
Akkaya, R., et al. (2019). "Wi-Fi decreases melatonin protective effect and increases hippocampal neuronal damage in pentylenetetrazole induced model seizures in rats." Pathophysiology 26(3-4): 375-379.	Frequency not relevant, electric current application, co-exposure or exposure condition unclear
Aksu, R., et al. (2010). "The efficiency of pulsed radiofrequency application on L5 and l6 dorsal roots in rabbits developing neuropathic pain." Regional Anesthesia and Pain Medicine 35(1): 11-15.	Frequency not relevant, electric current application, co-exposure or exposure condition unclear
Alassiri, M., et al. (2020). "Exposure to cell phones reduces heart rate variability in both normal-weight and obese normotensive medical students." Explore 16(4): 264-270.	Frequency not relevant, electric current application, co-exposure or exposure condition unclear

Albert, E. N. (1979). "Reversibility of microwave-induced blood-brain-barrier permeability." Radio Science 14(6): 323-327.	Frequency not relevant, electric current application, co-exposure or exposure condition unclear
Albert, E. N. And J. M. Kerns (1981). "Reversible microwave effects on the blood-brain barrier." Brain Research 230(1-2): 153-164.	Frequency not relevant, electric current application, co-exposure or exposure condition unclear
Albert, E. N. And M. Desantis (1975). "Do microwaves alter nervous system structure?" Annals Of The New York Academy Of Sciences 247: 87-108.	Frequency not relevant, electric current application, co-exposure or exposure condition unclear
Alexiades-Armenakas, M., J. S. Dover and K. A. Arndt (2008). "Unipolar radiofrequency treatment to improve the appearance of cellulite." Journal of Cosmetic and Laser Therapy 10(3): 148-153.	Frequency not relevant, electric current application, co-exposure or exposure condition unclear
Al-Khlaiwi, T. And S. S. Habib (2021). "Association of Excessive Mobile Phone Usage With Sleep Quality and Fatigue Severity: an Epidemiologic Survey in Saudi Population." Khyber Medical University Journal 13(2): 60-65.	Frequency not relevant, electric current application, co-exposure or exposure condition unclear
Al-Khlaiwi, T. M., et al. (2020). "The association of smart mobile phone usage with cognitive function impairment in Saudi adult population." Pakistan Journal of Medical Sciences 36(7): 1628-1633.	Frequency not relevant, electric current application, co-exposure or exposure condition unclear
Al-Khlaiwi, T., et al. (2022). "Comparison of mobile phone usage and physical activity on glycemic status, body composition & lifestyle in male Saudi mobile phone users." Heliyon 8(9): e10646.	Frequency not relevant, electric current application, co-exposure or exposure condition unclear
Alvarez, L. X., et al. (2019). "Effect of Targeted Pulsed Electromagnetic Field Therapy on Canine Postoperative Hemilaminectomy: A Double-Blind, Randomized, Placebo-Controlled Clinical Trial." Journal of the American Animal Hospital Association 55(2): 83-91.	Frequency not relevant, electric current application, co-exposure or exposure condition unclear
Alvarez, N., et al. (2008). "The effects of radiofrequency on skin: Experimental study." Lasers in Surgery and Medicine 40(2): 76-82.	Frequency not relevant, electric current application, co-exposure or exposure condition unclear
Amiri, F., et al. (2022). "The association between self-reported mobile phone usage with blood pressure and heart rate: evidence from a cross-sectional study." BMC Public Health 22: 2031.	Type of study not relevant or study not EMF/health-related

Andrianome, S., et al. (2017). "An assessment of the autonomic nervous system in the electrohypersensitive population: a heart rate variability and skin conductance study." Journal of Applied Physiology 123(5): 1055-1062.	Frequency not relevant, electric current application, co-exposure or exposure condition unclear
Andrianome, S., L. Yahia-Cherif and B. Selmaoui (2019). "Effect of short exposure to radiofrequency electromagnetic fields on saliva biomarkers: a study on the electrohypersensitive individuals." International Journal of Radiation Biology 95(6): 788-792.	Frequency not relevant, electric current application, co-exposure or exposure condition unclear
Anghileri, L. J., E. Mayayo and J. L. Domingo (2006). "Iron-radiofrequency synergism in lymphomagenesis." Immunopharmacology and Immunotoxicology 28(1): 175-183.	Frequency not relevant, electric current application, co-exposure or exposure condition unclear
Anghileri, L. J., et al. (2005). "Radiofrequency-induced carcinogenesis: cellular calcium homeostasis changes as a triggering factor." International Journal Of Radiation Biology 81(3): 205-209.	Frequency not relevant, electric current application, co-exposure or exposure condition unclear
Anguera, M. D. And R. J. Gianini (2014). "Prevalence of fatigue reported by physiotherapists operating diathermy equipment for microwave." Revista Brasileira de Epidemiologia (Brazilian Journal of Epidemiology) 17(3): 577-581.	Frequency not relevant, electric current application, co-exposure or exposure condition unclear
Appleton, B. And G. C. Mccrossan (1972). "Microwave lens effects in humans." Archives of Ophthalmology 88(3): 259-262.	Frequency not relevant, electric current application, co-exposure or exposure condition unclear
Appleton, B., et al. (1975). "Microwave lens effects in humans. II. Results of five-year survey." Archives of Ophthalmology 93(4): 257-258.	Frequency not relevant, electric current application, co-exposure or exposure condition unclear
Asci, H., et al. (2023). "Combined Pulsed Magnetic Field and Radiofrequency Electromagnetic Field Enhances MMP-9, Collagen-4, VEGF Synthesis to Improve Wound Healing Via Hif-1α/enos Pathway." Aesthetic Plastic Surgery.	Frequency not relevant, electric current application, co-exposure or exposure condition unclear
Ashani, Y., F. H. Henry and G. N. Catravas (1980). "Combined effects of anticholinesterase drugs and low-level microwave radiation." Radiation Research 84(3): 496-503.	Frequency not relevant, electric current application, co-exposure or exposure condition unclear
Atzmon, I., et al. (2012). "Cancer risks in the Druze Isifya Village: Reasons and RF/MW antennas." Pathophysiology 19(1): 21-28.	Frequency not relevant, electric current application, co-exposure or exposure condition unclear

Aurell, E. And B. Tengroth (1973). "Lenticular and retinal changes secondary to microwave exposure." Acta Ophthalmologica 51(6): 764-771.	Frequency not relevant, electric current application, co-exposure or exposure condition unclear
Austin, G. M. And S. Horvath (1949). "Production of convulsions in rats by exposure to ultra high frequency electrical currents (radar)." Am J Med Sci 218(1): 115.	Type of study not relevant or study not EMF/health-related
Aweda, M. A., S. Gbenebitse and R. O. Meindinyo (2010). "Microwave radiation exposures affect the ldl, hdl, tcl and trg status in rats." International Journal Of The Physical Sciences 5(7): 1015-1022.	Frequency not relevant, electric current application, co-exposure or exposure condition unclear
Ay, S. And D. Evcik (2009). "The effects of pulsed electromagnetic fields in the treatment of knee osteoarthritis: a randomized, placebo-controlled trial." Rheumatology International 29(6): 663-666.	Frequency not relevant, electric current application, co-exposure or exposure condition unclear
Aydin, F., et al. (2021). "The Effects of Heart-to-Mobile Phone Distance on the Circulatory System." Konuralp Tip Dergisi 13(1): 130-134.	Frequency not relevant, electric current application, co-exposure or exposure condition unclear
Azmy, R., et al. (2020). "Effects of mobile phones electromagnetic radiation on patients with epilepsy: an EEG study." The Egyptian Journal of Neurology, Psychiatry and Neurosurgery 56: 36.	Frequency not relevant, electric current application, co-exposure or exposure condition unclear
Bach, S. A., M. Baldwin and S. Lewis (1959). Some effects of ultrahigh frequency energy on primate cerebral activity, Rome Air Development Center (RADC): 82-92.	Frequency not relevant, electric current application, co-exposure or exposure condition unclear
Badzhinyan, S. A., et al. (2001). "Lethal effect of electromagnetic radiation of the millimeter wavelength range on cell cultures of chicken embryo." Doklady Biochemistry and Biophysics 377: 94-95.	Non-mammal/in vitro study
Bagatskaya, Y. V. And Y. V. Gura (2013). "Hypoalgesia Induced by Preliminary Microwave Irradiation of an Acupuncture Point: Effects on Somatic Pain in Mice." Neurophysiology 45(5-6): 453-458.	Frequency not relevant, electric current application, co-exposure or exposure condition unclear
Bagdasarova, I. V., A. V. Rudenko and E. N. Tumanyants (2001). "Experimental clinical study of the effect of millimeter waves on microbial and inflammatory renal diseases." Critical Reviews in Biomedical Engineering 29(5-6): 635-643.	Frequency not relevant, electric current application, co-exposure or exposure condition unclear

Bagnato, G. L., et al. (2016). "Pulsed electromagnetic fields in knee osteoarthritis: a double blind, placebo-controlled, randomized clinical trial." Rheumatology 55(4): 755-762.	Frequency not relevant, electric current application, co-exposure or exposure condition unclear
Baillie, H. D. (1970). Thermal and non-thermal cataractogenesis by microwaves, U.S. Department of Health, Education, and Welfare (HEW), Public Health Service (PHS), Bureau of Radiological Health (BRH): 59-65.	Frequency not relevant, electric current application, co-exposure or exposure condition unclear
Bakacak, M., et al. (2015). "The effects of electromagnetic fields on the number of ovarian primordial follicles: An experimental study." The Kaohsiung Journal of Medical Sciences 31(6): 287-292.	Frequency not relevant, electric current application, co-exposure or exposure condition unclear
Baker, R. J. And V. Smith (1977). "Experimental Arrangement for Invivo X-Ray And Microwave Irradiation Of Small Animals." Physics In Medicine and Biology 22(1): 147-148.	Type of study not relevant or study not EMF/health-related
Baldi, I., et al. (2011). "Occupational and residential exposure to electromagnetic fields and risk of brain tumors in adults: a case-control study in Gironde, France." International Journal of Cancer 129(6): 1477-1484.	Frequency not relevant, electric current application, co-exposure or exposure condition unclear
Baldwin, M., S. A. Bach and S. A. Lewis (1960). "Effects of radio-frequency energy on primate cerebral activity." Neurology 10: 178-187.	Frequency not relevant, electric current application, co-exposure or exposure condition unclear
Balode, Z. (1996). "Assessment of radio-frequency electromagnetic radiation by the micronucleus test in bovine peripheral erythrocytes." Science of the Total Evironment 180(1): 81-85.	Frequency not relevant, electric current application, co-exposure or exposure condition unclear
Band, P. R., et al. (2004). "Identification of Occupational Cancer Risks in British Columbia: A Population-Based Case-Control Study of 769 Cases of Non-Hodgkin's Lymphoma Analyzed by Histopathology Subtypes." Journal of Occupational and Environmental Medicine 46(5): 479-489.	Frequency not relevant, electric current application, co-exposure or exposure condition unclear
Band, P. R., et al. (2005). "Identification of occupational cancer risks in British Columbia: a population-based case-control study of 1129 cases of bladder cancer." Journal of Occupational and Environmental Medicine 47(8): 854-858.	Frequency not relevant, electric current application, co-exposure or exposure condition unclear

Banerjee, K., et al. (2022). "Effect of Reproductive and Lifestyle Factors on Anti-Mullerian Hormone Levels in Women of Indian Origin." Journal of Human Reproductive Sciences 15(3): 259-271.	Frequency not relevant, electric current application, co-exposure or exposure condition unclear
Banerjee, R., A. Goldfeder and J. Mitra (1983). "Sister chromatid exchanges and chromosome aberrations induced by radiosensitizing agents in bone marrow cells of treated tumor-bearing mice." J Natl Cancer Inst 70(3): 517-521.	Frequency not relevant, electric current application, co-exposure or exposure condition unclear
Bansal, D., et al. (2022). "Effect of Duration of Mobile Phone Use on the Salivary Flow and Total Antioxidant Capacity of Saliva and Salivary Immunoglobulin A Level: A Cross-sectional Study." Journal of International Society of Preventive & Community Dentistry 12(2): 260-265.	Frequency not relevant, electric current application, co-exposure or exposure condition unclear
Bansal, N., A. S. Dhaliwal and K. S. Mann (2015). Determination of Loss Tangent of Human Tear Film at 9.8 GHz. *AIP Conf. Proc.* 1675, 030081	Type of study not relevant or study not EMF/health-related
Baranski, S. (1971). "Effect of microwaves on the reaction of the leukocytic system." Acta Physiologica Polonica 22(6): 898.	Article not in English/German
Barbas-Monjo, M., et al. (2023). "Presence of Differences in the Radiofrequency Parameters Applied to Complex Pressure Ulcers: A Secondary Analysis." Medicina (Kaunas) 59(3), 516.	Frequency not relevant, electric current application, co-exposure or exposure condition unclear
Barbora, A. And R. Minnes (2021). "Targeted antiviral treatment using non-ionizing radiation therapy for SARS-cov-2 and viral pandemics preparedness: Technique, methods and practical notes for clinical application." Plos One 16(5): e0251780.	Non-mammal/in vitro study
Barron, C. I. And A. A. Baraff (1958). "Medical considerations of exposure to microwaves (radar)." Journal of the American Medical Association 168(9): 1194-1199.	Frequency not relevant, electric current application, co-exposure or exposure condition unclear
Barron, C. I., A. A. Love and A. A. Baraff (1955). "Physical evaluation of personnel exposed to microwave emanations." Journal of Aviation Medicine 26(6): 442-452.	Frequency not relevant, electric current application, co-exposure or exposure condition unclear
Barthélémy A., A. Mouchard, AS. Villégier (2016). “Glial markers and emotional memory in rats following cerebral radiofrequency exposures.” 2016 IEEE Radio and Antenna Days of the Indian Ocean (RADIO), Reunion, France. IEEE, 2016: 1-2	Frequency not relevant, electric current application, co-exposure or exposure condition unclear

Barthélémy, A., et al. (2016). "Glial markers and emotional memory in rats following acute cerebral radiofrequency exposures." Environmental Science and Pollution Research 23(24): 25343-25355.	Frequency not relevant, electric current application, co-exposure or exposure condition unclear
Baste, V., et al. (2012). "Pregnancy outcomes after paternal radiofrequency field exposure aboard fast patrol boats." Journal of Occupational and Environmental Medicine 54(4): 431-438.	Frequency not relevant, electric current application, co-exposure or exposure condition unclear
Baste, V., T. Riise and B. E. Moen (2008). "Radiofrequency electromagnetic fields; male infertility and sex ratio of offspring." Eur J Epidemiol 23, 369–377	Frequency not relevant, electric current application, co-exposure or exposure condition unclear
Bastuji-Garin, S., S. Richardson and R. Zittoun (1990). "Acute leukaemia in workers exposed to electromagnetic fields." European Journal of Cancer & Clinical Oncology 26(11-12): 1119-1120.	Frequency not relevant, electric current application, co-exposure or exposure condition unclear
Baumgardt-Elms, C., et al. (2002). "Testicular cancer and electromagnetic fields (EMF) in the workplace: results of a population-based case-control study in Germany." Cancer Causes and Control 13(10): 895-902.	Frequency not relevant, electric current application, co-exposure or exposure condition unclear
Baus, R. And J. D. Fleming (1959). Biological effects of microwave radiation with limited body heating, Rome Air Development Center (RADC): 291-313.	Article not peer-reviewed
Bayatli, E., et al. (2022). "Epidemiological Markers in Adult Glial Tumors: A Single-Center Experience and The Utility of the Classification System." Turk Onkoloji Dergisi (Turkish Journal of Oncology) 37(2): 105-114.	Frequency not relevant, electric current application, co-exposure or exposure condition unclear
Behrens, T., et al. (2010). "Occupational exposure to electromagnetic fields and sex-differential risk of uveal melanoma." Occupational and Environmental Medicine 67(11): 751-759.	Frequency not relevant, electric current application, co-exposure or exposure condition unclear
Bektas, H., M. S. Bektas and S. Dasdag (2022). "Effect of mobile phone usage duration during pregnancy on the general motor movements of infants." Biotechnology & Biotechnological Equipment 36(1): 56-66.	Frequency not relevant, electric current application, co-exposure or exposure condition unclear
Bektas, H., S. Dasdag and M. S. Bektas (2020). "Comparison of effects of 2.4 ghz Wi-Fi and mobile phone exposure on human placenta and cord blood." Biotechnology & Biotechnological Equipment 34(1): 154-162.	Frequency not relevant, electric current application, co-exposure or exposure condition unclear

Belyavskaya, A., et al. (2020). "Changes In Male Reproductive System Under Adverse Environmental Conditions." Archiv Euromedica 10(2): 14-17.	Frequency not relevant, electric current application, co-exposure or exposure condition unclear
Belyavskaya, A., et al. (2020). "Microwave Radiation and Male Reproductive Function." Archiv Euromedica 10(2): 18-19.	Frequency not relevant, electric current application, co-exposure or exposure condition unclear
Berg, G., et al. (2006). "Occupational exposure to radio frequency/microwave radiation and the risk of brain tumors: Interphone Study Group, Germany." American Journal of Epidemiology 164(6): 538-548.	Frequency not relevant, electric current application, co-exposure or exposure condition unclear
Berman, E., H. B. Carter and D. House (1981). "Observations of Rat Fetuses after Irradiation with 2450-MHz (CW) Microwaves." Journal Of Microwave Power and Electromagnetic Energy 16(1): 9-13.	Frequency not relevant, electric current application, co-exposure or exposure condition unclear
Bermant, R. I., et al. (1979). "Classical conditionig of microwave-induced hyperthermia in rats." Radio Science 14: 201-207.	Frequency not relevant, electric current application, co-exposure or exposure condition unclear
Bernat, R. (1985). "Glutathione concentration and peptidase activity in the lens after exposure to microwaves." Acta Physiologica Polonica 36(5-6): 360-365.	Frequency not relevant, electric current application, co-exposure or exposure condition unclear
Bertone, A., et al. (2006). "Effective bone hemostasis and healing using radiofrequency and conductive fluid." Clinical Orthopaedics and Related Research 446: 278-285.	Frequency not relevant, electric current application, co-exposure or exposure condition unclear
Bhangari, D. S., A. C. Bhagali and R. V. Kshirsagar (2018). “Brain Wave Frequency Variation by Electromagnetic Field & its Optimization Results.” 2018 Second International Conference on Intelligent Computing and Control Systems (ICICCS), 1679-1683	Frequency not relevant, electric current application, co-exposure or exposure condition unclear
Bini, M., et al. (1986). "Exposure of workers to intense RF electric fields that leak from plastic sealers." Journal Of Microwave Power and Electromagnetic Energy 21(1): 33-40.	Frequency not relevant, electric current application, co-exposure or exposure condition unclear
Birenbaum, L., et al. (1969). "Effect of microwaves on the eye." IEEE Transactions on Biomedical Engineering 16(1): 7-14.	Frequency not relevant, electric current application, co-exposure or exposure condition unclear
Birenbaum, L., et al. (1969). "Effects of microwaves on the rabbit eye." The Journal of Microwave Power 4(4): 232-243.	Frequency not relevant, electric current application, co-exposure or exposure condition unclear

Bisceglia, B., et al. (1986). "Cytogenetic Observations on Microwave Exposed Bovine Lymphocytes." Microwave Journal 29(5): 115-115.	Type of study not relevant or study not EMF/health-related
Bise, W. (1978). "Low power radio-frequency and microwave effects on human electroencephalogram and behavior." Physiological Chemistry and Physics 10(5): 387-398.	Frequency not relevant, electric current application, co-exposure or exposure condition unclear
Blackmore, S. J. And N. Rose (2002). "Testing the bioelectric shield." Alternative Therapies in Health and Medicine 8(5): 62-67.	Frequency not relevant, electric current application, co-exposure or exposure condition unclear
Blay, R. M., et al. (2020). "Influence of Lifestyle and Environmental Factors on Semen Quality in Ghanaian Men." International Journal of Reproductive Medicine 2020: 6908458.	Frequency not relevant, electric current application, co-exposure or exposure condition unclear
Blumen, M. B., et al. (2008). "Comparative study of four radiofrequency generators for the treatment of snoring." Otolaryngology - Head and Neck Surgery 138(3): 294-299.	Frequency not relevant, electric current application, co-exposure or exposure condition unclear
Bogers, R. P., et al. (2013). "Design of an ecological momentary assessment study of exposure to radiofrequency electromagnetic fields and non-specific physical symptoms." BMJ Open 3(8): e002933.	Type of study not relevant or study not EMF/health-related
Bogers, R. P., et al. (2018). "Individual variation in temporal relationships between exposure to radiofrequency electromagnetic fields and non-specific physical symptoms: A new approach in studying 'electrosensitivity'." Environment International 121: 297-307.	Frequency not relevant, electric current application, co-exposure or exposure condition unclear
Bolte, J. F. B., et al. (2019). "Ecological momentary assessment study of exposure to radiofrequency electromagnetic fields and non-specific physical symptoms with self-declared electrosensitives." Environment International 131: 104948.	Frequency not relevant, electric current application, co-exposure or exposure condition unclear
Bouji, M., et al. (2020). "Impact of Cerebral Radiofrequency Exposures on Oxidative Stress and Corticosterone in a Rat Model of Alzheimer's Disease." Journal of Alzheimer's Disease 73(2): 467-476.	Frequency not relevant, electric current application, co-exposure or exposure condition unclear

Bouthet, C., et al. (1988). "Bioelectromagnetism - Effects Of A Magnetic-Field With Or Without Superimposed Microwave Irradiation On Nk Cell-Activity In Balb/C Mice." Archives Internationales de Physiologie De Biochimie et de Biophysique 96(4): A417.	Type of study not relevant or study not EMF/health-related
Bragin, D. E., et al. (2015). "Increases in microvascular perfusion and tissue oxygenation via pulsed electromagnetic fields in the healthy rat brain." Journal of Neurosurgery 122(5): 1239-1247.	Frequency not relevant, electric current application, co-exposure or exposure condition unclear
Bragin, D. E., et al. (2018). "Pulsed Electromagnetic Field (PEMF) Mitigates High Intracranial Pressure (ICP) Induced Microvascular Shunting (MVS) in Rats." Acta Neurochirurgica. Supplement 126: 93-95.	Frequency not relevant, electric current application, co-exposure or exposure condition unclear
Braithwaite, L. A., et al. (1993). “Microwave Radiation as a Heat Source For Young Livestock.” Livestock environment IV : Fourth International Symposium, University of Warwick, Coventry, England, 6-9 July 1993. ASAE Publication, volume 93-3; American Society of Agricultural Engineers 1993: 609-616.	Frequency not relevant, electric current application, co-exposure or exposure condition unclear
Brasil, L. J., et al. (2020). "Effects of Pulsed Radiofrequency on a Standard Model of Muscle Injury in Rats." Anesth Pain Med 10(1): e97372.	Frequency not relevant, electric current application, co-exposure or exposure condition unclear
Braun, H. And G. Dhom (1956). "[Animal experiments with microwaves]." Strahlentherapie 99(4): 617-623.	Frequency not relevant, electric current application, co-exposure or exposure condition unclear
Braun, H. And G. Dhom (1956). "Tierexperimentelle Untersuchungen mit Mikrowellen." Strahlentherapie 99(4): 617-623.	Frequency not relevant, electric current application, co-exposure or exposure condition unclear
Bristow, I., et al. (2017). "Microwave therapy for cutaneous human papilloma virus infection." European Journal of Dermatology 27(5): 511-518.	Frequency not relevant, electric current application, co-exposure or exposure condition unclear
Brook, J., et al. (2012). "Pulsed radiofrequency electromagnetic field therapy: a potential novel treatment of plantar fasciitis." J Foot Ankle Surg 51(3): 312-316.	Frequency not relevant, electric current application, co-exposure or exposure condition unclear
Brown, P. V. K. And L. E. Larsen (1980). "Differing effects of pulsed and CW microwave energy upon nerve function as detected by birefringence measurements." IEEE Transactions on Microwave Theory and Techniques 28(10): 1126-1133.	Frequency not relevant, electric current application, co-exposure or exposure condition unclear

Bruce-Wolfe, V. And D. R. Justesen (1979). "Microwave-induced hyperthermia and the visually evoked electrocortical response of the guinea pig." Radio Science 14: 187-191.	Frequency not relevant, electric current application, co-exposure or exposure condition unclear
Brückner, V., F. Zywietz and H. Jung (1979). "Über den Einfluß von Mikrowellen-induzierter Lokalhyperthermie und Röntgenstrahlung auf das Walker-Karzinom der Ratte." Strahlentherapie 155(1): 44-50.	Frequency not relevant, electric current application, co-exposure or exposure condition unclear
Bryan, R. N. (1966). "Retrograde amnesia: effects of handling and microwave radiation." Science 153(3738): 897-899.	Frequency not relevant, electric current application, co-exposure or exposure condition unclear
Bunin, G. R., et al. (1990). "Neuroblastoma and parental occupation." American Journal of Epidemiology 131(5): 776-780.	Frequency not relevant, electric current application, co-exposure or exposure condition unclear
Burlaka, A., et al. (2014). "Changes in mitochondrial functioning with electromagnetic radiation of ultra high frequency as revealed by electron paramagnetic resonance methods." International Journal of Radiation Biology 90(5): 357-362.	Frequency not relevant, electric current application, co-exposure or exposure condition unclear
Byun, K. A., et al. (2023). "Radiofrequency Irradiation Attenuated UVB-Induced Skin Pigmentation by Modulating ATP Release and CD39 Expression." International Journal of Molecular Sciences 24(6): 5506.	Frequency not relevant, electric current application, co-exposure or exposure condition unclear
Cabré-Riera, A., et al. (2020). "Estimated whole-brain and lobe-specific radiofrequency electromagnetic fields doses and brain volumes in preadolescents." Environment International 142: 105808.	Frequency not relevant, electric current application, co-exposure or exposure condition unclear
Cabré-Riera, A., et al. (2021). "Association between estimated whole-brain radiofrequency electromagnetic fields dose and cognitive function in preadolescents and adolescents." International Journal of Hygiene and Environmental Health 231: 113659.	Frequency not relevant, electric current application, co-exposure or exposure condition unclear
Cabré-Riera, A., et al. (2022). "Estimated all-day and evening whole-brain radiofrequency electromagnetic fields doses, and sleep in preadolescents." Environmental Research 204: 112291.	Frequency not relevant, electric current application, co-exposure or exposure condition unclear

Cairnie, A. B. And R. K. Harding (1981). "Cytological studies in mouse testis irradiated with 2.45-GHz continuous-wave microwaves." Radiation Research 87(1): 100-108.	Frequency not relevant, electric current application, co-exposure or exposure condition unclear
Calis, P., et al. (2021). "Does Exposure of Smart Phones during Pregnancy Affect the Offspring's Ovarian Reserve? A Rat Model Study." Fetal and Pediatric Pathology 40(2): 142-152.	Frequency not relevant, electric current application, co-exposure or exposure condition unclear
Calvo, A. (2013). "Conventional radiofrequency treatment in five patients with trigeminal neuralgia." Medicina Oral, Patología Oral y Cirugía Bucal 18(1): e76-e80.	Frequency not relevant, electric current application, co-exposure or exposure condition unclear
Calvo, A., A. Jimenez and R. March (2013). "Radiofrequency treatment of cervicogenic headache." Medicina Oral, Patología Oral y Cirugía Bucal 18(2): e293-e297.	Type of study not relevant or study not EMF/health-related
Capareli, F., et al. (2023). "Low-energy amplitude-modulated electromagnetic field exposure: Feasibility study in patients with hepatocellular carcinoma." Cancer Medicine 12(11): 12402-12412.	Frequency not relevant, electric current application, co-exposure or exposure condition unclear
Carpenter, R. L. And C. A. Van Ummersen (1968). "The action of microwave radiation on the eye." The Journal of Microwave Power 3(1): 3-19.	Type of study not relevant or study not EMF/health-related
Carpenter, R. L., E. S. Ferri and G. J. Hagan (1974). Assessing microwaves as a hazard to the eye - Progress and problems, Biologic Effects and Health Hazards of Microwave Radiation: Proceedings of an International Symposium, Warsaw, 15-18 October, 1973. Polish Medical Publications, Warsaw, Poland, 1974; 178-184.	Frequency not relevant, electric current application, co-exposure or exposure condition unclear
Castagna, A., et al. (2011). "Radioelectric asymmetric brain stimulation and lingual apex repositioning in patients with atypical deglutition." Journal of Multidisciplinary Healthcare 4: 209-213.	No control group/condition
Castaño-Vinyals, G., et al. (2022). "Wireless phone use in childhood and adolescence and neuroepithelial brain tumours: Results from the international MOBI-Kids study." Environ Int 160: 107069.	Frequency not relevant, electric current application, co-exposure or exposure condition unclear
Castren, J., et al. (1982). "On the microwave exposure." Acta Ophthalmologica 60(4): 647-654.	Frequency not relevant, electric current application, co-exposure or exposure condition unclear

Catravas, G. N., et al. (1976). "Proceedings: Biochemical changes in the brain of rats exposed to microwaves of low power density." Journal of Microwave Power 11(2): 147-148.	Type of study not relevant or study not EMF/health-related
Chandel, P., et al. (2022). "Determination of short-interval time estimates in humans exposed to radiofrequency electromagnetic radiation." Journal of Environmental Biology 43(3): 369-376.	Frequency not relevant, electric current application, co-exposure or exposure condition unclear
Chang, D. C., et al. (1992). "Rapid Loading of Molecules into Animal Cells using a Radio-Frequency Electric Field." Biology Bulletin 183(2): 364-365.	Frequency not relevant, electric current application, co-exposure or exposure condition unclear
Chao, S. C., et al. (2008). "Percutaneous pulsed radiofrequency in the treatment of cervical and lumbar radicular pain." Surgical Neurology 70(1): 59-65.	Frequency not relevant, electric current application, co-exposure or exposure condition unclear
Chen, B., et al. (2017). "Complementary effects of negative-pressure wound therapy and pulsed radiofrequency energy on cutaneous wound healing in diabetic mice." Plastic & Reconstructive Surgery 139(1): 105-117.	Frequency not relevant, electric current application, co-exposure or exposure condition unclear
Chen, H. G., et al. (2022). "Association between electronic device usage and sperm quality parameters in healthy men screened as potential sperm donors." Environmental Pollution 312: 120089.	Frequency not relevant, electric current application, co-exposure or exposure condition unclear
Chernovetz, M. E., D. R. Justesen and A. F. Oke (1977). "A teratological study of the rat: Microwave and infrared radiations compared." Radio Science 12: 191-197.	Frequency not relevant, electric current application, co-exposure or exposure condition unclear
Chiang, H. And M. Wang (1984). "Morphological change in mouse sperm following microwave exposure." Journal of Bioelectricity 3(3): 367-372.	Frequency not relevant, electric current application, co-exposure or exposure condition unclear
Chiang, H., et al. (1989). "Health effects of environmental electromagnetic fields." Journal of Bioelectricity 8(1): 127-131.	Frequency not relevant, electric current application, co-exposure or exposure condition unclear
Choi, G. S., et al. (2011). "Short-term effects of pulsed radiofrequency on chronic refractory cervical radicular pain." Annals of Rehabilitation Medicine 35(6): 826-832.	Frequency not relevant, electric current application, co-exposure or exposure condition unclear
Choi, H. J., et al. (2012). "Clinical outcomes of pulsed radiofrequency neuromodulation for the treatment of occipital neuralgia." Journal of Korean Neurosurgical Society 51(5): 281-285.	Frequency not relevant, electric current application, co-exposure or exposure condition unclear

Choi, S. Y., et al. (2018). "Improvement in abdominal and flank contouring by a novel adipocyte-selective non-contact radiofrequency device." Lasers in Surgery & Medicine 50: 738-744	Frequency not relevant, electric current application, co-exposure or exposure condition unclear
Choi, S., et al. (2013). "Internal-specific morphological analysis of sciatic nerve fibers in a radiofrequency-induced animal neuropathic pain model." Plos One 8(9): e73913.	Frequency not relevant, electric current application, co-exposure or exposure condition unclear
Chongchitpaisan, W., et al. (2021). "Trigger of a migraine headache among Thai adolescents smartphone users: a time series study." Environmental Analysis, Health and Toxicology 36(1): e2021006-2021000.	Frequency not relevant, electric current application, co-exposure or exposure condition unclear
Chou, C. K. And A. W. Guy (1977). "Charakteristics of microwave-induced cochlear microphonics." Radio Science 12: 221-227.	Frequency not relevant, electric current application, co-exposure or exposure condition unclear
Chou, C. K. And R. Galambos (1979). "Middle-ear structures contribute little to auditory perception of microwaves." The Journal of Microwave Power 14(4): 321-326.	Frequency not relevant, electric current application, co-exposure or exposure condition unclear
Chou, C. K., A. W. Guy and R. Galambos (1976). "Microwave-induced cochlear microphonics in cats." The Journal of Microwave Power 11(2): 171-173.	Frequency not relevant, electric current application, co-exposure or exposure condition unclear
Chukanova, G. A. and G. G. Shurda (2003). “Influence of microwave radiation on body resistance level to causative agents of certain diseases.” 4th International Conference on Antenna Theory and Techniques (Cat. No.03EX699), Sevastopol, Ukraine. Band 2; IEEE, 2003: 807-808	Type of study not relevant or study not EMF/health-related
Chye, C. L., et al. (2015). "Pulsed radiofrequency treatment of articular branches of femoral and obturator nerves for chronic hip pain." Clinical Interventions in Aging 10: 569-574.	Frequency not relevant, electric current application, co-exposure or exposure condition unclear
Cipolletta, L., et al. (2005). "Delivery of radiofrequency energy to the gastroesophageal junction (Stretta procedure) for the treatment of gastroesophageal reflux disease." Surg Endosc 19(6): 849-853.	Frequency not relevant, electric current application, co-exposure or exposure condition unclear
Clearly, S. F. And B. S. Pasternack (1966). "Lenticular changes in microwave workers. A statistical study." Archives of Environmental Health 12(1): 23-29.	Frequency not relevant, electric current application, co-exposure or exposure condition unclear

Cleary, S. F., B. S. Pasternack and G. W. Beebe (1965). "Cataract incidence in radar workers." Archives of Environmental Health 11(2): 179-182.	Frequency not relevant, electric current application, co-exposure or exposure condition unclear
Cobb, B. L., et al. (2000). "Neural and behavioral teratological evaluation of rats exposed to ultra-wideband electromagnetic fields." Bioelectromagnetics 21(7): 524-537.	Frequency not relevant, electric current application, co-exposure or exposure condition unclear
Cobbs, C., et al. (2019). "An early feasibility study of the Nativis Voyager(®) device in patients with recurrent glioblastoma: first cohort in US." CNS Oncology 8(1): CNS30.	Frequency not relevant, electric current application, co-exposure or exposure condition unclear
Cohen, J., et al. (2019). "Mild hyperthermia as a localized radiosensitizer for deep-seated tumors: investigation in an orthotopic prostate cancer model in mice." The British Journal of Radiology 92(1095): 20180759.	Frequency not relevant, electric current application, co-exposure or exposure condition unclear
Çöl, N., Ö. Kömürcü-Karuserci and C. Demirel (2021). "The possible effects of maternal electronic media device usage during pregnancy on children's sleep patterns." Turkish Archives of Pediatrics 56(3): 254-260.	Frequency not relevant, electric current application, co-exposure or exposure condition unclear
Collodel, G., et al. (2012). "The effect of radio electric asymmetric conveyer treatment on sperm parameters of subfertile stallions: a pilot study." Reproductive Biology 12(3): 277-284.	Frequency not relevant, electric current application, co-exposure or exposure condition unclear
Cooper, T., et al. (1965). "Effect Of Pyridoxine and Pyridoxal on Circulatory Response of Rats to Microwave Irradiation." Experientia 21(1): 28-29.	Frequency not relevant, electric current application, co-exposure or exposure condition unclear
Costa, F. P., et al. (2011). "Treatment of advanced hepatocellular carcinoma with very low levels of amplitude-modulated electromagnetic fields." British Journal of Cancer 105(5): 640-648.	Frequency not relevant, electric current application, co-exposure or exposure condition unclear
Czerski, P. (1974). "Microwave Irradiation and the Circadian Rhythm of Bone Marrow Cell Mitosis." The Journal of Microwave Power 9(1): 31-37.	Frequency not relevant, electric current application, co-exposure or exposure condition unclear
Czerski, P., M. Siekierzynski and A. Gidynski (1974). "Health surveillance of personnel occupationally exposed to microwaves. I. Theoretical considerations and practical aspects." Aerospace Medicine 45: 1137-1142.	Frequency not relevant, electric current application, co-exposure or exposure condition unclear

Czyz, C. N., et al. (2012). "Efficacy of pulsed electromagnetic energy in postoperative recovery from blepharoplasty." Dermatologic Surgery 38(3): 445-450.	Frequency not relevant, electric current application, co-exposure or exposure condition unclear
Dabouis, V., et al. (2016). "First epidemiological study on occupational radar exposure in the French Navy: a 26-year cohort study." International Journal of Environmental Health Research 26(2): 131-144.	Frequency not relevant, electric current application, co-exposure or exposure condition unclear
Daels, J. (1973). "Microwave heating of the uterine wall during parturition." Obstetrics and Gynecology 42(1): 76-79.	Frequency not relevant, electric current application, co-exposure or exposure condition unclear
Daels, J. (1976). "Proceedings: Microwave heating of the uterine wall during parturition." The Journal of Microwave Power 11(2): 166-167.	Frequency not relevant, electric current application, co-exposure or exposure condition unclear
Daily Jr, L., et al. (1948). "Effects of microwave diathermy on the eye." American Journal of Physiology 155: 432.	Type of study not relevant or study not EMF/health-related
Daily Jr, L., et al. (1950). "The effects of microwave diathermy on the eye; an experimental study." American Journal of Ophthalmology 33(8): 1241-1254	Frequency not relevant, electric current application, co-exposure or exposure condition unclear
Daily Jr, L., et al. (1951). "Influence of microwaves on certain enzyme systems in the lens of the eye." American Journal of Ophthalmology 34(9): 1301-1306.	Frequency not relevant, electric current application, co-exposure or exposure condition unclear
D'Ancona, F. C., et al. (1999). "Results of high-energy transurethral microwave thermotherapy in patients categorized according to the American Society of Anesthesiologists operative risk classification." Urology 53(2): 322-328.	Frequency not relevant, electric current application, co-exposure or exposure condition unclear
Dandrea, J. A. (1995). Effects of Microwave Radiation Exposure on Behavioral Performance in nonhuman Primates. Radiofrequency Radiation Standards 274: 271-277.	Type of study not relevant or study not EMF/health-related
Daniels, W. M., et al. (2009). "The effect of electromagnetic radiation in the mobile phone range on the behaviour of the rat." Metabolic Brain Disease 24(4): 629-641.	Frequency not relevant, electric current application, co-exposure or exposure condition unclear
Danulescu, R., et al. (2011). "Nervous System and Neuroendocrine Effects in Long Term Occupational Exposure to Microwaves." Environmental Engineering and Management Journal 10(4): 481-489.	Frequency not relevant, electric current application, co-exposure or exposure condition unclear

Dasdag, S., et al. (1998). "Effects of microwaves on the phagocytic activity of variously treated rat macrophages." Electro- and Magnetobiology 17: 185-194.	Non-mammal/in vitro study
Dastamooz, S., S. T. Broujeni and N. Sarahian (2023). "A primary study on rat fetal development and brain-derived neurotrophic factor levels under the control of electromagnetic fields." Journal of Public Health in Africa 14(6): 2347.	Frequency not relevant, electric current application, co-exposure or exposure condition unclear
Davis, R. L. And F. K. Mostofi (1993). "Cluster of testicular cancer in police officers exposed to hand-held radar." American Journal of Industrial Medicine 24(2): 231-233.	Frequency not relevant, electric current application, co-exposure or exposure condition unclear
De Giudici, P., et al. (2021). "Radiofrequency exposure of people living near mobile-phone base stations in France." Environ Res 194: 110500.	Type of study not relevant or study not EMF/health-related
De la Rosette, J. J., et al. (1994). "Transurethral microwave thermotherapy (TUMT) in benign prostatic hyperplasia: placebo versus TUMT." Urology 44(1): 58-63.	Frequency not relevant, electric current application, co-exposure or exposure condition unclear
De Lorge, J. O. (1983). The thermal basis for disruption of operant behavior by microwaves in three animal species, Academic Press, Microwaves and Thermoregulation: 379-399.	Article not peer-reviewed
De Roos, A. J., et al. (2001). "Parental occupational exposures to electromagnetic fields and radiation and the incidence of neuroblastoma in offspring." Epidemiology 12(5): 508-517.	Frequency not relevant, electric current application, co-exposure or exposure condition unclear
Degrave, E., et al. (2005). "All-cause mortality among Belgian military radar operators: a 40-year controlled longitudinal study." European Journal of Epidemiology 20(8): 677-681.	Frequency not relevant, electric current application, co-exposure or exposure condition unclear
Degrave, E., et al. (2009). "Causes of death among Belgian professional military radar operators: a 37-year retrospective cohort study." International Journal of Cancer 124(4): 945-951.	Frequency not relevant, electric current application, co-exposure or exposure condition unclear
Deichmann, W. B., E. Bernal and M. Keplinger (1959). "Effects of environmental temperature and air volume exchange on survival of rats exposed to microwave radiation of 24,000 megacycles." Industrial Medicine & Surgery 28: 535-538.	RF EMF used to heat (> 42°C) and/or destroy tissue

Deichmann, W. B., et al. (1963). "Chronic Exposure of Dogs to Microwave Radiation of 24,000 Megacycles and a Power Density of 20 Mw/Cm2. Techn Note RADC-TDR-62-604." RADC TDR 86: 1-29.	Frequency not relevant, electric current application, co-exposure or exposure condition unclear
Deichmann, W. B., et al. (1963). "Effects on dogs of chronic exposure to microwave radiation." Journal of Occupational Medicine 5: 418-425.	No control group/condition
Demers, P. A., et al. (1991). "Occupational exposure to electromagnetic fields and breast cancer in men." American Journal of Epidemiology 134(4): 340-347.	Frequency not relevant, electric current application, co-exposure or exposure condition unclear
Desai, S. C., et al. (2016). "Minithyrotomy with radiofrequency-induced thermotherapy for the treatment of adductor spasmodic dysphonia." Laryngoscope 126(10): 2325-2329.	Frequency not relevant, electric current application, co-exposure or exposure condition unclear
Deschaux, P. And J. P. Pelissier (1977). "Effect of microwaves on steroids plasma levels in male rats." The Journal of Microwave Power 12(1): 46-47.	Frequency not relevant, electric current application, co-exposure or exposure condition unclear
Deschaux, P., et al. (1984). "Effect of Microwave Irradiation (2450 MHz) on Murine Cyto-Toxic Lymphocyte and Natural-Killer (Nk) Cells." Journal Of Microwave Power And Electromagnetic Energy 19(2): 107-110.	Frequency not relevant, electric current application, co-exposure or exposure condition unclear
Devyatkov, N. D., et al. (2001). "Effect of low-energy and high-peak-power nanosecond pulses of microwave radiation on malignant tumors." Critical Reviews in Biomedical Engineering 29(1): 98-110.	Type of study not relevant or study not EMF/health-related
Dewitt, J. R. And J. A. D'Andrea (1982). "Synergistic effects of microwaves and pentobarbital in laboratory rats." The Journal of Microwave Power 17(4): 282-283.	Type of study not relevant or study not EMF/health-related
Dias, J. F. (1965). "Eye Disease from Natural and Man-Made Radiation." Journal of the International College of Surgeons 43: 505-513.	Type of study not relevant or study not EMF/health-related
Dibaise, J. K., R. E. Brand and E. M. Quigley (2002). "Endoluminal delivery of radiofrequency energy to the gastroesophageal junction in uncomplicated GERD: efficacy and potential mechanism of action." Am J Gastroenterol 97(4): 833-842.	Frequency not relevant, electric current application, co-exposure or exposure condition unclear

Dietzel, F. (1974). "Tumor-Synchronisation durch Mikrowellen. Ein tierexperimenteller Beitrag zur Beeinflußbarkeit der Tumor-Proliferationskinetik in vivo durch nicht-ionisierende Strahlen." Strahlentherapie 148(5): 531-542.	Frequency not relevant, electric current application, co-exposure or exposure condition unclear
Dietzel, F., et al. (1975). "Microwaves in radiotherapy of tumors - alternative to heavy particles?" Strahlentherapie 149(4): 438-441.	Frequency not relevant, electric current application, co-exposure or exposure condition unclear
Dietzel, F., G. Linhart and G. Kovacs (1978). "Thermoradiotherapie im Tierexperiment: Tumorwachstum und Tumorheilungen." Strahlentherapie 154(12): 861-868.	Frequency not relevant, electric current application, co-exposure or exposure condition unclear
Djavan, B., et al. (1999). "High-energy transurethral microwave thermotherapy in patients with acute urinary retention due to benign prostatic hyperplasia." Urology 54(1): 18-22.	Frequency not relevant, electric current application, co-exposure or exposure condition unclear
Djirikian, D., et al. (1997). "Cerebral ischemic tolerance in rats elicited by microwave irradiation focused to the head." Faseb Journal 11(3): 3605-3605.	Type of study not relevant or study not EMF/health-related
Djordjevic, Z., et al. (1979). "A study of the health status of radar workers." Aviation, Space & Environmental Medicine 50(4): 396-398.	Frequency not relevant, electric current application, co-exposure or exposure condition unclear
Djordjevich, Z., N. Lazarevich and V. Djokovich (1977). "Studies on the hematologic effects of long-term, low-dose microwave exposure." Aviation, Space & Environmental Medicine: 516-518.	Frequency not relevant, electric current application, co-exposure or exposure condition unclear
Dokovic, V., Z. Dordevic and N. Lazarevic (1979). "Histomorphological Changes in the Testicles and Epididymis of Rats Exposed to High-Intensity Microwave-Radiation (70 Mw-Cm2)." Acta Veterinaria-Beograd 29(3-4): 121-128.	Frequency not relevant, electric current application, co-exposure or exposure condition unclear
Dokovic, V., Z. Dordevic and N. Lazarevic (1979). "Pathohistological Investigations of Effects of Different Intensity Microwave Radiation (10-50 Mw-Cm2) on the Reproductive-Organs of Male-Rats." Acta Veterinaria-Beograd 29(1-2): 55-62.	Frequency not relevant, electric current application, co-exposure or exposure condition unclear

Dömötör, Z., et al. (2022). "An idiographic approach to Idiopathic Environmental Intolerance attributed to Electromagnetic Fields (IEI-EMF) Part II. Ecological momentary assessment of three individuals with severe IEI-EMF." Heliyon 8(5): e09421.	Frequency not relevant, electric current application, co-exposure or exposure condition unclear
Dooley, E. S., J. Y. Gillenwater and E. D. Frohlich (1963). "Altered Renopressor Response-Pattern to Endotoxin Radiated with Radio-Frequency Energy. Rep 565." Report USAMRL: 1-21.	Type of study not relevant or study not EMF/health-related
Dragicevic, N., et al. (2011). "Long-term electromagnetic field treatment enhances brain mitochondrial function of both Alzheimer's transgenic mice and normal mice: a mechanism for electromagnetic field-induced cognitive benefit?" Neuroscience 185: 135-149.	Frequency not relevant, electric current application, co-exposure or exposure condition unclear
Drecun, M., et al. (1982). "EEG Response in Rats During Postnatal-Development After Repeated 2450 MHz Microwave-Radiation in Utero." Periodicum Biologorum 84(2): 128-131.	Frequency not relevant, electric current application, co-exposure or exposure condition unclear
Drecun, M., et al. (1983). "The Effects of Chronic Microwave Irradiation on the EEG of Adult Rats." Iugoslavica Physiologica et Pharmacologica Acta 19(1): 28-30.	Frequency not relevant, electric current application, co-exposure or exposure condition unclear
Drecun, M., et al. (1985). "EEG Changes in the Rat During Postnatal-Development after repeated 2450 MHz Microwave-Radiation in utero." Periodicum Biologorum 87(2): 220-223.	Frequency not relevant, electric current application, co-exposure or exposure condition unclear
Drecun, M., et al. (1989). "Effects of Low-Level Pulsed Microwave-Radiation on the EEG during Rat-Brain Development." Periodicum Biologorum 91(2): 225-230.	Frequency not relevant, electric current application, co-exposure or exposure condition unclear
Drobakhin et al. (2007). "Influence of microwave irradiation on the blood of men." 2007 6th International Conference on Antenna Theory and Techniques, Sevastopol, Ukraine. IEEE, 2007: 315-316	Frequency not relevant, electric current application, co-exposure or exposure condition unclear
Drobakhin, O. O., et al. (2005). Influence of microwave irradiation on the blood of men, IEEE: 315-316.	Frequency not relevant, electric current application, co-exposure or exposure condition unclear

Duk, I., M. Swietlikowski and S. Grabiec (1979). "The development of infestation in rats with Nippostrongylus brasiliensis (Travassos 1914) exposed to microwave action." Bull Acad Pol Sci Biol 27(3): 223-227.	Non-mammal/in vitro study
Dunaevskiy, G., et al. (2023). "Reduction of amputations of frostbitten limbs by treatment using microwave rewarming." Scientific Reports 13: 1362.	Frequency not relevant, electric current application, co-exposure or exposure condition unclear
Eakin, S. And W. D. Thompson (1962). "Effects of Microwave-Radiation on Activity Level of Rats." Psychological Reports 11(1): 192-192.	Frequency not relevant, electric current application, co-exposure or exposure condition unclear
Echchgadda, I., et al. (2022). "Changes in the excitability of primary hippocampal neurons following exposure to 3.0 GHz radiofrequency electromagnetic fields." Scientific Reports 12: 3506.	Non-mammal/in vitro study
Eeftens, M., et al. (2023). "The association between real-life markers of phone use and cognitive performance, health-related quality of life and sleep." Environmental Research 231: 116011.	Frequency not relevant, electric current application, co-exposure or exposure condition unclear
El-Domyati, M., et al. (2013). "Expression of p53 Protein After Nonablative Rejuvenation: The Other Side of the Coin." Dermatologic Surgery 39(6): 934-943.	Frequency not relevant, electric current application, co-exposure or exposure condition unclear
Electromagnetics, A., et al. (2009). Using Electromagnetic Microwave Field in Treatment of Lumbar Pain: 1741-1743.	Frequency not relevant, electric current application, co-exposure or exposure condition unclear
Elkhouly, A., M. F. A. Malek and S. Arza (2019). Effect of Wearing Protective Mask During Exposure to EMF on Body Temperature and Heart Rate, IEEE: 1-5.	Frequency not relevant, electric current application, co-exposure or exposure condition unclear
Elnaggar, R. K. (2020). "A Randomized, Controlled Trial on the Effectiveness of Photobiomodulation Therapy and Non-Contact Selective-Field Radiofrequency on Abdominal Adiposity in Adolescents with Obesity." Lasers Surg Med 52(9): 873-881.	Frequency not relevant, electric current application, co-exposure or exposure condition unclear
Elwood, J. M. And A. W. Wood (2019). "Animal studies of exposures to radiofrequency fields (vol 132, pg 98, 2019)." New Zealand Medical Journal 132(1507): 114-114.	Type of study not relevant or study not EMF/health-related

Ely, T. S., D. E. Goldman and J. Z. Hearon (1964). "Heating characteristics of laboratory animals exposed to ten-centimeter microwaves." IEEE Trans Biomed Eng 11(4): 123-135.	Frequency not relevant, electric current application, co-exposure or exposure condition unclear
Emmanouilides, C. (2008). "Anticancer effects on leiomyosarcoma-bearing Wistar rats after electromagnetic radiation of resonant radiofrequencies." Hell J Nucl Med 11(2): 122	Type of study not relevant or study not EMF/health-related
Eriksson, M. And M. Karlsson (1992). "Occupational and other environmental factors and multiple myeloma: a population based case-control study." British Journal of Industrial Medicine 49(2): 95-103.	Frequency not relevant, electric current application, co-exposure or exposure condition unclear
Esparza-Miñana, J. M. And G. Mazzinari (2019). "Adaptation of an Ultrasound-Guided Technique for Pulsed Radiofrequency on Axillary and Suprascapular Nerves in the Treatment of Shoulder Pain." Pain Med 20(8): 1547-1550.	Frequency not relevant, electric current application, co-exposure or exposure condition unclear
Essman, L. And C. S. Wise (1950). "Local effects of microwave radiation on tissues in the albino rat." Arch Phys Med Rehabil 31(8): 502-507.	Frequency not relevant, electric current application, co-exposure or exposure condition unclear
Eulitz, C., et al. (1998). "Mobile phones modulate response patterns of human brain activity." neuroreport 9(14): 3229-3232.	Frequency not relevant, electric current application, co-exposure or exposure condition unclear
Euma, D. Adang and A. Vander Vorst (2004). Long-term epidemiological study on low-level microwave exposure of rats: 665-668.	Type of study not relevant or study not EMF/health-related
Fabricius, H. A., et al. (1978). "[Changes in clinical and immunological laboratory parameters of healthy adults after exposure to a one-hour 40 degree C-whole-body hyperthermia (author's transl)]." Klin Wochenschr 56(21): 1049-1056.	Frequency not relevant, electric current application, co-exposure or exposure condition unclear
Fajardo, L. F., et al. (1988). "Hyperthermia inhibits angiogenesis." Radiation Research 114(2): 297-306.	Frequency not relevant, electric current application, co-exposure or exposure condition unclear
Feldman, Y., et al. (2008). "Human skin as arrays of helical antennas in the millimeter and submillimeter wave range." Physical Review Letters 100(12): 128102.	Frequency not relevant, electric current application, co-exposure or exposure condition unclear
Fincham, S. M., et al. (2000). "Is occupation a risk factor for thyroid cancer? Canadian Cancer Registries Epidemiology Research Group." Journal of Occupational and Environmental Medicine 42(3): 318-322.	Frequency not relevant, electric current application, co-exposure or exposure condition unclear

Finger, P. T., et al. (1984). "Hyperthermic treatment of intraocular tumors." Arch Ophthalmol 102(10): 1477-1481.	RF EMF used to heat (> 42°C) and/or destroy tissue
Finkelstein, M. M. (1998). "Cancer incidence among Ontario police officers." American Journal of Industrial Medicine 34(2): 157-162.	Frequency not relevant, electric current application, co-exposure or exposure condition unclear
Flaherty, J. A. (1994). "The effect of non ionising electromagnetic radiation on RAAF personnel during World War II." Aust Fam Physician 23(5): 902-905.	Type of study not relevant or study not EMF/health-related
Floratos, D. L., et al. (2000). "High energy transurethral microwave thermotherapy for the treatment of patients in urinary retention." The Journal of Urology 163(5): 1457-1460.	Frequency not relevant, electric current application, co-exposure or exposure condition unclear
Foley-Nolan, D., et al. (1992). "Low energy high frequency pulsed electromagnetic therapy for acute whiplash injuries. A double blind randomized controlled study." Scandinavian Journal of Rehabilitation Medicine 24(1): 51-59.	Frequency not relevant, electric current application, co-exposure or exposure condition unclear
Fontani, V., et al. (2012). "Neuropsychophysical optimization by REAC technology in the treatment of: sense of stress and confusion. Psychometric evaluation in a randomized, single blind, sham-controlled naturalistic study." Patient Preference and Adherence 6: 195-199.	Frequency not relevant, electric current application, co-exposure or exposure condition unclear
Fontani, V., et al. (2012). "Noninvasive radioelectric asymmetric conveyor brain stimulation treatment improves balance in individuals over 65 suffering from neurological diseases: pilot study." Therapeutics and Clinical Risk Management 8: 73-78.	Frequency not relevant, electric current application, co-exposure or exposure condition unclear
Foster, K. R. And E. D. Finch (1974). "Microwave hearing: evidence for thermoacoustic auditory stimulation by pulsed microwaves." Science 185(147): 256-258.	Type of study not relevant or study not EMF/health-related
Freeman, G. And G. E. Gibson (1984). "Stress Indexes in Blood of Animals Restrained for Focused Microwave Irradiation." Federation Proceedings 43(3): 772-772.	Type of study not relevant or study not EMF/health-related
Frei, M. R. And J. R. Jauchem (1992). "Thermoregulatory responses of rats exposed to 9.3-GHz microwaves: a comparison of E and H orientation." Physiological Chemistry and Physics and Medical NMR 24(1): 1-10.	No control group/condition

Frei, M. R., et al. (1990). "Field orientation effects during 5.6-GHz radiofrequency irradiation of rats." Aviation, Space & Environmental Medicine 61(12): 1125-1129.	No control group/condition
Frey, A. H. (1961). "Auditory system response to radio-frequency energy." Aerospace Medicine 32: 1140-1142.	Frequency not relevant, electric current application, co-exposure or exposure condition unclear
Frey, A. H. (1962). "Human auditory system responce to modulated electromagnetic energy." Journal of Applied Physiology 17(4): 689-692.	Type of study not relevant or study not EMF/health-related
Frey, A. H. (1984). "Possible modification of the blood-vitreous humor barrier of the eye with electromagnetic energy." Journal of Bioelectricity 3(1-2): 281-292.	Frequency not relevant, electric current application, co-exposure or exposure condition unclear
Frey, A. H. And E. Coren (1979). "Holographic assessment of a hypothesized microwave hearing mechanism." Science 206(4415): 232-234.	Frequency not relevant, electric current application, co-exposure or exposure condition unclear
Frey, A. H. And E. S. Eichert (1986). "Modification of the heart function with low intensity electromagnetic energy." Journal of Bioelectricity 5(2): 201-210.	Non-mammal/in vitro study
Frey, A. H. And J. Spector (1986). "Exposure to RF electromagnetic energy decreases aggressive behavior." Aggressive Behavior 12(4): 285-291.	Frequency not relevant, electric current application, co-exposure or exposure condition unclear
Frey, A. H. And L. S. Wesler (1983). "Dopamine receptors and microwave energy exposure." Journal of Bioelectricity 2(2-3): 145-157.	Frequency not relevant, electric current application, co-exposure or exposure condition unclear
Frey, A. H. And L. S. Wesler (1984). "Morphine effects appear to be potentiated by microwave energy exposure." Journal of Bioelectricity 3(3): 373-383.	Frequency not relevant, electric current application, co-exposure or exposure condition unclear
Frey, A. H. And L. S. Wesler (1990). "Interaction of psychoactive drugs with exposure to electromagnetic fields." Journal of Bioelectricity 9(2): 187-196.	Frequency not relevant, electric current application, co-exposure or exposure condition unclear
Frey, A. H. And R. Messenger Jr (1973). "Human perception of illumination with pulsed ultrahigh-frequency electromagnetic energy." Science 181(97): 356-358.	Frequency not relevant, electric current application, co-exposure or exposure condition unclear
Friedenthal, E., et al. (1982). "Clinical experiences with combined hyperthermia and radiotherapy in the treatment of cancer." Progress in Clinical and Biological Research 107: 751-760.	Frequency not relevant, electric current application, co-exposure or exposure condition unclear

Fritz, K., et al. (2016). "A novel selective RF applicator for reducing thigh circumference: a clinical evaluation." Dermatol Ther 29(2): 92-95.	Frequency not relevant, electric current application, co-exposure or exposure condition unclear
Fritzer, G., et al. (2007). "Effects of short- and long-term pulsed radiofrequency electromagnetic fields on night sleep and cognitive functions in healthy subjects." Bioelectromagnetics 28(4): 316-325.	Frequency not relevant, electric current application, co-exposure or exposure condition unclear
Frykberg, R. G., et al. (2011). "The Use of Pulsed Radio Frequency Energy Therapy in Treating Lower Extremity Wounds: Results of a Retrospective Study of a Wound Registry." Ostomy Wound Management 57(3): 22-29.	Frequency not relevant, electric current application, co-exposure or exposure condition unclear
Fujita, S., et al. (2012). "Combined microwave irradiation and intraarticular glutamine administration-induced HSP70 expression therapy prevents cartilage degradation in a rat osteoarthritis model." J Orthop Res 30(3): 401-407.	Frequency not relevant, electric current application, co-exposure or exposure condition unclear
Gabrhelik, T., P. Michalek and M. Adamus (2011). "Pulsed Radiofrequency Therapy versus Greater Occipital Nerve Block in the Management of Refractory Cervicogenic Headache - A Pilot Study." Prague Medical Report 112(4): 279-287.	Frequency not relevant, electric current application, co-exposure or exposure condition unclear
Gabriel, C. (1998). "Biological effects of RF and microwaves." Measurement & Control 31(6): 166-169.	Type of study not relevant or study not EMF/health-related
Gadzhiev, A. M., L. F. Ismailova and A. B. Shabanova (2004). "Adaptive changes of redox status in rat brain tissues due to decimeter microwave irradiation." Journal Of Neurochemistry 88: 35-35.	Type of study not relevant or study not EMF/health-related
Gagnon, Z. E., V. A. Fernandez and J. A. Conetta (2000). "The effects of broad-band electromagnetic field exposure on mice (Mus musculus)." Journal of Environmental Science and Health, Part A: Toxic/Hazardous Substances & Environmental Engineering 35(3): 299-311.	Frequency not relevant, electric current application, co-exposure or exposure condition unclear
Gajta, A., et al. (2021). "The effects of microwave radiation generated by mobile phones on the tear film." Revista Română de Medicină de Laborator 29(3): 277-286.	Frequency not relevant, electric current application, co-exposure or exposure condition unclear
Gangwar, V., et al. (2023). "Association of Autonomic Balance with Phone Call Duration in Healthy Individuals." Cureus 15(1): e33566.	Frequency not relevant, electric current application, co-exposure or exposure condition unclear

Gao, Y., et al. (2023). "Modifiable factors for benign salivary gland neoplasms: A Mendelian randomization study." Oral Diseases.	Frequency not relevant, electric current application, co-exposure or exposure condition unclear
Garaj-Vrhovac, V. (1999). "Micronucleus assay and lymphocyte mitotic activity in risk assessment of occupational exposure to microwave radiation." Chemosphere 39(13): 2301-2312.	Frequency not relevant, electric current application, co-exposure or exposure condition unclear
Garaj-Vrhovac, V. And A. Fucic (1993). "The rate of elimination of chromosomal aberrations after accidental exposure to microwave radiation." Bioelectrochemistry and Bioenergetics 30: 319-325.	Frequency not relevant, electric current application, co-exposure or exposure condition unclear
Garajvrhovac, V., A. Fucic and B. Pevalekkozlina (1993). "The Rate Of Elimination Of Chromosomal-Aberrations After Accidental Exposure To Microwave-Radiation." Bioelectrochemistry And Bioenergetics 30(1-3): 319-325.	Frequency not relevant, electric current application, co-exposure or exposure condition unclear
Garaj-Vrhovac, V., A. Fucic and D. Horvat (1990). "Comparison of chromosome aberration and micronucleus induction in human lymphocytes after occupational exposure to vinyl chloride monomer and microwave radiation." Periodicum Biologorum 92(4): 411-416.	Frequency not relevant, electric current application, co-exposure or exposure condition unclear
Garaj-Vrhovac, V., et al. (2011). "Assessment of cytogenetic damage and oxidative stress in personnel occupationally exposed to the pulsed microwave radiation of marine radar equipment." International Journal of Hygiene and Environmental Health 214(1): 59-65.	Type of study not relevant or study not EMF/health-related
Garson, O. M., et al. (1991). "A chromosomal study of workers with long-term exposure to radio-frequency radiation." The Medical Journal of Australia 155(5): 289-292.	Frequency not relevant, electric current application, co-exposure or exposure condition unclear
Garvanova, M., I. Garvanov and D. Borissova (2020). The Influence of Electromagnetic Fields on Human Brain, IEEE: 1-4.	Frequency not relevant, electric current application, co-exposure or exposure condition unclear
Garyaev, P. P., et al. (2007). "Effect of electromagnetic radiation modulated by biostructures on the course of alloxan-induced diabetes mellitus in rats." Bulletin Of Experimental Biology And Medicine 143(2): 197-199.	Frequency not relevant, electric current application, co-exposure or exposure condition unclear

Gavrish, N. N., et al. (2022). "Evaluation of the Biological Effects of Ozone and Electromagnetic Pulses under Combined Exposure from an Electric Discharge Unit." Biology Bulletin 49(12): 2336-2341.	Frequency not relevant, electric current application, co-exposure or exposure condition unclear
Geronikolou, S. A., et al. (2023). "Neuroendocrine System Adaptation during Consecutive Extrinsic Stimuli: A Pilot Dynamic Study." Children 10(2): 248.	Frequency not relevant, electric current application, co-exposure or exposure condition unclear
Ghandehari, M., D. Sadri and S. Farhadi (2021). "Micronucleus Assay in Cell Phone Users: Importance of Oral Mucosa Screening." International Journal of Preventive Medicine 12: 125.	Frequency not relevant, electric current application, co-exposure or exposure condition unclear
Ghosn, R., et al. (2015). "Radiofrequency signal affects alpha band in resting electroencephalogram." Journal of Neurophysiology 113(7): 2753-2759.	Frequency not relevant, electric current application, co-exposure or exposure condition unclear
Glaser, D. A., et al. (2012). "A Randomized, Blinded Clinical Evaluation of a Novel Microwave Device for Treating Axillary Hyperhidrosis: The Dermatologic Reduction in Underarm Perspiration Study." Dermatologic Surgery 38(2): 185-191.	Frequency not relevant, electric current application, co-exposure or exposure condition unclear
Glushkova, O. V., et al. (2003). "Immunocorrective effects of ultra high-frequency waves on tumor-bearing mice." Biofizika 48(2): 281-288.	Article not in English/German
Glushkova, O. V., et al. (2007). "Effects of centimeter waves on the immune system of mice in endotoxic shock." Biofizika 52(5): 938-946.	Article not in English/German
Goerres, H. P. And K. Gerbert (1976). "Sex ratio in offspring of pilots: a contribution to stress research." Aviat Space Environ Med 47(8): 889-892.	Frequency not relevant, electric current application, co-exposure or exposure condition unclear
Gofeld, M., et al. (2013). "Pulsed radiofrequency of suprascapular nerve for chronic shoulder pain: a randomized double-blind active placebo-controlled study." Pain Practice 13(2): 96-103.	Frequency not relevant, electric current application, co-exposure or exposure condition unclear
Gold, J. H., et al. (1973). "Life-Span Of Mice Exposed To Radio-Frequency Electromagnetic Fields." Journal Of The Electrochemical Society 120(3): C115-+.	Type of study not relevant or study not EMF/health-related
Gold, M. H., et al. (2017). "Safety, efficacy, and usage compliance of home-use device utilizing RF and light energies for treating periorbital wrinkles." Journal of Cosmetic Dermatology 16(1): 95-102.	Frequency not relevant, electric current application, co-exposure or exposure condition unclear

Goldoni, J. (1990). "Hematological changes in peripheral blood of workers occupationally exposed to microwave radiation." Health Physics 58(2): 205-207.	Frequency not relevant, electric current application, co-exposure or exposure condition unclear
Goldoni, J., M. Durek and Z. Koren (1993). "Health status of personnel occupationally exposed to radiowaves." Arhiv za Higijenu Rada i Toksikologiju (Archives of Industrial Hygiene and Toxicology) 44(3): 223-228.	Frequency not relevant, electric current application, co-exposure or exposure condition unclear
Goldsmith, J. R. (1996). "Balancing the interests of patients, science and employees: Case study of RF (microwave) exposures of US embassy staff in eastern European posts." Science of the Total Environment 184(1-2): 83-89.	Frequency not relevant, electric current application, co-exposure or exposure condition unclear
Goldsmith, J. R. (1997). "Epidemiologic evidence relevant to radar (microwave) effects." Environmental Health Perspectives 105: 1579-1587.	Type of study not relevant or study not EMF/health-related
Golomb, B. A. (2018). "Diplomats' Mystery Illness and Pulsed Radiofrequency/Microwave Radiation." Neural Comput 30(11): 2882-2985.	Type of study not relevant or study not EMF/health-related
Gonzalez-Rubio, J., et al. (2017). "Radiofrequency electromagnetic fields and some cancers of unknown etiology: An ecological study." Science of the Total Evironment 599-600: 834-843.	Type of study not relevant or study not EMF/health-related
Gorbans, I. And A. Jurenoks (2019). "Some Aspects of Good Practice for Safe Use of Wi-Fi, Based on Experiments and Standards." Applied Computer Systems 24(2): 161-165.	Non-mammal/in vitro study
Gordon, C. J. (1983). "Behavioral And Autonomic Thermoregulation in Mice Exposed To Microwave-Radiation." Federation Proceedings 42(4): 744-744.	Type of study not relevant or study not EMF/health-related
Gordon, C. J. And E. C. White (1982). "Distinction Between Heating Rate And Total Heat Absorption In The Microwave-Exposed Mouse." Physiological Zoology 55(3): 300-308.	Frequency not relevant, electric current application, co-exposure or exposure condition unclear
Gordon, C. J. And M. D. Long (1984). "Ventilatory frequency of mouse and hamster during microwave-induced heat exposure." Respiration Physiology 56(1): 81-90.	Frequency not relevant, electric current application, co-exposure or exposure condition unclear

Grayson, J. K. (1996). "Radiation exposure, socioeconomic status, and brain tumor risk in the US Air Force: a nested case-control study." American Journal of Epidemiology 143(5): 480-486.	Frequency not relevant, electric current application, co-exposure or exposure condition unclear
Grigoriev, Y. G., et al. (2010). "Autoimmune processes after long-term low-level exposure to electromagnetic fields (experimental results). Part 1. Mobile communications and changes in electromagnetic conditions for the population. Need for additional substantiation of existing hygienic s." Biophysics 55(6): 1041-1045.	Frequency not relevant, electric current application, co-exposure or exposure condition unclear
Groves, F. D., et al. (2002). "Cancer in Korean war navy technicians: mortality survey after 40 years." American Journal of Epidemiology 155(9): 810-818.	Frequency not relevant, electric current application, co-exposure or exposure condition unclear
Guberan, E., et al. (1994). "Gender ratio of offspring and exposure to shortwave radiation among female physiotherapists." Scandinavian Journal of Work, Environment & Health 20(5): 345-348.	Frequency not relevant, electric current application, co-exposure or exposure condition unclear
Gudzkova, T. N., et al. (2011). "Morphofuctional aspects of antitumor activity of low-intensity microwave resonance radiation in experiment." Bull Exp Biol Med 150(5): 659-663.	Frequency not relevant, electric current application, co-exposure or exposure condition unclear
Guerriero, F., et al. (2015). "An innovative intervention for the treatment of cognitive impairment-Emisymmetric bilateral stimulation improves cognitive functions in Alzheimer's disease and mild cognitive impairment: an open-label study." Neuropsychiatric Disease and Treatment 11: 2391-2404.	No control group/condition
Guessab, A., G. Lescoat and J. Maniey (1983). "Influence of postnatal exposition to microwaves on brain and hypothalamo-pituitary monoamines in the adult male rat." Physiologie 20(2): 71-74.	Frequency not relevant, electric current application, co-exposure or exposure condition unclear
Guo, H., et al. (2011). "Comparing different physical factors on serum TNF-alpha levels, chondrocyte apoptosis, caspase-3 and caspase-8 expression in osteoarthritis of the knee in rabbits." Joint, Bone, Spine 78(6): 604-610.	Frequency not relevant, electric current application, co-exposure or exposure condition unclear
Guo, K., et al. (2020). "Effects of acute exposure to ultra-wideband pulsed electromagnetic fields on the liver and kidneys of mice." Electromagnetic Biology and Medicine 39(2): 109-122.	Frequency not relevant, electric current application, co-exposure or exposure condition unclear

Gupta, A., et al. (2018). Microwave frequency-electromagnetic field 10 ghz radiation exposure impact on rat skin: An oxidative stress insight, IEEE: 18.	Article not peer-reviewed
Gura, E. N. And E. M. Gonchar (2006). "Microwave-Induced and Pharmacological Suppression of Somatic Pain under Conditions of the Formalin Test in Mice." Neurophysiology 38(1): 39-44.	Frequency not relevant, electric current application, co-exposure or exposure condition unclear
Gura, E. V., E. V. Bagatskaya and Y. P. Limanskii (2002). "Involvement of the Serotonergic System in Analgesia Induced by the Influence of Low-Intensity Microwaves on an Antipain Acupuncture Point." Neurophysiology 34(4): 291-296.	Frequency not relevant, electric current application, co-exposure or exposure condition unclear
Gura, Y. V. And Y. V. Bagatskaya (2012). "Analgesic Effects of Preliminary Microwave Irradiation of an Acupuncture Point on Visceral Pain in Mice." Neurophysiology 44(3): 234-239.	Frequency not relevant, electric current application, co-exposure or exposure condition unclear
Guttari, B. (1989). "Simultaneous Quantitative High-Performance Liquid-Chromatographic Determination of Cyclic Adenosine-3',5'-Monophosphate And Adenosine In Mouse-Brain After Microwave Irradiation - Application To Isoprenaline Effect Invivo." Journal Of Chromatography-Biomedical Applications 489(2): 394-398.	Type of study not relevant or study not EMF/health-related
Guy, A. W., et al. (1975). "Microwave-induced acoustic effects in mammalian auditory systems and physical materials." Annals Of The New York Academy of Sciences 247: 194-218.	No control group/condition
Guy, A. W., et al. (1976). "Proceedings: Study of the effects of chronic low level microwave radiation on rabbits." The Journal of Microwave Power 11(2): 134-135.	Frequency not relevant, electric current application, co-exposure or exposure condition unclear
Guy, A. W., et al. (1980). "Long-term 2450 mhz CW microwave irradiation of rabbits: Methodology and evaluation of ocular and physiologic effects." The Journal of Microwave Power 15(1): 37-44.	Frequency not relevant, electric current application, co-exposure or exposure condition unclear
Hallberg, O., O. Johansson and H. Eger (2016). "A melanoma trend forecast from 2002 - what happened then?" Electromagnetic Biology and Medicine 35(2): 103-105.	Frequency not relevant, electric current application, co-exposure or exposure condition unclear

Hamburger, S., J. N. Logue and P. M. Silverman (1983). "Occupational exposure to non-ionizing radiation and an association with heart disease: an exploratory study." Journal of Chronic Diseases 36(11): 791-802.	Frequency not relevant, electric current application, co-exposure or exposure condition unclear
Hamilton, P. M., et al. (1984). "Radiation procedures performed on U.S. women during pregnancy: findings from two 1980 surveys." Public Health Rep 99(2): 146-151.	Frequency not relevant, electric current application, co-exposure or exposure condition unclear
Hao, Y. H., et al. (2018). "HIF-1 alpha regulates COXIV subunits, a potential mechanism of self-protective response to microwave induced mitochondrial damages in neurons." Scientific Reports 8.	Frequency not relevant, electric current application, co-exposure or exposure condition unclear
Hao, Y. H., et al. (2018). "HIF-1α regulates COXIV subunits, a potential mechanism of self-protective response to microwave induced mitochondrial damages in neurons." Scientific Reports 8: 10403.	Frequency not relevant, electric current application, co-exposure or exposure condition unclear
Hao, Y., et al. (2018). "Autophagy mediates the degradation of synaptic vesicles: A potential mechanism of synaptic plasticity injury induced by microwave exposure in rats." Physiology & Behavior 188: 119-127.	Frequency not relevant, electric current application, co-exposure or exposure condition unclear
Hao, Y., et al. (2022). "High-Power Electromagnetic Pulse Exposure of Healthy Mice: Assessment of Effects on Mice Cognitions, Neuronal Activities, and Hippocampal Structures." Frontiers in Cellular Neuroscience 16: 898164.	Frequency not relevant, electric current application, co-exposure or exposure condition unclear
Hao, Y., et al. (2022). "Microwave radiation induces neuronal autophagy through mir-30a-5p/ampkα2 signal pathway." Bioscience Reports 42(4): BSR20212584.	Frequency not relevant, electric current application, co-exposure or exposure condition unclear
Hardell, L., et al. (1998). "Case-control study on risk factors for testicular cancer." International Journal of Oncology 13(6): 1299-1303.	Frequency not relevant, electric current application, co-exposure or exposure condition unclear
Hartard, M., et al. (2023). "Electromagnetic Induction for Treatment of Unspecific Back Pain: A Prospective Randomized Sham-Controlled Clinical Trial." Journal of Rehabilitation Medicine 55: jrm00389.	Frequency not relevant, electric current application, co-exposure or exposure condition unclear

Hässig, M., et al. (2015). "Gesundheitliche Probleme von Nutztieren mit Verdacht auf nichtionisierende Strahlung oder elektrische Kriechströme als Ursache: Ergebnis einer Fragebogenerhebung." Schweizer Archiv für Tierheilkunde 157(11): 615-617.	Frequency not relevant, electric current application, co-exposure or exposure condition unclear
Hatano, T., et al. (2021). "Pathological Changes in Axillary Hyperhidrosis and Axillary Osmidrosis Induced by Microwave Treatment: Comparison of Single- and Double-Pass Irradiation." Lasers Surg Med 53(9): 1220-1226.	Frequency not relevant, electric current application, co-exposure or exposure condition unclear
Hatch, E. E., et al. (2021). "Male cellular telephone exposure, fecundability, and semen quality: results from two preconception cohort studies." Human Reproduction 36(5): 1395-1404.	Frequency not relevant, electric current application, co-exposure or exposure condition unclear
Hathaway, J. A., et al. (1977). "Ocular medical surveillance on microwave and laser workers." Journal of Occupational Medicine 19(10): 683-688.	Frequency not relevant, electric current application, co-exposure or exposure condition unclear
Hawkins, S. F., M. A. Medina and W. B. Stavinoha (1977). "Measurement Of Atp, Adp And Amp In Mouse Lung After Inactivation By Microwave Irradiation." Federation Proceedings 36(3): 480-480.	Type of study not relevant or study not EMF/health-related
Hayes, R. B., et al. (1990). "Occupation And Risk For Testicular Cancer - A Case-Control Study." International Journal Of Epidemiology 19(4): 825-831.	Frequency not relevant, electric current application, co-exposure or exposure condition unclear
Hayes, R. B., et al. (1990). "Occupation and risk for testicular cancer: a case-control study." International Journal Of Epidemiology 19(4): 825-831.	Frequency not relevant, electric current application, co-exposure or exposure condition unclear
Hayre, N., M. Palm and P. Jenkin (2016). "A Clinical Evaluation of a Next Generation, Non-Invasive, Selective Radiofrequency, Hands-Free, Body-Shaping Device." Journal Of Drugs In Dermatology 15(12): 1557-1561.	Frequency not relevant, electric current application, co-exposure or exposure condition unclear
He, G. L., et al. (2021). "The involvement of microglial CX3CR1 in heat acclimation-induced amelioration of adult hippocampal neurogenesis impairment in EMF-exposed mice." Brain Research Bulletin 177: 181-193.	Frequency not relevant, electric current application, co-exposure or exposure condition unclear

Heikkinen, P., et al. (1999). "Chronic exposure to 50-Hz magnetic fields or 900-mhz electromagnetic fields does not alter nocturnal 6-hydroxymelatonin sulfate secretion in CBA/S mice." Electro- and Magnetobiology 18(1): 33-42.	Frequency not relevant, electric current application, co-exposure or exposure condition unclear
Heinmets, F. (1982). "Pilot Experiments on Temperature Cycling In Rats Exposed Repetitively To Radiofrequency Radiation (Rfr)." Physiological Chemistry And Physics 14(6): 519-531.	Frequency not relevant, electric current application, co-exposure or exposure condition unclear
Hendler, E. (1968). Cutaneous Receptor Response to Microwave Irradiation." Thermal Problems in Aerospace Medicine: Proceedings of Two Conferences of the Aerospace Medical Panel of AGARD held at Lisbon (1964) and Furstenfeldbruck (1965). AGARDograph, Band 111; Maidenhead, 1968; 149-161.	Article not peer-reviewed
Hendler, E. And J. D. Hardy (1960). "Infrared and microwave effects on skin heating and temperature sensation." IRE Transactions on Medical Electronics ME-7(3): 143-152.	Type of study not relevant or study not EMF/health-related
Hendler, E., J. D. Hardy and D. Murgatroyd (1963). Skin Heating and Temperature Sensation Produced by Infrared and Microwave Irradiation, Reinhold Publishing Corp.: 211-230.	Article not peer-reviewed
Hjeresen, D. L. And J. Diaz (1988). "Ontogeny of susceptibility to experimental febrile seizures in rats." Dev Psychobiol 21(3): 261-275.	Frequency not relevant, electric current application, co-exposure or exposure condition unclear
Hjollund, N. H., J. P. Bonde and J. Skotte (1997). "Semen analysis of personnel operating military radar equipment." Reproductive Toxicology 11(6): 897.	Frequency not relevant, electric current application, co-exposure or exposure condition unclear
Ho, H. S. And W. P. Edwards (1979). "Effect of Environmental-Temperature and Average Dose-Rate of Microwave Radiation on The Oxygen-Consumption Rate of Mice." Radiation And Environmental Biophysics 16(4): 325-338.	Frequency not relevant, electric current application, co-exposure or exposure condition unclear
Ho, H. S. And W. P. Edwards (1979). "The effect of environmental temperature and average dose rate of microwave radiation on the oxygen-consumption rate of mice." Radiation And Environmental Biophysics 16(4): 325-338.	Frequency not relevant, electric current application, co-exposure or exposure condition unclear

Holtgrewe, M., et al. (2018). "Multisite de novo mutations in human offspring after paternal exposure to ionizing radiation." Sci Rep 8(1): 14611.	Frequency not relevant, electric current application, co-exposure or exposure condition unclear
Hong, S., et al. (2020). "Enriched Environment Decreases Cognitive Impairment in Elderly Rats with Prenatal Mobile Phone Exposure." Frontiers in Aging Neuroscience 12: 162.	Frequency not relevant, electric current application, co-exposure or exposure condition unclear
Hosseini, M. A., et al. (2019). "Evaluating short-term exposure to wi-fi signals on students' reaction time, short-term memory and reasoning ability." Radiation Protection Dosimetry 187(3): 279-285.	Frequency not relevant, electric current application, co-exposure or exposure condition unclear
Hu, H. X., et al. (2019). "Effect of Modified Wuzi Yanzong Pill () on Tip60-Mediated Apoptosis in Testis of Male Rats after Microwave Radiation." Chinese Journal of Integrative Medicine 25(5): 342-347.	Frequency not relevant, electric current application, co-exposure or exposure condition unclear
Huang, P. C., et al. (2022). "Physiological changes and symptoms associated with short-term exposure to electromagnetic fields: a randomized crossover provocation study." Environmental Health 21: 31.	Frequency not relevant, electric current application, co-exposure or exposure condition unclear
Hugander, A., et al. (1983). "Liver blood flow studies during local hyperthermia. An experimental study in rats." Clin Oncol 9(4): 303-310.	Frequency not relevant, electric current application, co-exposure or exposure condition unclear
Hugander, A., et al. (1983). "Total body hyperthermia induced by a computerized microwave technique: studies in normal rats and in rats with liver tumors." Anticancer Res 3(3): 161-165.	Frequency not relevant, electric current application, co-exposure or exposure condition unclear
Huss, A., et al. (2015). "Environmental Radiofrequency Electromagnetic Fields Exposure at Home, Mobile and Cordless Phone Use, and Sleep Problems in 7-Year-Old Children." Plos One 10(10): e0139869.	Frequency not relevant, electric current application, co-exposure or exposure condition unclear
Hussain, I., et al. (2021). "Quantifying Physiological Biomarkers of a Microwave Brain Stimulation Device." Sensors 21(5): 1896.	Frequency not relevant, electric current application, co-exposure or exposure condition unclear
Hussien, N. I., A. M. Mousa and A. A. Shoman (2022). "Decreased level of plasma nesfatin-1 in rats exposed to cell phone radiation is correlated with thyroid dysfunction, oxidative stress, and apoptosis." Archives of Physiology and Biochemistry 128(6): 1486-1492.	Frequency not relevant, electric current application, co-exposure or exposure condition unclear

Hutt, B. K., et al. (1952). "The influence of microwave irradiation on bone temperature in dog and man." Am J Phys Med 31(6): 422-428.	Frequency not relevant, electric current application, co-exposure or exposure condition unclear
Iannitti, T., et al. (2013). "Pulsed electromagnetic field therapy for management of osteoarthritis-related pain, stiffness and physical function: clinical experience in the elderly." Clinical Interventions in Aging 8: 1289-1293.	Frequency not relevant, electric current application, co-exposure or exposure condition unclear
Ibraheim, M. H. And A. Amin (2020). "Ameliorative Effect of Vitamin E against Radiofrequency Radiation Emitted from Mobile Phone-Induced Hematological and Histopathological Alterations in Male Albino Mice." Arab Journal of Nuclear Sciences and Applications 53(4): 26-38.	Frequency not relevant, electric current application, co-exposure or exposure condition unclear
Igarashi, Y., et al. (2015). "Pathophysiology of microwave-induced traumatic brain injury." Biomedical Reports 3(4): 468-472.	Frequency not relevant, electric current application, co-exposure or exposure condition unclear
Ikarashi, Y. And Y. Maruyama (1984). "High-Performance Liquid-Chromatographic Analysis Of Regional Catecholamines And 3,4-Dihydroxyphenylacetic Acid In Rat-Brain Following Microwave Irradiation." Biogenic Amines 1(4): 341-357.	Frequency not relevant, electric current application, co-exposure or exposure condition unclear
Int Radiat Protect, A., et al. (1992). Electrophysiological Changes In Rats After Modulated Microwave Irradiation: 1140-1143.	Article not peer-reviewed
Jamakosmanovic, A., et al. (1981). "The Levels of ATP, ADP and AMP in Rat Brain during Postnatal Development Following "in Utero" 2450 mhz Microwave Irradiation." Periodicum Biologorum 83: 151-152.	Frequency not relevant, electric current application, co-exposure or exposure condition unclear
Jamakosmanovic, A., et al. (1985). "Effects Of Different Repetition Rates And Pulse Widths On The Level Of Atp, Adp And Amp In Rat-Brain Following Repetitive 2450 Mhz Microwave Irradiation." Periodicum Biologorum 87(2): 233-234.	Frequency not relevant, electric current application, co-exposure or exposure condition unclear
Jamakosmanovic, A., M. Drecun and L. M. Shore (1979). "Observations On Eeg Response Of Young-Rats Following Chronic Whole-Body 2450 Mhz Microwave Irradiation." Periodicum Biologorum 81(1): 40-40.	Frequency not relevant, electric current application, co-exposure or exposure condition unclear

Jamaludin, N., et al. (2017). "The Effect of Smartphone's Radiation Frequency and Exposure Duration on NADPH Oxidase 5 (NOX5) Level in Sperm Parameters." Sains Malaysiana 46(9): 1597-1602.	Frequency not relevant, electric current application, co-exposure or exposure condition unclear
Jarideh, S., et al. (2015). "Does Occupational Exposure of Shahid Dastghieb International Airport Workers to Radiofrequency Radiation Affect Their Short Term Memory and Reaction Time?" Journal of Biomedical Physics & Engineering 5(3): 143-150.	Frequency not relevant, electric current application, co-exposure or exposure condition unclear
Jauchem, J. R., et al. (1998). "Ultra-wideband electromagnetic pulses: lack of effects on heart rate and blood pressure during two-minute exposures of rats." Bioelectromagnetics 19(5): 330-333.	Frequency not relevant, electric current application, co-exposure or exposure condition unclear
Jauchem, J. R., K. L. Ryan and M. R. Frei (2000). "Cardiovascular and thermal effects of microwave irradiation at 1 and/or 10 ghz in anesthetized rats." Bioelectromagnetics 21(3): 159-166.	No control group/condition
Jauchem, J. R., M. R. Frei and F. Heinmets (1988). "Thermal responses to 5.6-ghz radiofrequency radiation in anesthetized rats: effect of chlorpromazine." Physiological Chemistry and Physics and Medical NMR 20(2): 135-143.	No control group/condition
Jeevitha, G. And G. Anuradha (2020). "Effect of hand-held mobile phones on the parotid gland: A cross sectional study." Journal of Indian Academy of Oral Medicine and Radiology 32(4): 335-340.	Frequency not relevant, electric current application, co-exposure or exposure condition unclear
Jevric, A., A. Jamakosmanovic and M. Nakas (1985). "Effects Of Different Repetition Rates And Pulse Widths On The Level Of Serotonin In Rat-Brain Following Repetitive 2450 Mhz Microwave Irradiation." Periodicum Biologorum 87(2): 235-236.	Frequency not relevant, electric current application, co-exposure or exposure condition unclear
Jevric, A., et al. (1983). "The Effects Of Repeated Whole-Body 2450mhz Microwave-Radiation On The Levels Of Serotonin, Adp And Inorganic-Phosphate In The Rat-Brain." Periodicum Biologorum 85(2): 206-207.	Frequency not relevant, electric current application, co-exposure or exposure condition unclear
Jia, F., et al. (2007). "Role of blood flow on RF exposure induced skin temperature elevations in rabbit ears." Bioelectromagnetics 28(3): 163-172.	Frequency not relevant, electric current application, co-exposure or exposure condition unclear

Jiang, D. P., et al. (2013). "Electromagnetic pulse exposure induces overexpression of beta amyloid protein in rats." Archives of Medical Research 44(3): 178-184.	Frequency not relevant, electric current application, co-exposure or exposure condition unclear
Johnson, L., R. M. Lebovitz and W. K. Samson (1984). "Germ-Cell Degeneration In Normal And Microwave Irradiated Rats." Journal Of Andrology 5(1): P28-P28.	Type of study not relevant or study not EMF/health-related
Jung, J. G., et al. (2017). "Effectiveness of pulsed electromagnetic field for pain caused by placement of initial orthodontic wire in female orthodontic patients: A preliminary single-blind randomized clinical trial." American Journal of Orthodontics and Dentofacial Orthopedics 152(5): 582-591.	Frequency not relevant, electric current application, co-exposure or exposure condition unclear
Justesen, D. R. And N. W. King (1970). Behavioral effects of low level microwave irradiation in the closed space situation, U.S. Department of Health, Education, and Welfare (HEW), Public Health Service (PHS), Bureau of Radiological Health (BRH)): 154-179.	Frequency not relevant, electric current application, co-exposure or exposure condition unclear
Kacprzyk, A., et al. (2020). "Does the short-term exposure to radiofrequency electromagnetic field originating from mobile phone affect auditory functions as measured by Acoustic Admittance and Evoked Otoacoustic Emission tests?" Electromagnetic Biology and Medicine 39(4): 411-418.	Frequency not relevant, electric current application, co-exposure or exposure condition unclear
Kadeh, H., et al. (2023). "A Comparative Evaluation of the Genotoxic Effects of Mobile Phone Radiation Using Buccal Micronucleus Assay." Journal of Dentistry 24(1): 118-124.	Frequency not relevant, electric current application, co-exposure or exposure condition unclear
Kakigi, R., et al. (2011). "Heat stress enhances mtor signaling after resistance exercise in human skeletal muscle." The Journal of Physiological Sciences 61(2): 131-140.	Frequency not relevant, electric current application, co-exposure or exposure condition unclear
Kallen, B., G. Malmquist and U. Moritz (1982). "Delivery outcome among physiotherapists in Sweden: is non-ionizing radiation a fetal hazard?" Archives of Environmental Health 37(2): 81-85.	Frequency not relevant, electric current application, co-exposure or exposure condition unclear
Kamal, A. (2001). "Effect of low pulsed microwaves on heart rate variability signals in mammals." Neurosciences (Riyadh) 6(1): 26-29.	No control group/condition

Kaminaka, C., et al. (2019). "Clinical and histological evaluation of a single high energy microwave treatment for primary axillary hyperhidrosis in Asians: A prospective, randomized, controlled, split-area comparative trial." Lasers in Surgery and Medicine 51(7): 592-599.	Frequency not relevant, electric current application, co-exposure or exposure condition unclear
Kantz, J., et al. (2005). "Insensitivity of cardiovascular function to low power cm-/mm-microwaves." International Journal of Environmental Health Research 15(3): 207-215.	Frequency not relevant, electric current application, co-exposure or exposure condition unclear
Kao, H. K., et al. (2013). "Collagen synthesis modulated in wounds treated by pulsed radiofrequency energy." Plastic & Reconstructive Surgery 131(4): 490e-498e.	Frequency not relevant, electric current application, co-exposure or exposure condition unclear
Kaplan, J., et al. (1982). "Biological and behavioral effects of prenatal and postnatal exposure to 2450-mhz electromagnetic radiation in the squirrel monkey." Radio Science 17(5): 135S-144S.	Frequency not relevant, electric current application, co-exposure or exposure condition unclear
Kaplan, S., et al. (1997). "Occupational risks for the development of brain tumors." American Journal of Industrial Medicine 31(1): 15-20.	Frequency not relevant, electric current application, co-exposure or exposure condition unclear
Karinen, A., et al. (2008). "Mobile phone radiation might alter protein expression in human skin." BMC Genomics 9: 77.	Frequency not relevant, electric current application, co-exposure or exposure condition unclear
Karipidis, K. K., et al. (2007). "Occupational exposure to ionizing and non-ionizing radiation and risk of glioma." Occupational Medicine (Oxford, England) 57(7): 518-524.	Frequency not relevant, electric current application, co-exposure or exposure condition unclear
Karlov, V. A., et al. (1991). "Uhf Therapy Of Millimetric Range For Vascular Disturbances Of The Brain." Sovetskaya Meditsina(3): 20-21.	Article not in English/German
Karuserci, O. K., N. Col and C. Demirel (2019). "May electromagnetic field exposure during pregnancy have a negative effect on anthropometric measurements of the newborn?" Cukurova Medical Journal 44: 290-295.	Frequency not relevant, electric current application, co-exposure or exposure condition unclear
Kása, P., K. Bánsághy and K. Gulya (1982). "Ultrastructural changes and diffusion of acetylcholine in rat brain after microwave irradiation." J Neurosci Methods 5(3): 215-220.	Frequency not relevant, electric current application, co-exposure or exposure condition unclear
Kaya, C., et al. (2020). "The effect of modifiable lifestyle factors on semen quality." Revista Internacional de Andrologia 18(4): 151-158.	Frequency not relevant, electric current application, co-exposure or exposure condition unclear

Kelley, J. T., et al. (1984). "The relationship between flash evoked potentials and evoked amplitude modulation patterns of an applied UHF electromagnetic field in the rat." Bioelectromagnetics 5(4): 365-375.	Frequency not relevant, electric current application, co-exposure or exposure condition unclear
Kemerov, S., M. Marinkev and D. Getova (1999). "Effects of low-intensity electromagnetic fields on behavioral activity of rats." Folia Medica (Plovdiv) 41(3): 75-80.	Frequency not relevant, electric current application, co-exposure or exposure condition unclear
Kerns, J. M. And H. P. Weinrib (1986). "Microwave irradiation of peripheral nerve following injury: a structural analysis of degeneration and regeneration." Experimental Neurology 92(3): 651-664.	Frequency not relevant, electric current application, co-exposure or exposure condition unclear
Kesari, K. K., S. Kumar and J. Behari (2011). "Pathophysiology Of Microwave Radiation: Mutagenic Effect On Rat Brain." European Journal Of Neurology 18: 305-305.	Type of study not relevant or study not EMF/health-related
Khaki-Khatibi, F., et al. (2019). "Relationship between the use of electronic devices and susceptibility to multiple sclerosis." Cognitive Neurodynamics 13(3): 287-292.	Frequency not relevant, electric current application, co-exposure or exposure condition unclear
Khalil, A. M., et al. (2022). "Mobile phone radiation might alter gene expression in the oral squamous epithelial cells." Egyptian Journal of Medical Human Genetics 23(1): 12.	Frequency not relevant, electric current application, co-exposure or exposure condition unclear
Khattab, N. F. And E. S. Marei (2019). "The role of ferulic acid in the amelioration of kidney changes of rats exposed to electromagnetic radiation." International Journal of Radiation Research 17(1): 75-85.	Frequency not relevant, electric current application, co-exposure or exposure condition unclear
Khodamoradi, E., et al. (2022). "Simultaneous effect of gamma and Wi-Fi radiation on gamma-H2Ax expression in peripheral blood of rat: A radio-protection note." Biochemistry and Biophysics Reports 30: 101232.	Frequency not relevant, electric current application, co-exposure or exposure condition unclear
Kim, D. W., et al. (2009). "Radiofrequency energy delivery to the anal canal: is it a promising new approach to the treatment of fecal incontinence?" American Journal of Surgery 197(1): 14-18.	Frequency not relevant, electric current application, co-exposure or exposure condition unclear
Kim, H. M., et al. (2022). "Radiofrequency Irradiation Mitigated UV-B-Induced Skin Pigmentation by Increasing Lymphangiogenesis." Molecules 27(2).	Frequency not relevant, electric current application, co-exposure or exposure condition unclear

Kim, H. S., et al. (2021). "Biological Effects of Exposure to a Radiofrequency Electromagnetic Field on the Placental Barrier in Pregnant Rats." Bioelectromagnetics 42(3): 191-199.	Frequency not relevant, electric current application, co-exposure or exposure condition unclear
Kim, J. H., et al. (2021). "Exposure to RF-EMF Alters Postsynaptic Structure and Hinders Neurite Outgrowth in Developing Hippocampal Neurons of Early Postnatal Mice." International Journal of Molecular Sciences 22(10): 5340.	Frequency not relevant, electric current application, co-exposure or exposure condition unclear
Kim, Y. A., et al. (1988). "Across The Rat Erythrocyte-Membrane Under Electromagnetic-Radiation Of Radiofrequency Range." Biofizika 33(5): 837-840.	Article not in English/German
Kinoshita, J. H., et al. (1966). "Biochemical changes in microwave cataracts." Documenta Ophthalmologica 20: 91-103.	Frequency not relevant, electric current application, co-exposure or exposure condition unclear
Kittel, A. And Z. Somosy (1994). “Distribution Of Ca-Atpase And Calcium-Ion In The Medial Habenula Of Mouse - Effects Of Low-Energy Microwave Irradiation.” Electron Microscopy 1994: Proceedings of the 13th International Congress on Electron Microscopy. Applications in Biological Sciences, Band 3A; Les Editions de Physique, Les Ulis, 1994: 679-680	Type of study not relevant or study not EMF/health-related
Kittel, A. And Z. Somosy (1994). Distribution of Ca-atpase and Calcium-Ion in the Medial Habenula of Mouse - Effects of Low-Energy Microwave Irradiation, Les Editions de Physique: 679-680.	Frequency not relevant, electric current application, co-exposure or exposure condition unclear
Kittel, A., et al. (1996). "Qualitative enzyme histochemistry and microanalysis reveals changes in ultrastructural distribution of calcium and calcium-activated atpases after microwave irradiation of the medial habenula." Acta Neuropathologica 92(4): 362-368.	Frequency not relevant, electric current application, co-exposure or exposure condition unclear
Klitzing, L. (2021). "Artificial EMG by WLAN-Exposure." Journal of Biostatistics and Biometric Applications 6(1): 101.	Frequency not relevant, electric current application, co-exposure or exposure condition unclear
Klug, S., et al. (1997). "The lack of effects of nonthermal RF electromagnetic fields on the development of rat embryos grown in culture." Life Sciences 61(18): 1789-1802.	Frequency not relevant, electric current application, co-exposure or exposure condition unclear
Kobayashi, K., et al. (1989). "Increase of brain ammonia after microwave irradiation and its mechanism." In Vivo 3(5): 339-343.	Frequency not relevant, electric current application, co-exposure or exposure condition unclear

Kocyigit, U. M., et al. (2018). "The effects of wireless electromagnetic fields on the activities of carbonic anhydrase and acetylcholinesterase enzymes in various tissues of rats." Journal of Biochemical and Molecular Toxicology 32(3): e22031.	Frequency not relevant, electric current application, co-exposure or exposure condition unclear
Kodera, S., et al. (2017). "Multiphysics and Thermal Response Models to Improve Accuracy of Local Temperature Estimation in Rat Cortex under Microwave Exposure." Int J Environ Res Public Health 14(4).	Type of study not relevant or study not EMF/health-related
Koldaev, V. M. (1970). "Effect of UHF electromagnetic field on rats during changes in the intensity of oxidative processes in the organism." Biulleten' Eksperimental'noi Biologii i Meditsiny 70(11): 69-70.	Frequency not relevant, electric current application, co-exposure or exposure condition unclear
Koldaev, V. M. (1973). "Effect of central nervous system stimulators and adrenal hormones on the outcome of acute damage to mice caused by an ultrahighfrequency field." Biulleten' Eksperimental'noi Biologii i Meditsiny 76(9): 27-28.	Article not in English/German
Koldaev, V. M. (1974). "Effect of ephedrine and nikethamide on the outcome of microwave lesions in mice." Bulletin Of Experimental Biology And Medicine 77(3): 298-301.	Frequency not relevant, electric current application, co-exposure or exposure condition unclear
Koldaev, V. M. (1979). "Effect Of General-Anesthetics On Mice After Microwave Irradiation." Bulletin Of Experimental Biology And Medicine 87(5): 438-440.	Frequency not relevant, electric current application, co-exposure or exposure condition unclear
Kolmodin-Hedman, B., et al. (1988). "Health problems among operators of plastic welding machines and exposure to radiofrequency electromagnetic fields." International Archives of Occupational and Environmental Health 60(4): 243-247.	Frequency not relevant, electric current application, co-exposure or exposure condition unclear
Kopani, M., et al. (2022). "PIXE analysis of iron in rabbit cerebellum after exposure to radiofrequency electromagnetic fields." Bratisl Lek Listy 123(12): 864-871.	Frequency not relevant, electric current application, co-exposure or exposure condition unclear
Korenevskiy, N. A., et al. (2022). "An Expert System for Assessing the State of Health of Operators of Human-Machine Systems Exposed to Radio-Frequency Electromagnetic Fields." BIOMEDICAL	Type of study not relevant or study not EMF/health-related

ENGINEERING-MEDITSINSKAYA TEKNIKA 55(5): 350-354.	
Korpan, N. N. And T. Saradeth (1995). "Clinical effects of continuous microwave for postoperative septic wound treatment: a double-blind controlled trial." American Journal of Surgery 170(3): 271-276.	Frequency not relevant, electric current application, co-exposure or exposure condition unclear
Kramer, P., et al. (1976). "Proceedings: Acute microwave irradiation and cataract formation in rabbits and monkeys." J Microw Power 11(2): 135-136.	Type of study not relevant or study not EMF/health-related
Krasnoshchekova, E. I., N. V. Gunko and L. A. Tkachenko (2005). "Histochemical Study of Effects of Weak Electromagnetic Field on Structures of the Rat Midbrain." Journal of Evolutionary Biochemistry and Physiology 41(1): 119-125.	Frequency not relevant, electric current application, co-exposure or exposure condition unclear
Krewski, D., et al. (2001). "Potential health risks of radiofrequency fields from wireless telecommunication devices." Journal Of Toxicology And Environmental Health-Part B-Critical ReviewS 4(1): 1-143.	Type of study not relevant or study not EMF/health-related
Krylova, I. N., et al. (1992). "Effect of microwave radiation on learning and memory." Bulletin Of Experimental Biology And Medicine 114(5): 1620–1622.	Frequency not relevant, electric current application, co-exposure or exposure condition unclear
Kryukova, O. V., et al. (2016). "Effect of electromagnetic microwave radiation on the growth of Ehrlich ascites carcinoma." Doklady Biological Sciences 470(1): 237-239.	Frequency not relevant, electric current application, co-exposure or exposure condition unclear
Kücer, N. And T. Pamukcu (2014). "Self-reported symptoms associated with exposure to electromagnetic fields: a questionnaire study." Electromagnetic Biology and Medicine 33(1): 15-17.	Frequency not relevant, electric current application, co-exposure or exposure condition unclear
Kues, H. A. And J. C. Monahan (1992). "Microwave-induced changes to the primate eye." John Hopkins APL Technical Digest 13(1): 244-255.	Frequency not relevant, electric current application, co-exposure or exposure condition unclear
Kumar, R. S., et al. (2009). "Hypoactivity of Wistar rats exposed to mobile phone on elevated plus maze." Indian Journal of Physiology and Pharmacology 53(3): 283-286.	Frequency not relevant, electric current application, co-exposure or exposure condition unclear

Kumar, S., et al. (2014). "Effect of electromagnetic irradiation produced by 3G mobile phone on male rat reproductive system in a simulated scenario." Indian Journal of Experimental Biology 52(9): 890-897.	Frequency not relevant, electric current application, co-exposure or exposure condition unclear
Kuzay, D., et al. (2017). "Oxidative effects of extremely low frequency magnetic field and radio frequency radiation on testes tissues of diabetic and healthy rats." Bratislavské Lekárske Listy 118(5): 278-282.	Frequency not relevant, electric current application, co-exposure or exposure condition unclear
Kwak, H. W., et al. (2013). "Postoperative effect of radiofrequency treatments on the rabbit dermal collagen fibrillary matrix." Microscopy Research and Technique 76(3): 219-224.	Frequency not relevant, electric current application, co-exposure or exposure condition unclear
La Vecchia, C., et al. (1990). "Occupation and the risk of bladder cancer." Int J Epidemiol 19(2): 264-268.	Frequency not relevant, electric current application, co-exposure or exposure condition unclear
Lagorio, S., et al. (1997). "Mortality of plastic-ware workers exposed to radiofrequencies." Bioelectromagnetics 18(6): 418-421.	Frequency not relevant, electric current application, co-exposure or exposure condition unclear
Lai, H., et al. (1984). "Microwave-induced post-exposure hyperthermia: Involvement of endogenous opioids and Serotonin." IEEE Transactions On Microwave Theory And Techniques 32(8): 882-887.	Frequency not relevant, electric current application, co-exposure or exposure condition unclear
Lai, H., et al. (1989). "Low-level microwave irradiation and central cholinergic systems." Pharmacology, Biochemistry and Behavior 33(1): 131-138.	Frequency not relevant, electric current application, co-exposure or exposure condition unclear
Lai, Y., et al. (2023). "Hippocampal ferroptosis is involved in learning and memory impairment in rats induced by microwave and electromagnetic pulse combined exposure." Environmental Science and Pollution Research 30(35): 83717-83727.	Frequency not relevant, electric current application, co-exposure or exposure condition unclear
Lalic, H., A. Lekic and B. Radosevic-Stasic (2001). "Comparison of chromosome aberrations in peripheral blood lymphocytes from people occupationally exposed to ionizing and radiofrequency radiation." Acta Medica Okayama 55(2): 117-127.	Frequency not relevant, electric current application, co-exposure or exposure condition unclear
Lancranjan, I., et al. (1975). "Gonadic function in workmen with long-term exposure to microwaves." Health Physics 29(3): 381-383.	Frequency not relevant, electric current application, co-exposure or exposure condition unclear

Lange, D. G., L. R. Mckenzie and A. M. Phelan (1993). "Alteration In Atpase Activity In Rat-Brain Homogenates Exposed To Low-Level Microwave-Radiation." Faseb Journal 7(3): A272-A272.	Type of study not relevant or study not EMF/health-related
Laroche, L. P., M. M. Zaret and A. F. Braun (1970). "An operational safety program for ophthalmic hazards of microwave." Archives of Environmental Health 20(3): 350-355.	Type of study not relevant or study not EMF/health-related
Larsen, A. I. (1991). "Congenital malformations and exposure to high-frequency electromagnetic radiation among Danish physiotherapists." Scandinavian Journal of Work, Environment & Health 17(5): 318-323.	Frequency not relevant, electric current application, co-exposure or exposure condition unclear
Larsen, A. I., J. Olsen and O. Svane (1991). "Gender-specific reproductive outcome and exposure to high-frequency electromagnetic radiation among physiotherapists." Scandinavian Journal of Work, Environment & Health 17(5): 324-329.	Frequency not relevant, electric current application, co-exposure or exposure condition unclear
Lebovitz, R. M. (1983). "Pulse modulated and continuous wave microwave radiation yield equivalent changes in operant behavior of rodents." Physiology & Behavior 30(6): 891-898.	Frequency not relevant, electric current application, co-exposure or exposure condition unclear
Lebovitz, R. M. (1983). "Pulse-Modulated And Continuous Wave Microwave-Radiation Yield Equivalent Changes In Operant-Behavior Of Rodents." Physiology & Behavior 30(6): 891-898.	Frequency not relevant, electric current application, co-exposure or exposure condition unclear
Lebovitz, R. M. And R. L. Seaman (1977). "Microwave hearing: The response of single auditory neurons in the cat pulsed microwave radiation." Radio Science 12: 229-236.	Frequency not relevant, electric current application, co-exposure or exposure condition unclear
Lebovitz, R. M. And R. L. Seaman (1980). "Microwave irradiation and instrumental behavior in rats: unitized irradiation and behavioral evaluation facility." Bioelectromagnetics 1(4): 415-428.	Frequency not relevant, electric current application, co-exposure or exposure condition unclear
Lee, C. M., et al. (2012). "Acute Effects of 835 mhz Radiofrequency Radiation Exposure to Mice Hippocampus and Cerebellum." Neurology 78.	Frequency not relevant, electric current application, co-exposure or exposure condition unclear
Lee, S. A. And H. G. Cha (2023). "The Effects of Microwave Diathermy on Pain and Function in Chronic Low Back Pain Patients." Iranian Journal of Public Health 52(1): 205-207.	Frequency not relevant, electric current application, co-exposure or exposure condition unclear

Lehmann, J. F., et al. (1979). "Microwave therapy: stray radiation, safety and effectiveness." Archives of Physical Medicine and Rehabilitation 60(12): 578-584.	Type of study not relevant or study not EMF/health-related
Lester, J. R. And D. F. Moore (1982). "Cancer incidence and electromagnetic radiation." Journal of Bioelectricity 1(1): 59-76.	Frequency not relevant, electric current application, co-exposure or exposure condition unclear
Lester, J. R. And D. F. Moore (1982). "Cancer mortality and air force bases." Journal of Bioelectricity 1(1): 77-82.	Frequency not relevant, electric current application, co-exposure or exposure condition unclear
Leszczynski, D. (2013). Effects of Radiofrequency-Modulated Electromagnetic Fields on Proteome. 990: 101-106.	Type of study not relevant or study not EMF/health-related
Li, B. F., et al. (2007). "Electromagnetic pulses induce fluctuations in blood pressure in rats." International Journal Of Radiation Biology 83(6): 421-429.	Frequency not relevant, electric current application, co-exposure or exposure condition unclear
Li, D. Y., et al. (2015). "Effect of pulsed radiofrequency on rat sciatic nerve chronic constriction injury: a preliminary study." Chinese Medical Journal 128(4): 540-544.	Frequency not relevant, electric current application, co-exposure or exposure condition unclear
Li, D., et al. (2022). "Hsp72-Based Effect and Mechanism of Microwave Radiation-Induced Cardiac Injury in Rats." Oxidative Medicine and Cellular Longevity 2022: 7145415.	Frequency not relevant, electric current application, co-exposure or exposure condition unclear
Li, D., et al. (2023). "Physiological and Psychological Stress of Microwave Radiation-Induced Cardiac Injury in Rats." International Journal of Molecular Sciences 24(7): 6237.	Frequency not relevant, electric current application, co-exposure or exposure condition unclear
Li, L., et al. (2021). "Effects of Ultrashort Wave Therapy on Inflammation and Macrophage Polarization after Acute Lung Injury in Rats." Bioelectromagnetics 42(6): 464-472.	Frequency not relevant, electric current application, co-exposure or exposure condition unclear
Li, Q., et al. (2011). "Pulsed Radio Frequency Energy Accelerates Wound Healing In Diabetic Mice." Plastic & Reconstructive Surgery 127(6): 2255-2262.	Frequency not relevant, electric current application, co-exposure or exposure condition unclear
Li, Y. Z., et al. (2014). "Microwave Radiation Injures Microvasculature Through Inducing Endoplasmic Reticulum Stress." Microcirculation 21(6): 490-498.	Frequency not relevant, electric current application, co-exposure or exposure condition unclear

Li, Y., et al. (2014). "Microwave radiation injuries microvasculature through inducing endoplasmic reticulum stress." Microcirculation 21(6): 490-498.	Frequency not relevant, electric current application, co-exposure or exposure condition unclear
Liburdy, R. P. (1979). "Radiofrequency radiation alters the immune system: modulation of T- and B-lymphocyte levels and cell-mediated immunocompetence by hyperthermic radiation." Radiation Research 77(1): 34-46.	Frequency not relevant, electric current application, co-exposure or exposure condition unclear
Limanskii, Y. P., et al. (1999). "Suppression of nociceptive reactions in mice under low-intensity microwave irradiation of acupuncture points." Neurophysiology 31(4): 263-267.	Frequency not relevant, electric current application, co-exposure or exposure condition unclear
Lin, J. C. And M. F. Lin (1982). "Microwave hyperthermia-induced blood-brain barrier alterations." Radiation Research 89(1): 77-87.	Frequency not relevant, electric current application, co-exposure or exposure condition unclear
Lin, J. C., P. M. K. Yuan and D. T. Jung (1998). "Enhancement of Anticancer Drug Delivery to the Brain by Microwave Induced Hyperthermia." Bioelectrochemistry And Bioenergetics 47(2): 259-264.	Frequency not relevant, electric current application, co-exposure or exposure condition unclear
Lin, J. C., R. J. Meltzer and F. K. Redding (1979). "Microwave-evoked brainstem potentials in cats." The Journal of Microwave Power 14(3): 291-296.	Frequency not relevant, electric current application, co-exposure or exposure condition unclear
Lisiewicz, J. (1993). "Immunotoxic and hematotoxic effects of occupational exposures." Folia Med Cracov 34(1-4): 29-47.	Type of study not relevant or study not EMF/health-related
Liu, H. F., et al. (2011). "Improvement of clinical parameters in patients with gastroesophageal reflux disease after radiofrequency energy delivery." World Journal of Gastroenterology 17(39): 4429-4433.	Frequency not relevant, electric current application, co-exposure or exposure condition unclear
Liu, N. A., et al. (2008). "Establishment of Imaging mass Spectrometry of biological tissues and its application on the Proteome analysis of microwave radiated rat hippocampus." Chinese Journal Of Analytical Chemistry 36(4): 421-425.	Frequency not relevant, electric current application, co-exposure or exposure condition unclear
Liu, Y. H., et al. (1988). "Long-Term Effects on Human Spermatogenesis after Microwave Treatment of Testes." Journal of Bioelectricity 7(1): 97-102.	Frequency not relevant, electric current application, co-exposure or exposure condition unclear

Liu, Y. Q., et al. (2015). "Dynamic Expression of Hyperpolarization-activated Cyclic Nucleotide-gated Cation Channel 4 Involved in Microwave Induced Pacemaker Cell Injuries." Biomedical and Environmental Sciences 28(11): 823-828.	Frequency not relevant, electric current application, co-exposure or exposure condition unclear
Liu, Y. Q., et al. (2015). "Pathological changes in the sinoatrial node tissues of rats caused by pulsed microwave exposure." Biomedical and Environmental Sciences 28(1): 72-75.	Frequency not relevant, electric current application, co-exposure or exposure condition unclear
Liu, Y., et al. (2010). “Effects of Microwave Irradiation on Translocation and Activation of Protein Kinase C in Rabbit Cerebellum.” Proceedings Of The 5th International Academic Conference on Environmental And Occupational Medicine 2010:264-266	Not available
Logani, M. K., et al. (2006). "Effect of millimeter waves on cyclophosphamide induced NF-kappab." Electromagnetic Biology and Medicine 25(1): 23-27.	Frequency not relevant, electric current application, co-exposure or exposure condition unclear
Logue, J. N., et al. (1985). "Congenital anomalies and paternal occupational exposure to shortwave, microwave, infrared, and acoustic radiation." Journal of Occupational Medicine 27(6): 451-452.	Frequency not relevant, electric current application, co-exposure or exposure condition unclear
Lojek, A., J. Cesnek and J. Misustova (1987). "The effect of microwave radiation on the primary igm response to sheep red blood cells in mice." Folia Biologica 33(3): 200-206.	Frequency not relevant, electric current application, co-exposure or exposure condition unclear
Lojek, A., J. Cesnek and J. Misustova (1987). "The Effect of Microwave-Radiation On The Primary Igm Response To Sheep Red-Blood-Cells In Mice." Folia Biologica 33(3): 200-206.	Frequency not relevant, electric current application, co-exposure or exposure condition unclear
López, I., et al. (2021). "What is the radiation before 5G?: a correlation study between measurements in situ and in real time and epidemiological indicators in Vallecas, Madrid." Environmental Research 194: 110734.	Frequency not relevant, electric current application, co-exposure or exposure condition unclear
Lotz, W. G. And S. M. Michaelson (1979). "Effects of hypophysectomy and dexamethasone on rat adrenal response to microwaves." Journal of Applied Physiology: Respiratory, Environmental and Exercise Physiology 47(6): 1284-1288.	Frequency not relevant, electric current application, co-exposure or exposure condition unclear
Lu, S. T., et al. (1999). "Ultrawide-band electromagnetic pulses induced hypotension in rats." Physiol Behav 65(4-5): 753-761.	Frequency not relevant, electric current application, co-exposure or exposure condition unclear

Luleci, N., et al. (2011). "Evaluation of patients' response to pulsed radiofrequency treatment applied to the suprascapular nerve in patients with chronic shoulder pain." Journal of Back and Musculoskeletal Rehabilitation 24(3): 189-194.	Frequency not relevant, electric current application, co-exposure or exposure condition unclear
Luo, F., et al. (2013). "Pulsed radiofrequency treatment for idiopathic trigeminal neuralgia: a retrospective analysis of the causes for ineffective pain relief." Eur J Pain 17(8): 1189-1192.	Frequency not relevant, electric current application, co-exposure or exposure condition unclear
Luo, Q., et al. (2009). "Pulse electromagnetic fields effects on serum E2 levels, chondrocyte apoptosis, and matrix metalloproteinase-13 expression in ovariectomized rats." Rheumatology International 29(8): 927-935.	Frequency not relevant, electric current application, co-exposure or exposure condition unclear
Luo, X., et al. (2021). "Electromagnetic field exposure-induced depression features could be alleviated by heat acclimation based on remodeling the gut microbiota." Ecotoxicology and Environmental Safety 228: 112980.	Frequency not relevant, electric current application, co-exposure or exposure condition unclear
Lv, B., et al. (2014). "The alteration of spontaneous low frequency oscillations caused by acute electromagnetic fields exposure." Clinical Neurophysiology 125(2): 277-286.	Frequency not relevant, electric current application, co-exposure or exposure condition unclear
Lv, B., et al. (2014). Whole brain EEG synchronization likelihood modulated by long term evolution electromagnetic fields exposure, IEEE: 986-989.	Frequency not relevant, electric current application, co-exposure or exposure condition unclear
Lyaginskaja, A. M., et al. (2010). "Autoimmune processes after long-term low-level exposure to electromagnetic fields (experimental results) part 5. Study of the influence of blood serum from rats exposed to low-level electromagnetic fields on pregnancy and fetal and offspring development." Biophysics 55(6): 1059-1066.	Frequency not relevant, electric current application, co-exposure or exposure condition unclear
Lyusov, V. A., et al. (2000). "Effects of electromagnetic radiation of the millimeter wavelength range on catecholamine metabolism in patients with arterial hypertension." Crit Rev Biomed Eng 28(5-6): 1-10.	Frequency not relevant, electric current application, co-exposure or exposure condition unclear
Ma, L., et al. (2023). "Transcriptomic and metabolomic studies on the protective effect of molecular hydrogen against nuclear electromagnetic pulse-induced brain damage." Frontiers in Public Health 11: 1103022.	Frequency not relevant, electric current application, co-exposure or exposure condition unclear

Maatman, R. C., et al. (2019). "A Randomized Controlled Trial to Evaluate the Effect of Pulsed Radiofrequency as a Treatment for Anterior Cutaneous Nerve Entrapment Syndrome in Comparison to Anterior Neurectomy." Pain Pract 19(7): 751-761.	Frequency not relevant, electric current application, co-exposure or exposure condition unclear
Maeda, K., T. Maeda and Y. Qi (2004). "In vitro and in vivo induction of human lovo cells into apoptotic process by non-invasive microwave treatment: a potentially novel approach for physical therapy of human colorectal cancer." Oncology Reports 11(4): 771-775.	Frequency not relevant, electric current application, co-exposure or exposure condition unclear
Magras, I. N. And T. D. Xenos (1997). "RF radiation-induced changes in the prenatal development of mice." Bioelectromagnetics 18(6): 455-461.	Frequency not relevant, electric current application, co-exposure or exposure condition unclear
Magro, V. I., et al. (2005). "Influencing of electromagnetic radiation on some immunological indexes of organism." 2005 5th International Conference on Antenna Theory and Techniques, Kyiv, Ukraine. IEEE, 2005: 488-490.	Frequency not relevant, electric current application, co-exposure or exposure condition unclear
Mahajan, R., D. Bansal and A. Khatter (2019). "EEG Based Cognitive Brain Mapping in Time Domain to Analyze EM Radiation Effect on Human Brain." Advanced Informatics for Computing Research, ICAICR 2018, Pt I. Communications in Computer and Information Science, Band 955; Springer, Singapore, 2019; 308-319	Frequency not relevant, electric current application, co-exposure or exposure condition unclear
Mahila (2021). "Effect of Wi-Fi Radiation on Heart Rate Variability, Salivary Cortisol Level and Cognition." Journal of Pharmaceutical Research International 33(41B): 229-232.	Frequency not relevant, electric current application, co-exposure or exposure condition unclear
Mahmood, M. N., A. H. Shaker and H. E. Mohammed (2022). "Estimation of some antioxidants in people exposed to electromagnetic waves from Internet towers in Samarra." Journal of Population Therapeutics and Clinical Pharmacology 29(2): E79-E87.	Frequency not relevant, electric current application, co-exposure or exposure condition unclear
Mahmoud, A. A., R. D. Mcafee and R. R. Bishop (1989). "Biological Effects On Swine And Primates Due To Power Frequency And Radio-Frequency Radiation." Electric Power Systems Research 17(1): 29-34.	Type of study not relevant or study not EMF/health-related

Mahmoud, N. M., R. S. Gomaa and A. E. Salem (2021). "Activation of liver X receptors ameliorates alterations in testicular function in rats exposed to electromagnetic radiation." Alexandria Journal of Medicine 57(1): 82-91.	Frequency not relevant, electric current application, co-exposure or exposure condition unclear
Majewska, K. (1968). "Investigations on the effect of microwaves on the eye." Polish Medical Journal 7(4): 989-994.	Frequency not relevant, electric current application, co-exposure or exposure condition unclear
Manikowskaczerska, E., P. Czerski and W. M. Leach (1982). "Microwave-Radiation Exposure Induced Translocations And Univalents During Spermatogenesis In Mice." Journal Of Cell Biology 95(2): A301-A301.	Type of study not relevant or study not EMF/health-related
Mannu, P., et al. (2011). "Noninvasive brain stimulation by radioelectric asymmetric conveyor in the treatment of agoraphobia: open-label, naturalistic study." Patient Preference and Adherence 5: 575-580.	No control group/condition
Mannu, P., et al. (2011). "Radio electric asymmetric brain stimulation in the treatment of behavioral and psychiatric symptoms in Alzheimer disease." Clinical Interventions in Aging 6: 207-211.	No control group/condition
Marcickiewicz, J., et al. (1986). "Microwave-Radiation Enhances Teratogenic Effect Of Cytosine-Arabinoside In Mice." Biology Of The Neonate 50(2): 75-82.	Frequency not relevant, electric current application, co-exposure or exposure condition unclear
Marino, A. A. (1995). "Time-dependent hematological changes in workers exposed to electromagnetic fields." American Industrial Hygiene Association Journal 56(2): 189-192.	Frequency not relevant, electric current application, co-exposure or exposure condition unclear
Marks, J., et al. (1961). "Microwave radiation to the anterior mediastinum of the dog. Histologic and electrocardiographic observations." Ohio State Med J 57: 274-279.	Frequency not relevant, electric current application, co-exposure or exposure condition unclear
Marks, J., et al. (1961). "Microwave radiation to the anterior mediastinum of the dog. II. Thermal, cardiovascular, respiratory and blood enzyme observations." Ohio State Med J 57: 1132-1135.	Frequency not relevant, electric current application, co-exposure or exposure condition unclear
Marks, J., et al. (1961). "Microwave radiation to the anterior mediastinum of the dog. II. Thermal, cardiovascular, respiratory and blood enzyme observations." Ohio State Medical Journal 57(10): 1132-1135.	Frequency not relevant, electric current application, co-exposure or exposure condition unclear

Marr, M. J., et al. (1988). "Microwaves as reinforcing events in a cold environment." Progress in Clinical and Biological Research 257: 219-234.	Type of study not relevant or study not EMF/health-related
Marshall, T. G. And T. J. R. Heil (2017). "Electrosmog and autoimmune disease." Immunologic Research 65(1): 129-135.	Frequency not relevant, electric current application, co-exposure or exposure condition unclear
Martens, A. L., et al. (2018). "Modeled and perceived RF-EMF, noise and air pollution and symptoms in a population cohort. Is perception key in predicting symptoms?" Science of the Total Evironment 639: 75-83.	Frequency not relevant, electric current application, co-exposure or exposure condition unclear
Maruyama, Y., R. Nakamura and K. Kobayashi (1980). "Effect of microwave irradiation on brain tissue structure and catecholamine distribution." Psychopharmacology (Berl) 67(2): 119-123.	Frequency not relevant, electric current application, co-exposure or exposure condition unclear
Maskey, D., et al. (2013). "Neuroprotective effect of ginseng against alteration of calcium binding proteins immunoreactivity in the mice hippocampus after radiofrequency exposure." biomed Research International: 812641.	Frequency not relevant, electric current application, co-exposure or exposure condition unclear
Mason, P. A., et al. (1997). "Amino acid concentrations in hypothalamic and caudate nuclei during microwave-induced thermal stress: analysis by microdialysis." Bioelectromagnetics 18(3): 277-283.	No control group/condition
Matrenina, V. L., V. M. Posadskaya and I. A. Rudakov (1978). "Temperature Reaction and Survival Of Experimental-Animals After Microwave Irradiation." Bulletin Of Experimental Biology and Medicine 86(9): 1146-1148.	Frequency not relevant, electric current application, co-exposure or exposure condition unclear
Matrenina, V. L., V. M. Posadskaya and I. A. Rudakov (1978). "Temperature Reaction and Survival of Experimental-Animals After Microwave Irradiation." Bulletin Of Experimental Biology and Medicine 86(9): 1146-1148.	Frequency not relevant, electric current application, co-exposure or exposure condition unclear
Matveev, A. G. (2001). "Application of millimeter waves in treating chronic prostatitis patients." Critical Reviews in Biomedical Engineering 29(3): 482-492.	Frequency not relevant, electric current application, co-exposure or exposure condition unclear
Mayrovitz, H. N. And P. B. Larsen (1995). "A Preliminary Study to Evaluate the Effect of Pulsed Radio Frequency Field Treatment on Lower Extremity Peri-Ulcer Skin Microcirculation of Diabetic Patients." Wounds 7(3): 90-93.	Frequency not relevant, electric current application, co-exposure or exposure condition unclear

Mayrovitz, H. N. And P. B. Larsen (1995). "A Preliminary-Study To Evaluate The Effect Of Pulsed Radio-Frequency Field Treatment On Lower-Extremity Peri-Ulcer Skin Microcirculation Of Diabetic-Patients." Wounds-A Compendium Of Clinical Research And Practice 7(3): 90-93.	Frequency not relevant, electric current application, co-exposure or exposure condition unclear
Mcafee, R. D. (1962). "Physiological Effects of Thermode and Microwave Stimulation of Peripheral Nerves." American Journal of Physiology 203(2): 374-378.	RF EMF used to heat (> 42°C) and/or destroy tissue
Mcafee, R. D. (1971). "Analeptic Effect Of Microwave Irradiation On Experimental Animals." Ieee Transactions On Microwave Theory And Techniques MT 19(2): 251.	Type of study not relevant or study not EMF/health-related
Mcafee, R. D., et al. (1979). "Absence of ocular pathology after repeated exposure of unanesthetized monkeys to 9.3-ghz microwaves." The Journal of Microwave Power 14(1): 41-44.	No control group/condition
Mcafee, R. D., et al. (1983). "Absence of deleterious effects of chronic microwave radiation on the eyes of rhesus monkeys." Ophthalmology 90(10): 1243-1245.	No control group/condition
Mcmeeken, J. M. And C. Bell (1990). "Effects of microwave irradiation on blood flow in the dog hindlimb." Exp Physiol 75(3): 367-374.	Frequency not relevant, electric current application, co-exposure or exposure condition unclear
Mcree, D. I., M. J. Galvin and C. L. Mitchell (1988). "Microwave effects on the cardiovascular system: a model for studying the responsivity of the autonomic nervous system to microwaves." Progress in Clinical and Biological Research 257: 153-177.	Frequency not relevant, electric current application, co-exposure or exposure condition unclear
Meister, A., S. Eggert and S. Goltz (1990). "[The perceptibility of a microwave field under experimental conditions]." Z Gesamte Hyg 36(3): 131-135.	Frequency not relevant, electric current application, co-exposure or exposure condition unclear
Meister, A., S. Eggert and S. Goltz (1990). "Die Wahrnehmung eines Mikrowellen-Feldes unter experimentellen Bedingungen." Zeitschrift für die gesamte Hygiene und ihre Grenzgebiete 36(3): 131-135.	Frequency not relevant, electric current application, co-exposure or exposure condition unclear
Menendez, M., et al. (2010). "Radiofrequency energy on cortical bone and soft tissue: a pilot study." Clinical Orthopaedics and Related Research 468(4): 1157-1164.	Frequency not relevant, electric current application, co-exposure or exposure condition unclear

Menini, M., et al. (2016). "Effects of pulsed electromagnetic fields on swelling and pain after implant surgery: a double-blind, randomized study." International Journal of Oral and Maxillofacial Surgery 45(3): 346-353.	Frequency not relevant, electric current application, co-exposure or exposure condition unclear
Meric, F., S. Dasdag and K. Vergili (1998). "Do radiofrequency radiation affect the auditory system of of people with occupational exposure?" Environmental Health and Preventive Medicine 3(1): 55-58.	Frequency not relevant, electric current application, co-exposure or exposure condition unclear
Merritt, J. H., et al. (1977). "Orientation effects on microwave-induced hyperthermia and neurochemical correlates." The Journal of Microwave Power 12(2): 167-172.	Frequency not relevant, electric current application, co-exposure or exposure condition unclear
Michaelson, S. M., et al. (1964). "The Hematologic Effects of Microwave Exposure." Aerosp Med 35: 824-829.	Frequency not relevant, electric current application, co-exposure or exposure condition unclear
Michaelson, S. M., J. W. Howland and W. B. Deichmann (1971). "Response of the dog to 24,000 and 1285 mhz microwave exposure." IMS Ind Med Surg 40(5): 18-23.	No control group/condition
Mickley, G. A., et al. (1994). "Disruption of a putative working memory task and selective expression of brain c-fos following microwave-induced hyperthermia." Physiology & Behavior 55(6): 1029-1038.	Frequency not relevant, electric current application, co-exposure or exposure condition unclear
Mikami, T. And H. Yamauchi (2022). "Preconditioning with whole-body or regional hyperthermia attenuates exercise-induced muscle damage in rodents." Physiol Res 71(1): 125-134.	Frequency not relevant, electric current application, co-exposure or exposure condition unclear
Milham Jr, S. (1988). "Increased mortality in amateur radio operators due to lymphatic and hematopoietic malignancies." American Journal of Epidemiology 127(1): 50-54.	Frequency not relevant, electric current application, co-exposure or exposure condition unclear
Milham, S. (2004). "Epidemiological studies of radio frequency exposures and human cancer." Bioelectromagnetics 25(4): 235	Type of study not relevant or study not EMF/health-related
Miller, D. B., C. F. Blackman and J. P. O'Callaghan (1987). "An increase in glial fibrillary acidic protein follows brain hyperthermia in rats." Brain Research 415(2): 371-374.	Frequency not relevant, electric current application, co-exposure or exposure condition unclear

Miller, S. A., M. E. Bronson and M. R. Murphy (1999). "Ultrawideband radiation and pentylenetetrazol-induced convulsions in rats." Bioelectromagnetics 20(5): 327-329.	Frequency not relevant, electric current application, co-exposure or exposure condition unclear
Milroy, W. C. And S. M. Michaelson (1972). "Thyroid pathophysiology of microwave radiation." Aerospace Medicine 43(10): 1126-1131.	Frequency not relevant, electric current application, co-exposure or exposure condition unclear
Minasyan, S. M., et al. (2007). "Effects of the action of microwave-frequency electromagnetic radiation on the spike activity of neurons in the supraoptic nucleus of the hypothalamus in rats." Neuroscience and Behavioral Physiology 37(2): 175-180.	Frequency not relevant, electric current application, co-exposure or exposure condition unclear
Mjoen, G., et al. (2006). "Paternal occupational exposure to radiofrequency electromagnetic fields and risk of adverse pregnancy outcome." European Journal of Epidemiology 21(7): 529-535.	Frequency not relevant, electric current application, co-exposure or exposure condition unclear
Modak, A. T., et al. (1976). "Use Of 300-Msec Microwave Irradiation for Enzyme Inactivation - Study of Effects of Sodium Pentobarbital on Acetylcholine Concentration in Mouse-Brain Regions." Journal Of Pharmacology and Experimental Therapeutics 197(2): 245-252.	Frequency not relevant, electric current application, co-exposure or exposure condition unclear
Mohamed, A. O., et al. (2023). "Exercise ameliorates hippocampal damage induced by Wi-Fi radiation ; a biochemical, histological, and immunohistochemical study." J Chem Neuroanat 2023; 129: 102252	Frequency not relevant, electric current application, co-exposure or exposure condition unclear
Mohammadianinejad, S. E., M. Babaei and P. Nazari (2016). "The Effects of Exposure to Low Frequency Electromagnetic Fields in the Treatment of Migraine Headache: A Cohort Study." Electronic Physician 8(12): 3445-3449.	Frequency not relevant, electric current application, co-exposure or exposure condition unclear
Mohler, E., et al. (2012). "Exposure to Radiofrequency Electromagnetic Fields and Sleep Quality: A Prospective Cohort Study." plos One 7(5): e37455.	Frequency not relevant, electric current application, co-exposure or exposure condition unclear
Mollerlokken, O. J. And B. E. Moen (2008). "Is fertility reduced among men exposed to radiofrequency fields in the Norwegian Navy?" Bioelectromagnetics 29(5): 345-352.	Frequency not relevant, electric current application, co-exposure or exposure condition unclear
Monahan, J. C. (1988). "Microwave--drug interactions in the cholinergic nervous system of the mouse."	Frequency not relevant, electric current application, co-exposure or exposure condition unclear

Progress in Clinical and Biological Research 257: 309-326.	
Monahan, J. C. And H. S. Ho (1977). "The effect of ambient temperature on the reduction of microwave energy absorption by mice." Radio Science 12: 257-262.	Frequency not relevant, electric current application, co-exposure or exposure condition unclear
Monahan, J. C. And W. W. Henton (1979). "The effect of psychoactive drugs on operant behavior induced by microwave radiation." Radio Science 14(6): 233-238.	Frequency not relevant, electric current application, co-exposure or exposure condition unclear
Moradpour, R., et al. (2020). "The protective effect of melatonin on liver damage induced by mobile phone radiation in mice model." International Journal of Radiation Research 18(1): 133-141.	Frequency not relevant, electric current application, co-exposure or exposure condition unclear
Mori, T. And G. W. Arendash (2011). "Long-term Electromagnetic Field Treatment Increases Brain Neuronal Activity: Linkage to Cognitive Benefit and Therapeutic Implications for Alzheimer's Disease." Journal of Alzheimer's Disease & Parkinsonism 1(2): 1000102.	Frequency not relevant, electric current application, co-exposure or exposure condition unclear
Mortazavi, S. A., et al. (2016). "The Fundamental Reasons Why Laptop Computers should not be Used on Your Lap." J Biomed Phys Eng 6(4): 279-284.	Type of study not relevant or study not EMF/health-related
Mortazavi, S. M. J., S. Taeb and N. Dehghan (2013). "Alterations of visual reaction time and short term memory in military radar personnel." Iranian Journal of Public Health 42(4): 428-435.	Frequency not relevant, electric current application, co-exposure or exposure condition unclear
Mortazavi, S. M. J., S.A.R. Mortazavi and M. Haghani (2019). "Evaluation of the Validity of a Nonlinear J-Shaped Dose-Response Relationship in Cancers Induced by Exposure to Radiofrequency Electromagnetic Fields." J Biomed Phys Eng 9(4): 487-494.	Type of study not relevant or study not EMF/health-related
Mortazavi, S. M., J. Ahmadi and M. Shariati (2007). "Prevalence of subjective poor health symptoms associated with exposure to electromagnetic fields among university students." Bioelectromagnetics 28(4): 326-330.	Frequency not relevant, electric current application, co-exposure or exposure condition unclear

Mortazavi, S. M., K. R. Shirazi and G. Mortazavi (2013). "The study of the effects of ionizing and non-ionizing radiations on birth weight of newborns to exposed mothers." Journal of Natural Science, Biology, and Medicine 4(1): 213-217.	Frequency not relevant, electric current application, co-exposure or exposure condition unclear
Mortazavi, S. M., M. Atefi and F. Kholghi (2011). "The pattern of mobile phone use and prevalence of self-reported symptoms in elementary and junior high school students in Shiraz, Iran." Iranian Journal of Medical Sciences 36(2): 96-103.	Frequency not relevant, electric current application, co-exposure or exposure condition unclear
Motamedi, M. H., et al. (2009). "Trigeminal neuralgia and radiofrequency." Journal of the California Dental Association 37(2): 109-114.	Frequency not relevant, electric current application, co-exposure or exposure condition unclear
Motzkin, S. M., et al. (1983). Effects of Low-level Millimeter Waves on Cellular and Subcellular Systems, Springer: 47-57.	Non-mammal/in vitro study
Movahedi, M. M., et al. (2017). "Short-term exposure to mobile jammer radiofrequency radiation adversely affects the human hearing." Biomedical Research-India 28(4): 1557-1559.	Frequency not relevant, electric current application, co-exposure or exposure condition unclear
Muhm, J. M. (1992). "Mortality investigation of workers in an electromagnetic pulse test program." Journal of Occupational Medicine 34(3): 287-292.	Frequency not relevant, electric current application, co-exposure or exposure condition unclear
Murphy, A. J., W. D. Paul and H. M. Hines (1950). "A comparative study of the temperature changes produced by various thermogenic agents." Archives of Physical Medicine 31(3): 151-156.	No control group/condition
Murphy, M. R. (1995). "Extrapolation Of Animal Radiofrequency Radiation (RRF) Bioeffects to Humans." Radiofrequency Radiation Standards. NATO ASI Series, Band 274; Springer, Boston, MA, 1995; 303-307	Type of study not relevant or study not EMF/health-related
Murphy, M. R. (2000). United States air force support of radio frequency radiation health and safety: Bioeffects, dosimetry, and standards. 82: 11-19.	Type of study not relevant or study not EMF/health-related
Nageswari, K. S., et al. (1982). "Effect of chronic microwave radiation on rabbit erythrocytes." Indian J Exp Biol 20(1): 13-15.	Frequency not relevant, electric current application, co-exposure or exposure condition unclear
Nageswari, K. S., et al. (1982). "Effect of prolonged low power microwave radiation on peripheral leucocyte counts in rabbits." Indian J Med Res 75: 453-459.	Frequency not relevant, electric current application, co-exposure or exposure condition unclear

Nakatani-Enomoto, S., et al. (2020). "Effects of electromagnetic fields from long-term evolution on awake electroencephalogram in healthy humans." Neuroscience Research 156: 102-107.	Frequency not relevant, electric current application, co-exposure or exposure condition unclear
Nakra, A. And M. Duhan (2015). "Reduction of electromagnetic radiation from GSM mobile phone on human brain by the use of Bluetooth headset and earphone." International Journal of Biomedical Engineering and Technology 17(1): 42-54.	Frequency not relevant, electric current application, co-exposure or exposure condition unclear
Nassar, S., et al. (2020). "The efficacy of radiofrequency, intense pulsed light and carboxytherapy in facial rejuvenation." J Cosmet Laser Ther 22(6-8): 256-264.	Frequency not relevant, electric current application, co-exposure or exposure condition unclear
Nasser, M., et al. (2018). "Dataset on significant role of Candesartan on cognitive functions in rats having memory impairment induced by electromagnetic waves." Data in Brief 21: 2390-2394.	Frequency not relevant, electric current application, co-exposure or exposure condition unclear
Nayak, S., et al. (2022). "Effect of Radiofrequency Waves of Mobile Phones on Distortion Product Otoacoustic Emissions." Journal of Health and Allied Sciences NU 12(2): 119-125.	Frequency not relevant, electric current application, co-exposure or exposure condition unclear
Neilly, J. P. And J. C. Lin (1986). "Interaction of ethanol and microwaves on the blood-brain barrier of rats." Bioelectromagnetics 7(4): 405-414.	RF EMF used to heat (> 42°C) and/or destroy tissue
Nelson, B. K., et al. (1991). "Marked increase in the teratogenicity of the combined administration of the industrial solvent 2-methoxyethanol and radiofrequency radiation in rats." Teratology 43(6): 621-634.	Frequency not relevant, electric current application, co-exposure or exposure condition unclear
Nelson, B. K., et al. (1997). "Interactions of radiofrequency radiation-induced hyperthermia and 2-methoxyethanol teratogenicity in rats." Bioelectromagnetics 18(5): 349-359.	Frequency not relevant, electric current application, co-exposure or exposure condition unclear
Nesin, E. (2006). "Suppression of Synthesis of Serotonin: Effect on Antinociception Induced by Microwave Irradiation and Analgesic Remedies." Neurophysiology 38(5-6): 427-428.	Frequency not relevant, electric current application, co-exposure or exposure condition unclear
Newton, J. M., et al. (2018). "Non-Invasive Radiofrequency Field Treatment of 4T1 Breast Tumors Induces T-cell Dependent Inflammatory Response." Sci Rep 8(1): 3474.	Frequency not relevant, electric current application, co-exposure or exposure condition unclear

Nicholls, B. And P. A. Racey (2007). "Bats avoid radar installations: could electromagnetic fields deter bats from colliding with wind turbines?" plos One 2(10): e297.	Frequency not relevant, electric current application, co-exposure or exposure condition unclear
Nicholls, B. And P. A. Racey (2009). "The aversive effect of electromagnetic radiation on foraging bats: a possible means of discouraging bats from approaching wind turbines." plos One 4(7): e6246.	Frequency not relevant, electric current application, co-exposure or exposure condition unclear
Nicolardi, G., et al. (2015). Modulation of pro-inflammatory response in a mouse model of Parkinson's disease by non-invasive physical approach, IEEE: 1-4.	Frequency not relevant, electric current application, co-exposure or exposure condition unclear
Nilsson, R., et al. (1989). "Microwave effects on the central nervous system - a study of radar mechanics." Health Physics 56(5): 777-779.	Frequency not relevant, electric current application, co-exposure or exposure condition unclear
Niu, F. S., et al. (2004). "The delayed astrocytes response in central nervous system of mice after pulsed microwave exposure." Neurobiology of Aging 25: S407-S407.	Type of study not relevant or study not EMF/health-related
Niu, T., et al. (2023). "Sirtuin 3 controls cardiac energetics and protects against oxidative stress in electromagnetic radiation-induced cardiomyopathy." Free Radical Biology & Medicine 205: 1-12.	Frequency not relevant, electric current application, co-exposure or exposure condition unclear
Novikova, N. S., et al. (2008). "Expression of the c-Fos gene in the rat hypothalamus in electrical pain stimulation and UHF stimulation of the skin." Neuroscience and Behavioral Physiology 38(4): 415-420.	Frequency not relevant, electric current application, co-exposure or exposure condition unclear
Novoselova, E. G., et al. (2001). "Effects of centimeter waves and combined magnetic field on tumor necrosis factor production in cells of mice with experimental cancer." Biofizika 46(1): 131-135.	Article not in English/German
Odland, L. T. (1973). "Radio-frequency energy: a hazard to workers?" IMS, Industrial Medicine and Surgery 42(7): 23-26.	Frequency not relevant, electric current application, co-exposure or exposure condition unclear
Oh, J. J., et al. (2018). "Effect of Electromagnetic Waves from Mobile Phones on Spermatogenesis in the Era of 4G-LTE." biomed Research International 2018: 1801798.	Frequency not relevant, electric current application, co-exposure or exposure condition unclear

Oh, S., et al. (2021). "6.5-GHz Brain Stimulation System Using Enhanced Probe Focusing and Switch-Driven Modulation." IEEE Transactions On Microwave Theory and Techniques 69(9): 4107-4117.	Type of study not relevant or study not EMF/health-related
Oh, S., et al. (2021). "Radiofrequency Irradiation Modulates TRPV1-Related Burning Sensation in Rosacea." Molecules 26(5).	Frequency not relevant, electric current application, co-exposure or exposure condition unclear
Ohmoto, Y., et al. (1996). "Sequential changes in cerebral blood flow, early neuropathological consequences and blood-brain barrier disruption following radiofrequency-induced localized hyperthermia in the rat." International Journal of Hyperthermia 12(3): 321-334.	Frequency not relevant, electric current application, co-exposure or exposure condition unclear
Okur, Z. H. And D. Sağir (2021). "Effects of cellular phone electromagnetic field exposure on the hippocampi of rats in childhood and adolescence." Neurological Sciences and Neurophysiology 38(2): 135-142.	Frequency not relevant, electric current application, co-exposure or exposure condition unclear
Olchowik, G. (2001). "Influence of hydrocortisone and microwave radiation on the mechanical characteristics of rat bone tissue." Cytobios 105(410): 147-152.	Frequency not relevant, electric current application, co-exposure or exposure condition unclear
Olsen, R. G. And J. C. Lin (1983). "Microwave-induced pressure waves in mammalian brains." IEEE Transactions on Biomedical Engineering 30(5): 289-294.	Frequency not relevant, electric current application, co-exposure or exposure condition unclear
Olsen, R. G., et al. (1987). "Rewarming of the hypothermic rhesus monkey with electromagnetic radiation." Bioelectromagnetics 8(2): 183-193.	Frequency not relevant, electric current application, co-exposure or exposure condition unclear
Onger, M. E., et al. (2016). "Effect of electromagnetic wave on bone healing in fixed and unfixed conditions." Journal of Craniofacial Surgery 27(6): 1606-1608.	Frequency not relevant, electric current application, co-exposure or exposure condition unclear
Osaka, A. (1978). "Use of microwave radiation in surgery and cancer therapy." J Microw Power 13(2): 155-161.	Frequency not relevant, electric current application, co-exposure or exposure condition unclear
Oscar, K. J., et al. (1981). "Local cerebral blood flow after microwave exposure." Brain Research 204(1): 220-225.	Frequency not relevant, electric current application, co-exposure or exposure condition unclear
Othman, E. O., M. S. Aly and S. M. El Nahas (2001). "Aneuploidy in workers occupationally exposed to	Frequency not relevant, electric current application, co-exposure or exposure condition unclear

electromagnetic field detected by FISH." Cytologia 66(2): 117-125.	
Othman, H., et al. (2017). "Effects of repeated restraint stress and wifi signal exposure on behavior and oxidative stress in rats." Metabolic Brain Disease 32(5): 1459-1469.	Frequency not relevant, electric current application, co-exposure or exposure condition unclear
Ouadah, N. S., K. Blazy and A. S. Villégier (2020). "Effect of Radiofrequency Electromagnetic Fields on Thermal Sensitivity in the Rat." International Journal of Environmental Research and Public Health 17(20): E7563.	Frequency not relevant, electric current application, co-exposure or exposure condition unclear
Ouellet-Hellstrom, R. And W. F. Stewart (1993). "Miscarriages among female physical therapists who report using radio- and microwave-frequency electromagnetic radiation." American Journal of Epidemiology 138(10): 775-786.	Frequency not relevant, electric current application, co-exposure or exposure condition unclear
Ovsyannikov, V. (2009). "Electromagnetic Fields - A Basic Carcinogenic Factor of Children Oncology." World Congress on Medical Physics and Biomedical Engineering September 7 - 12, 2009 Munich, Germany. IFMBE Proceedings, Band 25; Springer, Heidelberg, 2009; 112-114	Type of study not relevant or study not EMF/health-related
Özdemir, E., et al. (2021). "The effect of 4.5 G (LTE Advanced-Pro network) mobile phone radiation on the optic nerve." Cutaneous and Ocular Toxicology 40(3): 198-206.	Frequency not relevant, electric current application, co-exposure or exposure condition unclear
Pachocki, K. A. And A. K. Gajewski (1991). "Exposure to electromagnetic fields and risk of leukemia." Roczniki Panstwowego Zakladu Higieny (Annals of National Institute of Hygiene) 42(3): 217-221.	Frequency not relevant, electric current application, co-exposure or exposure condition unclear
Pagadala, P., V. Shankar and M. E. Sumathi (2022). "Effect of RFEMR on NSE and MDA levels in Sprague Dawley rats." Bioinformation 18(6): 501-505.	Frequency not relevant, electric current application, co-exposure or exposure condition unclear
Palmer, G. C., et al. (1975). "Action Of Psychoactive-Drugs On Cyclic-Amp Levels In Mouse Cerebral-Cortex And Lung Following Microwave Irradiation." Pharmacologist 17(2): 233-233.	Type of study not relevant or study not EMF/health-related

Pan, S. Y., A. M. Ugnat and Y. Mao (2005). "Occupational risk factors for brain cancer in Canada." Journal of Occupational and Environmental Medicine 47(7): 704-717.	Frequency not relevant, electric current application, co-exposure or exposure condition unclear
Papageorgiou, C. C., et al. (2011). "Effects of Wi-Fi signals on the P300 component of event-related potentials during an auditory Hayling task." Journal of Integrative Neuroscience 10(2): 189-202.	Frequency not relevant, electric current application, co-exposure or exposure condition unclear
Pardhiya, S., et al. (2022). "Cumulative effects of manganese nanoparticle and radiofrequency radiation in male Wistar rats." Drug and Chemical Toxicology 45(3): 1395-1407.	Frequency not relevant, electric current application, co-exposure or exposure condition unclear
Pardhiya, S., et al. (2022). "Modulatory role of Bovine serum albumin conjugated manganese dioxide nanoparticle on microwave radiation induced alterations in reproductive parameters of rat." Reproductive Toxicology 113: 136-149.	Frequency not relevant, electric current application, co-exposure or exposure condition unclear
Park, S., et al. (2012). "A new antenna system for microwave non-invasive hyperthermia lipolysis." Annu Int Conf IEEE Eng Med Biol Soc 2012: 5683-5686.	Type of study not relevant or study not EMF/health-related
Patil, A. A., et al. (1986). "Electromagnetic field focusing (EFF) probe in aneurysm thrombosis. Preliminary report." Acta Neurochir (Wien) 81(1-2): 68-71.	Frequency not relevant, electric current application, co-exposure or exposure condition unclear
Paulraj, R. and J. Behari (2008). "Biochemical Biophysical Changes of Rats Brain Exposed to Low Intensity Microwave Radiation." 2008 International Conference on Recent Advances in Microwave Theory and Applications, Jaipur, India. IEEE, 2008: 561-563	Article not peer-reviewed
Paulraj, R. and J. Behari (2019). "Low level microwave radiation: Enzymatic, morphological and DNA alteration in rat." 2019 URSI Asia-Pacific Radio Science Conference (AP-RASC), New Delhi, India. IEEE, 2019: 1	Type of study not relevant or study not EMF/health-related
Peleg, M., et al. (2023). "On radar and radio exposure and cancer in the military setting." Environmental Research 216: 114610.	Frequency not relevant, electric current application, co-exposure or exposure condition unclear
Peleg, M., O. Nativ and E. D. Richter (2018). "Radio frequency radiation-related cancer: assessing causation in the occupational/military setting." Environmental Research 163: 123-133.	Frequency not relevant, electric current application, co-exposure or exposure condition unclear

Pellegrini, A., et al. (1994). “Effects Of Microwave Exposure On Sympathetic Innervation Of Rat Myocardium.” Phys Med 1994; 10: 84-85	Frequency not relevant, electric current application, co-exposure or exposure condition unclear
Pena-Philippides, J. C., et al. (2014). "Effect of pulsed electromagnetic field (PEMF) on infarct size and inflammation after cerebral ischemia in mice." Translational Stroke Research 5(4): 491-500.	Frequency not relevant, electric current application, co-exposure or exposure condition unclear
Peng, R., et al. (2017). "The Resarch on the Autophagy and Its Markers in Hippocampal Neurons of Rats induced by Microwave Radiation." European Biophysics Journal With Biophysics Letters 46: S295-S295.	Type of study not relevant or study not EMF/health-related
Perez-Bruzon, R. N., et al. (2010). "Demodulation effect is observed in neurons by exposure to low frequency modulated microwaves." Journal of Physics: Conference Series 200: 122008.	Non-mammal/in vitro study
Perov, S. Y., N. B. Rubtsova and O. V. Belaya (2022). "Status of the Neuroendocrine System in Animals Chronically Exposed to Electromagnetic Fields of 5G Mobile Network Base Stations." Bulletin Of Experimental Biology And Medicine 174(2): 277-279.	Frequency not relevant, electric current application, co-exposure or exposure condition unclear
Pershin, S. B., et al. (2013). "Immunorehabilitating Effect of Ultrahigh Frequency Electromagnetic Fields in Immunocompromised Animals." Bulletin Of Experimental Biology And Medicine 155(2): 221-223.	Frequency not relevant, electric current application, co-exposure or exposure condition unclear
Pershin, S. B., V. D. Sidorov and S. M. Zubkova (1994). "Microwaves in the Induction of Immune Response to VI-Antigen." Electro- and Magnetobiology 13(3): 203-209.	Frequency not relevant, electric current application, co-exposure or exposure condition unclear
Persson, B. R., et al. (1992). "Increased permeability of the blood-brain barrier induced by magnetic and electromagnetic fields." Annals Of The New York Academy Of Sciences 649: 356-358.	Frequency not relevant, electric current application, co-exposure or exposure condition unclear
Pertsov, S. S., et al. (2018). "Dynamics of Metabolic Parameters in Rats during Repeated Exposure to Modulated Low-Intensity UHF Radiation." Bulletin Of Experimental Biology And Medicine 165(4): 419-423.	Frequency not relevant, electric current application, co-exposure or exposure condition unclear
Perumal, M., et al. (2022). "Pulsed electromagnetic fields for post-appendicectomy pain management: a randomized, placebo-controlled trial." Trials 23: 874.	Frequency not relevant, electric current application, co-exposure or exposure condition unclear

Petitdant, N., et al. (2016). "Cerebral radiofrequency exposures during adolescence: Impact on astrocytes and brain functions in healthy and pathologic rat models." Bioelectromagnetics 37(5): 338-350.	Frequency not relevant, electric current application, co-exposure or exposure condition unclear
Petitdant, N., et al. (2018). "Alteration of adaptive behaviors of progeny after maternal mobile phone exposure." Environmental Science and Pollution Research 25(11): 10894-10903.	Frequency not relevant, electric current application, co-exposure or exposure condition unclear
Petrova, E. V., et al. (2005). "Actions of pulsed ultra-broadband electromagnetic irradiation on the EEG and sleep in laboratory animals." Neuroscience and Behavioral Physiology 35(2): 165-170.	Frequency not relevant, electric current application, co-exposure or exposure condition unclear
Pham, L. P., et al. (1996). "Experimental nonpenetrating transscleral cyclodiathermy in rabbits." Ophthalmic Surg Lasers 27(4): 301-307.	Frequency not relevant, electric current application, co-exposure or exposure condition unclear
Pinakatt, T. L., A. W. Richardson and T. Cooper (1965). "Effect Of Digitoxin On Circulatory Response Of Rats To Microwave Irradiation." Archives Internationales de Pharmacodynamie et de Therapie 156(1): 151.	Frequency not relevant, electric current application, co-exposure or exposure condition unclear
Pinakatt, T. L., A. W. Richardson and T. Cooper (1965). "Effect of digitoxin on the circulatory response of rats to microwave irradiation." Arch Int Pharmacodyn Ther 156(1): 151-160.	Frequency not relevant, electric current application, co-exposure or exposure condition unclear
Pinakatt, T., T. Cooper and A. W. Richardson (1963). "Effect of ouabain on the circulatory response to microwave hyperthermia in rat." Aerosp Med 34: 497-499.	Frequency not relevant, electric current application, co-exposure or exposure condition unclear
Pinakatt, T., T. Cooper and A. W. Richardson (1963). "Effect of ouabain on the circulatory response to microwave hyperthermia in rat." Aerospace Medicine 34: 497-499.	Frequency not relevant, electric current application, co-exposure or exposure condition unclear
Pitenin, I. V. And A. G. Subbota (1965). "Formation of Gastric Ulcer in Rabbits Following Microwave Irradiation of Epigastrium." Bulletin Of Experimental Biology and Medicine, USSR 60(9): 1025.	Frequency not relevant, electric current application, co-exposure or exposure condition unclear
Pitenin, I. V. And A. G. Subbota (1965). "Formation of Gastric Ulcer in Rabbits Following Microwave Irradiation of Epigastrium." Bulletin Of Experimental Biology and Medicine 60(9): 1025-1028.	Frequency not relevant, electric current application, co-exposure or exposure condition unclear

Pletnev, S. D. (2000). "The use of millimeter band electromagnetic waves in clinical oncology." Critical Reviews in Biomedical Engineering 28(3-4): 573-587.	Frequency not relevant, electric current application, co-exposure or exposure condition unclear
Pobzhitk, V., A. Grebeshe, and N. V. Tyagin (1961). "Influence of a Super-High Frequency Pulsed Electromagnetic Field on Conception and Course of Pregnancy in White Mice." Bulletin of Experimental Biology and Medicine 51(5): 615.	Frequency not relevant, electric current application, co-exposure or exposure condition unclear
Pobzhitkov, V. A., N. V. Tyagin and A. M. Grebeshechnikova (1961). "The influence of a super-high frequency pulsed electro-magnetic field on conception and the course of pregnancy in white mice." Biull Eksp Biol Med 51: 615-618.	Frequency not relevant, electric current application, co-exposure or exposure condition unclear
Pobzhitkov, V. A., N. V. Tyagin and A. M. Grebeshechnikova (1961). "The influence of a super-high frequency pulsed electro-magnetic field on conception and the course of pregnancy in white mice." Biulleten' Eksperimental'noi Biologii i Meditsiny 51: 615-618.	Frequency not relevant, electric current application, co-exposure or exposure condition unclear
Pollack, H. (1979). "Epidemiologic data on American personnel in the Moscow embassy." Bulletin of the New York Academy of Medicine 55(11): 1182-1186.	Type of study not relevant or study not EMF/health-related
Pumprla, J., et al. (2015). "Non-contact radiofrequency-induced reduction of subcutaneous abdominal fat correlates with initial cardiovascular autonomic balance and fat tissue hormones: safety analysis." F1000Res 4: 49.	Frequency not relevant, electric current application, co-exposure or exposure condition unclear
Qiao, S., et al. (2014). "Reduction of phosphorylated synapsin I (Ser-553) leads to spatial memory impairment by attenuating GABA release after microwave exposure in Wistar rats." plos One 9(4): e95503.	Frequency not relevant, electric current application, co-exposure or exposure condition unclear
Qiao, Y., et al. (2023). "Enhancing Microwave Dynamic Effects via Surface States of Ultrasmall 2D MOF Triggered by Interface Confinement for Antibiotics-Free Therapy." Adv Sci (Weinh) 10(21): e2300084.	Frequency not relevant, electric current application, co-exposure or exposure condition unclear
Qubty, D., et al. (2021). "No Significant Effects of Cellphone Electromagnetic Radiation on Mice Memory or Anxiety: Some Mixed Effects on Traumatic Brain Injured Mice." Neurotrauma Reports 2(1): 381-390.	Frequency not relevant, electric current application, co-exposure or exposure condition unclear

Quock, R. M., et al. (1986). "Microwave facilitation of methylatropine antagonism of central cholinomimetic drug effects." Radiation Research 105: 328-340.	Frequency not relevant, electric current application, co-exposure or exposure condition unclear
Rabenok, L., V. Grimalsky and J. De La Hidalga (2006). A method of treatment of purulent-inflammatory diseases of the hand in outpatient clinic with using electromagnetic microwave field. 854: 81-+.	Frequency not relevant, electric current application, co-exposure or exposure condition unclear
Rabini, A., et al. (2012). "Deep heating therapy via microwave diathermy relieves pain and improves physical function in patients with knee osteoarthritis: a double-blind randomized clinical trial." European Journal of Physical and Rehabilitation Medicine 48(4): 549-560.	Frequency not relevant, electric current application, co-exposure or exposure condition unclear
Rangkooy, H., A. Rahmati and B. F. Dehaghi (2023). "Base transceiver station antenna exposure and workers' health." International Journal of Occupational Safety and Ergonomics 29(2): 863-868.	Frequency not relevant, electric current application, co-exposure or exposure condition unclear
Rashidova, A. M. (2021). "Differential Effects of Decimetric Electromagnetic Microwaves on Pyruvate Kinase Activity in the Rat Brain during Ontogenesis." Journal of Evolutionary Biochemistry and Physiology 57(2): 241-251.	Frequency not relevant, electric current application, co-exposure or exposure condition unclear
Raslear, T. G., et al. (1993). The Effects Of High-Peak Power Pulsed Microwave Irradiation On Time Bisection In Rats: 671-674.	Article not peer-reviewed
Rasouli, J., et al. (2012). "Attenuation of interleukin-1beta by pulsed electromagnetic fields after traumatic brain injury." Neuroscience Letters 519(1): 4-8.	Frequency not relevant, electric current application, co-exposure or exposure condition unclear
Rawe, I. M., et al. (2012). "Control of postoperative pain with a wearable continuously operating pulsed radiofrequency energy device: a preliminary study." Aesthetic Plastic Surgery 36(2): 458-463.	Frequency not relevant, electric current application, co-exposure or exposure condition unclear
Redmayne, M., E. Smith and M. J. Abramson (2013). "The relationship between adolescents' well-being and their wireless phone use: a cross-sectional study." Environmental Health 12: 90.	Type of study not relevant or study not EMF/health-related
Reeves, G. I. (2000). "Review of extensive workups of 34 patients overexposed to radiofrequency radiation." Aviat Space Environ Med 71(3): 206-215.	Type of study not relevant or study not EMF/health-related

Reif, J. S., N. Pearce and J. Fraser (1989). "Occupational risks for brain cancer: a New Zealand Cancer Registry-based study." Journal of Occupational Medicine 31(10): 863-867.	Frequency not relevant, electric current application, co-exposure or exposure condition unclear
Ren, H., et al. (2018). "Pulsed Radiofrequency Applied to the Sciatic Nerve Improves Neuropathic Pain by Down-regulating The Expression of Calcitonin Gene-related Peptide in the Dorsal Root Ganglion." Int J Med Sci 15(2): 153-160.	Frequency not relevant, electric current application, co-exposure or exposure condition unclear
Rhatomy, S., et al. (2020). "The efficacy of pulsed radiofrequency intervention of the lumbar dorsal root ganglion in patients with chronic lumbar radicular pain." The Medical Journal of Malaysia 75(2): 124-129.	Frequency not relevant, electric current application, co-exposure or exposure condition unclear
Richardson, A. W., T. D. Duane and H. M. Hines (1948). "Experimental lenticular opacities produced by microwave irradiations." Archives of Physical Medicine 29(12): 765-769.	Frequency not relevant, electric current application, co-exposure or exposure condition unclear
Richardson, A. W., T. D. Duane and H. M. Hines (1951). "Experimental cataract produced by three cenimeter pulsed microwave irradiations." AMA Archives of Ophthalmology 45(4): 382-386.	Frequency not relevant, electric current application, co-exposure or exposure condition unclear
Richter, E., et al. (2000). "Cancer in radar technicians exposed to radiofrequency/microwave radiation: sentinel episodes." International Journal of Occupational and Environmental Health 6(3): 187-193.	Type of study not relevant or study not EMF/health-related
Richter, W. R. And D. H. Yost (1964). "A Morphological Study Of Rat Testicular Tissue Exposed To Rf Energy. REP 601." Rep US Army Med Res Lab: 1-3.	Frequency not relevant, electric current application, co-exposure or exposure condition unclear
Rinaldi, S., et al. (2010). "A new approach on stress-related depression & anxiety: Neuro-Psycho- Physical-Optimization with Radio Electric Asymmetric-Conveyer." The Indian Journal of Medical Research 132: 189-194.	Frequency not relevant, electric current application, co-exposure or exposure condition unclear
Rinaldi, S., et al. (2010). "Psychological and symptomatic stress-related disorders with radio-electric treatment: psychometric evaluation." Stress and Health: Journal of the International Society for the Investigation of Stress 26(5): 350-358.	Frequency not relevant, electric current application, co-exposure or exposure condition unclear

Rinaldi, S., et al. (2011). "Stress-related psycho-physiological disorders: randomized single blind placebo controlled naturalistic study of psychometric evaluation using a radio electric asymmetric treatment." Health and Quality of Life Outcomes 9: 54.	Frequency not relevant, electric current application, co-exposure or exposure condition unclear
Rittweger, J., et al. (1995). "Influence of modulated high-frequency electromagnetic fields on the functional organization and dynamics of the common brainstem system." Bioelectrochemistry And Bioenergetics 37: 31-37.	Frequency not relevant, electric current application, co-exposure or exposure condition unclear
Robinette, C. D., C. Silverman and S. Jablon (1980). "Effects upon health of occupational exposure to microwave radiation (radar)." American Journal of Epidemiology 112(1): 39-53.	Frequency not relevant, electric current application, co-exposure or exposure condition unclear
Rodrigues, N. C. P., et al. (2021). "The Effect of Continuous Low-Intensity Exposure to Electromagnetic Fields from Radio Base Stations to Cancer Mortality in Brazil." International Journal of Environmental Research and Public Health 18(3): 1229.	Frequency not relevant, electric current application, co-exposure or exposure condition unclear
Rohde, C. H., et al. (2015). "Pulsed Electromagnetic Fields Reduce Postoperative Interleukin-1beta, Pain, and Inflammation: A Double-Blind, Placebo-Controlled Study in TRAM Flap Breast Reconstruction Patients." Plastic & Reconstructive Surgery 135(5): 808e-817e.	Frequency not relevant, electric current application, co-exposure or exposure condition unclear
Rohde, C., et al. (2010). "Effects of pulsed electromagnetic fields on interleukin-1 beta and postoperative pain: a double-blind, placebo-controlled, pilot study in breast reduction patients." Plastic & Reconstructive Surgery 125(6): 1620-1629.	Frequency not relevant, electric current application, co-exposure or exposure condition unclear
Rohof, O. (2012). "Intradiscal pulsed radiofrequency application following provocative discography for the management of degenerative disc disease and concordant pain: a pilot study." Pain Pract 12(5): 342-349.	Frequency not relevant, electric current application, co-exposure or exposure condition unclear
Röösli, M., E. Mohler and P. Frei (2010). "Sense and sensibility in the context of radiofrequency electromagnetic field exposure." Comptes Rendus Physique 11(9-10): 576-584.	Frequency not relevant, electric current application, co-exposure or exposure condition unclear

Rorvik, D. M. (1975). "What's better than the pill, vasectomy, celibacy and rhythm?" Esquire 83(1): 100-102.	Article not peer-reviewed
Rosenbaum, P. F., et al. (1994). "Occupational exposures associated with male breast cancer." American Journal of Epidemiology 139(1): 30-36.	Frequency not relevant, electric current application, co-exposure or exposure condition unclear
Roszkowski, W., et al. (1980). "Does whole-body hyperthermia therapy involve participation of the immune system?" International Journal of Cancer 25(2): 289-292.	Frequency not relevant, electric current application, co-exposure or exposure condition unclear
Roszkowski, W., et al. (1980). "Effect of whole-body microwave hyperthermia on delayed cutaneous hypersensitivity in tumor-bearing mice." Journal of Cancer Research and Clinical Oncology 97(1): 23-29.	Frequency not relevant, electric current application, co-exposure or exposure condition unclear
Roszkowski, W., et al. (1980). "The search for an influence of whole-body microwave hyperthermia on anti-tumor immunity." Journal of Cancer Research and Clinical Oncology 96(3): 311-317.	Frequency not relevant, electric current application, co-exposure or exposure condition unclear
Roszkowski, W., et al. (1981). "Effect of whole-body hyperthermia on delayed cutaneous hypersensitivity to oxazolone in mice." J Clin Lab Immunol 6(3): 257-261.	Frequency not relevant, electric current application, co-exposure or exposure condition unclear
Rotkovska, D. And A. Vacek (1976). "Proceedings: Modification of the repair of radiation damage of haematopoiesis in mice by microwaves." J Microw Power 11(2): 141-143.	Frequency not relevant, electric current application, co-exposure or exposure condition unclear
Rotkovska, D., et al. (1973). "Non-Contact Determination Of Temperature-Changes In Mice During Microwave Irradiation." Physiologia Bohemoslovaca 22(4): 359-363.	Frequency not relevant, electric current application, co-exposure or exposure condition unclear
Rotkovská, D., et al. (1973). "Non-contact determination of temperature changes in mice during microwave irradiation." Physiol Bohemoslov 22(4): 359-363.	Frequency not relevant, electric current application, co-exposure or exposure condition unclear
Roux, C., et al. (1986). "Association of Microwaves and Ionizing-Radiation - Potentiation of Teratogenic Effects in the Rat." Radiation Research 108(3): 317-326.	Frequency not relevant, electric current application, co-exposure or exposure condition unclear
Rudnev, M. I., D. P. Morozova and V. P. Artiukh (1987). "Effect of Microwave-Radiation of the Blood-Serum Lipid-Content in Rats." Vrachebnoe Delo(10): 118-121.	Article not in English/German

Rugh, R. And M. Mcmanaway (1976). "Are Mouse Fetuses Uniformly Sensitive to Microwave Radiation." Teratology 13(2): A34-A35.	Type of study not relevant or study not EMF/health-related
Rugh, R. And M. Mcmanaway (1976). "Comparison of Ionizing and Microwave Radiations with Respect to Their Effects on Rodent Embryo and Fetus." Teratology 14(2): 251-251.	Type of study not relevant or study not EMF/health-related
Rugh, R., et al. (1975). "Responses Of Mouse to Microwave Radiation During Estrous-Cycle and Pregnancy." Radiation Research 62(2): 225-241.	Frequency not relevant, electric current application, co-exposure or exposure condition unclear
Rugh, R., et al. (1975). "Responses of the mouse to microwave radiation during estrous cycle and pregnancy." Radiation Research 62(1): 225-241.	Frequency not relevant, electric current application, co-exposure or exposure condition unclear
Rugh, R., H. Ho and M. Mcmanaway (1976). "The relation of dose rate of microwave radiation to the time of death and total absorbed dose in mice." The Journal of Microwave Power 11(3): 279-281.	Frequency not relevant, electric current application, co-exposure or exposure condition unclear
Rugh, R., H. Ho and M. Mcmanaway (1976). "The relation of dose rate of microwave radiation to the time of death and total absorbed dose in the mouse." J Microw Power 11(3): 279-281.	Frequency not relevant, electric current application, co-exposure or exposure condition unclear
Rupp, T., J. Montet and J. W. Frazer (1975). "Comparison of Thermal and Radio-Frequency Exposure Effects on Trace-Metal Content of Blood-Plasma and Liver-Cell Fractions of Rodents." Annals of The New York Academy of Sciences 247(Feb28): 282-291.	Frequency not relevant, electric current application, co-exposure or exposure condition unclear
Rusnani, A. et al. (2008). “Microwave Radiation Effect - A Test on White Mice.” 2008 IEEE International RF and Microwave Conference, Kuala Lumpur, Malaysia. IEEE, 2008: 262-267	Frequency not relevant, electric current application, co-exposure or exposure condition unclear
Saddiki-Traki, F., G. Lescoat and J. Maniey (1986). "Effects of postnatal microwave exposure on thyrotropin level in the adult male rat." Journal de Physiologie 81: 3-6.	Frequency not relevant, electric current application, co-exposure or exposure condition unclear
Sadetzki, S., et al. (2014). "The MOBI-Kids Study Protocol: Challenges in Assessing Childhood and Adolescent Exposure to Electromagnetic Fields from Wireless Telecommunication Technologies and Possible Association with Brain Tumor Risk." Front Public Health 2: 124.	Type of study not relevant or study not EMF/health-related

Safiruddin, F., et al. (2013). "Long-term self-reported treatment effects and experience of radiofrequency-induced thermotherapy of the inferior turbinates performed under local anesthesia: a retrospective analysis." European Archives of Oto-Rhino-Laryngology 270(6): 1849-1853.	Frequency not relevant, electric current application, co-exposure or exposure condition unclear
Sajin, G. et al. (2001). "Tumour remission in rats by irradiation with non-thermal power levels microwaves." 2001 Conference Proceedings of the 23rd Annual International Conference of the IEEE Engineering in Medicine and Biology Society, Istanbul, Turkey. Band 4; IEEE, 2001: 3913-3916	Frequency not relevant, electric current application, co-exposure or exposure condition unclear
Salsi, B. And I. Fusco (2022). "Non-invasive system delivering microwaves energy for unwanted fat reduction and submental skin tightening: Clinical evidence." Journal of Cosmetic Dermatology 21(11): 5657-5664.	Frequency not relevant, electric current application, co-exposure or exposure condition unclear
Salzberg, C. A., et al. (1995). "The effects of non-thermal pulsed electromagnetic energy on wound healing of pressure ulcers in spinal cord-injured patients: a randomized, double-blind study." Ostomy Wound Management 41(3): 42.	Frequency not relevant, electric current application, co-exposure or exposure condition unclear
Samaras, G. M., L. R. Muroff and G. E. Anderson (1971). "Prolongation of Life During High-Intensity Microwave Exposures." IEEE Transactions on Microwave Theory And Techniques 61(5): 245-247.	Frequency not relevant, electric current application, co-exposure or exposure condition unclear
Santini, R. (1982). "Effect of Low-Level Microwave Irradiation on The Duodenal Electrical-Activity of the Unanesthetized Rat." Journal of Microwave Power and Electromagnetic Energy 17(4): 329-334.	Frequency not relevant, electric current application, co-exposure or exposure condition unclear
Sanza, J. N. And J. De Lorge (1977). "Fixed interval behavior of rats to microwaves at low power densities." Radio Science 12: 273-277.	Frequency not relevant, electric current application, co-exposure or exposure condition unclear
Sapozink, M. D., D. R. Goffinet and G. M. Hahn (1982). "Combined Continuous Low-Dose Rate Irradiation and Radiofrequency Hyperthermia in the C3h Mouse." Radiation Research 91(2): 312-312.	Type of study not relevant or study not EMF/health-related
Sarkar, S., B. Kumari and S. Ali (2006). "Effect of radiofrequency electromagnetic field on human DNA." Defence Science Journal 56(2): 199-208.	Type of study not relevant or study not EMF/health-related

Satoh, T., S. Nakasone and A. Nishimoto (1989). "Cerebral blood flow response to the tissue temperature in tumour and brain tissues." Int J Hyperthermia 5(6): 683-696.	Frequency not relevant, electric current application, co-exposure or exposure condition unclear
Savić, S., et al. (1983). "The effect of microwave radiation on the eye lens in rats." Arh Hig Rada Toksikol 34(4): 301-304.	Frequency not relevant, electric current application, co-exposure or exposure condition unclear
Schiffner, G., F. Zywietz and W. Lierse (1988). "Histologic And Morphometric Investigations In Rhabdomyosarcomas Of Rats After Irradiation And Microwave Hyperthermia." Strahlentherapie Und Onkologie 164(2): 94-104.	Frequency not relevant, electric current application, co-exposure or exposure condition unclear
Schmidt-Pokrzywniak, A., et al. (2004). "Case-control study on uveal melanoma (RIFA): rational and design." BMC Ophthalmol 4: 11.	Type of study not relevant or study not EMF/health-related
Schmidt-Pokrzywniak, A., et al. (2010). "A case-control study: occupational cooking and the risk of uveal melanoma." BMC Ophthalmol 10: 26.	Frequency not relevant, electric current application, co-exposure or exposure condition unclear
Schneider, R. (2022). "Mobile phone induced EMF stress is reversed upon the use of protective devices: results from two experiments testing different boundary conditions." Electromagnetic Biology and Medicine 41(4): 429-438.	Frequency not relevant, electric current application, co-exposure or exposure condition unclear
Schrader, S. M., et al. (1998). "Reproductive function in relation to duty assignments among military personnel." Reproductive Toxicology 12(4): 465-468.	Frequency not relevant, electric current application, co-exposure or exposure condition unclear
Schvarcz, C. A., et al. (2021). "Modulated Electro-Hyperthermia Induces a Prominent Local Stress Response and Growth Inhibition in Mouse Breast Cancer Isografts." Cancers 13(7): 1744.	Frequency not relevant, electric current application, co-exposure or exposure condition unclear
Seaman, R. L. And J. R. Jauchem (2004). "Rat electrocardiogram during acute exposure to synchronized bursts of ultra-wideband pulses." IEEE Transactions On Plasma Science 32(4): 1644-1652.	Frequency not relevant, electric current application, co-exposure or exposure condition unclear
Seaman, R. L. And R. M. Lebovitz (1987). "Auditory unit responses to single-pulse and twin-pulse microwave stimuli." Hearing Research 26(1): 105-116.	Frequency not relevant, electric current application, co-exposure or exposure condition unclear

Seaman, R. L., et al. (1998). "Ultra-wideband electromagnetic pulses and morphine-induced changes in nociception and activity in mice." Physiology & Behavior 65(2): 263-270.	Frequency not relevant, electric current application, co-exposure or exposure condition unclear
Seaman, R. L., et al. (1999). "Hyperactivity caused by a nitric oxide synthase inhibitor is countered by ultra-wideband pulses." Bioelectromagnetics 20(7): 431-439.	Frequency not relevant, electric current application, co-exposure or exposure condition unclear
Seo, T., et al. (2018). "Noninvasive Brain Stimulation Using a Modulated Microwave Signal." Journal of Electromagnetic Engineering and Science 18(1): 70-72.	Type of study not relevant or study not EMF/health-related
Serin, M., et al. (2023). "Investigation of gene expression levels in thyroid tissues of rats treated with Wi-Fi electromagnetic wave (2.4-3 GHz Wi-Fi RF-EMF)." Journal of Molecular Structure 1288: 135741.	Frequency not relevant, electric current application, co-exposure or exposure condition unclear
Seth, H. S. And S. M. Michaelson (1965). "Microwave cataractogenesis." Journal of Occupational Medicine 46: 439-442.	Frequency not relevant, electric current application, co-exposure or exposure condition unclear
Seth, H. S. And S. M. Michaelson (1965). "Microwave Cataractogenesis." J Occup Med 7: 439-442.	Frequency not relevant, electric current application, co-exposure or exposure condition unclear
Shacklett, D. E., T. J. Tredici and D. L. Epstein (1975). "Evaluation of possible microwave-induced lens changes in the United States Air Force." Aviation, Space & Environmental Medicine 46(11): 1403-1406.	Frequency not relevant, electric current application, co-exposure or exposure condition unclear
Shaheen, W., et al. (2021). "Effect of Antioxidants Intake on Oxidative Stress Among Mobile Phone Users." Egyptian Journal of Chemistry 64(7): 3903-3912.	Frequency not relevant, electric current application, co-exposure or exposure condition unclear
Shahi, A., et al. (2021). "Does exposure to radiation emitted from mobile jammers influence the spatial memory?" International Journal of Radiation Research 19(4): 993-1000.	Frequency not relevant, electric current application, co-exposure or exposure condition unclear
Shandala, M. G. (1988). "Possible physiological mechanisms for neurobehavioral effects of electromagnetic exposure." Progress in Clinical and Biological Research 257: 367-376.	Frequency not relevant, electric current application, co-exposure or exposure condition unclear
Shanin, S. N., et al. (2005). "Natural killer cell cytotoxic activity and c-Fos protein synthesis in rat hypothalamic cells after painful electric stimulation of	Frequency not relevant, electric current application, co-exposure or exposure condition unclear

the hind limbs and EHF irradiation of the skin." Medical Science Monitor 11(9): BR309-BR315.	
Shapira, S., et al. (2023). "Occupational Exposure to Nonionizing Radiation and Risk for Malignancy in Young Adults." Military Medicine.	Frequency not relevant, electric current application, co-exposure or exposure condition unclear
Sharaf, N. E., et al. (2019). "Role of Designed Bio-Geometrical Forms in Antagonizing Neurobehavioral Burden of Wi-Fi Radiation: Evidence-Based Experimental Study." Biomedical & Pharmacology Journal 12(3): 1217-1234.	Frequency not relevant, electric current application, co-exposure or exposure condition unclear
Sharma, D., A. Chauhan and S. Thakur (2022). "Effect of Mobile Phone use on Hearing in Young Adults: An Observational Study." Indian Journal of Otolaryngology and Head and Neck Surgery 74: 3754-3757.	Frequency not relevant, electric current application, co-exposure or exposure condition unclear
Sharp, J. C. And C. J. Paperiello (1971). "The effects of microwave exposure on thymidine-3H uptake in albino rats." Radiat Res 45(2): 434-439.	Frequency not relevant, electric current application, co-exposure or exposure condition unclear
Sharpless, N. S. And L. L. Brown (1978). "Use of Microwave Irradiation to Prevent Postmortem Catecholamine Metabolism - Evidence for Tissue Disruption Artifact in a Discrete Region of Rat-Brain." Brain Research 140(1): 171-176.	Frequency not relevant, electric current application, co-exposure or exposure condition unclear
Sharqawi, M., et al. (2022). "The Impact of Lifestyle on Sperm Function, Telomere Length, and IVF Outcomes." American Journal of Men's Health 16(5): 15579883221119931.	Frequency not relevant, electric current application, co-exposure or exposure condition unclear
Shen, H., et al. (2011). "The regimen of gemcitabine and cisplatin combined with radio frequency hyperthermia for advanced non-small cell lung cancer: a phase II study." International Journal of Hyperthermia 27(1): 27-32.	Frequency not relevant, electric current application, co-exposure or exposure condition unclear
Shih, Y. W., et al. (2020). "The Association Between Smartphone Use and Breast Cancer Risk Among Taiwanese Women: A Case-Control Study." Cancer Management and Research 12: 10799-10807.	Frequency not relevant, electric current application, co-exposure or exposure condition unclear

Shirai, T., et al. (2017). "No adverse effects detected for simultaneous whole-body exposure to multiple-frequency radiofrequency electromagnetic fields for rats in the intrauterine and pre- and post-weaning periods." Journal of Radiation Research 58(1): 48-58.	Frequency not relevant, electric current application, co-exposure or exposure condition unclear
Shojaeifard, M. B., et al. (2018). "Electromagnetic Fields of Mobile Phone Jammer Exposure on Blood Factors in Rats." Journal of Biomedical Physics & Engineering 8(4): 403-408.	Frequency not relevant, electric current application, co-exposure or exposure condition unclear
Shokoohi-Rad, S., et al. (2020). "Comparison of Intraocular Pressure Changes Due to Exposure to Mobile Phone Electromagnetics Radiations in Normal and Glaucoma Eye." Middle East African Journal of Ophthalmology 27(1): 10-13.	Frequency not relevant, electric current application, co-exposure or exposure condition unclear
Shrivastav, S., et al. (1983). "Microwave hyperthermia and its effect on tumor blood flow in rats." Cancer Research 43(10): 4665-4669.	Frequency not relevant, electric current application, co-exposure or exposure condition unclear
Shrivastav, S., W. T. Joines and R. L. Jirtle (1985). "Effect of 5-hydroxytryptamine on tissue blood flow and microwave heating of rat tumors." Cancer Research 45(7): 3203-3208.	Frequency not relevant, electric current application, co-exposure or exposure condition unclear
Shui, X. P., et al. (2023). "Effects of millimeter-wave for preventing joint stiffness in the immobilized knee rat model." The Knee 42: 236-245.	Frequency not relevant, electric current application, co-exposure or exposure condition unclear
Sibghatullah, H., et al. (2021). "Amelioration of Cell Phone and Wi Fi induced Pancreatic Damage and Hyperglycemia (Diabetes Mellitus) with Pomegranate and Vit E in Rats." Journal of Pharmaceutical Research International 33(54B): 204-215.	Frequency not relevant, electric current application, co-exposure or exposure condition unclear
Sidorenko, A. V. And V. V. Tsaryuk (2000). "Effects of microwave radiation and strychnine on cerebral biopotentials in narcotized rats." Bulletin of Experimental Biology and Medicine 130(9): 835-837.	Frequency not relevant, electric current application, co-exposure or exposure condition unclear
Siekierzynski, M., et al. (1974). "Health surveillance of personnel occupationally exposed to microwaves. III. Lens translucency." Aerospace Medicine 45(10): 1146-1148.	Frequency not relevant, electric current application, co-exposure or exposure condition unclear
Siekierzynski, M., et al. (1974). "Health surveillance of personnel occupationally exposed to microwaves. II. Functional disturbances." Aerospace Medicine 45(10): 1143-1145.	Frequency not relevant, electric current application, co-exposure or exposure condition unclear

Singh, K. V., et al. (2019). Cellphone radiofrequency radiation induced inflammatory response and oxidative stress in rat brain, IEEE: 1.	Frequency not relevant, electric current application, co-exposure or exposure condition unclear
Singh, K. V., et al. (2022). "Effects of mobile phone electromagnetic radiation on rat hippocampus proteome." Environmental Toxicology 37(4): 836-847.	Frequency not relevant, electric current application, co-exposure or exposure condition unclear
Singh, M. M., et al. (2022). "Does exposure to radiofrequency radiation (RFR) affect the circadian rhythm of rest-activity patterns and behavioral sleep variables in humans?" Biological Rhythm Research 53(9): 1414-1438.	Frequency not relevant, electric current application, co-exposure or exposure condition unclear
Singh, S. And N. Kapoor (2015). "Occupational EMF exposure from radar at X and Ku frequency band and plasma catecholamine levels." Bioelectromagnetics 36(6): 444-450.	Type of study not relevant or study not EMF/health-related
Singh, S., K. V. Mani and N. Kapoor (2015). "Effect of occupational EMF exposure from radar at two different frequency bands on plasma melatonin and serotonin levels." International Journal of Radiation Biology 91(5): 426-434.	Frequency not relevant, electric current application, co-exposure or exposure condition unclear
Sinotova, O. A., et al. (2004). "A comparison of the effects of millimeter and centimeter waves on tumor necrosis factor production in mouse cells." Biofizika 49(3): 545-550.	Article not in English/German
Sitko, S. P., et al. (1996). "Impact of millimeter wave electromagnetic radiation upon Guerin's carcinoma in rats and Lewis lung carcinoma in mice." Experimental Oncology 18(4): 419-422.	Frequency not relevant, electric current application, co-exposure or exposure condition unclear
Slone, H. T., J. W. Kosh and J. J. Freeman (1986). "The effect of hemicholinium-3 and physostigmine on choline in rat brain." Res Commun Chem Pathol Pharmacol 52(1): 133-136.	Frequency not relevant, electric current application, co-exposure or exposure condition unclear
Slotman, G. J., et al. (1980). "The effect of radiofrequency hyperthermia on the Ca755 murine adenocarcinoma." Cancer 46(9): 1992-1995.	Frequency not relevant, electric current application, co-exposure or exposure condition unclear
Smeds, H., et al. (2018). "Occurrence of primary brain tumors in cochlear implant patients in Sweden between 1989 and 2014." Clinical Epidemiology 10: 1401-1405.	Frequency not relevant, electric current application, co-exposure or exposure condition unclear

Smialowicz, R. J., J. B. Kinn and J. A. Elder (1979). "Perinatal exposure of rats to 2450 MHz CW microwave radiation: Effects on lymphocytes." Radio Science 14: 147-153.	Frequency not relevant, electric current application, co-exposure or exposure condition unclear
Smialowicz, R. J., P. L. Brugnolotti and M. M. Riddle (1981). "Complement Receptor Positive Spleen-Cells in Microwave (2450-MHz)-Irradiated Mice." Journal of Microwave Power and Electromagnetic Energy 16(1): 73-77.	Frequency not relevant, electric current application, co-exposure or exposure condition unclear
Sokolovic, D. And D. M. Sokolovic (2014). "Apoptosis and Oxidative Stress Induced by Exposure of Microwave Radiation In Rat Thymus: Modulatory Effect of Melatonin." Value Health 17(7): A543.	Type of study not relevant or study not EMF/health-related
Sokolovic, D., et al. (2013). "Melatonin protects rat thymus against oxidative stress caused by exposure to microwaves and modulates proliferation/apoptosis of thymocytes." General Physiology and Biophysics 32(1): 79-90.	Frequency not relevant, electric current application, co-exposure or exposure condition unclear
Sokolovic, D., et al. (2015). "The Effects of Melatonin on Oxidative Stress Parameters and DNA Fragmentation in Testicular Tissue of Rats Exposed to Microwave Radiation." Advances in Clinical and Experimental Medicine 24(3): 429-436.	Frequency not relevant, electric current application, co-exposure or exposure condition unclear
Soldani, P., et al. (1995). "Age-related changes in the noradrenergic pattern and receptor responses of the rat cardiovascular system after repeated microwave exposure." International Journal of Neuroscience 82(1-2): 61-69.	Frequency not relevant, electric current application, co-exposure or exposure condition unclear
Soldani, P., et al. (1995). "Age-Related-Changes in the Noradrenergic Pattern and Receptor Responses of the Rat Cardiovascular-System After Repeated Microwave Exposure." International Journal of Neuroscience 82(1-2): 61-69.	Frequency not relevant, electric current application, co-exposure or exposure condition unclear
Soleimani, H., et al. (2022). "Comparison of the effect of changing the spatial distance with exposure time to mobile phones radiation on the structure and function of the testis in NMRI mice." Journal Of Microwave Power and Electromagnetic Energy 56(2): 87-102.	Frequency not relevant, electric current application, co-exposure or exposure condition unclear
Son, M., et al. (2020). "Radiofrequency irradiation attenuates angiogenesis and inflammation in UVB-induced rosacea in mouse skin." Exp Dermatol 29(7): 659-666.	Frequency not relevant, electric current application, co-exposure or exposure condition unclear

Sorahan, T., et al. (1999). "Maternal occupational exposure to electromagnetic fields before, during, and after pregnancy in relation to risks of childhood cancers: findings from the Oxford Survey of Childhood Cancers, 1953-1981 deaths." American Journal of Industrial Medicine 35(4): 348-357.	Frequency not relevant, electric current application, co-exposure or exposure condition unclear
Sorrell, R. G., et al. (2018). "Evaluation of pulsed electromagnetic field therapy for the treatment of chronic postoperative pain following lumbar surgery: a pilot, double-blind, randomized, sham-controlled clinical trial." Journal of Pain Research 11: 1209-1222.	Frequency not relevant, electric current application, co-exposure or exposure condition unclear
Srujana Aravinda, V. S., et al. (2022). "A cross-sectional and histological analysis to understand the cytological effects of cell phone radiation on buccal mucosa of children." Journal of the Indian Society of Pedodontics and Preventive Dentistry 40(1): 74-80.	Frequency not relevant, electric current application, co-exposure or exposure condition unclear
Stang, A., et al. (2001). "The possible role of radiofrequency radiation in the development of uveal melanoma." Epidemiology 12(1): 7-12.	Frequency not relevant, electric current application, co-exposure or exposure condition unclear
Stavinoh.Wb, B. Pepelko and P. W. Smith (1970). "Microwave Radiation to Inactivate Cholinesterase in Rat Brain Prior to Analysis for Acetylcholine." Pharmacologist 12(2): 257-&.	Type of study not relevant or study not EMF/health-related
Stern, S. (1980). "Behavioral effects of microwaves." Neurobehavioral Toxicology 2(1): 49-58.	Type of study not relevant or study not EMF/health-related
Sterzer, F., et al. (2000). "Microwave treatments for prostate disease." Ieee Transactions on Microwave Theory And Techniques 48(11): 1885-1891.	Frequency not relevant, electric current application, co-exposure or exposure condition unclear
Stoner, E. K. (1951). "The effect of microwave radiation on the peripheral pulse volume, digital skin temperature and digital blood flow in man." Arch Phys Med Rehabil 32(6): 408-416.	Frequency not relevant, electric current application, co-exposure or exposure condition unclear
Storm, F. K., et al. (1981). "Clinical radiofrequency hyperthermia by magnetic-loop induction." The Journal of Microwave Power 16(2): 179-184.	Frequency not relevant, electric current application, co-exposure or exposure condition unclear
Strauch, B., et al. (2006). "Pulsed magnetic field therapy increases tensile strength in a rat Achilles' tendon repair model." J Hand Surg Am 31(7): 1131-1135.	Frequency not relevant, electric current application, co-exposure or exposure condition unclear

Strauch, B., et al. (2006). "Pulsed magnetic field therapy increases tensile strength in a rat Achilles' tendon repair model." Journal Of Hand Surgery-American Volume 31A(7): 1131-1135.	Frequency not relevant, electric current application, co-exposure or exposure condition unclear
Subbotina, T. I., et al. (2004). "Effect of delta-rhythm-modulated extremely high frequency electromagnetic radiation on rats." Bulletin Of Experimental Biology and Medicine 137(5): 423-424.	Frequency not relevant, electric current application, co-exposure or exposure condition unclear
Subbotina, T. I., et al. (2006). "Effect of low-intensity extremely high frequency radiation on reproductive function in wistar rats." Bulletin Of Experimental Biology and Medicine 142(2): 189-190.	Frequency not relevant, electric current application, co-exposure or exposure condition unclear
Suh, D. H., et al. (2017). "Clinical efficacy and safety evaluation of a novel fractional unipolar radiofrequency device on facial tightening: A preliminary report." Journal of Cosmetic Dermatology 16(2): 199-204.	Frequency not relevant, electric current application, co-exposure or exposure condition unclear
Sultangaliyeva, I., et al. (2020). "The influence of electromagnetic radiation of cell phones on the behavior of animals." Veterinary World 13(3): 549-555.	Frequency not relevant, electric current application, co-exposure or exposure condition unclear
Swicord, M. L. And J. J. Morrissey (1999). In vivo laboratory experiments related to cellular telephone communications: 111-121.	Type of study not relevant or study not EMF/health-related
Szabo, I., et al. (2004). "Destruction of cutaneous melanoma with millimeter wave hyperthermia in mice." IEEE Transactions on Plasma Science 32(4): 1653-1660.	Frequency not relevant, electric current application, co-exposure or exposure condition unclear
Szabo, L. D., et al. (1986). Biological effects of electromagnetic fields II. Biochemical, embryological and electrophysiological effects of microwaves, Elsevier: 181-184.	Article not peer-reviewed
Szabo, L. D., et al. (1992). Electrophysiological Changes in Rats After Modulated Microwave Irradiation, International Radiation Protection Association: 1140-1143.	Article not peer-reviewed
Szmigielski, S. (1996). "Cancer morbidity in subjects occupationally exposed to high frequency (radiofrequency and microwave) electromagnetic radiation." Science of the Total Environment 180(1): 9-17.	Frequency not relevant, electric current application, co-exposure or exposure condition unclear

Taheri, M., et al. (2017). "The effect of Base Transceiver Station waves on some immunological and hematological factors in exposed persons." Human Antibodies 25(1-2): 31-37.	Frequency not relevant, electric current application, co-exposure or exposure condition unclear
Takahashi, K. A., et al. (2009). "Hyperthermia for the treatment of articular cartilage with osteoarthritis." International Journal of Hyperthermia 25(8): 661-667.	Type of study not relevant or study not EMF/health-related
Takahashi, T., et al. (2003). "Extended two-year results of radio-frequency energy delivery for the treatment of fecal incontinence (the Secca procedure)." Diseases of the Colon and Rectum 46(6): 711-715.	Frequency not relevant, electric current application, co-exposure or exposure condition unclear
Takahashi-Monroy, T., et al. (2008). "SECCA procedure for the treatment of fecal incontinence: results of five-year follow-up." Diseases of the Colon and Rectum 51(3): 355-359.	Frequency not relevant, electric current application, co-exposure or exposure condition unclear
Tan, Y., et al. (2023). "The application of microwaves in axillary hyperhidrosis: Curative effect observation of a pathological examination over 1 year." Journal of Cosmetic Dermatology.	No control group/condition
Tassone, E. E., J. C. Page and M. J. Slepian (2023). "Assessing the Effects of Pulsed Electromagnetic Therapy on Painful Diabetic Distal Symmetric Peripheral Neuropathy: A Double-Blind Randomized Controlled Trial." Journal of Diabetes Science and Technology: 19322968231190413.	Frequency not relevant, electric current application, co-exposure or exposure condition unclear
Taverner, M. G., T. L. Ward and T. E. Loughnan (2010). "Transcutaneous pulsed radiofrequency treatment in patients with painful knee awaiting total knee joint replacement." The Clinical Journal of Pain 26(5): 429-432.	Frequency not relevant, electric current application, co-exposure or exposure condition unclear
Taylor, E. M. And B. T. Ashleman (1974). "Analysis of central nervous system involvement in the microwave auditory effect." Brain Research 74(2): 201-208.	Frequency not relevant, electric current application, co-exposure or exposure condition unclear
Taylor, E. M. And B. T. Ashleman (1975). "Some effects of electromagnetic radiation on the brain and spinal cord of cats." Annals of The New York Academy of Sciences 247: 63-73.	Frequency not relevant, electric current application, co-exposure or exposure condition unclear
Taylor, E. M., et al. (2015). "Pulsed electromagnetic fields dosing impacts postoperative pain in breast reduction patients." Journal of Surgical Research 193(1): 504-510.	Frequency not relevant, electric current application, co-exposure or exposure condition unclear

Teng, J., et al. (1991). “Variations of pain threshold and norepinephrine release in rabbits due to microwave stimulation.” 1991 IEEE MTT-S International Microwave Symposium Digest, Boston, MA, USA. Band 2; IEEE, 1991: 801-804	Frequency not relevant, electric current application, co-exposure or exposure condition unclear
Teng, J., et al. (1995). “Variations of the somatosensory evoked potentials due to microwave fields in rabbits' spinal cord.” Proceedings of 1995 IEEE MTT-S International Microwave Symposium, Orlando, FL, USA. Band 1; IEEE, 1995: 295-298	Frequency not relevant, electric current application, co-exposure or exposure condition unclear
Thamilselvan, S., et al. (2021). "Micronuclei analysis in people residing within 25 m of radiation-exposed areas around mobile towers in Chennai, India: An observational study." Journal of International Oral Health 13(4): 350-355.	Frequency not relevant, electric current application, co-exposure or exposure condition unclear
Thomas, J. R., L. S. Burch and S. S. Yeandle (1979). "Microwave radiation and chlordiazepoxide: synergistic effects on fixed-interval behavior." Science 203(4387): 1357-1358.	Frequency not relevant, electric current application, co-exposure or exposure condition unclear
Thomas, S., et al. (2008). "Exposure to mobile telecommunication networks assessed using personal dosimetry and well-being in children and adolescents: the German mobilee-study." Environ Health 7: 54.	Type of study not relevant or study not EMF/health-related
Thomas, T. L., et al. (1987). "Brain tumor mortality risk among men with electrical and electronics jobs: a case-control study." Journal of the National Cancer Institute 79(2): 233-238.	Frequency not relevant, electric current application, co-exposure or exposure condition unclear
Thomson, R. A. E., S. M. Michaelson and J. W. Howland (1960). "Leukocyte Changes in Normal Dogs Exposed to Microwaves." Federation Proceedings 19(1): 71-71.	Type of study not relevant or study not EMF/health-related
Thomson, R. A., S. M. Michaelson and J. W. Howland (1965). "Modification Of X-Irradiation Lethality in Mice by Microwaves (Radar)." Radiat Res 24: 631-635.	Frequency not relevant, electric current application, co-exposure or exposure condition unclear
Thomson, R. A., S. M. Michaelson and J. W. Howland (1966). "Leukocyte response following simultaneous ionizing and microwave (radar) irradiation." Blood 28(2): 157-162.	Frequency not relevant, electric current application, co-exposure or exposure condition unclear

Thuroczy, G., et al. (1993). “Correlation Between Cerebral-Circulation and Electrical-Activity (EEG) in the Regulatory Physiological Mechanism of the Central-Nervous-System after Microwave Exposure of Rats.” Electricity and Magnetism in Biology and Medicine: Review and Research Papers presented at the First World Congress for Electricity and Magnetism in Biology and Medicine, Orlando, Florida, 1992. San Francisco Press, 1993; 743-746	Frequency not relevant, electric current application, co-exposure or exposure condition unclear
Tian, Y., et al. (2019). "The relationship between microwave radiation injury and abnormal lipid metabolism." Chemistry and Physics of Lipids 225: 104802.	Frequency not relevant, electric current application, co-exposure or exposure condition unclear
Tikhonchuk, V. S. (1978). "Cumulative effect in microwave irradiation." Biol Bull Acad Sci USSR 5(3): 351-353.	Frequency not relevant, electric current application, co-exposure or exposure condition unclear
Tohidi, F. Z., et al. (2021). "Long-term exposure to electromagnetic radiation from mobile phones can cause considerable changes in the balance of Bax/Bcl2 mrna expression in the hippocampus of mice." Electromagnetic Biology and Medicine 40(1): 131-137.	Frequency not relevant, electric current application, co-exposure or exposure condition unclear
Traini, E., et al. (2023). "Time course of health complaints attributed to RF-EMF exposure and predictors of electromagnetic hypersensitivity over 10 years in a prospective cohort of Dutch adults." Science of the Total Environment 856: 159240.	Frequency not relevant, electric current application, co-exposure or exposure condition unclear
Trelles, M. A., et al. (2010). "Histological findings in adipocytes when cellulite is treated with a variable-emission radiofrequency system." Lasers in Medical Science 25(2): 191-195.	Frequency not relevant, electric current application, co-exposure or exposure condition unclear
Tripathi, R., et al. (2022). "Simultaneous exposure to electromagnetic field from mobile phone and unimpeded fructose drinking during pre-, peri-, and post-pubertal stages perturbs the hypothalamic and hepatic regulation of energy homeostasis by early adulthood: experimental evidence." Environmental Science and Pollution Research 29(5): 7438-7451.	Frequency not relevant, electric current application, co-exposure or exposure condition unclear
Trofè, A., et al. (2023). "Effect of Pulsed Electromagnetic Fields (pemfs) on Muscular Activation during Cycling: A Single-Blind Controlled Pilot Study." Healthcare 11(6): 922.	Frequency not relevant, electric current application, co-exposure or exposure condition unclear

Tsai, T. H., et al. (2012). "Visualizing radiofrequency-skin interaction using multiphoton microscopy in vivo." J Dermatol Sci 65(2): 95-101.	Frequency not relevant, electric current application, co-exposure or exposure condition unclear
Tuschl, H., et al. (1999). "Occupational exposure to high frequency electromagnetic fields and its effect on human immune parameters." International Journal of Occupational Medicine and Environmental Health 12(3): 239-251.	Frequency not relevant, electric current application, co-exposure or exposure condition unclear
Tuteja, D., et al. (2021). "Effect of mobile phone usage on cognitive functions, sleep pattern, visuospatial ability in Parkinsons patients	Frequency not relevant, electric current application, co-exposure or exposure condition unclear
Tyazhelov, V. V., et al. (1979). "Some peculiarities of auditory sensations evoked by pulsed microwave fields." Radio Science 14: 259-263.	Frequency not relevant, electric current application, co-exposure or exposure condition unclear
Tynes, T., A. Andersen and F. Langmark (1992). "Incidence of cancer in Norwegian workers potentially exposed to electromagnetic fields." American Journal of Epidemiology 136(1): 81-88.	Frequency not relevant, electric current application, co-exposure or exposure condition unclear
Urquhart, J. D., et al. (1991). "Case-control study of leukaemia and non-Hodgkin's lymphoma in children in Caithness near the Dounreay nuclear installation." BMJ 302(6778): 687-692.	Frequency not relevant, electric current application, co-exposure or exposure condition unclear
Usman, J. D., et al. (2020). "Assessment of electromagnetic fields, vibration and sound exposure effects from multiple transceiver mobile phones on oxidative stress levels in serum, brain and heart tissue." Scientific African 7: e00271.	Frequency not relevant, electric current application, co-exposure or exposure condition unclear
Valtonen, E. J. (1966). "The effects of microwave radiation on the cellular elements in the peritoneal fluid and peripheral blood of the rat." Acta Rheumatol Scand 12(4): 291-299.	Frequency not relevant, electric current application, co-exposure or exposure condition unclear
Van Boxem, K., et al. (2011). "Pulsed Radiofrequency Treatment Adjacent to the Lumbar Dorsal Root Ganglion for the Management of Lumbosacral Radicular Syndrome: A Clinical Audit." Pain Medicine 12(9): 1322-1330.	Frequency not relevant, electric current application, co-exposure or exposure condition unclear
Van Moorselaar, I., et al. (2017). "Effects of personalised exposure on self-rated electromagnetic hypersensitivity and sensibility - A double-blind randomised controlled trial." Environment International 99: 255-262.	Frequency not relevant, electric current application, co-exposure or exposure condition unclear

Van Zundert, J., et al. (2002). "Application of radiofrequency treatment in practical pain management: state of the art." Pain Practice 2(3): 269-278.	Type of study not relevant or study not EMF/health-related
Van Zundert, J., et al. (2003). "Percutaneous pulsed radiofrequency treatment of the cervical dorsal root ganglion in the treatment of chronic cervical pain syndromes: a clinical audit." Neuromodulation 6(1): 6-14.	Frequency not relevant, electric current application, co-exposure or exposure condition unclear
Van Zundert, J., et al. (2007). "Pulsed radiofrequency adjacent to the cervical dorsal root ganglion in chronic cervical radicular pain: a double blind sham controlled randomized clinical trial." Pain 127(1-2): 173-182.	Frequency not relevant, electric current application, co-exposure or exposure condition unclear
Vander Vorst, A. And D. Adang (2005). "Epidemiology study on rats exposed to microwaves." IEEE Microwave Magazine 6(4): 112-113.	Type of study not relevant or study not EMF/health-related
Vangelova, K., et al. (2007). "Changes in excretion rates of stress hormones in medical staff exposed to electromagnetic radiation." The Environmentalist 27(4): 551-555.	Frequency not relevant, electric current application, co-exposure or exposure condition unclear
Vangelova, K., M. Israel and S. Mihaylov (2002). "The effect of low level radiofrequency electromagnetic radiation on the excretion rates of stress hormones in operators during 24-hour shifts." Central European Journal of Public Health 10(1-2): 24-28.	Frequency not relevant, electric current application, co-exposure or exposure condition unclear
Varcaccio-Garofalo, G., et al. (1995). "Analgesic properties of electromagnetic field therapy in patients with chronic pelvic pain." Clinical and Experimental Obstetrics and Gynecology 22(4): 350-354.	Frequency not relevant, electric current application, co-exposure or exposure condition unclear
Varma, M. M. And E. A. Traboulay (1977). "Comparison of native and microwave irradiated DNA." Experientia 33(12): 1649-1650.	Frequency not relevant, electric current application, co-exposure or exposure condition unclear
Vaupel, P. W., J. Otte and R. Manz (1982). "Oxygenation of malignant tumors after localized microwave hyperthermia." Radiation And Environmental Biophysics 20(4): 289-300.	Frequency not relevant, electric current application, co-exposure or exposure condition unclear
Vecsei, Z., et al. (2018). "Short-term radiofrequency exposure from new generation mobile phones reduces EEG alpha power with no effects on cognitive performance." Scientific Reports 8: 18010.	Frequency not relevant, electric current application, co-exposure or exposure condition unclear

Vecsei, Z., G. Thuróczy and I. Hernádi (2018). "The Effect of a Single 30-Min Long Term Evolution Mobile Phone-Like Exposure on Thermal Pain Threshold of Young Healthy Volunteers." International Journal of Environmental Research and Public Health 15(9).	Frequency not relevant, electric current application, co-exposure or exposure condition unclear
Verma, S., et al. (2018). X-Band Microwave Radiation Induced Biological Effects in Rats Skin: Plausible Role of Heat Shock Proteins, IEEE: 175-177.	Article not peer-reviewed
Vignati, M. And L. Giuliani (1997). "Radiofrequency exposure near high-voltage lines." Environmental Health Perspectives 105: 1569-1573.	Type of study not relevant or study not EMF/health-related
Vijayalaxmi, et al. (1999). "Frequency of micronuclei in the blood and bone marrow cells of mice exposed to ultra-wideband electromagnetic radiation." International Journal of Radiation Biology 75(1): 115-120.	Frequency not relevant, electric current application, co-exposure or exposure condition unclear
Vila, J., et al. (2018). "Occupational exposure to high-frequency electromagnetic fields and brain tumor risk in the INTEROCC study: An individualized assessment approach." Environment International 119: 353-365.	Frequency not relevant, electric current application, co-exposure or exposure condition unclear
Virkkunen, P. (1982). "Plasma And Red-Cell Volumes of Microwave Irradiated Mice-Tissues." Acta Physiologica Scandinavica 115(2): 161-164.	Frequency not relevant, electric current application, co-exposure or exposure condition unclear
Vitulli, W. F., et al. (1986). "Behavioral thermoregulation with microwave radiation of albino rats." Perceptual and Motor Skills 62(3): 831-840.	Frequency not relevant, electric current application, co-exposure or exposure condition unclear
Vitulli, W. F., et al. (1987). "Behavioral effects of microwave reinforcement schedules and variations in microwave intensity on albino rats." Perceptual and Motor Skills 65(3): 787-795.	Frequency not relevant, electric current application, co-exposure or exposure condition unclear
Vitulli, W. F., et al. (1992). "Alcohol Effects on Behavioral Thermoregulation With Microwave-Radiation." Psychological Reports 70(3): 1160-1162.	Frequency not relevant, electric current application, co-exposure or exposure condition unclear
Vitulli, W. F., et al. (1993). "Aspirin (acetylsalicylic acid) effects on behavioral thermoregulation with microwave radiation." Percept Mot Skills 77(1): 187-191.	Frequency not relevant, electric current application, co-exposure or exposure condition unclear

Von Klitzing, L. (1995). "Low-Frequency pulsed electromagnetic fields influence EEG of man." Physica Medica: European Journal of Medical Physics XI(2): 77-80.	Frequency not relevant, electric current application, co-exposure or exposure condition unclear
Wang, B., et al. (2022). "Efficacy and safety of non-surgical short-wave radiofrequency treatment of mild-to-moderate erythematotelangiectatic rosacea: a prospective, open-label pilot study." Archives of Dermatological Research 314(4): 341-347.	Frequency not relevant, electric current application, co-exposure or exposure condition unclear
Wang, C. Z., et al. (2015). "Pulsed Microwave Exposure Real Time and Dynamically Reduces Reactive Oxygen Species In Rat Pheochromocytoma Cells In Vivo." Microwave And Optical Technology Letters 57(10): 2395-2400.	Non-mammal/in vitro study
Wang, H., et al. (2018). "Protective Role of NMDAR for Microwave-Induced Synaptic Plasticity Injuries in Primary Hippocampal Neurons." Cellular Physiology and Biochemistry 51(1): 97-112.	Non-mammal/in vitro study
Wang, L. F., et al. (2012). "Application of H-1-NMR-based metabolomics for detecting injury induced by long-term microwave exposure in Wistar rats' urine." Analytical And Bioanalytical Chemistry 404(1): 69-78.	Frequency not relevant, electric current application, co-exposure or exposure condition unclear
Wang, L. F., et al. (2015). "Microwave-Induced Structural and Functional Injury of Hippocampal and PC12 Cells Is Accompanied by Abnormal Changes in the NMDAR-PSD95-camkii Pathway." Pathobiology 82(5): 181-194.	Frequency not relevant, electric current application, co-exposure or exposure condition unclear
Wang, L. F., et al. (2016). "Identification of a Novel Rat NR2B Subunit Gene Promoter Region Variant and Its Association with Microwave-Induced Neuron Impairment." Molecular Neurobiology 53(4): 2100-2111.	Frequency not relevant, electric current application, co-exposure or exposure condition unclear
Wang, L., et al. (2009). "Abnormality of synaptic vesicular associated proteins in cerebral cortex and hippocampus after microwave exposure." Synapse 63(11): 1010-1016.	Frequency not relevant, electric current application, co-exposure or exposure condition unclear
Wang, N. And H. Li (2023). "Therapeutic effect of the temperature-controlled radio frequency technology in female sexual dysfunction." Ginekologia Polska.	Frequency not relevant, electric current application, co-exposure or exposure condition unclear

Wang, Y., et al. (2023). "Inhibition of ferroptosis alleviates high-power microwave-induced myocardial injury." Frontiers in Cardiovascular Medicine 10: 1157752.	Frequency not relevant, electric current application, co-exposure or exposure condition unclear
Wardzinski, E. K., et al. (2022). "Mobile Phone Radiation Deflects Brain Energy Homeostasis and Prompts Human Food Ingestion." Nutrients 14(2): 339.	Frequency not relevant, electric current application, co-exposure or exposure condition unclear
Way, W. I., H. Kritikos and H. Schwan (1981). "Thermoregulatory physiologic responses in the human body exposed to microwave radiation." Bioelectromagnetics 2(4): 341-356.	Type of study not relevant or study not EMF/health-related
Wei, X., et al. (2019). "Histone acetyltransferase TIP60 mediates spermatozoa damage induced by microwave irradiation in rats." International Journal Of Clinical And Experimental Medicine 12(7): 8032-8043.	Frequency not relevant, electric current application, co-exposure or exposure condition unclear
Wei, Y., et al. (2019). "Modulation of resting-state brain functional connectivity by exposure to acute fourth-generation long-term evolution electromagnetic field: An fmri study." Bioelectromagnetics 40(1): 42-51.	Frequency not relevant, electric current application, co-exposure or exposure condition unclear
Wenzel, C., et al. (2002). "ACTH-Stimulationstest bei Kühen unter dem Einfluss hochfrequenter elektromagnetischer Felder." Tierarztliche Praxis Ausgabe G, Grosstiere/Nutztiere 30: 144-147.	Frequency not relevant, electric current application, co-exposure or exposure condition unclear
Weyandt, T. B., et al. (1996). "Semen analysis of military personnel associated with military duty assignments." Reproductive Toxicology 10(6): 521-528.	Frequency not relevant, electric current application, co-exposure or exposure condition unclear
Whungtrakulchai, T., W. Charoenwat and P. Sittiprapaporn (2017). “Wearable lightweight electroencephalographic study on dialing 4G mobile phone by LINE application.” 2017 14th International Conference on Electrical Engineering/Electronics, Computer, Telecommunications and Information Technology (ECTI-CON), Phuket, Thailand. IEEE, 2017	Frequency not relevant, electric current application, co-exposure or exposure condition unclear
Wilén, J., et al. (2004). "Electromagnetic field exposure and health among RF plastic sealer operators." Bioelectromagnetics 25(1): 5-15.	Frequency not relevant, electric current application, co-exposure or exposure condition unclear
Wilen, J., et al. (2007). "Changes in heart rate variability among RF plastic sealer operators." Bioelectromagnetics 28(1): 76-79.	Frequency not relevant, electric current application, co-exposure or exposure condition unclear

Wilkins 3rd, J. R. And V. D. Hundley (1990). "Paternal occupational exposure to electromagnetic fields and neuroblastoma in offspring." American Journal of Epidemiology 131(6): 995-1008.	Frequency not relevant, electric current application, co-exposure or exposure condition unclear
Willett, E. V., et al. (2003). "Occupational exposure to electromagnetic fields and acute leukaemia: analysis of a case-control study." Occupational and Environmental Medicine 60(8): 577-583.	Frequency not relevant, electric current application, co-exposure or exposure condition unclear
Williams, C., et al. (2000). A new method for quantitative analysis of magnetic resonance images of in vivo prostate glands following transurethral microwave radiation therapy for benign prostatic hyperplasia. 1214: 619-624.	Frequency not relevant, electric current application, co-exposure or exposure condition unclear
Wilson, B. S., et al. (1980). "Alterations in activity at auditory nuclei of the rat induced by exposure to microwave radiation: autoradiographic evidence using [14C]2-deoxy-D-glucose." Brain Research 187(2): 291-306.	Frequency not relevant, electric current application, co-exposure or exposure condition unclear
Wiwanitkit, V. (2013). "Radiofrequency radiation and human ferritin." J Med Signals Sens 3(1): 61.	Type of study not relevant or study not EMF/health-related
Wu, B., et al. (2012). "Changes in spinal cord met-enkephalin levels and mechanical threshold values of pain after pulsed radio frequency in a spared nerve injury rat model." Neurological Research 34(4): 408-414.	Frequency not relevant, electric current application, co-exposure or exposure condition unclear
Wu, H., et al. (2018). "Activation of TLR signalling regulates microwave radiation-mediated impairment of spermatogenesis in rat testis." Andrologia 50(1).	Frequency not relevant, electric current application, co-exposure or exposure condition unclear
Wu, H., et al. (2023). "Effect of wifi signal exposure in utero and early life on neurodevelopment and behaviors of rats." Environmental Science and Pollution Research.	Frequency not relevant, electric current application, co-exposure or exposure condition unclear
Xenos, T. D. And I. N. Magras (2003). "Low power density RF-radiation effects on experimental animal embryos and foetuses." Biological Effects of Electromagnetic Radiation. Springer, Berlin, Heidelberg, 2003; 579-602	Article not peer-reviewed
Xia, Z., et al. (2021). "Lipidomics of Serum and Hippocampus Reveal the Protective Effects of Fermented Soybean Lipid on Rats of Microwave-Induced Cognitive Damage." ACS Chemical Neuroscience 12(12): 2122-2132.	Frequency not relevant, electric current application, co-exposure or exposure condition unclear

Xiong, L., et al. (2015). "Microwave exposure impairs synaptic plasticity in the rat hippocampus and PC12 cells through over-activation of the NMDA receptor signaling pathway." Biomedical and Environmental Sciences 28(1): 13-24.	Frequency not relevant, electric current application, co-exposure or exposure condition unclear
Xu, Z. W., et al. (2007). "Theophylline attenuates microwave-induced impairment of memory acquisition." Neurosci Lett 412(2): 129-133.	Frequency not relevant, electric current application, co-exposure or exposure condition unclear
Yamazaki, K., et al. (2022). "Measurement of personal radio frequency exposure in Japan: The Hokkaido Study on the Environment and Children's health." Environ Res 216(Pt 1): 114429.	Frequency not relevant, electric current application, co-exposure or exposure condition unclear
Yamazaki, K., et al. (2023). "Measurement of personal radio frequency exposure in Japan: The Hokkaido Study on the Environment and Children's health." Environmental Research 216: 114429.	Frequency not relevant, electric current application, co-exposure or exposure condition unclear
Yang, H. K., et al. (1983). "Effects of microwave exposure on the hamster immune system. I. Natural killer cell activity." Bioelectromagnetics 4(2): 123-139.	Frequency not relevant, electric current application, co-exposure or exposure condition unclear
Yang, L., et al. (2017). "Long-Term Evolution electromagnetic fields exposure modulates the resting state EEG on alpha and beta bands." Clinical EEG and Neuroscience 48(3): 168-175.	Frequency not relevant, electric current application, co-exposure or exposure condition unclear
Yang, L., et al. (2021). "Functional and network analyses of human exposure to long-term evolution signal." Environmental Science and Pollution Research 28(5): 5755-5773.	Frequency not relevant, electric current application, co-exposure or exposure condition unclear
Yang, L., et al. (2021). "No Alteration Between Intrinsic Connectivity Networks by a Pilot Study on Localized Exposure to the Fourth-Generation Wireless Communication Signals." Front Public Health 9: 734370.	Frequency not relevant, electric current application, co-exposure or exposure condition unclear
Yang, L., et al. (2022). "No Alteration Between Intrinsic Connectivity Networks by a Pilot Study on Localized Exposure to the Fourth-Generation Wireless Communication Signals." Frontiers in Public Health 9: 734370.	Frequency not relevant, electric current application, co-exposure or exposure condition unclear
Yao, K. T. (1978). "Microwave radiation-induced chromosomal aberrations in corneal epithelium of Chinese hamsters." The Journal of Heredity 69(6): 409-412.	Frequency not relevant, electric current application, co-exposure or exposure condition unclear

Yao, K. T. S. (1978). "Microwave Radiation-Induced Chromosomal-Aberrations in Corneal Epithelium Of Chinese Hamsters." Journal Of Heredity 69(6): 409-412.	Frequency not relevant, electric current application, co-exposure or exposure condition unclear
Yavaş, M. C. (2020). "The effect of short-term electromagnetic fields caused by mobile phones on the electrical activity of alpha and beta brain waves." Annals of Clinical and Analytical Medicine 11(5): 474-478.	Frequency not relevant, electric current application, co-exposure or exposure condition unclear
Yazdanpanahi, M., et al. (2023). "Evaluating the Effect of Jammer Radiation on Learning and Memory in Male Rats." Journal of Biomedical Physics & Engineering 13(1): 29-38.	Frequency not relevant, electric current application, co-exposure or exposure condition unclear
Ye, J., et al. (2001). "Low power density microwave radiation induced early changes in rabbit lens epithelial cells." Chinese Medical Journal 114(12): 1290-1294.	Frequency not relevant, electric current application, co-exposure or exposure condition unclear
Ye, J., et al. (2002). "Changes in gap junctional intercellular communication in rabbits lens epithelial cells induced by low power density microwave radiation." Chinese Medical Journal 115(12): 1873-1876.	Frequency not relevant, electric current application, co-exposure or exposure condition unclear
Yeargers, E. K., et al. (1975). "Effects of microwave radiation of enzymes." Annals Of The New York Academy of Sciences 247: 301-304.	Frequency not relevant, electric current application, co-exposure or exposure condition unclear
Yeh, C. C., et al. (2015). "Long-Term Anti-Allodynic Effect of Immediate Pulsed Radiofrequency Modulation through Down-Regulation of Insulin-Like Growth Factor 2 in a Neuropathic Pain Model." Int J Mol Sci 16(11): 27156-27170.	Frequency not relevant, electric current application, co-exposure or exposure condition unclear
Yerushalmi, A., et al. (1984). "Systemic alteration in blood glucose levels following localized deep microwave hyperthermia." The Journal of Microwave Power 19(1): 73-76.	Frequency not relevant, electric current application, co-exposure or exposure condition unclear
Yerushalmi, A., et al. (1985). "Localized deep microwave hyperthermia in the treatment of poor operative risk patients with benign prostatic hyperplasia." The Journal of Urology 133(5): 873-876.	Frequency not relevant, electric current application, co-exposure or exposure condition unclear
Yildirim, M. E., et al. (2015). "What is harmful for male fertility: Cell phone or the wireless internet?" The Kaohsiung Journal of Medical Sciences 31(9): 480-484.	Frequency not relevant, electric current application, co-exposure or exposure condition unclear

Yinhui, P., et al. (2019). "Effect of cell phone radiation on neutrophil of mice." International Journal of Radiation Biology 95(8): 1178-1184.	Frequency not relevant, electric current application, co-exposure or exposure condition unclear
Yoon, H., et al. (2012). Microwave Thermal Radiation Effects on Skin Tissues. 8548	Non-mammal/in vitro study
Yu, G., et al. (2020). "Long-term exposure to 4G smartphone radiofrequency electromagnetic radiation diminished male reproductive potential by directly disrupting Spock3-MMP2-BTB axis in the testes of adult rats." Science of the Total Environment 698: 133860.	Frequency not relevant, electric current application, co-exposure or exposure condition unclear
Zarei, S., et al. (2015). "A Challenging Issue in the Etiology of Speech Problems: The Effect of Maternal Exposure to Electromagnetic Fields on Speech Problems in the Offspring." Journal of Biomedical Physics & Engineering 5(3): 151-154.	Frequency not relevant, electric current application, co-exposure or exposure condition unclear
Zarei, S., et al. (2019). "Mother's Exposure to Electromagnetic Fields before and during Pregnancy is Associated with Risk of Speech Problems in Offspring." Journal of Biomedical Physics & Engineering 9(1): 61-68.	Frequency not relevant, electric current application, co-exposure or exposure condition unclear
Zaret, M. M. And M. Eisenbud (1961). Preliminary results of studies of the lenticular effects of microwaves among exposed personnel, Plenum Press: 293–308.	Frequency not relevant, electric current application, co-exposure or exposure condition unclear
Zeng, L., et al. (2011). "mnsod expression inhibited by electromagnetic pulse radiation in the rat testis." Electromagnetic Biology and Medicine 30(4): 205-218.	Frequency not relevant, electric current application, co-exposure or exposure condition unclear
Zerbinati, N., et al. (2019). "New System Delivering Microwaves Energy for Inducing Subcutaneous Fat Reduction: In - Vivo Histological and Ultrastructural Evidence." Open Access Maced J Med Sci 7(18): 2991-2997.	Retracted article
Zhang, F., et al. (2014). "Intrabiliary RF heat-enhanced local chemotherapy of a cholangiocarcinoma cell line: monitoring with dual-modality imaging - preclinical study." Radiology 270(2): 400-408.	Frequency not relevant, electric current application, co-exposure or exposure condition unclear
Zhang, K. D., et al. (2017). "Apoptosis of Lewis Lung Carcinoma Cells Induced by Microwave via p53 and Proapoptotic Proteins In vivo." Chinese Medical Journal 130(1): 15-22.	Frequency not relevant, electric current application, co-exposure or exposure condition unclear

Zhang, S., et al. (2022). "Effects of mobile phone use on semen parameters: a cross-sectional study of 1634 men in China." Reproduction, Fertility, and Development 34(9): 669-678.	Frequency not relevant, electric current application, co-exposure or exposure condition unclear
Zhang, X., et al. (2014). "The compound Chinese medicine "Kang Fu Ling" protects against high power microwave-induced myocardial injury." Plos One 9(7): e101532.	Frequency not relevant, electric current application, co-exposure or exposure condition unclear
Zhang, Y., et al. (2019). "Effect and Safety Evaluation of XETHRU X4 Radar Radiation on Sexual Hormone Levels in Mice." Annu Int Conf IEEE Eng Med Biol Soc 2019: 1318-1320.	Frequency not relevant, electric current application, co-exposure or exposure condition unclear
Zhang, Y., et al. (2022). "Cinnarizine dissolving microneedles against microwave-induced brain injury." Biomed Pharmacother 155: 113779.	Frequency not relevant, electric current application, co-exposure or exposure condition unclear
Zhang, Y., et al. (2023). "Mobile Phone Use, Genetic Susceptibility and New-Onset Chronic Kidney Diseases." International Journal of Public Health 68: 1605358.	Frequency not relevant, electric current application, co-exposure or exposure condition unclear
Zhao, D., et al. (2021). "Risk of congenital heart disease due to exposure to common electrical appliances during early pregnancy: a case-control study." Environmental Science and Pollution Research 28(4): 4739-4748.	Frequency not relevant, electric current application, co-exposure or exposure condition unclear
Zhao, L., et al. (2012). "Relationship between Cognition Function and Hippocampus Structure after Long-term Microwave Exposure." Biomedical and Environmental Sciences 25(2): 182-188.	Frequency not relevant, electric current application, co-exposure or exposure condition unclear
Zhao, L., et al. (2014). "micrornas: novel mechanism involved in the pathogenesis of microwave exposure on rats' hippocampus." Journal of Molecular Neuroscience 53(2): 222-230.	Frequency not relevant, electric current application, co-exposure or exposure condition unclear
Zhao, L., et al. (2020). "Inhibition of microrna 219 Expression Protects Synaptic Plasticity via Activating NMDAR1, camkiiγ, and p-CREB after Microwave Radiation." Biomedical and Environmental Sciences 33(5): 359-364.	Frequency not relevant, electric current application, co-exposure or exposure condition unclear
Zhao, L., et al. (2022). "The underlying molecular mechanism of intratumoral radiofrequency hyperthermia-enhanced chemotherapy of pancreatic cancer." Journal of Interventional Medicine 5(2): 57-63.	Frequency not relevant, electric current application, co-exposure or exposure condition unclear

Zhi, W. J., et al. (2018). "Microwave radiation leading to shrinkage of dendritic spines in hippocampal neurons mediated by SNK-SPAR pathway." Brain Research 1679: 134-143.	Frequency not relevant, electric current application, co-exposure or exposure condition unclear
Zhi, W. J., et al. (2022). "Low p-SYN1 (Ser-553) Expression Leads to Abnormal Neurotransmitter Release of GABA Induced by Up-Regulated Cdk5 after Microwave Exposure: Insights on Protection and Treatment of Microwave-Induced Cognitive Dysfunction." Current Issues in Molecular Biology 44(1): 206-221.	Frequency not relevant, electric current application, co-exposure or exposure condition unclear
Zhou, H., et al. (2023). "Copper-cysteamine nanoparticle-mediated microwave dynamic therapy improves cancer treatment with induction of ferroptosis." Bioactive Materials 24: 322-330.	Frequency not relevant, electric current application, co-exposure or exposure condition unclear
Zhu, R. Q., et al. (2022). "Transcriptome Sequencing of mrna and lncrna in Hippocampal Tissues of Rats under Microwave Exposure." Biomedical and Environmental Sciences 35(11): 1079-1084.	Frequency not relevant, electric current application, co-exposure or exposure condition unclear
Zhu, S., et al. (2020). "The toxic effect of mobile phone radiation on rabbit organs." All Life 13(1): 252-258.	Frequency not relevant, electric current application, co-exposure or exposure condition unclear
Zhukova, G. V., et al. (2017). "The pattern of the immune Processes during activation therapy with the use of microwave electromagnetic radiation." Cardiometry(11): 35-45.	Frequency not relevant, electric current application, co-exposure or exposure condition unclear
Zilov, V. G., et al. (2018). "Effects of Electromagnetic Fields Modulated by Infralow Frequencies on the Production of Stem Cells." Bulletin Of Experimental Biology and Medicine 164(5): 685-687.	Frequency not relevant, electric current application, co-exposure or exposure condition unclear
Zippo, A. G., et al. (2015). "Electrophysiological effects of non-invasive Radio Electric Asymmetric Conveyor (REAC) on thalamocortical neural activities and perturbed experimental conditions." Scientific Reports 5: 18200.	Frequency not relevant, electric current application, co-exposure or exposure condition unclear
Zook, B. C. And S. J. Simmens (2005). "Neurogenic tumors in rats induced by ethylnitrosourea." Exp Toxicol Pathol 57(1): 7-14.	Frequency not relevant, electric current application, co-exposure or exposure condition unclear

Zou, Y., et al. (2018). "Comparative study of the proliferative ability of skeletal muscle satellite cells under microwave irradiation in fractures with titanium alloy internal fixation in rabbits." Experimental and Therapeutic Medicine 16(6): 4357-4366.	Frequency not relevant, electric current application, co-exposure or exposure condition unclear
Zufry, H., et al. (2023). "Effects of mobile phone electromagnetic radiation on thyroid glands and hormones in Rattus norvegicus brain: An analysis of thyroid function, reactive oxygen species, and monocarboxylate transporter 8." Journal of Advanced Pharmaceutical Technology & Research 14(2): 63-68.	Frequency not relevant, electric current application, co-exposure or exposure condition unclear
Zukauskas, G. And K. Dapsys (1991). "Bioelectrical Homeostasis as A Component of Acupuncture Mechanism." Acupuncture & Electro-Therapeutics Research 16(3-4): 117-126.	Frequency not relevant, electric current application, co-exposure or exposure condition unclear
