# Supplementary material for: Physiological and health-related effects of 5G-relevant radiofrequency fields: A systematic review of experimental studies in humans and mammals
Source: PLoS One. 2026 Nov 4;21(11):e0361104. doi: 10.1371/journal.pone.0361104 (PMC13734471; doi:10.1371/journal.pone.0361104)
Supplement: Supplementary file 5 [file pone.0361104.s005.pdf]

S5 Extracted study parameters.

**General information**	
Identifier	Internal number
Citation	Authors, title, journal, year, volume, page numbers, DOI
Funding source	Industry, public, independent (NGO), mixed, unknown
Declaration of Competing Interest	Yes, no, not stated
**Study design**	Experimental animal study (EA), human controlled trial (HCT) or epidemiological study (ES)
**1. Experimental studies (HCT, EA)**	
Study group	Human (healthy, unhealthy) or animal strain/species
Sex	Male, female, not stated
Age	Range, mean, SD, not stated?
**Study size**	
Exposure group(s)	Number and size of groups
Control group(s)	Number and size of groups
Control group setup	Sham-exposed, non-exposed, positive control, cage control, not specified
**2. Epidemiological studies (ES)**	
Epidemiological study type	Prospective or retrospective cohort (Co) and case-control (CaCo) studies
Country	Details
Observation period	Time span in years, follow-up time
Study population	General population, occupational
Definition of study population	Description or name
Age	Range, mean, SD in years
Sex	Male, female, not stated
Number of participants (Co)	Number
Number of cases and controls (CaCo)	Number
Confounder (ES)	Details
**Exposure**	
Exposure source	Details
Frequency range	Numeric value in GHz
Modulation type	Continuous wave (CW), amplitude modulation (AM), frequency modulation (FM), pulsed, etc.; related details (e.g., pulse duration and repetition rate)

Exposure duration	Minutes or hours (per day), days per week and total period, intermittent
**Details of internal exposure metric**	
Specific absorption rate (SAR)	Local or whole-body SAR, numeric value in W/kg
Specific absorption (SA)	Numeric value in J/kg
Induced electric field strength	Numeric value in V/m
Internal magnetic field strength	Numeric value in A/m
**Details of external exposure metric**	Time average, spatial-surface average, or peak value
Incident power flux density	Numeric value in W/m^2
Incident energy density	Numeric value in J/m^2
Transmitted (absorbed) power flux density	Numeric value in W/m^2
Transmitted (absorbed) energy density	Numeric value in J/m^2
**Details of external exposure metric (at the location of the exposed body)**	Time average, spatial-surface average, or peak value
Incident electric field strength	Numeric value in V/m
Incident magnetic field strength	Numeric value in A/m
Exposure metrics in ES	E.g., cumulative or average use, cumulative exposure per lifetime (assessed by questionnaire or JEM)
Exposure validation	Measurement, simulation, questionnaire, JEM
Environmental conditions (e.g., temperature control, background level)	Details
**Outcome**	
Endpoint 1	Details
Assessment method for Endpoint 1	Details
Endpoint 2	Details
Assessment method for Endpoint 2	Details
Endpoint x	Details
Assessment method for Endpoint x	Details
...	
**Results**	
Significant results	Details
Conclusion (according to the authors)	Details
Effect found (according to the authors)	Yes, no
Remarks	Details

Abbreviations: AM – amplitude modulation, CaCo - case-control study, Co - cohort study, CW -continuous wave, DOI - digital object identifier, EA – experimental animal study, ES – epidemiological study, FM - frequency modulation, HCT – human controlled trial, NGO – non-governmental organization, SD – standard deviation
