# Supplementary material for: Physiological and health-related effects of 5G-relevant radiofrequency fields: A systematic review of experimental studies in humans and mammals
Source: PLoS One. 2026 Nov 4;21(11):e0361104. doi: 10.1371/journal.pone.0361104 (PMC13734471; doi:10.1371/journal.pone.0361104)
Supplement: Supplementary file 6 [file pone.0361104.s006.pdf]

S6 Risk-of-Bias. Risk-of-Bias domains and questions according to OHAT.

The OHAT Risk-of-Bias (RoB) Rating Tool for Human and Animal Studies (NTP 2015) will be followed as precisely as possible for every assessed RoB criterion. However, especially with a view on the peculiarities of EMF exposure, some specifications and amendments have to be applied on the tool. In the following, these modifications are presented for each RoB criterion. Background information and further elucidations on each RoB domains can be found in OHAT RoB tool (NTP 2015).

**Selection Bias**
**Question 1: Was administered dose or exposure level adequately randomized?**<br>*We recognize that given reporting practices for experimental animal studies it is unlikely that the allocation method will be explicitly reported in most studies. Thus, in cases where randomization is reported but the method is unknown, we will classify studies as "probably low risk of bias". As there are no approaches to assess cross-over studies mentioned in the OHAT protocol, we followed the recommendations for the Cochrane protocol by Ding et al. 2015[1]. For this criterion, randomization in a cross-over design applies to the order of treatments.*
Experimental animals (EA), Human Controlled Trial (HCT)
Definitely Low Risk of Bias (++)
• direct evidence that subjects or animals were allocated to any study group including controls using a method with a random component<br>**OR**<br>• direct evidence that sequences of exposure were allocated using a method with a random component (in case of crossover studies, according to Cochrane protocol (Ding et al. 2015)[1]
Probably Low Risk of Bias (+)
• indirect evidence that subjects or animals were allocated to any study group, including controls, using a method with a random component<br>**OR**<br>• it is deemed that allocation without a clearly random component during the study would not appreciably bias results<br>**OR**<br>• indirect evidence that sequences of exposure were allocated using a method with a random component (in case of crossover studies)
Probably High Risk of Bias (-)
• indirect evidence that subjects or animals were allocated to study groups using a method with a non-random component<br>**OR**<br>• there is insufficient information provided about how subjects were allocated to study groups (record "NR" as basis for answer)<br>**OR**<br>• indirect evidence that sequences of exposure were allocated using a method with a non-random component (in case of crossover studies
Definitely High Risk of Bias (--)
• direct evidence that subjects were allocated to study groups using a non-random method (e.g., according to investigator's judgment)

[1] Ding H, Hu GL, Zheng XY, Chen Q, Threapleton DE, et al. (2015) The Method Quality of Cross-Over Studies Involved in Cochrane Systematic Reviews. PLOS ONE 10(4): e0120519. https://doi.org/10.1371/journal.pone.0120519

**OR**<br>• direct evidence that sequences of exposure were allocated using a method with a non-random component (in case of crossover studies)
**Question 2: Was allocation to study groups adequately concealed?**<br>*As there are no approaches to assess cross-over studies mentioned in the OHAT protocol, we followed the recommendations for the Cochrane protocol by Ding et al. 2015[1]. Accordingly, for cross-over studies, this criterion assesses the blinding of the determination of the sequence of treatments. In the case of study designs, in which all subjects subsequently perform different (sham-)exposure runs in a randomized order, this criterion does not apply (n.a.).*
EA, HCT
Definitely Low Risk of Bias (++)
• direct evidence that at the time of assigning study groups research personnel (and subjects in HCT) were adequately blinded to study groups and it is unlikely that they could have broken the blinding until after assignment was complete and irrevocable
Probably Low Risk of Bias (+)
• indirect evidence that at the time of assigning study groups research personnel (and subjects in HCT) were adequately blinded to study groups and it is unlikely that they could have broken the blinding until after assignment was complete and irrevocable<br>**OR**<br>• it is deemed that lack of adequate blinding at the time of assigning study groups would not appreciably bias results
Probably High Risk of Bias (-)
• indirect evidence that at the time of recruitment it was possible for the research personnel (and subjects in HCT) to know what study group subjects were allocated to, or it is likely that they could have broken the blinding of allocation before recruitment was complete and irrevocable<br>**OR**<br>• there is insufficient information provided about allocation to study groups (record "NR" as basis for answer)
Definitely High Risk of Bias (--)
• direct evidence that at the time of recruitment it was possible for the research personnel (and subjects in HCT) to know what study group subjects were allocated to

**Performance Bias**
**Question 3: Were experimental conditions identical across study groups?**<br>*In the original OHAT protocol, this criterion only applies to animal studies. However, in human studies with exposure to EMF, systematic differences between the experimental conditions during exposure versus sham exposure could substantially bias the outcomes. Therefore, we deemed this criterion crucial for a well-controlled design in human studies. For HCT investigating EMF, we specifically assessed the presence and quality of sham exposure in the control group(s) or conditions in this criterion.*
EA, HCT
Definitely Low Risk of Bias (++)
• direct evidence that exposure-associated side effects and any other conditions were identical across study groups, i.e., a sham exposure is explicitly mentioned<br>**AND**

• the sham exposure apparatus was identical to the exposure apparatus<br>**AND**<br>• all potential confounders (e.g., temperature) of the exposure were considered and, if necessary, were emulated in the sham exposure or potential confounders are counteracted<br>**AND**<br>• sham-exposed subjects or animals are treated as exposed ones<br>**AND**<br>• sham exposure and exposure occurred at the same time (or at the same time of the day) and conditions<br>**AND**<br>• any differences are small, well documented, and justified
Probably Low Risk of Bias (+)
• indirect evidence that exposure-associated side effects and any other conditions were identical across study groups, i.e., a sham exposure is explicitly mentioned<br>**AND**<br>• sham exposure apparatus is at least very similar to the exposure apparatus<br>**AND**<br>• exposure confounders (e.g., temperature) were not fully considered and emulated or were not counteracted<br>**AND**<br>• sham-exposed subjects or animals were treated as exposed ones<br>**AND**<br>• sham exposure and exposure took place, at least, at comparable times and conditions<br>**AND**<br>• any differences are documented and justified<br>**OR**<br>• it is deemed that exposure-associated side effects did not exist or would not appreciably bias results
Probably High Risk of Bias (-)
• indirect evidence that exposure-associated side effects or some other conditions differed between study groups<br>**OR**<br>• a sham exposure is explicitly mentioned or there is indirect evidence for the existence of a sham exposure, but no or insufficient details are provided to assume identical conditions across study groups/conditions<br>**OR**<br>• insufficient information is provided (e.g., not reported or "NR")
Definitely High Risk of Bias (--)
• direct evidence that exposure-associated side effects or some other conditions differed between study groups<br>**OR**<br>• direct evidence that there was no sham exposure<br>**OR**<br>• it is very likely that there was no sham exposure because only terms, such as "control group" or "control condition" without further explanations, are used

Question 4: Were the research personnel and subjects blinded to the study group during the study?
EA, HCT
Definitely Low Risk of Bias (++)
• direct evidence that research personnel (and subjects in HCT) were adequately blinded to study groups and it is unlikely that they could have broken the blinding until the full completion of the experiment performance
Probably Low Risk of Bias (+)
• indirect evidence that research personnel (and subjects in HCT) were adequately blinded to study groups and it is unlikely that they could have broken the blinding during the study<br>**OR**<br>• it is deemed that lack of adequate blinding during the study would not appreciably bias results
Probably High Risk of Bias (-)
• indirect evidence that it was possible for research personnel (or subjects in HCT) to infer the study groups<br>**OR**<br>• there is insufficient information provided about blinding to study groups during the study (record "NR" as basis for answer)
Definitely High Risk of Bias (--)
• direct evidence for lack of adequate blinding of the study groups, including no blinding or incomplete blinding of research personnel (and subjects in HCT)
**Attrition/Exclusion Bias**
**Question 5: Were outcome data complete without attrition or exclusion from analysis?**
EA, HCT
Definitely Low Risk of Bias (++)
• direct evidence that there was no loss of subjects or animals during the study and outcome data were complete<br>**OR**<br>• loss of subjects (i.e., incomplete outcome data) was adequately addressed and reasons were documented when subjects or animals were removed from a study
Probably Low Risk of Bias (+)
• indirect evidence that loss of subjects or animals (i.e., incomplete outcome data) was adequately addressed and reasons were documented when subjects or animals were removed from a study<br>**OR**<br>• it is deemed that the proportion lost to follow-up would not appreciably bias results
Probably High Risk of Bias (-)
• indirect evidence that loss of subjects or animals (i.e., incomplete outcome data) was unacceptably large (greater than 20% in each group) and not adequately addressed<br>**OR**<br>• there is insufficient information provided about numbers of subjects or animals lost to follow-up (record "NR" as basis for answer).
Definitely High Risk of Bias (--)
• direct evidence that loss of subjects or animals (i.e., incomplete outcome data) was unacceptably large and not adequately addressed

Detection Bias
**Question 6: Can we be confident in the exposure characterization?**<br>*In this criterion, we will specifically assess the quality of the RF EMF exposure characterization. The following basic pieces of information are required for EA and HCT: frequency, modulation scheme, description of the signal source and exposure set-up, methods and devices used for dosimetry (measurement, calculation, simulation), valid exposure metric, exposure duration, and background exposure measurements.*<br>*Studies are considered to have a risk of bias if there is insufficient exposure contrast between the exposed and non-exposed groups. Insufficient exposure contrast may appear in cases where low exposure levels are applied without shielding the experimental setup from environmental background exposure. Taking into account assessment uncertainties and fluctuations of environmental radiofrequency fields, an exposure level less than 10 dB (factor 3.16 for field strengths and factor 10 for power flux or energy density) above RF background level is assumed to be insufficient.*
EA, HCT
Definitely Low Risk of Bias (++)
• direct evidence that the exposure was adequately characterized (*e.g., all of the above-mentioned exposure information was provided, deemed suitable and sensible*)<br>**AND**<br>• consistently administered (i.e., with the same method and time frame) across study groups<br>**AND**<br>• there is direct evidence that exposure contrast between different groups was sufficient, in particular when only one exposure level was applied, direct evidence must exist that environmental background field strength was sufficiently lower than the applied exposure level (either by reported measurement data of background fields or by using a setup in which exposed animals/subjects are shielded from environmental fields)
Probably Low Risk of Bias (+)
• indirect evidence that the exposure was adequately characterized *(e.g., most of the above-mentioned exposure information was provided, comprehensible enough to still be deemed suitable and sensible)*<br>**AND**<br>• consistently administered (i.e., with the same method and time frame) across study groups<br>**AND**<br>• there is indirect evidence that exposure contrast between different groups was sufficient, in particular when only one exposure level was applied, indirect evidence must exist that environmental background field strength was sufficiently lower than the applied exposure level *(e.g., no shielding against environmental fields and no measurement data of background fields available, but applied exposure level is well above typical environmental field strengths)*
Probably High Risk of Bias (-)
• indirect evidence that the exposure was assessed using poorly validated methods or does not allow the assumption of sufficient exposure contrast *(e.g. background exposure was not measured, and exposure contrast was expected to be low)*<br>**OR**<br>• many important parameters lacking *(e.g. exposure source)*<br>**OR**<br>• there is insufficient information provided about the validity of the exposure assessment method, but no evidence for concern (record "NR" as basis for answer) *(e.g., measurement method not described)*

• direct evidence that the exposure was assessed using poorly validated methods (*e.g., studies reporting exposure metrics for which the method of exposure assessment is not provided, or the provided exposure metric has been calculated or measured for situations which do not reflect the actual experimental conditions, like SAR values from product compliance testing provided by mobile phone manufacturer OR background exposure was measured and exposure contrast is proven to be low)*<br>**OR**<br>• most important parameters are lacking *(e.g., exposure duration)*
**Question 7: Can we be confident in the outcome assessment?**
EA, HCT
Definitely Low Risk of Bias (++)
• direct evidence that the outcome was assessed using well-established methods<br>**AND**<br>• subjects had been followed-up for the same length of time in all study groups **AND**<br>• the outcome assessors (including study subjects, if outcomes were self-reported) were adequately blinded to the study groups, and it is unlikely that they could have broken the blinding prior to assessing outcomes
• indirect evidence that the outcome was assessed using acceptable methods<br>**AND**<br>• subjects or animals had been followed for the same length of time in all study groups<br>**OR**<br>• it is deemed that the outcome assessment methods used would not appreciably bias results<br>**AND**<br>there is indirect evidence that the outcome assessors (including study subjects, if outcomes were self-reported) were adequately blinded to the study group, and it is unlikely that they could have broken the blinding prior to assessing outcomes<br>**OR**<br>it is deemed that lack of adequate blinding of outcome assessors would not appreciably bias results, which is more likely to apply to objective outcome measures
Probably High Risk of Bias (-)
• indirect evidence that the outcome assessment method is an insensitive instrument<br>**OR**<br>• the length of follow-up differed by study group<br>**OR**<br>• it was possible for outcome assessors (including study subjects if outcomes were self-reported) to infer the study group prior to reporting outcomes<br>**OR**<br>• there is insufficient information provided about blinding of outcome assessors (record "NR" as basis for answer).
Definitely High Risk of Bias (--)
• direct evidence that the outcome assessment method is an insensitive instrument<br>**OR**<br>• the length of follow-up differed by study group<br>**OR**

• direct evidence for lack of adequate blinding of outcome assessors (including study subjects if outcomes were self-reported), including no blinding or incomplete blinding

**Selective Reporting Bias**
**Question 8: Were all measured outcomes reported?**
EA, HCT
Definitely Low Risk of Bias (++)
• direct evidence that all of the study's measured endpoints (primary and secondary) outlined in the protocol, methods, abstract, and/or introduction (that are relevant for the evaluation) have been reported
Probably Low Risk of Bias (+)
• indirect evidence that all of the study's measured endpoints (primary and secondary) outlined in the protocol, methods, abstract, and/or introduction (that are relevant for the evaluation) have been reported<br>**OR**<br>• analyses that had not been planned in advance are clearly indicated and it is deemed that the unplanned analyses were appropriate and that selective reporting would not appreciably bias results. This would include outcomes reported with insufficient detail, such as reporting only results that were statistically significant (or not)
Probably High Risk of Bias (-)
• indirect evidence that not all of the study's measured endpoints (primary and secondary) outlined in the protocol, methods, abstract, and/or introduction (that are relevant for the evaluation) have been reported<br>**OR**<br>• indirect evidence that unplanned analyses were included, which may appreciably bias results<br>**OR**<br>• there is insufficient information provided about selective outcome reporting (record "NR" as basis for answer)
Definitely High Risk of Bias (--)
• direct evidence that not all of the study's measured endpoints (primary and secondary) outlined in the protocol, methods, abstract, and/or introduction (that are relevant for the evaluation) have been reported. In addition to not reporting outcomes, this would include reporting outcomes based on composite score without individual outcome components or outcomes reported using measurements, analysis methods or subsets of the data (e.g., subscales) that were not pre-specified or reporting outcomes not pre-specified, or that unplanned analyses were included, which would appreciably bias results

**Other Bias**
**Question 9: Were there any other potential threats to internal validity?**
EA, HCT
Definitely Low Risk of Bias (++)
• direct evidence that appropriate statistics were performed<br>**AND**<br>• direct evidence that researchers adhered to the study protocol<br>**AND**

• direct evidence that the study design accounts for important confounding variables in EA and HCT (if applicable)
Probably Low Risk of Bias (+)
• indirect evidence that appropriate statistics were performed<br>**AND**<br>• indirect evidence that researchers adhered to the study protocol<br>**AND**<br>• indirect evidence that the study design accounts for important confounding variables in EA and HCT (if applicable)
Probably High Risk of Bias (-)
• indirect evidence that inappropriate statistics were performed<br>**OR**<br>• there is insufficient information provided about appropriateness of statistical methods (record "NR" as basis for answer)<br>**OR**<br>• indirect evidence that researchers did not adhere to the study protocol<br>**OR**<br>• indirect evidence that there were any other potential threats to internal validity (e.g., important confounding variables in EA and HCT)
Definitely High Risk of Bias (--)
• direct evidence that inappropriate statistics were performed OR no statistical analysis was performed<br>**OR**<br>• direct evidence that researchers did not adhere to the study protocol<br>**OR**<br>• direct evidence that there were any other potential threats to internal validity (e.g., important confounding variables in EA and HCT)
