# Supplementary material for: Physiological and health-related effects of 5G-relevant radiofrequency fields: A systematic review of experimental studies in humans and mammals
Source: PLoS One. 2026 Nov 4;21(11):e0361104. doi: 10.1371/journal.pone.0361104 (PMC13734471; doi:10.1371/journal.pone.0361104)
Supplement: Supplementary file 7 [file pone.0361104.s007.pdf]

S7 Example of Study Appraisal. Placement of studies into one of three quality categories (1st, 2nd, 3rd tier).

Quality category	Guidance		Risk-of-bias criteria (Criteria in bold and with black frames indicate key criteria.)								
			**Confidence in exposure**	**Confidence in outcome**	**Identical conditions**	Randomization	Allocation concealment	Blinding (during study)	Attrition/exclusion rate	Reporting	Other potential threats
**1st tier (high quality)**	"definitely low" or "probably low" risk of bias for all key criteria AND "definitely low" or "probably low" risk of bias for ≥ 50% of other criteria	example 1	+	++	+	+	+	−	−	++	+
**2nd tier (moderate quality)**	study does not meet criteria for 1st or 3rd tier	example 2	++	−	+	−	−	+	+	++	−
		example 3	−	−−	+	+	−	++	−	+	+
**3rd tier (low quality)**	"definitely high" or "probably high" risk of bias for all key criteria OR "definitely high" or "probably high" risk of bias for ≥ 50% of key criteria and ≥ 50% other criteria	example 4	+	−	−	−	−	++	+	−−	+
		example 5	−−	−	−	+	−	++	−	−	−

Based on risk-of-bias ratings for the applied criteria: "++" definitely low risk of bias, "+" probably low risk of bias, "-" probably high risk of bias, or "--" definitely high risk of bias. Adapted from the approach recommended by the National Toxicology Program's Office of Health Assessment and Translation (NTP 2015; 2019). To be placed into the 1st tier, a study must be rated as "definitely low risk of bias" or "probably low risk of bias" for all key criteria. Additionally, ≥ 50% of the remaining criteria must be rated as "definitely low risk of bias" or "probably low risk of bias". To be placed into the 3rd tier, a study must be rated as "definitely high risk of bias" or "probably high risk of bias" for all key criteria. Alternatively, ≥ 50% of the key criteria and ≥ 50% of the remaining criteria must be rated as "definitely high risk of bias" or "probably high risk of bias". Studies which could neither be assigned to the 1st tier nor 3rd tier are placed into the 2nd tier.
