# Supplementary material for: Physiological and health-related effects of 5G-relevant radiofrequency fields: A systematic review of experimental studies in humans and mammals
Source: PLoS One. 2026 Nov 4;21(11):e0361104. doi: 10.1371/journal.pone.0361104 (PMC13734471; doi:10.1371/journal.pone.0361104)
Supplement: Supplementary file 8 [file pone.0361104.s008.pdf]

S8 Risk-of-Bias Figure. Risk-of-bias ratings for 3rd tier studies.

	Randomization	Allocation concealment	**Identical conditions**	Blinding (during study)	Attrition/exclusion rate	**Confidence in exposure**	**Confidence in outcome**	Reporting	Other potential threats	**Study Quality**
Akdag et al. (1999b)	-	-	-	-	++	+	-	++	+	3rd tier
Akdag et al. (2002)	-	-	-	-	+	--	-	-	-	3rd tier
Appleton et al. (1975)	-	-	--	-	-	++	-	+	-	3rd tier
Baranski et al. (1967)	-	-	-	-	-	--	-	--	--	3rd tier
Baranski et al. (1971)	-	-	--	-	-	+	-	+	--	3rd tier
Baranski et al. (1972a)	-	-	--	-	-	--	-	-	-	3rd tier
Baranski et al. (1972b)	-	-	--	-	-	--	-	-	-	3rd tier
Bertharion et al. (1971)	-	-	--	-	-	--	--	-	-	3rd tier
Cain et al. (1978)	-	--	-	-	-	-	-	+	-	3rd tier
Chiang et al. (1987)	+	-	-	-	-	-	+	++	+	3rd tier
Crouzier et al. (2014)	+	-	-	-	+	-	-	+	-	3rd tier
Dasdag et al. (2001)	-	-	-	-	++	-	+	++	-	3rd tier
Deichmann et al. (1959)	-	-	-	-	-	+	-	-	-	3rd tier
Deichmann et al. (1964)	-	-	-	-	+	+	-	-	+	3rd tier
deLorge et al. (1984)	--	n.a.	-	-	+	++	-	+	-	3rd tier
Drobakhin et al. (2020)	-	-	--	-	-	--	--	-	--	3rd tier
Drobakhin et al. (2021)	-	-	--	--	+	--	--	-	-	3rd tier
Van Eeghem et al. (2017)	-	-	-	-	+	-	-	-	-	3rd tier
Fontani et al. (2011)	++	-	--	--	-	--	--	-	--	3rd tier
Glushkova et al. (2007)	-	-	-	++	-	+	-	-	--	3rd tier
Gostyukhina et al. (2022)	+	-	--	--	+	-	-	+	+	3rd tier
Gunn et al. (1961)	-	-	--	-	-	+	-	+	-	3rd tier
Jensh et al. (1984b)	+	-	-	-	-	+	-	++	+	3rd tier
Liu et al. (2022)	+	-	-	-	-	+	-	-	--	3rd tier
Manikowska et al. (1979)	-	-	--	-	-	-	-	+	-	3rd tier
McAfee et al. (1985)	-	-	--	-	+	+	-	-	--	3rd tier
Mikolajczyk et al. (1991)	-	-	--	-	-	+	-	++	-	3rd tier
Olazaran et al. (2013)	++	-	-	-	+	--	++	-	-	3rd tier
Moussa (2009)	-	-	--	-	-	--	-	+	-	3rd tier
Paulraj & Behari (2012b)	-	-	-	-	-	+	-	-	-	3rd tier
Paulsson et al. (1979)	-	-	--	-	-	+	-	+	-	3rd tier
Prausnitz et al. (1962)	-	-	-	-	-	+	-	--	--	3rd tier
Pu et al. (1997)	+	-	-	-	++	--	-	++	-	3rd tier
Ray & Behari (1990)	-	-	-	-	+	-	-	+	-	3rd tier
Rifat et al. (2017)	-	-	-	-	++	-	-	++	-	3rd tier
Rinaldi et al. (2014)	++	-	-	++	-	--	-	-	-	3rd tier
Roberti et al. (1975)	+	-	-	-	-	+	-	-	-	3rd tier
Servantje et al. (1975)	-	-	-	-	-	--	-	-	--	3rd tier
Sharma et al. (2014b)	-	-	-	-	++	+	-	++	-	3rd tier
Sharma et al. (2017a)	-	-	-	-	-	+	-	+	-	3rd tier
Sharma et al. (2017b)	-	-	-	-	-	+	-	++	-	3rd tier
Sheikh et al. (2013)	-	-	--	-	+	--	-	++	-	3rd tier
Shtemberg et al. (2001)	-	-	-	-	--	--	-	+	-	3rd tier
Szmigielski et al. (1975)	-	-	--	-	+	--	-	+	--	3rd tier
Szmigielski et al. (1977a)	-	-	--	-	-	+	-	++	+	3rd tier
Szmigielski et al. (1977b)	-	-	-	-	+	-	+	+	+	3rd tier
Teng et al. (1996)	-	-	-	-	-	++	-	-	-	3rd tier
Thomas et al. (1975)	+	n.a.	-	-	-	+	-	+	--	3rd tier
Thuroczy et al. (1994)	-	-	-	-	-	-	+	-	+	3rd tier
Varma et al. (1975)	-	-	-	-	-	-	-	+	-	3rd tier
Walani et al. (2014)	-	-	-	-	-	+	-	+	--	3rd tier
Wang et al. (2023b)	--	-	-	-	-	--	+	+	+	3rd tier
Yang et al. (2022)	+	-	-	-	-	-	+	++	+	3rd tier
Yao et al. (2022)	+	-	-	-	-	-	-	-	--	3rd tier
Yin et al. (2021)	++	-	--	-	+	--	-	+	-	3rd tier
Zhang et al. (2015)	-	-	++	-	-	-	-	++	-	3rd tier
Zhao et al. (2022)	+	-	-	-	-	-	+	++	+	3rd tier

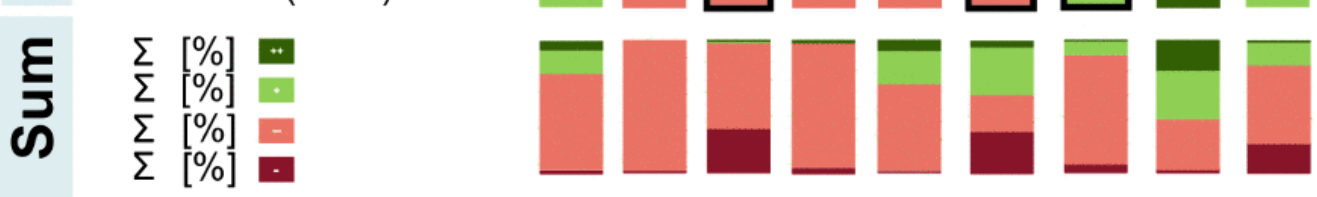

- ++ definitely low risk of bias
- + probably low risk of bias
- - probably high risk of bias
- -- definitely high risk of bias

Figure S1. Risk-of-bias ratings for 3rd tier studies (n = 57). Criteria ratings served as the basis for the assignment of individual studies to the quality categories. Black frames indicate key risk-of-bias criteria. The list of references for all 3rd tier studies can be found in S16 Reference List.
