# Supplementary material for: Physiological and health-related effects of 5G-relevant radiofrequency fields: A systematic review of experimental studies in humans and mammals
Source: PLoS One. 2026 Nov 4;21(11):e0361104. doi: 10.1371/journal.pone.0361104 (PMC13734471; doi:10.1371/journal.pone.0361104)
Supplement: Supplementary file 9 [file pone.0361104.s009.pdf]

**S9 Exposure Table.** Comparison of exposure metrics with ICNIRP limits (1st and 2nd tier studies).

| Authors, study design | Exposure specifications | Relevant exposure metrics | Conversion | Comparison to limit values |
|---|---|---|---|---|
| **CNS, cognitive functions, and behavior** | | | | |
| Bektas et al. 2022<br><br>EA<br><br>Study quality: 2nd tier | GSM signal generator (antenna), 3.5 GHz (5G), PW (rate 217 Hz; GSM)<br><br>2 conditions: sham exposure and exposure (28.1 V/m, PD 1.6 W/m², SAR 37 mW/kg (whole-body), SAR 323 mW/kg and SAR 195 mW/kg (for 1 g and 10 g averaging, respectively (gray matter)) (measurement, calculation/simulation)<br><br>Head exposure (1 cm distance to antenna)<br><br>Exposure duration: 2 h/day, 5 days/week for 1 month (30 days)<br><br>Remarks: 7 immobilized rats were simultaneously exposed in a plexiglass carousel with the antenna in the center | 28.1 V/m<br><br>PD 1.6 W/m²<br><br>SAR 37 mW/kg (whole-body)<br><br>SAR 323 mW/kg for 1 g gray matter)<br><br>SAR 195 mW/kg (for 10g averaging gray matter) | 28.1 V/m<br><br>PD 1.6 W/m<br><br>0,037 W/kg<br><br>0,323 W/kg<br><br>0,195 W/kg | n.a.<br><br>below<br><br>below<br><br>n.a. for 1 g<br><br>below<br><br>**Exposure below limit values***|
| * Note: Due to different anatomy, body geometry and dimensions, the absorption distribution in the tissue of the test animals is significantly different from that in humans with the same externally applied field strength or power flux density. If the dosimetric data provided by the authors are correct, it can be assumed that the SAR averaged over the brain and the maximum SAR occurring in the cortex of the brain was greater in the test animals than it would be the case in humans if the limit values were exhausted. | | | | |
| Chiang et al. 1984<br><br>EA<br><br>Study quality: 2nd tier | Radar, 3.0 GHz, PW (rate 935 pps, width 1.2 µs)<br><br>7 conditions: control group, sham exposure and 5 exposure conditions; group 1 (PD 0.1 mW/cm²), groups 2 and 6 (PD 0.5 mW/cm²), group 3 (PD 1 mW/cm²), group 4 (PD 5 mW/cm²), group 5 (PD 0.3 mW/cm²) (measurement)<br><br>Whole body exposure<br><br>Exposure duration: 3 h<br><br>Remarks: animals exposed individually with their bodies parallel to the E-vector | Group 1: PD 0.1 mW/cm²<br><br>Group 5: PD 0.3 mW/cm²<br><br>Groups 2 and 6: PD 0.5 mW/cm²<br><br>Group 3: PD 1 mW/cm<br><br>Group 4: PD 5 mW/cm² | 1 W/m²<br><br>3 W/m²<br><br>5 W/m²<br><br>10 W/m²<br><br>40 W/m² | below<br><br>below<br><br>below<br><br>exactly at limit value<br><br>above<br><br>**Exposure below and above limit values*** |

| Authors, study design | Exposure specifications | Relevant exposure metrics | Conversion | Comparison to limit values |
|---|---|---|---|---|
| **CNS, cognitive functions, and behavior** | | | | |
| * Note: Due to different anatomy, body geometry and dimensions, the absorption distribution in the tissue of the test animals is significantly different from that in humans with the same externally applied field strength or power flux density. If the dosimetric data provided by the authors are correct, it can be assumed that the SAR averaged over the brain and the maximum SAR occurring in the cortex of the brain was greater in the test animals, at least for PD values ≥ 3 W/m$^2$, than it would be the case in humans if the limit values were exhausted. | | | | |
| D'Andrea et al. 1994<br><br>EA<br><br>Study quality: 1st tier | Horn antenna, 5.620 GHz, PW (rate 100 pps, width 2.8 ps (radar) and 50 ns (Stanford linear energy doubler; SLED)<br><br>7 conditions: sham exposure and 6 exposure groups:<br>group 1 (radar pulse, SAR 2 W/kg (whole body), PD 56 W/cm² (peak)),<br>group 2 (radar pulse, SAR 4 W/kg, PD 128 W/cm²),<br>group 3 (radar pulse, SAR 6 W/kg, PD 277 W/cm²),<br>group 4 (SLED pulse, SAR 2 W/kg, PD 518 W/cm²),<br>group 5 (SLED pulse, SAR 4 W/kg, PD 1270 W/cm²),<br>group 6 (SLED pulse, SAR 6 W/kg, PD 2520 W/cm²)<br>(measurement, calibration)<br><br>Whole body exposure (focus on head)<br><br>Exposure duration: 20 min/session (probably one session for each exposure condition)<br><br>Remarks: animals partially restrained | Group 1: SAR 2 W/kg<br><br>Group 2: SAR 4 W/kg<br><br>Group 3: SAR 6 W/kg<br><br>Group 4: SAR 2 W/kg<br><br>Group 5: SAR 4 W/kg<br><br>Group 6: SAR 6 W/kg | SAR 2 W/kg<br><br>SAR 4 W/kg<br><br>SAR 6 W/kg<br><br>SAR 2 W/kg<br><br>SAR 4 W/kg<br><br>SAR 6 W/kg<br><br>Additionally, PD peak values of at least 56 W/cm² = 560000 W/m² | above<br><br>above<br><br>above<br><br>above<br><br>above<br><br>above<br><br>**Exposure above limit values** |
| De Lorge and Ezell 1980<br><br>EA<br><br>Study quality: 2nd tier | Horn antenna, 5.62 GHz, PW (rate 662 pps, width 0.5 or 2 µs)<br><br>9 conditions: sham exposure and 8 exposure conditions (PD 7.5, 11.5, 16, 26, 31.5, 38.5, 42, and 48.5 mW/cm²)<br>(measurement, calculation/simulation)<br><br>Exposure of right side of the rat<br><br>Exposure duration: 1-7 x 40-min sessions at each PD, 1 session/day, 5 day/week | PD 7.5 mW/cm²<br>PD 11.5 mW/cm²<br>PD 16 mW/cm²<br>PD 26 mW/cm²<br>PD 31.5 mW/cm²<br>PD 38.5 mW/cm²<br>PD 42 mW/cm²<br>PD 48.5 mW/cm² | 75 W/m²<br>115 W/m²<br>160 W/m²<br>260 W/m²<br>315 W/m²<br>385 W/m²<br>420 W/m²<br>485 W/m² | above<br>above<br>above<br>above<br>above<br>above<br>above<br>above<br><br>**Exposure above limit values***  |
| *Relevant temperature increase in animals likely at higher exposure levels. | | | | |

| Authors, study design | Exposure specifications | Relevant exposure metrics | Conversion | Comparison to limit values |
|---|---|---|---|---|
| **CNS, cognitive functions, and behavior** | | | | |
| Jamal et al. 2023<br><br>HCT, cross-over study<br><br>Study quality: 2nd tier | Horn antenna, 3.5 GHz (5G)<br>PW (width 577 µs, rate 4.6 ms)<br><br>2 conditions: sham exposure and 3.5 GHz exposure (2 V/m (rms; at the head; 1.5 V/m at the trunk), PD 0.68 W/m² peak power, SAR 0.037 ± 0.11 mW/kg (head-averaged), SAR 0.008 ± 0.019 mW/kg (brain-averaged))<br>(measurement, calculation/simulation)<br><br>Head and trunk exposure<br><br>Exposure duration: approx. 25 min and 30 s<br><br>Remarks: volunteers were exposed in two sessions with a maximum of one week apart; (sham) exposure sessions comprised separate conditions with eyes open and eyes closed | 2 V/m (at the head)<br>1.5 V/m (at the trunk)<br><br>PD 0.68 W/m² peak power<br><br>SAR 0.037 ± 0.11 mW/kg (head-averaged)<br><br>SAR 0.008 ± 0.019 mW/kg (brain-averaged) | 2 V/m<br>1.5 V/m<br><br>PD 0.68 W/m²<br><br>0.000037 ± 0.00011 W/kg<br><br>0.000008 ± 0.000019 W/kg | n.a.<br><br>below<br><br>below<br><br>below<br><br>**Exposure below limit values** |
| Mickley et al. 1997<br><br>EA<br><br>Study quality: 2nd tier | Transmitter, 5.6 GHz, PW (width and rate not provided)<br><br>2 conditions: sham exposure and exposure (SAR 11.72 W/kg (averaged, head/neck)<br>(calculation/simulation)<br><br>Head exposure<br><br>Exposure duration: 45 min<br><br>Remarks: pulse rate automatically controlled by using feedback from *in vivo* temperature probe, animals were anaesthetized | SAR 11.72 W/kg | 11.72 W/kg | above<br><br>**Exposure above limit values***|
| * Relevant temperature increase in animals likely. | | | | |

| Authors, study design | Exposure specifications | Relevant exposure metrics | Conversion | Comparison to limit values |
|---|---|---|---|---|
| **CNS, cognitive functions, and behavior** | | | | |
| Olazaran et al. 2014<br><br>HCT, cross-over study<br><br>Study quality: 2nd tier | REAC-Emitter, 10.5 GHz, PW (single pulse)<br><br>2 conditions: sham exposure and exposure (7 µW/kg SAR) (no data provided on exposure validation)<br><br>Exposure duration: a single 250 ms burst<br><br>Remarks: pulse applied at the ear through metallic tip of REAC | SAR 7 µW/kg | 0.000007 W/kg | below<br><br>**Exposure below limit values** |
| Paulraj and Behari 2006<br><br>EA<br><br>Study quality: 2nd tier | Horn antenna, 16.5 GHz, AM<br><br>2 conditions: sham exposure and exposure (PD 1.0 mW/cm², SAR 2.01 W/kg) (measurement, calculation/simulation, calibration)<br><br>Whole body exposure<br><br>Exposure duration: 2 h/day for 35 days (maybe except weekend, not clearly stated)<br><br>Remarks: 2 rats were exposed simultaneously | PD 1.0 mW/cm²<br><br>SAR 2.01 W/kg | 10 W/m²<br><br>2.01 W/kg | exactly at limit value<br><br>above<br><br>**Exposure above limit values***  |
| *The information on SAR provided by the authors does not appear consistent based on physical considerations (SAR in the animals was presumably < 1 W/kg). Due to different anatomy, body geometry and dimensions, the absorption distribution in the tissue of the test animals is also significantly different from that in humans with the same externally applied field strength or power flux density. Overall, it can be assumed from this study that the SAR averaged over the brain and the maximum SAR occurring in the cortex of the brain was greater in the test animals than would be the case in humans if the limit values were exhausted. | | | | |

| Authors, study design | Exposure specifications | Relevant exposure metrics | Conversion | Comparison to limit values |
|---|---|---|---|---|
| **CNS, cognitive functions, and behavior** | | | | |
| Paulraj and Behari 2012<br><br>EA<br><br>Study quality: 1st tier | Pyramidal horn antenna, 9.9 GHz, square wave modulated (1 kHz)<br><br>2 conditions: sham exposure and exposure (PD 0.125 mW/cm², SAR 1.0 W/kg (whole body))<br>(measurement, calculation/simulation)<br><br>Whole body exposure<br><br>Exposure duration: 2 h/day, 6 days/week for 35 days<br><br>Remarks: 2 rats were exposed simultaneously | PD 0.125 mW/cm²<br><br>SAR 1.0 W/kg (whole body)) | 1.25 W/m²<br><br>1.0 W/kg | below<br><br>comparison not feasible*<br><br>**Exposure assessment in comparison to limit values not feasible*** |
| * The information on SAR provided by the authors does not appear consistent based on physical considerations (whole body SAR in the animals was presumably < 1 W/kg). Due to different anatomy, body geometry and dimensions, the absorption distribution in the tissue of the test animals is also significantly different from that in humans with the same externally applied field strength or power flux density. Due to these inconsistencies, it is not possible to establish a relation between the SAR values occurring in the test animals and the SAR values occurring in humans. | | | | |
| Qin et al. 2024<br><br>EA<br><br>Study quality: 2nd tier | Antenna, 4.9 GHz<br><br>2 conditions: sham exposure and exposure (PD 50 W/m²)<br>(no data provided on exposure validation)<br><br>Whole body exposure<br><br>Exposure duration: 1 h/day for 21 days | PD 50 W/m² | 50 W/m² | above<br><br>**Exposure above limit values** |
| Qin et al. 2023<br><br>EA<br><br>Study quality: 2nd tier | Antenna, 4.9 GHz<br><br>2 conditions: sham exposure and exposure (PD 50 ± 2.5 W/m²)<br>(no data provided on exposure validation)<br><br>Whole body exposure<br><br>Exposure duration: 1 h/day for 6-7 days (depending on endpoint) | PD 50 ± 2.5 W/m² | 50 ± 2.5 W/m² | above<br><br>**Exposure above limit values** |

| Authors, study design | Exposure specifications | Relevant exposure metrics | Conversion | Comparison to limit values |
|---|---|---|---|---|
| **CNS, cognitive functions, and behavior** | | | | |
| Quock et al. 1994<br><br>EA<br><br>Study quality: 2nd tier | Horn antenna, 4.7 GHz, CW<br><br>4 conditions: sham exposure and 3 exposure groups (SAR 4, 12, and 36 W/kg) (measurement)<br><br>Whole body exposure<br><br>Exposure duration: 5 min<br><br>Remarks: animals restrained | SAR 4 W/kg<br>SAR 12 W/kg<br>SAR 36 W/kg | 4 W/kg<br>12 W/kg<br>36 W/kg | above<br>above<br>above<br><br>**Exposure above limit values*** |
| * Relevant temperature increase in animals is possible at 12 W/kg and probable at 36 W/kg. | | | | |
| Raslear et al. 1993<br><br>EA<br><br>Study quality: 2nd tier | circular waveguide, 3.0 GHz, PW (width 80 ns, rate 0.125 pps)<br><br>7 conditions: sham exposure, cage control, 5 exposure groups (0, -20, -30, -40, -50 dB) (peak power 700 MW; SAR 0.072 W/kg (whole-body time-averaged), SARs in the brain ranged from 0.057-0.087 W/kg) (measurement, calculation/simulation)<br><br>Whole body exposure<br><br>Exposure duration: 200 pulses (during approx. 25 min)<br><br>Environmental conditions: influence of co-exposure to X-rays and noise emitted by the exposure system was investigated<br><br>Exposure remarks: 2 animals exposed simultaneously; animals restrained; SAR values for 24 body locations provided; antenna output was attenuated by wire screen of varying mesh densities | SAR 0.072 W/kg (whole-body time-averaged)<br><br>SARs in the brain:<br>Min. 0.057 W/kg<br>Max. 0.087 W/kg | 0.072 W/kg<br><br><br>0.057 W/kg<br>0.087 W/kg | below<br><br><br>below<br>below<br><br>**Exposure below limit values** |

| Authors, study design | Exposure specifications | Relevant exposure metrics | Conversion | Comparison to limit values |
|---|---|---|---|---|
| **CNS, cognitive functions, and behavior** | | | | |
| Rui et al. 2022<br><br>EA<br><br>Study quality: 2nd tier | Horn antenna, 5.8 GHz<br><br>3 conditions: sham exposure and 2 exposure groups (PD 74.25 W/m², SAR 1.15 W/kg (whole body), SAR 2.33 W/kg (brain)): group 1 (2 h/day), group 2 (4 h/day)<br>(measurement)<br><br>Whole body exposure<br><br>Exposure duration: 2 h or 4 h per day for 15 days<br><br>Remarks: rats were placed individually in boxes, arranged in 5 × 5 array and placed on the shelf with the lateral side facing the antenna (3.3 m away from the antenna) | PD 74.25 W/m²<br><br>SAR 1.15 W/kg (whole body)<br><br>SAR 2.33 W/kg (brain) | 74.25 W/m²<br><br>1.15 W/kg<br><br>2.33 W/kg | above<br><br>above<br><br>above<br><br>**Exposure above limit values (for whole body)** |
| Sahingöz Yildirim et al. 2022<br><br>EA<br><br>Study quality: 2nd tier | Horn antenna, 10.715 GHz<br><br>2 conditions: control and exposure group (PD 0.84 mW/cm², SAR 0.725 W/kg (average))<br>(calculation/simulation)<br><br>Whole body exposure<br><br>Exposure duration: 12 hours/day until birth (22 or 25 days) | PD 0.84 mW/cm²<br><br>SAR 0.725 W/kg (average)) | PD 8.4 W/m²<br><br>SAR 0.725 W/kg (average)) | below<br><br>above*<br><br>**Exposure above limit values***|
| *The SAR values occurring in the internal body tissues of animals can be assumed to be significantly higher than the corresponding SAR values occurring in humans at the same incident power flux density. It is therefore likely that the SAR values in internal body tissues in animals in this study were greater than those in humans if the limit value was exhausted. | | | | |

| Authors, study design | Exposure specifications | Relevant exposure metrics | Conversion | Comparison to limit values |
|---|---|---|---|---|
| **CNS, cognitive functions, and behavior** | | | | |
| Sharma et al. 2014a<br><br>EA<br><br>Study quality: 2nd tier | Horn antenna, 10 GHz<br><br>2 conditions: sham exposure,<br>10 GHz exposure (PD 0.25 mW/cm², SAR 0.1790 W/kg) (measurement, calculation/simulation)<br><br>Head exposure<br><br>Exposure duration: 2 h/day for 30 days<br><br>Remarks: animals were immobilized | PD 0.25 mW/cm²<br><br>SAR 0.1790 W/kg | 2.5 W/m²<br><br>0.1790 W/kg | below<br><br>no SAR limit value defined for local exposure > 6 GHz<br><br>**Exposure below limit values***  |
| *The SAR values occurring in the internal body tissues of animals can be assumed to be significantly higher than the corresponding SAR values occurring in humans at the same incident power flux density. Therefore, it cannot be ruled out that the SAR values in internal body tissues in animals in this study were greater than those in humans if the limit value was exhausted. | | | | |
| Wang et al. 2022<br><br>EA<br><br>Study quality: 2nd tier | Horn antenna, 4.3 GHz<br><br>2 conditions: sham exposure, 4.3 GHz exposure (average PD 10 mW/cm², SAR 2.5 W/kg) (calculation/simulation)<br><br>Whole body exposure<br><br>Exposure duration: 6 min | average PD 10 mW/cm²<br><br>SAR 2.5 W/kg | 100 W/m²<br><br>2.5 W/kg | above<br><br>above<br><br>**Exposure above limit values** |

| Authors, study design | Exposure specifications | Relevant exposure metrics | Conversion | Comparison to limit values |
|---|---|---|---|---|
| **CNS, cognitive functions, and behavior** | | | | |
| Wang et al. 2023<br><br>EA<br><br>Study quality: 2nd tier | Horn antenna, 9.375 GHz<br><br>3 conditions: sham exposure,<br>exposure group 1 (PD 10 mW/cm², SAR 3.375 W/kg (whole-body)),<br>exposure group 2 (PD 30 mW/cm², SAR 10.125 W/kg (whole-body))<br>(calculation/simulation)<br><br>Whole body exposure<br><br>Exposure duration: 10 min<br><br>Remarks: restrained rats were exposed with incident direction of the microwave from the dorsal side to the ventral side on a rotating platform | Exposure group 1: PD 10 mW/cm²<br>SAR 3.375 W/kg (whole-body)<br><br>exposure group 2: PD 30 mW/cm²<br>SAR 10.125 W/kg (whole-body) | 100 W/m²<br>3.375 W/kg<br><br>300 W/m²<br>10.125 W/kg | above<br>above<br><br>above<br>above<br><br>**Exposure above limit values***|
| * Relevant temperature increase in animals likely at higher exposure level. | | | | |
| Zhu et al. 2021<br><br>EA<br><br>Study quality: 2nd tier | Horn antenna, 4.3 GHz, PW (rate 200 pps, width 500 ns)<br><br>2 conditions: sham exposure and exposure (PD 10 mW/cm², SAR 3.3 W/kg (average))<br>(measurement, calculation/simulation)<br><br>Whole body exposure<br><br>Exposure duration: 6 min<br><br>Remarks: rat cages rotating during exposure; 35 rats were exposed in two batches simultaneously (housed individually in separate compartments); rectal temperature of 5 rats controlled before and after exposure | PD 10 mW/cm²<br><br>SAR 3.3 W/kg (average)) | 100 W/m²<br><br>3.3 W/kg | above<br><br>above<br><br>**Exposure above limit values** |

| Authors, study design | Exposure specifications | Relevant exposure metrics | Conversion | Comparison to limit values |
|---|---|---|---|---|
| **Immune system** | | | | |
| Fesenko et al. 1999<br><br>EA<br><br>Study quality: 2nd tier | Horn antenna, 10 GHz, 8.15–18 GHz, swept frequency (direct and reversed sweep times were 1.0 s and 16 ms, respectively)<br><br>5 conditions: sham exposure, cage control, control group and 2 exposure groups (PD 1 µW/cm$^2$ (spatial average), PD 0.2-1.6 µW/cm$^2$ (range), SAR 2-5 mW/kg): group 1 (10 GHz) and group 2 (swept frequency 8.15–18 GHz) (measurement)<br><br>Whole body exposure<br><br>Exposure duration: 0.5 h-7 days | PD 1 µW/cm$^2$ (spatial average)<br>PD 0.2-1.6 µW/cm$^2$ (range)<br><br>SAR 2-5 mW/kg | 0.01 W/m$^2$<br>0.002 - 0.016 W/m$^2$<br><br>0.002 - 0.005 W/kg | below<br>below<br><br>below*<br><br>**Exposure below limit values***|
| *The SAR values occurring in the animal internal body tissues can be assumed to be significantly higher than the corresponding SAR values occurring in humans at the same incident power flux density. | | | | |
| Liddle et al. 1980<br><br>EA<br><br>Study quality: 1st tier | Horn antenna, 9 GHz, PW (rate 970-1000, width 1.0 ps)<br><br>3 conditions: cage control, sham exposure and exposure (PD 10 mW/cm$^2$ (average), PD 10 W/cm$^2$ (peak), SAR 3.3 mW/g or 4.7 mW/g (depending on used model)<br>(measurement, calculation/simulation)<br><br>Whole body exposure (probably; explicitly described)<br><br>Exposure duration: 2 h/day for 5 days | PD 10 mW/cm$^2$ (average)<br>PD 10 W/cm$^2$ (peak)<br><br>SAR 3.3 mW/g or<br>4.7 mW/g | 100 W/m$^2$<br>100000 W/m$^2$<br><br>SAR 3.3 W/kg or<br>4.7 W/kg | above<br>-<br><br>above<br>above<br><br>**Exposure above limit values** |
| Liddle et al. 1986<br><br>EA<br><br>Study quality: 1st tier | Horn antenna, 9 GHz, PW (rate 970-1000, width 1.0 ps)<br><br>2 conditions: sham exposure and exposure (PD 1.0 mW/cm$^2$ (average), PD 1 W/cm$^2$ (peak), SAR 0.47 W/kg)<br>(measurement, calculation/simulation)<br><br>Whole body exposure (probably; explicitly described)<br><br>Exposure duration: 2h/day for 5 days | PD 1.0 mW/cm$^2$ (average)<br>PD 1 W/cm$^2$ (peak)<br><br>SAR 0.47 W/kg | 10 W/m$^2$<br>10000 W/m$^2$<br><br>0.47 W/kg | exactly at limit value<br>-<br><br>above<br><br>**Exposure above limit values** |

| Authors, study design | Exposure specifications | Relevant exposure metrics | Conversion | Comparison to limit values |
|---|---|---|---|---|
| **Immune system** | | | | |
| Novoselova et al. 1999<br><br>EA<br><br>Study quality: 2nd tier | Horn antenna, 8.15–18 GHz, swept frequency (direct and reversed sweep times were 1.0 s and 16 ms, respectively)<br><br>3 conditions: sham exposure, cage control, and exposure (PD 1 μW/cm² (spatial average), PD 0.2-1.6 μW/cm² (range), SAR 2-5 mW/kg) (measurement)<br><br>Whole body exposure<br><br>Exposure duration: 5 hours | PD 1 μW/cm² (spatial average)<br>PD 0.2-1.6 μW/cm² (range)<br><br>SAR 2-5 mW/kg | 0.01 W/m²<br>0.002 - 0.016 W/m²<br><br>0.002 - 0.005 W/kg | below<br>below<br><br>below*<br><br>**Exposure below limit values***|
| *The SAR values occurring in the animal internal body tissues can be assumed to be significantly higher than the corresponding SAR values occurring in humans at the same incident power flux density. | | | | |
| Novoselova et al. 2004<br><br>EA<br><br>Study quality: 1st tier | Rectangular horn-antenna, 8.15-18 GHz, swept frequency (direct and reversed sweep times were 1.0 s and 16 ms, respectively)<br><br>2 conditions: sham exposure and 2 exposure groups (PD 1 μW/cm² (spatial average), PD 0.4-1.6 μW/cm² (range), SAR 2-5 mW/kg) (measurement)<br><br>Exposure duration: 1.5 h/day for up to 30 days (starting on day 1 after transplantation of carcinoma cells)<br><br>Remarks: animal cancer model was produced by subcutaneous injection of Ehrlich ascite carcinoma cells in one hind limb | PD 1 μW/cm² (spatial average)<br>PD 0.4-1.6 μW/cm² (range)<br><br>SAR 2-5 mW/kg | 0.01 W/m²,<br>0.004 - 0.016 W/m²<br><br>0.002 - 0.005 W/kg | below<br>below<br><br>below*<br><br>**Exposure below limit values***|
| *The SAR values occurring in the animal internal body tissues can be assumed to be significantly higher than the corresponding SAR values occurring in humans at the same incident power flux density. | | | | |

| Authors, study design | Exposure specifications | Relevant exposure metrics | Conversion | Comparison to limit values |
|---|---|---|---|---|
| **Immune system** | | | | |
| Novoselova et al. 2017<br><br>EA<br><br>Study quality: 2nd tier | Rectangular horn antenna, 8.15–18 GHz, sweep frequency (direct and reversed sweep times were 1.0 s and 16 ms, respectively)<br><br>3 conditions: control, sham exposure and exposure (PD 0.4 µW/cm²-1.6 µW/cm², 1 µW/cm² (average)<br>(measurement)<br><br>Whole body exposure<br><br>Exposure duration: 1 h | PD 0.4 µW/cm²-1.6 µW/cm²,<br>1 µW/cm² (average) | 0.004 - 0.016 W/m²<br>0.01 W/m² | below<br>below<br><br>**Exposure below limit values***  |
| *The SAR values occurring in the animal internal body tissues can be assumed to be significantly higher than the corresponding SAR values occurring in humans at the same incident power flux density. | | | | |
| Rifat et al. 2013<br><br>EA<br><br>Study quality: 2nd tier | Horn antenna, 10 GHz<br><br>2 conditions: sham exposure and exposure (PD 0.25 mW/cm², SAR 0.1790 W/kg)<br>(measurement, calculation/simulation)<br><br>Whole body exposure<br><br>Exposure duration: 2h/day for 30 consecutive days | PD 0.25 mW/cm²<br><br>SAR 0.1790 W/kg | 2.5 W/m²<br><br>0.1790 W/kg | below<br><br>above*<br><br>**Exposure above limit values*** |
| *The SAR values occurring in the internal body tissues of animals can be assumed to be significantly higher than the corresponding SAR values occurring in humans at the same incident power flux density. Therefore, it cannot be ruled out that the SAR values in internal body tissues in animals in this study were greater than those in humans if the limit value was exhausted. | | | | |

| Authors, study design | Exposure specifications | Relevant exposure metrics | Conversion | Comparison to limit values |
|---|---|---|---|---|
| **Immune system** | | | | |
| Shao and Chiang 1989<br><br>EA<br><br>Study quality: 1st tier | Horn antenna, 3 GHz, PW (rate 933 Hz, width 1.2 µs)<br><br>17 conditions: 8 sham exposure groups and 9 exposure groups: group 1 (PD 1 mW/cm²), groups 2, 8, 9 (PD 5 mW/cm²), groups 3, 5, 6, 7 (PD 7 mW/cm²), group 4 (PD 12 mW/cm²)<br>(measurement)<br><br>Whole body exposure<br><br>Exposure duration: 60 min/day for 2 days<br><br>Remarks: animals were restrained | PD 1 mW/cm²<br><br>PD 5 mW/cm²<br><br>PD 7 mW/cm²<br><br>PD 12 mW/cm² | 10 W/m²<br><br>50 W/m²<br><br>70 W/m²<br><br>120 W/m² | exactly at limit values<br><br>above<br><br>above<br><br>above<br><br>**Exposure exactly at and above limit values***|
| *The SAR values occurring in the internal body tissues of animals can be assumed to be significantly higher than the corresponding SAR values occurring in humans at the same incident power flux density. Therefore, it cannot be ruled out that the SAR values in internal body tissues in animals in this study were greater than those in humans if the limit value was exhausted. | | | | |
| Veyret et al. 1991<br><br>EA<br><br>Study quality: 1st tier | Coaxial antenna, 9.4 GHz ± 0.01 GHz, PW (width 1 ps, rate 1,000 pps, 5 ns rise time)<br>with and without AM differentially modulated at 14-41 MHz<br><br>50 conditions: 25 sham exposure groups and 25 exposure groups (PD 30 ± 2 µW/cm² (RMS), SAR ~0.015 W/kg): PW with and without AM differentially modulated at 14-41 MHz<br>(measurement, calculation/simulation)<br><br>Whole body exposure<br><br>Exposure duration: 10 h/day for 5 days | PD 30 ± 2 µW/cm² (RMS)<br><br>SAR ~0.015 W/kg | 0.3 W/m²<br><br>0.015 W/kg | below<br><br>below<br><br>**Exposure below limit values*** |
| *The SAR values occurring in the internal body tissues of animals can be assumed to be significantly higher than the corresponding SAR values occurring in humans at the same incident power flux density. | | | | |

| Authors, study design | Exposure specifications | Relevant exposure metrics | Conversion | Comparison to limit values |
|---|---|---|---|---|
| **Thermoregulation and heat-related effects** | | | | |
| Blick et al. 1997<br><br>HCT<br><br>Study quality: 2nd tier | Different horn antennas, 7.5 GHz and 10.0 GHz<br><br>>3 conditions: control condition and 2 exposure conditions (different not further described intensities): condition 1 (7.5 GHz) and condition 2 (10 GHz) (measurement, calibration)<br><br>Exposure of the middle of the uncovered back (327 cm²)<br><br>Exposure duration: 20 single 10 s-stimuli for each frequency (at 1 min intervals)<br><br>Remarks: subjects were exposed in the E orientation (long axis of subject parallel to E-vector) | The detection thresholds were<br><br>19.6 ± 2.9 mW/cm² at 10 GHz and<br><br>19.5 ± 2.9 mW/cm² at 7.5 GHz | 196 ± 29 W/m²<br><br>195 ± 29 W/m² | above<br><br>above<br><br>**Exposure above limit values** |
| Frei et al. 1989<br><br>EA<br><br>Study quality: 1st tier | Antenna (CW), horn antenna (PW), 9.3 GHz, CW, PW (width 2 µs, rate 500 pps)<br><br>4 conditions: condition 1 (PD 30 mW/cm², SAR 9.3 W/kg (CW)), condition 2 (PD 30 mW/cm², SAR 9.3 W/kg (PW)), condition 3 (PD 60 mW/cm², SAR 18.6 W/kg (CW)), condition 4 (PD 60 mW/cm², SAR 18.6 W/kg (PW)) (measurement, calculation/simulation)<br><br>Whole body exposure<br><br>Exposure duration: until colonic temperature increased by 1°C from 38.5°C to 39.5°C (between approx. 13-41 min for one cycle, according to Figure)<br><br>Remarks: exposure was conducted to cyclically increase colonic temperature from 38.5 to 39.5°C; 8 x 1°C cycles in each animal (2 at 60 mW/cm², CW; 2 at 60 mW/cm², PW; 2 at 30 mW/cm², CW; 2 at 30 mW/cm², PW); anesthetized rats were exposed in H-orientation (left lateral exposure, long axis of animal parallel to the magnetic field) | condition 1: PD 30 mW/cm², SAR 9.3 W/kg (CW)<br><br>condition 2: PD 30 mW/cm², SAR 9.3 W/kg (PW)<br><br>condition 3: PD 60 mW/cm², SAR 18.6 W/kg (CW)<br><br>condition 4: PD 60 mW/cm², SAR 18.6 W/kg (PW) | PD 300 W/m², SAR 9.3 W/kg (CW)<br><br>PD 300 W/m², SAR 9.3 W/kg (PW)<br><br>PD 600 W/m², SAR 18.6 W/kg (CW)<br><br>PD 600 W/m², SAR 18.6 W/kg (PW) | above<br><br>above<br><br>above<br><br>above<br><br>**Exposure above limit values***  |
| *Relevant temperature increase in animals likely. | | | | |

| Authors, study design | Exposure specifications | Relevant exposure metrics | Conversion | Comparison to limit values |
|---|---|---|---|---|
| **Thermoregulation and heat-related effects** | | | | |
| Ijima et al. 2023<br><br>EA<br><br>Study quality: 2nd tier | Patch antenna, 26.5 GHz, CW<br><br>5 conditions: sham exposure (0 W/cm²) and 4 exposure groups: group 1 (APD 125 W/m²), group 2 (APD 250 W/m²), group 3 (APD 370 W/m²), group 4 (APD 500 W/m²)<br>(measurement, calculation/simulation)<br><br>Exposure of dorsal skin<br><br>Exposure duration: 18 min<br><br>Remarks: dorsal hair of rats shorn; anaesthetized rats were restrained and exposed individually; abdomen placed on a heating pad (39.5°C) | group 1: APD 125 W/m²<br><br>group 2: APD 250 W/m²<br><br>group 3: APD 370 W/m²<br><br>group 4: APD 500 W/m² | group 1: APD 125 W/m²<br><br>group 2: APD 250 W/m²<br><br>group 3: APD 370 W/m²<br><br>group 4: APD 500 W/m² | above<br><br>above<br><br>above<br><br>above<br><br>**Exposure above limit values***<br> |
| *Relevant temperature increase in animals likely (at least for APD ≥250 W/m²) | | | | |
| Ijima et al. 2023<br><br>EA<br><br>Study quality: 1st tier | Lens antenna (horn antenna and dielectric lens), 28.0 GHz, CW<br><br>3 conditions: sham exposure and 2 exposure groups: group 1 (APD 122 W/m², SAR 3.7 W/kg (whole body)), group 2 (APD 237 W/m², SAR 7.2 W/kg (whole body))<br>(measurement, calculation/simulation)<br><br>Whole body exposure/exposure of shorn dorsal side<br><br>Exposure duration: 40 min<br><br>Remarks: dorsal hair of rats shorn; rats restrained and exposed individually | group 1: APD 122 W/m², SAR 3.7 W/kg (whole body)<br><br>group 2: APD 237 W/m², SAR 7.2 W/kg (whole body) | group 1: APD 122 W/m², SAR 3.7 W/kg (whole body))<br><br>group 2 (APD 237 W/m², SAR 7.2 W/kg (whole body)) | above<br><br>above<br><br>**Exposure above limit values*** |
| *Relevant temperature increase in animals at 237 W/m² likely | | | | |

| Authors, study design | Exposure specifications | Relevant exposure metrics | Conversion | Comparison to limit values |
|---|---|---|---|---|
| **Thermoregulation and heat-related effects** | | | | |
| Jauchem et al. 1984<br><br>EA<br><br>Study quality: 2nd tier | Antenna, 5.6 GHz, CW, PW (width 1 or 2 µs, rate 250 or 500 pps)<br><br>4 conditions: condition 1 (PD 30 mW/cm², SAR 6 W/kg (CW)), condition 2 (PD 30 mW/cm², SAR 6 W/kg (PW)), condition 3 (PD 60 mW/cm², SAR 12 W/kg (CW)), condition 4 (PD 60 mW/cm², SAR 12 W/kg (PW)) (measurement, calculation/simulation)<br><br>Whole body exposure<br><br>Exposure duration: up to approx. 210 min. for 8 x 1°C-cycles (derived from a Figure); for one cycle at 60 mW/cm² approx. 6.3 ± 0.3 min and at 30 mW/cm² approx. 14.9 ± 1.2 min)<br><br>Exposure remarks: exposure was conducted to cyclically increase colonic temperature from 38.5 to 39.5°C; 8 x 1°C cycles in each animal; anesthetized rats were exposed in H-orientation | condition 1: PD 30 mW/cm², SAR 6 W/kg (CW)<br><br>condition 2: PD 30 mW/cm², SAR 6 W/kg (PW)<br><br>condition 3: PD 60 mW/cm², SAR 12 W/kg (CW)<br><br>condition 4: PD 60 mW/cm², SAR 12 W/kg (PW) | PD 300 W/m², SAR 6 W/kg (CW)<br><br>PD 300 W/m², SAR 6 W/kg (PW)<br><br>PD 600 W/m², SAR 12 W/kg (CW)<br><br>PD 600 W/m², SAR 12 W/kg (PW) | above<br><br>above<br><br>above<br><br>above<br><br>**Exposure above limit values***|
| *Relevant temperature increase in animals likely | | | | |

| Authors, study design | Exposure specifications | Relevant exposure metrics | Conversion | Comparison to limit values |
|---|---|---|---|---|
| **Male fertility** | | | | |
| Kumar et al. 2011<br><br>EA<br><br>Study quality: 2nd tier | Horn antenna, 10 GHz<br><br>2 conditions: sham exposure and exposure (PD 0.21 mW/cm², SAR 0.014 W/kg)<br>(measurement, calculation/simulation)<br><br>Whole body exposure<br><br>Exposure duration: 2 h/day for 45 days | PD 0.21 mW/cm²<br><br>SAR 0.014 W/kg | PD 2.1 W/m²<br><br>SAR 0.014 W/kg | below<br><br>below<br><br>**Exposure below limit values***|
| *The SAR values occurring in the internal body tissues of animals can be assumed to be significantly higher than the corresponding SAR values occurring in humans at the same incident power flux density. Therefore, it cannot be ruled out that the SAR values in internal body tissues in animals in this study were greater than those in humans if the limit value was exhausted. | | | | |
| Kumar et al. 2012<br><br>EA<br><br>Study quality: 2nd tier | Horn antenna, 10 GHz<br><br>2 conditions: sham exposure, exposure (PD 0.21 mW/cm², SAR 0.014 W/kg)<br>(measurement, calculation/simulation)<br><br>Whole body exposure<br><br>Exposure duration: 2 h/day for 45 days | PD 0.21 mW/cm²<br><br>SAR 0.014 W/kg | PD 2.1 W/m²<br><br>SAR 0.014 W/kg | below<br><br>below<br><br>**Exposure below limit values***|
| *The SAR values occurring in the internal body tissues of animals can be assumed to be significantly higher than the corresponding SAR values occurring in humans at the same incident power flux density. Therefore, it cannot be ruled out that the SAR values in internal body tissues in animals in this study were greater than those in humans if the limit value was exhausted. | | | | |
| Kumar et al. 2013<br><br>EA<br><br>Study quality: 2nd tier | Horn antenna, 10 GHz<br><br>2 conditions: sham exposure and exposure (PD 0.21 mW/cm², SAR 0.014 W/kg)<br>(measurement, calculation/simulation)<br><br>Whole body exposure<br><br>Exposure duration: 2 h/day for 45 days | PD 0.21 mW/cm²<br><br>SAR 0.014 W/kg | PD 2.1 W/m²<br><br>SAR 0.014 W/kg | below<br><br>below<br><br>**Exposure below limit values***|

| Authors, study design | Exposure specifications | Relevant exposure metrics | Conversion | Comparison to limit values |
|---|---|---|---|---|
| **Male fertility** | | | | |
| *The SAR values occurring in the internal body tissues of animals can be assumed to be significantly higher than the corresponding SAR values occurring in humans at the same incident power flux density. Therefore, it cannot be ruled out that the SAR values in internal body tissues in animals in this study were greater than those in humans if the limit value was exhausted. | | | | |
| Xue et al. 2022<br><br>EA<br><br>Study quality: 2nd tier | Horn antenna, 5.8 GHz<br><br>2 conditions: sham exposure and exposure (PD 74.25 W/m², SAR 1.15 W/kg (whole body), SAR 3.36 W/kg (testis)), (measurement, calculation/simulation)<br><br>Whole body exposure<br><br>Exposure duration: 1 h/day for 30 days | PD 74.25 W/m²<br><br>SAR 1.15 W/kg (whole body)<br><br>SAR 3.36 W/kg (testis) | PD 74.25 W/m²<br><br>SAR 1.15 W/kg<br><br>SAR 3.36 W/kg | above<br><br>above<br><br>-<br><br>**Exposure above limit values** |

| Authors, study design | Exposure specifications | Relevant exposure metrics | Conversion | Comparison to limit values |
|---|---|---|---|---|
| **Systemic effects** | | | | |
| Adang et al. 2009<br><br>EA<br><br>Study quality: 2nd tier | Horn antennas, 9.70 GHz, CW<br><br>2 conditions: sham exposure and exposure (PD 3.20 W/m² (mean) ± 30%) (measurement)<br><br>Whole body exposure<br><br>Exposure duration: 2 h/day, 7 days/week for up to 21 months | PD 3.20 W/m² (mean) ± 30% | PD 3.20 W/m² (mean) ± 30% | below<br><br>**Exposure below limit values***|
| *The SAR values occurring in the internal body tissues of animals can be assumed to be significantly higher than the corresponding SAR values occurring in humans at the same incident power flux density. Therefore, it cannot be ruled out that the SAR values in internal body tissues in animals in this study were greater than those in humans if the limit value was exhausted. | | | | |
| De Seze et al. 2020<br><br>EA<br><br>Study quality: 2nd tier | Horn antenna, 3.7 GHz and 10 GHz, PW (width 1-2.5 ns)<br><br>5 conditions: sham exposure and 4 exposure groups: group 1 (SAR 0.34 W/kg), group 2 (SAR 4.7 W/kg), group 3 (SAR 22 W/kg), group 4 (SAR 0.83 W/kg)<br>(measurement, calculation/simulation)<br><br>Whole body exposure<br><br>Exposure duration: exposure group 1 (10 s every 5 min for 1 hour), exposure group 2 (2 x 8 min), exposure group 3 (14 min), exposure group 4 (2 x 8 min repeated with 10 min interval on 5 days/week for 8 weeks) | group 1: SAR 0.34 W/kg<br><br>group 2: SAR 4.7 W/kg<br><br>group 3: SAR 22 W/kg<br><br>group 4: SAR 0.83 W/kg | SAR 0.34 W/kg<br><br>SAR 4.7 W/kg<br><br>SAR 22 W/kg<br><br>SAR 0.83 W/kg | above<br><br>above<br><br>above<br><br>above<br><br>**Exposure above limit values*** |
| *Relevant temperature increase in animals at SAR 22 W/kg likely. | | | | |
| Kössler et al. 1981<br><br>EA<br><br>Study quality: 1st tier | Horn antenna, 3.3 GHz<br><br>2 conditions: sham exposure and exposure (PD 2 mW/cm² (average)) (measurement)<br><br>Whole body exposure<br><br>Exposure duration: 6 h/day, 5 days/week for 12 weeks | PD 2 mW/cm² (average)) | PD 20 W/m² (average) | above<br><br>**Exposure above limit values** |

| Authors, study design | Exposure specifications | Relevant exposure metrics | Conversion | Comparison to limit values |
|---|---|---|---|---|
| **Systemic effects** | | | | |
| Jensh 1984a<br><br>EA<br><br>Study quality: 2nd tier | Horn antenna, 6.0 GHz<br><br>4 conditions: cage control, exposure chamber control, sham exposure, exposure (35 mW/cm², average SAR 7.28 W/kg)<br>(measurement)<br><br>Whole body exposure<br><br>Exposure duration: 8 h/day for 21 days during pregnancy<br><br>Remarks: power density was determined in preliminary experiments to not increase body temperature | 35 mW/cm²<br><br>average SAR 7.28 W/kg | 350 W/m²<br><br>average SAR 7.28 W/kg | above<br><br>above<br><br>**Exposure above limit values***|
| *Relevant temperature increase in animals cannot be ruled out. | | | | |
| Pyrpaso-poulou et al. 2004<br><br>EA<br><br>Study quality: 2nd tier | Horn antenna, 9.4 GHz, PW (GSM-like: width 20 ms, rate 50 Hz)<br><br>2 conditions: sham exposure and 2 exposure groups (PD 5 µW/cm², SAR 0.5 mW/kg (max.)): group 1 (exposed during days 1-3 post coitum), group 2 (exposed during days 4-7 post coitum)<br>(measurement, calculation/simulation)<br><br>Whole body exposure<br><br>Exposure duration: 3 to 4 days | PD 5 µW/cm²<br><br>SAR 0.5 mW/kg (max.) | PD 0.05 W/m²<br><br>SAR 0.0005 W/kg | below<br><br>below<br><br>**Exposure below limit values*** |
| *The SAR values occurring in the internal body tissues of animals can be assumed to be significantly higher than the corresponding SAR values occurring in humans at the same incident power flux density. | | | | |

| Authors, study design | Exposure specifications | Relevant exposure metrics | Conversion | Comparison to limit values |
|---|---|---|---|---|
| **Other topics** | | | | |
| Akdag et al. 1999<br><br>EA<br><br>Study quality: 2nd tier | Horn antenna, 9.450 GHz, CW<br><br>2 conditions: sham exposure and exposure (PD 2.65 mW/cm², SAR 1.80 W/kg)<br>(measurement)<br><br>Whole body exposure<br><br>Exposure duration: 1h/day for 21 days | PD 2.65 mW/cm²<br><br>SAR 1.80 W/kg | PD 26.5 W/cm²<br><br>SAR 1.80 W/kg | above<br><br>above<br><br>**Exposure above limit values** |
| Bektas et al. 2023<br><br>EA<br><br>Study quality: 2nd tier | Antenna, 3.5 GHz<br><br>4 conditions: 2 sham exposure groups and 2 exposure groups (28.1 V/m, PD 1.6 W/m², SAR 37 mW/kg (whole-body))<br>(measurement, calculation/simulation)<br><br>Exposure with head in direction of antenna<br><br>Exposure duration: 2 h/day for 30 days<br><br>Remarks: 7 rats were exposed in a carousel with the antenna placed in the center | 28.1 V/m<br><br>PD 1.6 W/m²<br><br>SAR 37 mW/kg | -<br><br>PD 1.6 W/m²<br><br>SAR 0.037 W/kg | -<br><br>below<br><br>below<br><br>**Exposure below limit values***|
| *The SAR values occurring in the internal body tissues of animals can be assumed to be significantly higher than the corresponding SAR values occurring in humans at the same incident power flux density. Therefore, it cannot be ruled out that the SAR values in internal body tissues in animals in this study were greater than those in humans if the limit value was exhausted. | | | | |

| Authors, study design | Exposure specifications | Relevant exposure metrics | Conversion | Comparison to limit values |
|---|---|---|---|---|
| **Other topics** | | | | |
| Hirsch et al. 1977<br><br>EA<br><br>Study quality: 2nd tier | Dish antenna, 3.0 GHz, CW<br><br>6 exposure conditions: group 1 (PD 50 mW/cm²), group 2 (100 mW/cm²), group 3 (200 mW/cm²), group 4 (300 mW/cm²), group 5 (400 mW/cm²), group 6 (500 mW/cm²)<br>(measurement, calculation/simulation)<br><br>Exposure of left eye<br><br>Exposure duration: 15 min/day for 30 days<br><br>Remarks: animals anesthetized during exposure; temperature probably > 42°C in strongest exposure groups | group 1: PD 50 mW/cm²<br><br>group 2: 100 mW/cm²<br><br>group 3: 200 mW/cm²<br><br>group 4: 300 mW/cm²<br><br>group 5: 400 mW/cm²<br><br>group 6: 500 mW/cm² | group 1: 500 W/m²<br><br>group 2: 1000 W/m²<br><br>group 3: 2000 W/m²<br><br>group 4: 3000 W/m²<br><br>group 5: 4000 W/m²<br><br>group 6: 5000 W/m² | above<br><br>above<br><br>above<br><br>above<br><br>above<br><br>above<br><br>**Exposure above limit values*** |
| *Relevant temperature increase in animals certain | | | | |
| Knyazeva et al. 2018<br><br>EA<br><br>Study quality: 2nd tier | Horn antenna, 10 GHz, PW (4000 pulses/session, width 100 ns)<br><br>5 conditions: sham exposure and 4 exposure groups (PD 1.5 × 10³ kW/cm² (peak), PD 1.5 × 10³ mW/cm² (average)): group 1 (pulse repetition rate 8 pps), group 2 (13 pps), group 3 (16 pps), group 4 (22 pps)<br>(measurement)<br><br>Whole body exposure<br><br>Exposure duration: 3 to 11 minutes, depending on the repetition frequency of the pulses (once/day for a 5-day period)<br><br>Remarks: animals probably restraint | PD 1.5 × 10³ kW/cm² (peak), PD 1.5 × 10³ mW/cm² (average) | PD 1500 kW/cm² (peak), PD 1500 mW/cm² (average) =<br>PD 15,000,000,000 W/m² (peak), PD 15,000 W/m² (average) | above<br><br>**Exposure above limit values** |

| Authors, study design | Exposure specifications | Relevant exposure metrics | Conversion | Comparison to limit values |
|---|---|---|---|---|
| **Other topics** | | | | |
| Kumar et al. 2010<br><br>EA<br><br>Study quality: 2nd tier | Horn antenna, 10 GHz<br><br>2 conditions: sham exposure and exposure (PD 0.214 mW/cm², SAR 0.014 W/kg) (measurement, calculation/simulation)<br><br>Whole body exposure<br><br>Exposure duration: 2 h/day for 45 days | PD 0.214 mW/cm²<br><br>SAR 0.014 W/kg | PD 2.14 W/m²<br><br>SAR 0.014 W/kg | below<br><br>below<br><br>**Exposure below limit values***|
| *The SAR values occurring in the internal body tissues of animals can be assumed to be significantly higher than the corresponding SAR values occurring in humans at the same incident power flux density. Therefore, it cannot be ruled out that the SAR values in internal body tissues in animals in this study were greater than those in humans if the limit value was exhausted. | | | | |
| Schrot and Hawkins 1974<br><br>EA<br><br>Study quality: 1st tier | Ellipsoidal antenna, 3.0 GHz, CW<br><br>16 conditions: combinations of different power densities (0.35-2.6 W/cm²) x durations (30-240 s), (measurement, calibration)<br><br>Head exposure<br><br>Exposure duration: 30, 60, 120 and 240 s<br><br>Remarks: animals were exposed individually with head facing the antenna and aligned with the longitudinal axis of the antenna | 0.35-2.6 W/cm² | 3500-26,000 W/m² | above<br><br>**Exposure above limit values*** |
| *Relevant temperature increase in animals certain | | | | |
| Sisodia et al. 2013<br><br>EA<br><br>Study quality: 2nd tier | Pyramidal horn antenna, 10 GHz<br><br>2 conditions: sham exposure and exposure (PD 0.25 mW/cm², SAR 0.1790 W/kg) (measurement, calculation/simulation)<br><br>Whole body exposure (probably; not explicitly described)<br><br>Exposure duration: 2 hours/day for 30 days | PD 0.25 mW/cm²<br><br>SAR 0.1790 W/kg | PD 2.5 W/m²<br><br>SAR 0.1790 W/kg | below<br><br>above*<br><br>**Exposure above limit values*** |
| *The SAR values occurring in the internal body tissues of animals can be assumed to be significantly higher than the corresponding SAR values occurring in humans at the same incident power flux density. Therefore, it cannot be ruled out that the SAR values in internal body tissues in animals in this study were greater than those in humans if the limit value was exhausted. | | | | |

| Authors, study design | Exposure specifications | Relevant exposure metrics | Conversion | Comparison to limit values |
|---|---|---|---|---|
| **Other topics** | | | | |
| Verma et al. 2021<br><br>EA<br><br>Study quality: 2nd tier | Horn antenna, 10.0 GHz, CW<br><br>3 conditions: sham exposure and 2 exposure groups: group 1 (PD 5 mW/cm² (5.23 ± 0.25 mW/cm²), SAR 0.5 ± 0.02 W/kg (whole body)), group 2 (PD 10 mW/cm² (10.01 ± 0.15 mW/cm²), SAR 1.0±0.03 W/kg (whole body)) (measurement, calculation/simulation)<br><br>Whole body exposure<br><br>Exposure duration: 3 h/day for 30 days<br><br>Remarks: rats were exposed in individual cages (n=5) placed together vertically below the antenna | group 1:<br>PD 5 mW/cm² (5.23 ± 0.25 mW/cm²)<br><br>SAR 0.5 ± 0.02 W/kg (whole body))<br><br>group 2:<br>PD 10 mW/cm² (10.01 ± 0.15 mW/cm²)<br><br>SAR 1.0 ± 0.03 W/kg (whole body)) | PD 50 W/m² (52.3 ± 2.5 W/m²)<br><br>SAR 0.5 ± 0.02 W/kg (whole body))<br><br>PD 100 W/m² (100.01 ± 1.5 W/m²)<br><br>SAR 1.0±0.03 W/kg (whole body)) | above<br><br>above<br><br>above<br><br>above<br><br>**Exposure above limit values** |
