# Supplementary material for: Physiological and health-related effects of 5G-relevant radiofrequency fields: A systematic review of experimental studies in humans and mammals
Source: PLoS One. 2026 Nov 4;21(11):e0361104. doi: 10.1371/journal.pone.0361104 (PMC13734471; doi:10.1371/journal.pone.0361104)
Supplement: Supplementary file 10 [file pone.0361104.s010.pdf]

S10 Evidence Synthesis. Assessment of confidence in the body of evidence synthesis on topic level.

Endpoint	Initial confidence (EA and HCT)	Factors decreasing confidence	Factors increasing confidence	Confidence in the body of evidence	Direction (effect or no effect)	Level of evidence for health effect
**Structural changes**	High	Risk of bias, unexplained inconsistency	-	Low	Effect	Only for changes in hippocampus: low (above limit values)
**Cognition**	High	Risk of bias, unexplained inconsistency	-	Low	No effect	Only for learning and spatial memory: inadequate
**Behavior**	High	Evidence synthesis not applicable due to heterogeneity	-	-	-	n.a.
**Biochemical changes**	High	Evidence synthesis not applicable due to heterogeneity	-	-	-	n.a.
**Brain activity**	High	Evidence synthesis not applicable due to risk of bias and small number of studies	-	-	-	n.a.
**Gene/protein expression**	High	Evidence synthesis not applicable due to heterogeneity, risk of bias and small number of studies	-	-	-	n.a.
**Alzheimer's disease therapy**	High	Evidence synthesis not applicable due to single study	-	-	-	n.a.

Endpoint	Initial confidence (EA and HCT)	Factors decreasing confidence	Factors increasing confidence	Confidence in the body of evidence	Direction (effect or no effect)	Level of evidence for health effect
**Cytokine secretion**	High	Risk of bias	-	Moderate	Effect	Moderate
**Antibody production**	High	Evidence synthesis not applicable due to heterogeneity	-	-	-	n.a.
**Effects on spleen**	High	Evidence synthesis not applicable due to heterogeneity, risk of bias and small number of studies	-	-	-	n.a.
**Thermoregulation**	High	Evidence synthesis not applicable due to risk of bias and small number of studies	-	-	-	n.a.
**Heart rate with temperature changes**	High	Evidence synthesis not applicable due to risk of bias and small number of studies	-	-	-	n.a.

Endpoint	Initial confidence (EA and HCT)	Factors decreasing confidence	Factors increasing confidence	Confidence in the body of evidence	Direction (effect or no effect)	Level of evidence for health effect
**Male fertility**	High	Evidence synthesis not applicable due to heterogeneity	-	-	-	n.a.
**Fetus & teratogenicity**	High	Evidence synthesis not applicable due to heterogeneity, risk of bias and the small number of studies	-	Very low	Effect	Inadequate
**General blood parameters**	High	Evidence synthesis not applicable due to heterogeneity	-	-	-	n.a.
**Hormonal changes**	High	Evidence synthesis not applicable due to heterogeneity	-	-	-	n.a.
**Oxidative stress**	High	Evidence synthesis not applicable due to heterogeneity	-	-	-	n.a.

Endpoint	Initial confidence (EA and HCT)	Factors decreasing confidence	Factors increasing confidence	Confidence in the body of evidence	Direction (effect or no effect)	Level of evidence for health effect
**Genotoxicity**	High	Evidence synthesis not applicable due to heterogeneity, risk of bias and the small number of studies	-	-	-	n.a.
**Eyes**	High	Evidence synthesis not applicable due to single study	-	-	-	n.a.
**Bones**	High	Evidence synthesis not applicable due to single study	-	-	-	n.a.
**Mortality (positive)**	High	Evidence synthesis not applicable due to heterogeneity	-	-	-	n.a.
**Mortality (negative)**	High	Evidence synthesis not applicable due to risk of bias and the small number of studies	-	-	-	n.a.
**Skin**	High	Evidence synthesis not applicable due to single study	-	-	-	n.a.
