# Supplementary material for: Physiological and health-related effects of 5G-relevant radiofrequency fields: A systematic review of experimental studies in humans and mammals
Source: PLoS One. 2026 Nov 4;21(11):e0361104. doi: 10.1371/journal.pone.0361104 (PMC13734471; doi:10.1371/journal.pone.0361104)
Supplement: Supplementary file 11 [file pone.0361104.s011.pdf]

S11 Search String. Link to search string for search in EMF-Portal for newly published articles.

The database was searched using the following criteria:

Topics: experimental studies, epidemiological studies, medical applications

Frequency ranges: radiofrequency (≥ 10 MHz), mobile communications

Time span: last 929 days (time span between last search (01.09.2023) and 18.03.2026)

Link: https://www.emf-portal.org/de/article/search/v2/results?query=%28topic%3Dexperimental+OR+topic%3Depidemiological+OR+topic%3Dmedical_application%29+AND+%28frequencyRange%3Dradio_frequency+OR+frequencyRange%3Dmobile_communications%29+AND+entry%3D929
