# Supplementary material for: Physiological and health-related effects of 5G-relevant radiofrequency fields: A systematic review of experimental studies in humans and mammals
Source: PLoS One. 2026 Nov 4;21(11):e0361104. doi: 10.1371/journal.pone.0361104 (PMC13734471; doi:10.1371/journal.pone.0361104)
Supplement: Supplementary file 12 [file pone.0361104.s012.pdf]

S12 Reference List. Reference list of newly identified articles.

1. Bektas H, Yildirim S, Cakir S, Dogu S, Altindag F. Ameliorative Role of Coenzyme Q10 in RF Radiation-Associated Testicular and Oxidative Impairments in a 3.5-GHz Exposure Model. Bioelectromagnetics. 2026;47(1): e70043. doi:10.1002/BEM.70043

2. Bodin R, Seewooruttun C, Corona A, Delanaud S, Pelletier A, Villégier AS. Sex-dependent impact of perinatal 5G electromagnetic field exposure in the adolescent rat behavior. Environ Sci Pollut Res Int. 2023;30(53): 113704–17. doi:10.1007/S11356-023-30256-7

3. Emre M, Karamazi Y, Emre T, Varan NE, Toyran T, Yücebilgiç G. Effect of high-frequency radiofrequency (6 GHz) electromagnetic radiation on oxidative stress and kidney morphology. Toxicol Ind Health. 2026;42: 124–136. doi:10.1177/07482337261422845

4. Gelenli Dolanbay E, Mert T, Caliskan Bender G, Bektas H, Uslu U, Fernandez-Rodriguez CE, et al. Male Reproductive and Cellular Damage After Prenatal 3.5 GHz Radiation Exposure: One-Year Postnatal Effects. Ann N Y Acad Sci. 2025;1554(1): 140–52. doi:10.1111/nyas.70116

5. Gelenli Dolanbay E, Mert T, Kurtoglu RN, Uslu U, Dasdag S. Prenatal Exposure to 3.5 GHz Radiofrequency Radiation and Long-Term Skin Histomorphometry: An 18-Month Experimental Rat Study. Medeni Med J. 2025;40(4): 209–16. doi:10.4274/MMJ.GALENOS.2025.48323

6. Ijima E, Nagai A, Li K, Hikage T, Kamizawa N, Hidaka E, et al. Histological and inflammatory effects of 26.5 GHz quasi-millimeter wave exposure on rat skin. Front Public Health. 2025;13: 1580155. doi: 10.3389/fpubh.2025.1580155

7. Jamal L, Michelant L, Delanaud S, Hugueville L, Mazet P, Lévêque P, et al. Autonomous nervous system responses to environmental-level exposure to 5G's first deployed band (3.5 GHz) in healthy human volunteers. Exp Physiol. 2024;109(12): 2122–33. doi: 10.1113/EP092083

8. Karamazı Y, Emre M, Uçar S, Aksoy G, Emre T, Tokuş M. Effect of 6 GHz radiofrequency electromagnetic field on the development of fetal bones. Electromagn Biol Med. 2025;44(1): 17–25. doi:10.1080/15368378.2024.2438608

9. Kojima M, Tasaki T, Kamijo T, Hada A, Suzuki Y, Kik A, et al. Investigation of the Ocular Response and Corneal Damage Threshold of Exposure to 28 GHz Quasi-millimeter Wave Exposure. Health Phys. 2025;128(6): 487–96. doi:10.1097/HP.0000000000001951

10. Lameth J, Royer J, Martin A, Marie C, Arnaud-Cormos D, Lévêque P, et al. Repeated Head Exposures to a 5G-3.5 GHz Signal Do Not Alter Behavior but Modify Intracortical Gene Expression in Adult Male Mice. Int J Mol Sci. 2025;26(6): 2459. doi:10.3390/IJMS26062459/S1

11. Li Y, Yao B, Men J, Pang Y, Gao J, Bai Y, et al. Oxidative stress and energy metabolism in male reproductive damage from single and combined high-power microwave exposure at 1.5 and 4.3GHz. Reproductive Toxicology. 2025;132: 108759. doi:10.1016/j.reprotox.2024.108759

12. Liu S, Cheng Y, Zhang X, Shen Y, Wu S, Chen Y, et al. Assessing effects of microwave electromagnetic field in APP/PS1 mice by in vivo GABA-weighted imaging via variable delay multipulse-chemical exchange saturation transfer MRI. Neurochem Int. 2026;194: 106131. doi:10.1016/j.neuint.2026.106131

13. Matsumoto A, Endo I, Ijima E, Hirata A, Kodera S, Ichiba M, et al. Single exposure to near-threshold 5G millimeter wave modifies restraint stress responses in rats. Environ Health Prev Med. 2025;30: 33. doi:10.1265/EHPM.24-00321

14. Michelant L, Baz T, Carrie A, Hugueville L, Lévêque P, Selmaoui B. Millimeter-wave high frequency 5G (26 GHz) electromagnetic fields do not modulate human brain electrical activity. Environ Res. 2026;289: 123349. doi:10.1016/J.ENVRES.2025.123349

15. Michelant L, Hugueville L, Lévêque P, Selmaoui B. No measurable impact of acute 26 GHz 5G exposure on salivary stress markers in healthy adults. Environ Res. 2026;290: 123439. doi:10.1016/J.ENVRES.2025.123439

16. Nikitina VN, Kalinina NI, Dubrovskaya EN, Plekhanov VP, Kovshov AA. Experimental Study of Animal Behavior under the Influence of the Electromagnetic Field of the 5G Mobile Communication Standard. Biology Bulletin. 2024;51(11): 3473–80. doi:10.1134/S1062359024701875

17. Obajuluwa AO, Lech JC, Amina D, Onwuka CC, Bolarinwa RA, Obajuluwa TM, et al. Single and combined neurotoxic, cytotoxic and genotoxic effects of 5GHz MIMO waves and computed tomography irradiation in male Wistar rats. Egyptian Journal of Basic and Applied Sciences. 2023;10(1): 835–45. doi:10.1080/2314808X.2023.2282243

18. Samoylova A V., Gostyukhina AA, Bolshakov MA, Yartsev V V., Evseeva SS, Doroshenko OS, et al. Combined Effects of Bone Marrow Cells and Pulsed Microwaves on Thermally Damaged Skin of Laboratory Rats. Bulletin of Experimental Biology and Medicine 2024 178:1. 2024;178(1): 91–5. doi:10.1007/S10517-024-06288-5

19. Seewooruttun C, Bouguila B, Corona A, Delanaud S, Bodin R, Bach V, et al. 5G Radiofrequency Exposure Reduces PRDM16 and C/EBP β mRNA Expression, Two Key Biomarkers for Brown Adipogenesis. Int J Mol Sci. 2025;26(6): 2792. doi:10.3390/IJMS26062792/S1

20. Sousouri G, Eicher C, D'Angelo RM, Billecocq M, Fussinger T, Studler M, et al. 5G radio-frequency-electromagnetic-field effects on the human sleep electroencephalogram: A randomized controlled study in CACNA1C genotyped volunteers. Neuroimage. 2025; 317. doi:10.1016/j.neuroimage.2025.121340

21. Sun L, Wang X, Ren K, Yao C, Wang H, Xu X, et al. Compound exposure of 2.8 GHz and 9.3 GHz microwave causes learning and memory impairment in rats. Heliyon. 2025;11(1): e41626. doi:10.1016/J.HELIYON.2025.E41626

22. Syed Taha SMA, Jaffar FHF, Hairulazam A, Vijay S, Jamaludin N, Zulkifli AF, et al. Prolonged 3.5 GHz and 24 GHz RF-EMF Exposure Alters Testicular Immune Balance, Apoptotic Gene Expression, and Sperm Function in Rats. Biomedicines. 2025;13(10): 2471. doi:10.3390/BIOMEDICINES13102471/S1

23. Torkan A, Zoghi M, Foroughimehr N, Jaberzadeh S. The Effect of 5G Mobile Phone Electromagnetic Exposure on Corticospinal and Intracortical Excitability in Healthy Adults: A Randomized Controlled Pilot Study. Brain Sci. 2025;15: 1134. doi:10.3390/BRAINSCI1511113424.

24. Wang X, Zhao X, Xu J, Li M, Sun B, Gao A, et al. The Impact of 9.375 GHz Microwave Radiation on the Emotional and Cognitive Abilities of Mice. Int J Mol Sci. 2025;26: 2871. doi:10.3390/IJMS26072871

25. Wang X, Zhou G, Lin J, Zhang Z, Qin T, Guo L, et al. Effects of 4.9 GHz Radiofrequency Field Exposure on Brain Metabolomic and Proteomic Characterization in Mice. Biology (Basel). 2024;13(10): 806. doi:10.3390/BIOLOGY13100806/S1

26. Yilmaz H, Tümkaya L, Mercantepe T, Yılmaz A, Gül F, Suzan ZT. Effects of 5 G mobile phone network electromagnetic field exposure on testicular endoplasmic reticulum stress and the protective role of coenzyme Q10. Arch Med Res. 2025;56: 103157. doi:10.1016/j.arcmed.2024.103157

27. Zhaowen Z, Ling G, Guiqiang Z, Jiajin L, Tongzhou Q, Jiangyi L, et al. Effects of paternal 5G RFR exposure on health of male offspring mice. Reproductive Toxicology. 2026;140: 109139. doi:10.1016/j.reprotox.2025.109139

28. Zhou G, Guo L, Qin T, Zhang Z, Gao P, Wang X, et al. Single and combined microwave induced anxiety-like behavior via NLRP3 dependent neuronal pyroptosis in hippocampus and amygdala in mice. Ecotoxicol Environ Saf. 2026;310: 119818. doi:10.1016/j.ecoenv.2026.119818
