# Supplementary material for: Physiological and health-related effects of 5G-relevant radiofrequency fields: A systematic review of experimental studies in humans and mammals
Source: PLoS One. 2026 Nov 4;21(11):e0361104. doi: 10.1371/journal.pone.0361104 (PMC13734471; doi:10.1371/journal.pone.0361104)
Supplement: Supplementary file 13 [file pone.0361104.s013.pdf]

S13 Reference List. References of supplement information (S2, S8, S9, S15, S1 Tables, S1 Table).

1. Adang D, Remacle C, Vander Vorst A. Results of a Long-Term Low-Level Microwave Exposure of Rats. IEEE Trans Microw Theory Tech. 2009;57: 2488–2497. doi:10.1109/TMTT.2009.2029667

2. Akdag MZ, Sert C, Erdinc L, Dasdag S, Buyukbayram H, Celik MS, et al. The evaluation of the role of microwave and vitamins on rat eyes related to lipid peroxidation and tissue damage. Biotechnology & Biotechnological Equipment. 2002;16: 131–137. doi:10.1080/13102818.2002.10819167

3. Akdag MZ, Sert C, Kaya H, Dasdag S, Celik MS. Effects of whole-body chronic microwave exposure on some hormones of variously treated rats. Biochem Arch. 1999a;15: 345–350.

4. Akdag Z, Celik MS, Ketani A, Nergiz Y, Deniz M, Dasdag S. Effect of chronic low-intensity microwave radiation on sperm count, sperm morphology, and testicular and epididymal tissues of rats. Electro- and Magnetobiology. 1999b;18: 133–145.

5. ANSES. Opinion on “Population exposure to electromagnetic fields associated with the deployment of 5G communication technology and the related health effects” updating the opinion of 12 April 2021. 2022. Available: https://www.anses.fr/en/system/files/AP2019SA0006EN-2.pdf

6. Appleton B, Hirsch SE, Brown PVK. Investigation of single-exposure microwave ocular effects at 3000 MHz. Ann N Y Acad Sci. 1975;247: 125–134. doi:10.1111/J.1749-6632.1975.TB35989.X

7. Baranski S. Effect of Chronic Microwave Irradiation on Blood Forming System of Guinea Pigs and Rabbits. Aerosp Med. 1971;42: 1196.

8. Baranski S. Histological and histochemical effect of microwave irradiation on the central nervous system of rabbits and guinea pigs. Am J Phys Med. 1972a;51: 182–191.

9. Baranski S. Effect of microwaves on the reactions of the WBC system. Acta Physiol Pol. 1972b;23: 685–695.

10. Baranski S, Edelwejn Z. Electroencephalographic and morphological studies on the effect of microwaves on the central nervous system. Acta Physiol Pol. 1967;18: 517–532.

11. Bektas H, Algul S, Altindag F, Yegin K, Akdag MZ, Dasdag S. Effects of 3.5 GHz radiofrequency radiation on ghrelin, nesfatin-1, and irisin level in diabetic and healthy brains. J Chem Neuroanat. 2022;126: 102168. doi:10.1016/j.jchemneu.2022.102168

12. Bektas H, Dasdag S, Nalbant A, Akdag MB, Demir C, Kavak S. 3.5 GHz radiofrequency radiation may affect biomechanics of bone and muscle of diabetics. Biotechnology & Biotechnological Equipment. 2023;37: 2199096. doi:10.1080/13102818.2023.2199096

13. Bertharion G, Servantie B, Joly R. Electro-Cortico-Graphic Modifications After Exposure to Microwave Fields, on the White Rat. J Microw Power. 1971;6: 62–63. doi:10.1080/00222739.1971.11688786

14. Blick DW, Adair ER, Hurt WD, Sherry CJ, Walters TJ, Merritt JH. Thresholds of microwave-evoked warmth sensations in human skin. Bioelectromagnetics. 1997;18: 403–409. doi:10.1002/(SICI)1521-186X(1997)18:6<403::AID-BEM1>3.0.CO;2-6

15. Cain CA, Rissmann WJ. Mammalian auditory responses to 3.0 GHz microwave pulses. IEEE Trans Biomed Eng. 1978;25: 288–293. doi:10.1109/TBME.1978.326343

16. Chiang H, Yao GD. Effects of pulsed microwave radiation pre-and post-natally on the developing brain in mice. Journal of Bioelectricity. 1987;6: 197–204.

17. Chiang H, Yao GD, Zhou S. Effects of microwave exposure at various power densities on mitochondrial marker enzymes in mouse brains. Journal of Bioelectricity. 1984;3: 361–366.

18. Chiaraviglio L, Elzanaty A, Alouini MS. Health risks associated with 5G exposure: A view from the communications engineering perspective. IEEE Open Journal of the Communications Society. 2021;2: 2131–2179. doi:10.1109/OJCOMS.2021.3106052

19. Crouzier D, Dabouis V, Gentilhomme E, Vignal R, Bourbon F, Fauvelle F, et al. Chronic Electromagnetic Exposure at Occupational Safety Level Does Not Affect the Metabolic Profile nor Cornea Healing after LASIK Surgery. J Ophthalmol. 2014; 762364. doi:10.1155/2014/762364

20. D’Andrea JA, Thomas A, Hatcher DJ. Rhesus monkey behavior during exposure to high-peak-power 5.62-GHz microwave pulses. Bioelectromagnetics. 1994;15: 163–176. doi:10.1002/bem.2250150207

21. Dasdag S, Sert C, Akdag MZ, Oflazoglu HD. Effects of microwaves and ELF magnetic field on the phagocytic activity of variously treated rat macrophages. Electro- and Magnetobiology. 2001;20: 177–184. doi:10.1081/JBC-100104141

22. de Lorge JO. Operant behavior and colonic temperature of Macaca mulatta exposed to radio frequency fields at and above resonant frequencies. Bioelectromagnetics. 1984;5: 233–246. doi:10.1002/bem.2250050211

23. de Lorge JO, Ezell CS. Observing-responses of rats exposed to 1.28- and 5.62-GHz microwaves. Bioelectromagnetics. 1980;1: 183–198. doi:10.1002/bem.2250010208

24. De Seze R, Poutriquet C, Gamez C, Maillot-Maréchal E, Robidel F, Lecomte A, et al. Repeated exposure to nanosecond high power pulsed microwaves increases cancer incidence in rat. PLoS One. 2020;15: e0226858–e0226858. doi:10.1371/JOURNAL.PONE.0226858

25. Deichmann WB, Stephens Jr FH, Keplinger M, Lampe KF. Acute Effects of Microwave Radiation on Experimental Animals (24,000 Megacycles). Journal of Occupational Medicine. 1959;1: 369–381.

26. Deichmann WB, Miale J, Landeen K. Effect of Microwave Radiation on hemopoietic System of Rat. Toxicol Appl Pharmacol. 1964;6: 71–77. doi:10.1016/0041-008X(64)90023-7

27. Di Ciaula A. Towards 5G communication systems: Are there health implications? Int J Hyg Environ Health. 2018;221: 367–375. doi:10.1016/J.IJHEH.2018.01.011

28. Drobakhin O, Magro V, Kosharnyi V, Rutgaizer V, Abdul-Ohly L. Microscopic Changes in the Myocardium of the Heart Wall of Rats under the Influence of Microwave Electromagnetic Radiation under the Conditions of Modeling Hypothyroidism. 2020 IEEE Ukrainian Microwave Week (UkrMW), Kharkiv, Ukraine. 2020; 586–589. doi:10.1109/UkrMW49653.2020.925266729.

29. Drobakhin OO, Magro VI, Koshamyi V V, Rutgaizer VH, Abdul-Ohly L V. The influence of microwave electromagnetic radiation on rat heart morphogenesis during thyroidectomy. Regul Mech Biosyst. 2021;12: 136–144. doi:10.15421/022121

30. European Parliament: Directorate-General for Parliamentary Research Services. Health impact of 5G - current state of knowledge of 5G-related carcinogenic and reproductive/developmental hazards as they emerge from epidemiological studies and in vivo experimental studies. European Parliament. 2021. doi:10.2861/657478

31. European Parliament: Directorate-General for Parliamentary Research Services. Environmental impact of 5G - A literature review of effects of radio-frequency electromagnetic field exposure of non-human vertebrates, invertebrates and plants. European Parliament. 2021. doi:10.2861/318352

32. Fesenko EE, Makar VR, Novoselova EG, Sadovnikov VB. Microwaves and cellular immunity. I. Effect of whole body microwave irradiation on tumor necrosis factor production in mouse cells. Bioelectrochemistry and Bioenergetics. 1999;49: 29–35. doi:10.1016/s0302-4598(99)00058-6

33. Fontani V, Rinaldi S, Aravagli L, Mannu P, Castagna A, Margotti ML. Noninvasive radioelectric asymmetric brain stimulation in the treatment of stress-related pain and physical problems: psychometric evaluation in a randomized, single-blind placebo-controlled, naturalistic study. Int J Gen Med. 2011;4: 681–686. doi:10.2147/IJGM.S24628

34. Frei MR, Jauchem JR, Heinmets F. Thermoregulatory responses of rats exposed to 9.3-GHz radiofrequency radiation. Radiat Environ Biophys. 1989;28: 67–77. doi:10.1007/BF01209724

35. Glushkova O V, Novoselova EG, Cherenkov DA, Novoselova T V, Lunin SM, Khrenov MO, et al. Effect of super-high frequency electromagnetic radiation on the immune status of mice in endotoxic shock. Biophysics (Oxf). 2007;52: 508–511. doi:10.1134/S0006350907050107

36. Gostyukhina AA, Samoylova A V, Bolshakov MA, Mochalova VM, Zaitsev K V, Kutenkov OP, et al. Stimulation of Burn Wound Healing in Rats by Nanosecond Repetitive Pulsed Microwave Radiation. Biol Bull. 2022;49: 532–537. doi:10.1134/S1062359022050089

37. Health Council of the Netherlands. Background document to the advisory report 5G and health. publication no 2020/16Ae. The Hague; 2020. Available: https://www.healthcouncil.nl/binaries/healthcouncil/documents/advisory-reports/2020/09/02/5g-and-health/Background-document-to-the-advisory-report-5G-and-health.pdf

38. Hinrikus H, Koppel T, Lass J, Orru H, Roosipuu P, Bachmann M. Possible health effects on the human brain by various generations of mobile telecommunication: a review based estimation of 5G impact. Int J Radiat Biol. 2022;98: 1210–1221. doi:10.1080/09553002.2022.2026516

39. Hirsch SE, Appleton B, Fine BS, Brown P V. Effects of repeated microwave irradiations to the albino rabbit eye. Invest Ophthalmol Vis Sci. 1977;16: 315–319. Available: https://iovs.arvojournals.org/article.aspx?articleid=2175452

40. Ijima E, Li K, Hikage T, Nagai A, Murakami Y, Arima T, et al. Intensity-dependent Temperature Rise Induced by Local Exposure to 26.5 GHz Quasi-Millimeter-Wave in Rat. In Vivo (Brooklyn). 2023a;37: 2092–2099. doi:10.21873/invivo.13306

41. Ijima E, Kodera S, Hirata A, Hikage T, Matsumoto A, Ishitake T, et al. Excessive whole-body exposure to 28 GHz quasi-millimeter wave induces thermoregulation accompanied by a change in skin blood flow proportion in rats. Front Public Health. 2023b;11: 1225896. doi:10.3389/fpubh.2023.1225896

42. Jamal L, Yahia-Cherif L, Hugueville L, Mazet P, Lévêque P, Selmaoui B. Assessment of Electrical Brain Activity of Healthy Volunteers Exposed to 3.5 GHz of 5G Signals within Environmental Levels: A Controlled-Randomised Study. Int J Environ Res Public Health. 2023;20: 6793. doi:10.3390/ijerph20186793

43. Jauchem JR, Frei MR, Heinmets F. Heart rate changes due to 5.6-GHz radiofrequency radiation: relation to average power density. Proceedings of the Society for Experimental Biology and Medicine. 1984;177: 383–387. doi:10.3181/00379727-177-41960

44. Jensh RP. Studies of the teratogenic potential of exposure of rats to 6000 MHz microwave radiation. I. Morphologic analysis at term. Radiat Res. 1984a;97: 272–281.

45. Jensh RP. Studies of the teratogenic potential of exposure of rats to 6000 MHz microwave radiation. II. Postnatal psychophysiologic evaluations. Radiat Res. 1984b;97: 282–301.

46. Karipidis K, Mate R, Urban D, Tinker R, Wood A. 5G mobile networks and health—a state-of-the-science review of the research into low-level RF fields above 6 GHz. J Expo Sci Environ Epidemiol. 2021;31: 585–605. doi:10.1038/s41370-021-00297-6

47. Knyazeva IR, Medvedev MA, Kutenkov OP, Vasilev AV, Gorokhovsky AA, Rostov VV. The Features of the Stress-Reaction to Repetitive Nanosecond Microwave Pulses. 2018 20th International Symposium on High-Current Electronics (ISHCE), Tomsk. 2018; 94–97. doi:10.1109/ISHCE.2018.8521190

48. Kössler F, Lange F, Kupfer J, Rothe R. The action of microwave radiation of low intensity on body weight, spontaneous activity and blood values of mice undergoing long-term experiments. Z ges Hyg. 1981;27: 44–47.

49. Kumar S, Behari J, Sisodia R. Impact of microwave at X-band in the aetiology of male infertility. Electromagn Biol Med. 2012;31: 223–232. doi:10.3109/15368378.2012.700293

50. Kumar S, Behari J, Sisodia R. Influence of electromagnetic fields on reproductive system of male rats. Int J Radiat Biol. 2013;89: 147–154. doi:10.3109/09553002.2013.741282

51. Kumar S, Kesari KK, Behari J. Evaluation of genotoxic effects in male Wistar rats following microwave exposure. Indian J Exp Biol. 2010;48: 586–592. Available: http://nopr.niscpr.res.in/bitstream/123456789/9081/1/IJEB%2048%286%29%20586-592.pdf

52. Kumar S, Kesari KK, Behari J. Influence of microwave exposure on fertility of male rats. Fertil Steril. 2011;95: 1500–1502. doi:10.1016/j.fertnstert.2010.04.078

53. Liddle CG, Putnam JP, Ail JS, Lewis JY, Bell B, West MW, et al. Alteration of circulating antibody response of mice exposed to 9-GHz pulsed microwaves. Bioelectromagnetics. 1980;1: 397–404. doi:10.1002/bem.2250010406

54. Liddle CG, Putnam JP, Lewter OH, West M, Morrow G. Circulating antibody response of mice exposed to 9-GHz pulsed microwave radiation. Bioelectromagnetics. 1986;7: 91–94. doi:10.1002/bem.2250070110

55. Liu JJ, Zhang HY, Chen X, Zhang GB, Lin JK, Feng H, et al. 20-Hydroxyecdysone Improves Neuronal Differentiation of Adult Hippocampal Neural Stem Cells in High Power Microwave Radiation-Exposed Rats. Biomedical and Environmental Sciences. 2022;35: 504–517. doi:10.3967/bes2022.068

56. Manikowska E, Luciani JM, Servantie B, Czerski P, Obrenovitch J, Stahl A. Effects of 9.4 GHz microwave exposure on meiosis in mice. Experientia. 1979;35: 388–390. doi:10.1007/BF01964370

57. McAfee RD, Ortiz-Lugo R, Bishop R, Gordon R. Safety of 9.3-GHz microwave radiant heating for possible caloric supplement and medical treatment. Journal of Microwave Power and Electromagnetic Energy. 1985;20: 13–16. doi:10.1080/16070658.1985.11720275

58. Mickley GA, Cobb BL, Farrell ST. Brain hyperthermia alters local cerebral glucose utilization: a comparison of hyperthermic agents. Int J Hyperthermia. 1997;13: 99–114. doi:10.3109/02656739709056434

59. Mikolajczyk H, Kamedula M, Ruppe I, Eggert S. Thermal Effects in Rats exposed to Heat at 40 degress C or to electromagnetic Fields. J Therm Biol. 1991;16: 115–119. doi:10.1016/0306-4565(91)90008-P

60. Moussa SA. Oxidative Stress in Rats Exposed to Microwave Radiation. Rom J Biophys. 2009;19: 149–158. Available: https://www.rjb.ro/articles/240/art07Moussa.pdf

61. Novoselova EG, Fesenko EE, Makar VR, Sadovnikov VB. Microwaves and cellular immunity. II. Immunostimulating effects of microwaves and naturally occurring antioxidant nutrients. Bioelectrochemistry and Bioenergetics. 1999;49: 37–41. doi:10.1016/s0302-4598(99)00059-8

62. Novoselova EG, Glushkova OV, Khrenov MO, Novoselova TV, Lunin SM, Fesenko EE. Extremely low-level microwaves attenuate immune imbalance induced by inhalation exposure to low-level toluene in mice. Int J Radiat Biol. 2017;93: 535–543. doi:10.1080/09553002.2017.1270473

63. Novoselova EG, Ogay VB, Sorokina OV, Glushkova OV, Sinotova OA, Fesenko EE. The Production of Tumor Necrosis Factor in Cells of Tumor-Bearing Mice after Total-Body Microwave Irradiation and Antioxidant Diet. Electromagn Biol Med. 2004;23: 167–180. doi:10.1081/LEBM-200042320

64. Olazaran J, Gonzalez B, Lopez-Alvarez J, Castagna A, Osa-Ruiz E, Herrero-Cano V, et al. Motor effects of REAC in advanced Alzheimer's disease: results from a pilot trial. J Alzheimers Dis. 2013;36: 297–302. doi:10.3233/JAD-130077

65. Olazaran J, Gonzalez B, Osa-Ruiz E, Felipe-Ruiz S, Boyano I, Fontani V, et al. Motor effects of radio electric asymmetric conveyer in Alzheimer's disease: results from a cross-over trial. J Alzheimers Dis. 2014;42: 325–332. doi:10.3233/JAD-140417

66. Paulraj R, Behari J. Single strand DNA breaks in rat brain cells exposed to microwave radiation. Mutation Research - Fundamental and Molecular Mechanism of Mutagenesis. 2006;596: 76–80. doi:10.1016/j.mrfmmm.2005.12.006

67. Paulraj R, Behari J. Biochemical Changes in Rat Brain Exposed to Low Intensity 9.9 GHz Microwave Radiation. Cell Biochem Biophys. 2012a;63: 97–102. doi:10.1007/s12013-012-9344-3

68. Paulraj R, Behari J. Enzymatic alterations in developing rat brain cells exposed to a low-intensity 16.5 GHz microwave radiation. Electromagn Biol Med. 2012b;31: 233–242. doi:10.3109/15368378.2012.700295

69. Paulsson LE, Hamnerius Y, Hansson HA, Sjostrand J. Retinal damage experimentally induced by microwave radiation at 55 mW/cm2. Acta Ophthalmol. 1979;57: 183–197. doi:10.1111/j.1755-3768.1979.tb00483.x

70. Prausnitz S, Süsskind C. Effects of chronic microwave irradiation on mice. IRE Trans Biomed Electron. 1962; 104–108. doi:10.1109/tbmel.1962.4322972

71. Pu JS, Chen J, Yang YH, Bai YQ. The effects of 3000-MHz microwave irradiation on electroencephalic energy and energy metabolism in mouse brain. Electro- and Magnetobiology. 1997;16: 243–247.

72. Pyrpasopoulou A, Kotoula V, Cheva A, Hytiroglou P, Nikolakaki E, Magras IN, et al. Bone morphogenetic protein expression in newborn rat kidneys after prenatal exposure to radiofrequency radiation. Bioelectromagnetics. 2004;25: 216–227. doi:10.1002/bem.10185

73. Qin T, Liu L, Wang X, Guo L, Lin J, Du J, et al. Combined effects of EMP and RF field on emotional behavior in mice. Front Public Health. 2023;11: 1087161. doi:10.3389/fpubh.2023.1087161

74. Qin TZ, Wang X, Du JZ, Lin JJ, Xue YZ, Guo L, et al. Effects of radiofrequency field from 5G communications on the spatial memory and emotionality in mice. Int J Environ Health Res. 2024; 316–327. doi:10.1080/09603123.2022.2149708

75. Quock RM, Klauenberg BJ, Hurt WD, Merritt JH. Influence of microwave exposure on chlordiazepoxide effects in the mouse staircase test. Pharmacol Biochem Behav. 1994;47: 845–849. doi:10.1016/0091-3057(94)90286-0

76. Raslear TG, Akyel Y, Bates F, Belt M, Lu ST. Temporal bisection in rats: the effects of high-peak-power pulsed microwave irradiation. Bioelectromagnetics. 1993;14: 459–478. doi:10.1002/bem.2250140507

77. Ray S, Behari J. Physiological changes in rats after exposure to low levels of microwaves. Radiat Res. 1990;123: 199–202. doi:10.2307/3577545

78. Rifat F, Sharma A, Srivastava P, Patni S, Sisodia R. Protective Effects of Prunus Avium Extract on 10 Ghz Induced Damages in the Spleen of Swiss Albino Mice. Indian J Appl Res. 2013;3: 680–684. doi:10.36106/ijar

79. Rifat F, Sisodia R. Modulation of 10 GHz Microwaves induced Biochemical Changes in different Organs of Swiss Albino Mice by Prunus Domestica Fruit Extract. Int J Pharm Sci Res. 2017;8: 136–144. doi:10.13040/IJPSR.0975-8232.8(1).136-44

80. Rinaldi S, Mura M, Castagna A, Fontani V. Long-lasting changes in brain activation induced by a single REAC technology pulse in Wi-Fi bands. randomized double-blind fMRI qualitative study. Sci Rep. 2014;4: 5668. doi:10.1038/srep05668

81. Roberti B, Heebels GH, Hendricx JC, de Greef AH, Wolthuis OL. Preliminary investigations of the effects of low-level microwave radiation on spontaneous motor activity in rats. Ann N Y Acad Sci. 1975;247: 417–424. doi:10.1111/j.1749-6632.1975.tb36017.x

82. Rui G, Liu LY, Guo L, Xue YZ, Lai PP, Gao P, et al. Effects of 5.8 GHz microwave on hippocampal synaptic plasticity of rats. Int J Environ Health Res. 2022;32: 2247–2259. doi:10.1080/09603123.2021.1952165

83. Russell CL. 5 G wireless telecommunications expansion: Public health and environmental implications. Environ Res. 2018;165: 484–495. doi:10.1016/j.envres.2018.01.016

84. Şahingöz Yildirim AG, Karaca E, Gözen O, Durmaz B, Yildiz T, Yildirim N, et al. The Effect of Radiofrequency Waves on Pregnant Mice in Association with Genes Involved in Neuronal Migration. Journal of Clinical Obstetrics & Gynecology. 2022;32: 111–119. doi:10.5336/jcog.2021-84287

85. Schrot J, Hawkins TD. Lethal effects of 3000 MHz radiation on the rat. Radiat Res. 1974;59: 504–512.

86. Servantie B, Servantie AM, Etienne J. Synchronization of cortical neurons by a pulsed microwave field as evidenced by spectral analysis of electrocorticograms from the white rat. Ann N Y Acad Sci. 1975;247: 82–86. doi:10.1111/j.1749-6632.1975.tb35985.x

87. Shao BJ, Chiang H. The Effects of Microwaves on the Immune System in Mice. Journal of Bioelectricity. 1989;8: 1–10.

88. Sharma A, Sisodia R, Bhatnagar D, Saxena VK. Spatial memory and learning performance and its relationship to protein synthesis of Swiss albino mice exposed to 10 GHz microwaves. Int J Radiat Biol. 2014a;90: 29–35. doi:10.3109/09553002.2013.835883

89. Sharma A, Bhatnagar D, Sisodia R. 10 GHz microwaves induced biochemical, learning and memory alteration in swiss albino mice brain. Indian J Appl Res. 2014b;4: 57–61. Available: https://www.worldwidejournals.com/indian-journal-of-applied-research-(IJAR)/recent_issues_pdf/2014/May/10-ghz-microwaves-induced-biochemical-learning-and-memory-alterations-in-swiss-albino-mice-brain_May_2014_7267173219_0921574.pdf

90. Sharma A, Kesari KK, Saxena VK, Sisodia R. Ten gigahertz microwave radiation impairs spatial memory, enzymes activity, and histopathology of developing mice brain. Mol Cell Biochem. 2017a;435: 1–13. doi:10.1007/s11010-017-3051-8

91. Sharma A, Kesari KK, Saxena VK, Sisodia R. The influence of prenatal 10 GHz microwave radiation exposure on a developing mice brain. Gen Physiol Biophys. 2017b;36: 41–51. doi:10.4149/gpb_2016026

92. Sheikh AQ, Taghian T, Hemingway B, Cho H, Kogan AB, Narmoneva DA. Regulation of endothelial MAPK/ERK signalling and capillary morphogenesis by low-amplitude electric field. J R Soc Interface. 2013;10: 20120548. doi:10.1098/rsif.2012.0548

93. Shtemberg AS, Uzbekov MG, Shikhov SN, Bazyan AS, Chernyakov GM. Some neurotropic effects of low-intensity electromagnetic waves in rats with different typological characteristics of higher nervous activity. Neurosci Behav Physiol. 2001;31: 547–553. doi:10.1023/a:1010435217212

94. Simkó M, Mattsson MO. 5G Wireless Communication and Health Effects—A Pragmatic Review Based on Available Studies Regarding 6 to 100 GHz. Int J Environ Res Public Health. 2019;16: 3406. doi:10.3390/ijerph16183406

95. Sisodia R, Rifat F, Sharma A, Srivastava P, Sharma K. Effects of 10-GHz microwaves on hematological parameters in Swiss albino mice and their modulation by Prunus avium. J Environ Pathol Toxicol Oncol. 2013;32: 205–217. doi:10.1615/jenvironpatholtoxicoloncol.2013007891

96. Sofri T, Rahim HA, Abdulmalek M, Rani A, Hafizi Omar M, Najib M, et al. Health Effects of 5G Base Station Exposure: A Systematic Review. IEEE Access. 2022;10: 41639–41656. doi:10.1109/ACCESS.2021.3139385

97. Szmigielski S, Jeljaszewicz J, Wiranowska M. Acute staphylococcal infections in rabbits irradiated with 3-GHz microwaves. Ann N Y Acad Sci. 1975;247: 305–311. doi:10.1111/j.1749-6632.1975.tb36006.x

98. Szmigielski S, Pulverer G, Hryniewicz W, Janiak M. Inhibition of tumor growth in mice by microwave hyperthermia, Streptolysin S and Colcemide. Radio Sci. 1977a;12: 185–189. doi:10.1029/RS012i06Sp00185

99. Szmigielski S, Luczak M, Janiak M, Kobus M, Laskowska B, de Clercq E, et al. In vitro and in vivo inhibition of virus multiplication by microwave hyperthemia. Arch Virol. 1977b;53: 71–77. doi:10.1007/BF01314848

100. Teng J, de Tournai DC, Duhamel F, Vandervorst A. No nonthermal effect observed under microwave irradiation of spinal cord. IEEE Trans Microw Theory Tech. 1996;44: 1942–1948.

101. Thomas JR, Finch ED, Fulk DW, Burch LS. Effects of low-level microwave radiation on behavioral baselines. Ann N Y Acad Sci. 1975;247: 425–432. doi:10.1111/j.1749-6632.1975.tb36018.x

102. Thuroczy G, Kubinyi G, Bodo M, Bakos J, Szabo LD. Simultaneous response of brain electrical activity (EEG) and cerebral circulation (REG) to microwave exposure in rats. Rev Environ Health. 1994;10: 135–148. doi:10.1515/reveh.1994.10.2.135

103. Van Eeghem V, El Arfani A, Arta A, Walrave L, Pourkazemi A, Bentea E, et al. Selective changes in locomotor activity in mice due to low-intensity microwaves amplitude modulated in the EEG spectral domain. Neuroscience. 2017;359: 40–48. doi:10.1016/j.neuroscience.2017.06.056

104. Varma MM, Traboulay Jr EA. Biological effects of microwave radiation on the testes of Swiss mice. Experientia. 1975;31: 301–302. doi:10.1007/BF01922549

105. Verma S, Keshri GK, Karmakar S, Mani K V, Chauhan S, Yadav A, et al. Effects of Microwave 10 GHz Radiation Exposure in the Skin of Rats: An Insight on Molecular Responses. Radiat Res. 2021;196: 404–416. doi:10.1667/RADE-20-00155.1

106. Veyret B, Bouthet C, Deschaux P, de Seze R, Geffard M, Joussot-Dubien J, et al. Antibody responses of mice exposed to low-power microwaves under combined, pulse-and-amplitude modulation. Bioelectromagnetics. 1991;12: 47–56. doi:10.1002/bem.2250120107

107. Walani S, Bhatnagar D, Sisodia R. Biochemical alterations in cerebellum of Swiss albino mice after 10 GHz microwave exposure. Int J Adv Res. 2014;2: 708–716. Available: https://www.journalijar.com/uploads/487_IJAR-4110.pdf

108. Wang H, Zhao H, Li C, Dong J, Zhao J, Yue H, et al. Disrupted Topological Organization of Brain Network in Rats with Spatial Memory Impairments Induced by Acute Microwave Radiation. Brain Sci. 2023a;13: 1006. doi:10.3390/brainsci13071006

109. Wang H, Liu Y, Sun Y, Dong J, Xu X, Zhao X, et al. Changes in cognitive function, synaptic structure and protein expression after long-term exposure to 2.856 and 9.375 GHz microwaves. Cell Commun Signal. 2023b;21: 34. doi:10.1186/s12964-022-01011-1

110. Wang H, Liu Y, Sun Y, Zhao L, Dong J, Xu X, et al. Changes in rat spatial learning and memory as well as serum exosome proteins after simultaneous exposure to 1.5 GHz and 4.3 GHz microwaves. Ecotoxicol Environ Saf. 2022;243: 113983. doi:10.1016/j.ecoenv.2022.113983

111. Wood A, Mate R, Karipidis K. Meta-analysis of in vitro and in vivo studies of the biological effects of low-level millimetre waves. J Expo Sci Environ Epidemiol. 2021;31: 606–613. doi:10.1038/s41370-021-00307-7

112. Xue Y, Guo L, Lin J, Lai P, Rui G, Liu L, et al. Effects of 5.8 GHz Microwaves on Testicular Structure and Function in Rats. Biomed Res Int. 2022;2022: 301–314. doi:10.1155/2022/5182172

113. Yang H, Zhang Y, Wu X, Gan P, Luo X, Zhong S, et al. Effects of Acute Exposure to 3500 MHz (5G) Radiofrequency Electromagnetic Radiation on Anxiety-Like Behavior and the

Auditory Cortex in Guinea Pigs. Bioelectromagnetics. 2022;43: 106–118. doi:10.1002/BEM.22388

114. Yao C, Wang H, Sun L, Ren K, Dong J, Zhang J, et al. The Biological Effects of Compound Microwave Exposure with 2.8 GHz and 9.3 GHz on Immune System: Transcriptomic and Proteomic Analysis. Cells. 2022;11: 3849. doi:10.3390/cells11233849

115. Yin Y, Xu X, Gao Y, Wang J, Yao B, Zhao L, et al. Abnormal Expression of Connexin43 in Cardiac Injury Induced by S-Band and X-Band Microwave Exposure in Rats. J Immunol Res. 2021;2021: 3985697. doi:10.1155/2021/3985697

116. Zhang Y, Li Z, Gao Y, Zhang C. Effects of fetal microwave radiation exposure on offspring behavior in mice. J Radiat Res. 2015;56: 261–268. doi:10.1093/jrr/rru097

117. Zhao L, Yao C, Wang H, Dong J, Zhang J, Xu X, et al. Immune Responses to Multi-Frequencies of 1.5 GHz and 4.3 GHz Microwave Exposure in Rats: Transcriptomic and Proteomic Analysis. Int J Mol Sci. 2022;23: 6949. doi:10.3390/ijms23136949

118. Zhu R, Wang H, Xu X, Zhao L, Zhang J, Dong J, et al. Effects of 1.5 and 4.3 GHz microwave radiation on cognitive function and hippocampal tissue structure in Wistar rats. Sci Rep. 2021;11: 10061. doi:10.1038/s41598-021-89348-4
