# Supplementary material for: Physiological and health-related effects of 5G-relevant radiofrequency fields: A systematic review of experimental studies in humans and mammals
Source: PLoS One. 2026 Nov 4;21(11):e0361104. doi: 10.1371/journal.pone.0361104 (PMC13734471; doi:10.1371/journal.pone.0361104)
Supplement: Supplementary file 15 [file pone.0361104.s015.pdf]

S1 Tables. Extracted study data (1st and 2nd tier studies).

Abbreviations: 4-HNE = 4-hydroxynonenal, AM = Amplitude-modulated, AOPP = advanced oxidized protein product, APD = absorbed power density, BMP = bone morphogenetic protein, COX-2 = cyclooxygenase-2, CW = continuous wave, CS = citrate synthase, EA = experimental animal study, ECG = electrocardiogram, EEG = electroencephalography, G6PD = glucose-6-phospahte dehydrogenase, GA-BSA = glutaric-anhydride conjugated bovine serum albumin, GLUT1 = glucose transporter, GSM = Global System for Mobile Communications, HCT = human controlled trial, HK = hexokinase, IL-1beta = interleukin-1beta, LDH = lactate dehydrogenase, LPO = lipid peroxidation, NOS2/iNOS = nitric oxide synthase 2, ODC = ornithine decarboxylase, PD = power density, PKC = calcium-dependent protein kinase, pps = pulses per second, PW = pulsed wave, REAC = radio electric asymmetric conveyer, RBC = red blood cells, RMS = root mean square, ROS = reactive oxygen species, S100B = S100 calcium binding protein B, SAR = specific absorption rate, SOD = superoxide dismutase, SRBC = sheep red blood cells, TEM = transmission electron microscopy, TNF = tumor necrosis factor, WBC = white blood cells

Table A. Extracted data of studies on CNS, cognition, and behavior.

Authors, study design	Study groups	Exposure (Assessment method)	Endpoints	Results Conclusion according to authors (Effect categorization according to authors)	Remarks
**CNS, cognitive functions, and behavior**					
Bektas et al. 2022<br><br>EA<br><br>Study quality: 2nd tier	Wistar albino rats (healthy and with induced diabetes) (male, 8-10 weeks)<br><br>Exposure group 1: healthy rats (n=7), exposure group 2: diabetes rats (n=7), sham exposure group 1: healthy rats (n=7), sham exposure group 2: diabetes rats (n=7)	GSM signal generator (antenna), 3.5 GHz (5G), PW (rate 217 Hz; GSM)<br><br>2 conditions: sham exposure and exposure (28.1 V/m, PD 1.6 W/m², SAR 37 mW/kg (whole-body), SAR 323 mW/kg and SAR 195 mW/kg (for 1 g and 10 g averaging, respectively (gray matter)) (measurement, calculation/simulation)<br><br>Head exposure (1 cm distance to antenna)<br><br>Exposure duration: 2 h/day, 5 days/week for 1 month (30 days)<br><br>Remarks: 7 immobilized rats were simultaneously exposed in a plexiglass carousel with the antenna in the center	Energy homeostasis and redox balance in the brains (total antioxidant status, total oxidant status, hydrogen peroxide, nesfatin, ghrelin, and irisin levels (ELISA))<br><br>Histological changes (synaptophysin and degenerated neurons)	Exposure caused significant increases in ghrelin, total oxidant and hydrogen peroxide levels and significant decreases in nesfatin-1, irisin and total antioxidant levels compared to appropriate sham exposure. It was also observed that the exposure increased the number of degenerated neurons in the hippocampus (no statistics).<br><br>Conclusion: The data indicate that 3.5 GHz exposure caused changes in the energy metabolism in both healthy and diabetic rat brains.<br><br>(Effect found)	

Authors, study design	Study groups	Exposure (Assessment method)	Endpoints	Results<br>Conclusion according to authors<br>(Effect categorization according to authors)	Remarks
**CNS, cognitive functions, and behavior**					
Chiang et al. 1984<br><br>EA<br><br>Study quality: 2nd tier	C57BL mice<br>(sex and age not stated)<br><br>Exposure group 1: n=10,<br>exposure group 2: n=10,<br>exposure group 3: n=10,<br>exposure group 4: n=10,<br>exposure group 5: n=? (additional experiment),<br>exposure group 6: n=? (additional experiment)<br><br>control group: n=? (additional experiment),<br>sham exposure group: n=10	Radar, 3.0 GHz, PW (rate 935 pps, width 1.2 µs)<br><br>7 conditions: control group, sham exposure and 5 exposure conditions; group 1 (PD 0.1 mW/cm²), groups 2 and 6 (PD 0.5 mW/cm²), group 3 (PD 1 mW/cm²), group 4 (PD 5 mW/cm²), group 5 (PD 0.3 mW/cm²)<br>(measurement)<br><br>Whole body exposure<br><br>Exposure duration: 3 h<br><br>Remarks: animals exposed individually with their bodies parallel to the E-vector	Enzyme levels in the brain (succinate dehydrogenase, monoamine oxidase (as mitochondrial marker enzymes))	A significant decrease of enzyme level was found from 0.5 mW/cm² or higher for both enzymes in hippocampus and hypothalamus, respectively.<br><br>Conclusion: The data might indicate an effect of exposure on the energy metabolism in the brain.<br><br>(Effect found)	Additional data for 2.45 GHz available but not extracted

Authors, study design	Study groups	Exposure (Assessment method)	Endpoints	Results Conclusion according to authors (Effect categorization according to authors)	Remarks
**CNS, cognitive functions, and behavior**					
D'Andrea et al. 1994<br><br>EA<br><br>Study quality: 1st tier	Rhesus monkeys (*Macaca mulatta*) (male, approximately 48 months)<br><br>exposure group 1: n=4, exposure group 2: n=4, exposure group 3: n=4, exposure group 4: n=4, exposure group 5: n=4, exposure group 6: n=4, sham exposure group: n=4<br><br>The same 4 animals were used in every group.	Horn antenna, 5.620 GHz, PW (rate 100 pps, width 2.8 µs (radar) and 50 ns (Stanford linear energy doubler; SLED)<br><br>7 conditions: sham exposure and 6 exposure groups:<br>group 1 (radar pulse, SAR 2 W/kg (whole body), PD 56 W/cm² (peak)),<br>group 2 (radar pulse, SAR 4 W/kg, PD 128 W/cm²),<br>group 3 (radar pulse, SAR 6 W/kg, PD 277 W/cm²),<br>group 4 (SLED pulse, SAR 2 W/kg, PD 518 W/cm²),<br>group 5 (SLED pulse, SAR 4 W/kg, PD 1270 W/cm²),<br>group 6 (SLED pulse, SAR 6 W/kg, PD 2520 W/cm²)<br>(measurement, calibration)<br><br>Whole body exposure (focus on head)<br><br>Exposure duration: 20 min/session (probably one session for each exposure condition)<br><br>Remarks: animals partially restrained	Color discrimination task (lever responding, reaction time, and earned food pellets)	Lever responding, reaction time, and earned food pellets were reduced during microwave exposure at 4 and 6 W/kg compared to sham exposure.<br><br>Conclusion: Exposure to both radar and SLED pulses induced behavioral changes at a threshold between SARs of 2 and 4 W/kg.<br><br>(Effect found)	

**Authors, study design**	**Study groups**	**Exposure (Assessment method)**	**Endpoints**	**Results Conclusion according to authors (Effect categorization according to authors)**	**Remarks**
**CNS, cognitive functions, and behavior**					
De Lorge and Ezell 1980<br><br>EA<br><br>Study quality: 2nd tier	Long-Evans rats (male, age not stated)<br><br>Exposure group (different conditions): n=8<br>sham exposure group: n=8<br><br>The same 8 animals were used in every group.	Horn antenna, 5.62 GHz, PW (rate 662 pps, width 0.5 or 2 µs)<br><br>9 conditions: sham exposure and 8 exposure conditions (PD 7.5, 11.5, 16, 26, 31.5, 38.5, 42, and 48.5 mW/cm²)<br>(measurement, calculation/simulation)<br><br>Exposure of right side of the rat<br><br>Exposure duration: 1-7 x 40-min sessions at each PD, 1 session/day, 5 day/week	Operant behavior: observing-response task/vigilance task (two-lever task)	The frequency of correct detections decreased with increases in PD and from 26 mW/cm² (= average whole-body SAR 4.94 W/kg) the observing-response rate of all rats was consistently affected (no statistics).<br><br>Conclusion: The behavioral disruption produced by microwave exposure was probably related to thermal effects.<br><br>(Effect found)	Conditions with exposure to 1.28 GHz were not relevant for review and therefore not extracted.

Authors, study design	Study groups	Exposure<br>(Assessment method)	Endpoints	Results<br>Conclusion according to authors<br>(Effect categorization according to authors)	Remarks
**CNS, cognitive functions, and behavior**					
Jamal et al. 2023<br><br>HCT, cross-over study<br><br>Study quality: 2nd tier	Healthy volunteers (male, female, 26.6 ± 4.7 years)<br><br>Exposure group: n=34,<br>sham exposure group: n=34 (same group as exposed ones)	Horn antenna, 3.5 GHz (5G)<br>PW (width 577 µs, rate 4.6 ms)<br><br>2 conditions: sham exposure and 3.5 GHz exposure (2 V/m (rms; at the head; 1.5 V/m at the trunk),<br>PD 0.68 W/m² peak power,<br>SAR 0.037 ± 0.11 mW/kg (head-averaged),<br>SAR 0.008 ± 0.019 mW/kg (brain-averaged))<br>(measurement, calculation/simulation)<br><br>Head and trunk exposure<br><br>Exposure duration: approx. 25 min and 30 s<br><br>Remarks: volunteers were exposed in two sessions with a maximum of one week apart; (sham) exposure sessions comprised separate conditions with eyes open and eyes closed	Brain activity (EEG)	An overall non-significant change was observed in the studied brain waves, except for a few electrodes in the alpha, theta, and delta spectra. This change was translated into an increase or decrease in the power spectral densities, in response to the eyes-open and eyes-closed conditions.<br><br>Conclusion: 3.5 GHz exposure within the regulatory levels did not affect brain activity in healthy young adults.<br><br>(No effect found)	Data on ECG, electrodermal activity and saliva stress markers included in the study shall be published elsewhere

**Authors, study design**	**Study groups**	**Exposure (Assessment method)**	**Endpoints**	**Results Conclusion according to authors (Effect categorization according to authors)**	**Remarks**
**CNS, cognitive functions, and behavior**					
Mickley et al. 1997<br><br>EA<br><br>Study quality: 2nd tier	Sprague-Dawley rats (male, age not stated)<br><br>Exposure group: n=7, sham exposure group: n=7	Transmitter, 5.6 GHz, PW (width and rate not provided)<br><br>2 conditions: sham exposure and exposure (SAR 11.72 W/kg (averaged, head/neck) (calculation/simulation)<br><br>Head exposure<br><br>Exposure duration: 45 min<br><br>Remarks: pulse rate automatically controlled by using feedback from *in vivo* temperature probe, animals were anaesthetized	Brain metabolism (local cerebral glucose utilization in different brain areas)<br><br>Brain temperature (via tympanic temperature), rectal temperature, subcutaneous temperature (neck)	Hyperthermia (created by microwave or hot air) caused a general rise in brain metabolism (glucose utilization). Similar changes were produced by the two agents as long as steady state and rate of local brain temperature increase were well matched.<br><br>Conclusion: Microwaves exert their effects on brain metabolism through primarily thermal mechanisms.<br><br>(Effect found)	Aim of study was to compare temperature increase of 2°C caused by microwave exposure or hot air (additional experiments with hot air for comparison); different pilot studies performed for exposure optimization
Olazaran et al. 2014<br><br>HCT, cross-over study<br><br>Study quality: 2nd tier	Patients with probable or possible Alzheimer's disease (male, female, mean age 84.1 years, SD 7.9)<br><br>Exposure group: n=31 (group A) and n=29 (group B) sham exposure group: n=31 (group A) and n=29 (group B)	REAC-Emitter, 10.5 GHz, PW (single pulse)<br><br>2 conditions: sham exposure and exposure (7 µW/kg SAR) (no data provided on exposure validation)<br><br>Exposure duration: a single 250 ms burst<br><br>Remarks: pulse applied at the ear through metallic tip of REAC	Motor efficacy measures: Axial Movement Test, Rating Scale for Gait Evaluation in Cognitive Deterioration, Steps × Seconds Test<br><br>Safety surveillance: record of falls, infections, emergency room visits, hospitalizations, and any other potentially relevant events	Axial movements improved 7 and 11 days after exposure in group A (but not in group B) and immediately after sham exposure (results not entirely clear).<br><br>Conclusion: Albeit not conclusive regarding efficacy, the feasibility, acceptability, and safety results of the present trial warrant further research.<br><br>(No effect found)	

**Authors, study design**	**Study groups**	**Exposure (Assessment method)**	**Endpoints**	**Results Conclusion according to authors (Effect categorization according to authors)**	**Remarks**
**CNS, cognitive functions, and behavior**					
Paulraj and Behari 2006<br><br>EA<br><br>Study quality: 2nd tier	Wistar rats (male, 35 days)<br><br>Exposure group: n=6, sham exposure group: n=6	Horn antenna, 16.5 GHz, AM<br><br>2 conditions: sham exposure and exposure (PD 1.0 mW/cm², SAR 2.01 W/kg) (measurement, calculation/simulation, calibration)<br><br>Whole body exposure<br><br>Exposure duration: 2 h/day for 35 days (maybe except weekend, not clearly stated)<br><br>Remarks: 2 rats were exposed simultaneously	DNA damage in the brain: DNA single strand breaks (Comet assay)	Exposure caused statistically significant increase in DNA single strand breaks in brain cells of the rats.<br><br>Conclusion: No further conclusion provided.<br><br>(Effect found)	Data of additional experiments with 2.45 GHz exposure were not extracted for this review.
Paulraj and Behari 2012a<br><br>EA<br><br>Study quality: 1st tier	Wistar rats (male, 30-35 days)<br>Exposure group: n=24 (8 for each endpoint (ODC, PKC, calcium efflux), sham exposure group: n=24 (8 for each endpoint)	Pyramidal horn antenna, 9.9 GHz, square wave modulated (1 kHz)<br><br>2 conditions: sham exposure and exposure (PD 0.125 mW/cm², SAR 1.0 W/kg (whole body)) (measurement, calculation/simulation)<br><br>Whole body exposure<br><br>Exposure duration: 2 h/day, 6 days/week for 35 days<br><br>Remarks: 2 rats were exposed simultaneously	Biochemical changes in the brain (calcium ion efflux; enzyme activities of calcium-dependent protein kinase (PKC) and ornithine decarboxylase (ODC))	Exposure significantly increased the calcium ion efflux and the activity of ODC and significantly decreased PKC activity.<br><br>Conclusion: The exposure may affect the function of the brain and its development.<br><br>(Effect found)	Data of additional (*in vitro*) experiments with isolated brains not extracted.

Authors, study design	Study groups	Exposure (Assessment method)	Endpoints	Results Conclusion according to authors (Effect categorization according to authors)	Remarks
**CNS, cognitive functions, and behavior**					
Qin et al. 2024<br><br>EA<br><br>Study quality: 2nd tier	C57BL/6 mice (male, 8 weeks)<br><br>Exposure group: n=12, sham exposure group: n=12	Antenna, 4.9 GHz<br><br>2 conditions: sham exposure and exposure (PD 50 W/m²) (no data provided on exposure validation)<br><br>Whole body exposure<br><br>Exposure duration: 1 h/day for 21 days	Effects on behavior: anxiety (Open field test), spatial memory (Y-maze test), depression-like behaviors (tail suspension test)<br><br>Effects on brain: morphology and histology of hippocampus and amygdaloid nucleus, neuronal damage (levels of neuron specific enolase (NSE) and S100 calcium binding protein B (S100B) in serum), apoptosis in hippocampal cell and amygdala, pyroptosis in hippocampus and amygdala (expression of Gasdermin D and Cleaved caspase-1)<br><br>Body weight	Exposure increased immobility time in tail suspension test as well as the levels of NSE, S100B, Gasdermin D and Cleaved caspase-1. The number of neurons in amygdala was reduced after exposure (no statistics).<br><br>Conclusion: Exposure may induce depression-like behavior in mice which might be associated with structural damage and neuronal loss through cell pyroptosis in amygdala.<br><br>(Effect found)	
Qin et al. 2023<br><br>EA<br><br>Study quality: 2nd tier	C57BL/6 mice (male, 8 weeks)<br><br>Exposure group: n=12, sham exposure group: n=12	Antenna, 4.9 GHz<br><br>2 conditions: sham exposure and exposure (PD 50 ± 2.5 W/m²) (no data provided on exposure validation)<br><br>Whole body exposure<br><br>Exposure duration: 1 h/day for 6-7 days (depending on endpoint)	Effects on behavior (exposure for 6 or 7 days): locomotor activity, exploratory and anxiety-like behavior (Open field test), responsiveness to novel environment and spatial memory (Y-maze test), depression-like behaviors (tail suspension test)<br><br>Effects on brain (exposure for 7 days): brain morphology and autophagy (3 animals/group), proteomic analysis of hippocampus (3 animals/group); protein expression of S100B and serotonin in serum (10 animals/group), hippocampal (and amygdala?) cell apoptosis<br><br>Body weight (after 1, 3, 5 and 7 days of exposure)	Protein expression of S100B in serum and the level of autophagy in amygdala increased while serotonin expression was decreased in exposed mice. Moreover, numerous deep staining cells, karyopyknosis and necrosis were found in amygdala (no statistics) and the number of neurons in amygdala decreased in exposed animals.<br><br>Conclusion: Exposure could induce structural damage and neuronal loss in mice amygdala.<br><br>(Effect found)	Two additional exposure groups (electromagnetic pulse (EMP) and co-exposure to EMP + 4.9 GHz) were irrelevant for review and therefore not extracted

**Authors, study design**	**Study groups**	**Exposure (Assessment method)**	**Endpoints**	**Results Conclusion according to authors (Effect categorization according to authors)**	**Remarks**
**CNS, cognitive functions, and behavior**					
Quock et al. 1994<br><br>EA<br><br>Study quality: 2nd tier	Swiss CD1 mice (male, age not stated)<br><br>Exposure group 1: n=?, exposure group 2: n=?, exposure group 3: n=?, sham exposure group: n=?	Horn antenna, 4.7 GHz, CW<br><br>4 conditions: sham exposure and 3 exposure groups (SAR 4, 12, and 36 W/kg) (measurement)<br><br>Whole body exposure<br><br>Exposure duration: 5 min<br><br>Remarks: animals restrained	Anxiolytic and sedative effects: mouse staircase test (number of rears, number of steps ascended)	No significant effects of microwave exposure on number of rears or number of ascended stairs.<br><br>Conclusion: Microwave exposure did not alter behavior.<br><br>(No effect found)	Groups exposed to 1.8 GHz and/or co-exposure to EMF and chlordiazepoxide were irrelevant for review and therefore not extracted

**Authors, study design**	**Study groups**	**Exposure (Assessment method)**	**Endpoints**	**Results Conclusion according to authors (Effect categorization according to authors)**	**Remarks**
**CNS, cognitive functions, and behavior**					
Raslear et al. 1993<br><br>EA<br><br>Study quality: 2nd tier	Albino Sprague-Dawley rats (male, 90-110 days)<br><br>Exposure group 1: n=8 (200 pulses; not clear if the same animals were used for all exposure conditions), exposure group 2: n=8 (200 pulses (-20 dB)), exposure group 3: n=8 (200 pulses (-30 dB)), exposure group 4: n=8 (200 pulses (-40 dB)), exposure group 5: n=8 (200 pulses (-50 dB)), cage control: n=8?, sham exposure group: n=8? (not clear if the same animals were used for exposure and sham exposure/cage control)	circular waveguide, 3.0 GHz, PW (width 80 ns, rate 0.125 pps)<br><br>7 conditions: sham exposure, cage control, 5 exposure groups (0, -20, -30, -40, -50 dB) (peak power 700 MW; SAR 0.072 W/kg (whole-body time-averaged), SARs in the brain ranged from 0.057-0.087 W/kg) (measurement, calculation/simulation)<br><br>Whole body exposure<br><br>Exposure duration: 200 pulses (during approx. 25 min)<br><br>Environmental conditions: influence of co-exposure to X-rays and noise emitted by the exposure system was investigated<br><br>Exposure remarks: 2 animals exposed simultaneously; animals restrained; SAR values for 24 body locations provided; antenna output was attenuated by wire screen of varying mesh densities	Sensory processing (perceptual and cognitive effects): discrimination performance in a bisection task	Dose-response effects were observed for discriminability, session time, and trial completions.<br><br>Microwave exposures affected cognitive function in the rats, particularly the decision-making process.<br><br>(Effect found)	

Authors, study design	Study groups	Exposure (Assessment method)	Endpoints	Results Conclusion according to authors (Effect categorization according to authors)	Remarks
**CNS, cognitive functions, and behavior**					
Rui et al. 2022<br><br>EA<br><br>Study quality: 2nd tier	Sprague-Dawley rats (male, age not stated)<br><br>Exposure group 1: n=25,<br>exposure group 2: n=25,<br>sham exposure group: n=25	Horn antenna, 5.8 GHz<br><br>3 conditions: sham exposure and 2 exposure groups (PD 74.25 W/m², SAR 1.15 W/kg (whole body), SAR 2.33 W/kg (brain)): group 1 (2 h/day), group 2 (4 h/day) (measurement)<br><br>Whole body exposure<br><br>Exposure duration: 2 h or 4 h per day for 15 days<br><br>Remarks: rats were placed individually in boxes, arranged in 5 × 5 array and placed on the shelf with the lateral side facing the antenna (3.3 m away from the antenna)	Cognitive functions: learning and memory ability (12-13 rats/test) (Morris water maze, novel object recognition test), fear conditioning test<br><br>Damage of hippocampal neurons: hippocampal morphology (4 rats), level of brain injury factors in serum (neuron specific enolase, S100B; 5 rats), mitochondrial membrane potential of hippocampal neurons (4 rats)<br><br>Hippocampal synaptic plasticity: density of dendritic spines (4 rats), ultrastructure of synapses (4 rats), level of PSD95 (4 rats), synaptophysin, p-CREB and CREB (4 rats)	No statistically significant effects.<br><br>Conclusion: Exposure to 5.8 GHz microwave could not affect the hippocampal synaptic plasticity of rats.<br><br>(No effect found)	
Sahingöz Yildirim et al. 2022<br><br>EA<br><br>Study quality: 2nd tier	Balb/c mice (pregnant/*in utero*) (male and female)<br><br>Exposure group: n=8, control group: n=7	Horn antenna, 10.715 GHz<br><br>2 conditions: control and exposure group (PD 0.84 mW/cm², SAR 0.725 W/kg (average)) (calculation/simulation)<br><br>Whole body exposure<br><br>Exposure duration: 12 hours/day until birth (22 or 25 days)	Expression of genes related to neuronal migration in 29/12 (exposure/control) newborn rats' brains (DCX, TUBA1, YWHAE, ARX, RELN, LARGE, FLNA)	The expressions of 6 of the 7 genes (ARX, FLNA, DCX, LARGE, RELN and YWHAE) were increased.<br><br>Conclusion: Microwave exposure may affect the development of fetuses as a consequence of gene expression changes.<br><br>(Effect found)	

Authors, study design	Study groups	Exposure (Assessment method)	Endpoints	Results Conclusion according to authors (Effect categorization according to authors)	Remarks
**CNS, cognitive functions, and behavior**					
Sharma et al. 2014a<br><br>EA<br><br>Study quality: 2nd tier	Swiss albino mice (male, 6-8 weeks)<br><br>Exposure group: n=?, sham exposure group: n=?	Horn antenna, 10 GHz<br><br>2 conditions: sham exposure, 10 GHz exposure (PD 0.25 mW/cm², SAR 0.1790 W/kg) (measurement, calculation/simulation)<br><br>Head exposure<br><br>Exposure duration: 2 h/day for 30 days<br><br>Remarks: animals were immobilized	Learning and memory (Radial arm maze test; 6 mice/group)<br><br>Biochemical changes (levels of lipid peroxidation, reduced glutathione, superoxide dismutase, catalase and proteins; 1, 7, 15 and 30 days after end of exposure; 6 mice/group)	Protein, superoxide dismutase and glutathione levels decreased in exposed animals' brains at all follow-up intervals while catalase and lipid peroxidation levels increased. Spatial learning and memory performance was reduced in exposed mice.<br><br>Conclusion: Free radicals causing oxidative stress may be responsible for altered biochemical levels of the brain and impairment of learning and working memory after 10 GHz exposure.<br><br>(Effect found)	
Wang et al. 2022<br><br>EA<br><br>Study quality: 2nd tier	Wistar rats (male, 6-8 weeks)<br><br>Exposure group: n=30 (?), sham exposure group: n=30 (?)	Horn antenna, 4.3 GHz<br><br>2 conditions: sham exposure, 4.3 GHz exposure (average PD 10 mW/cm², SAR 2.5 W/kg) (calculation/simulation)<br><br>Whole body exposure<br><br>Exposure duration: 6 min	Spatial learning and memory (Morris water maze test; 10 rats/group)<br><br>Hippocampal ultrastructure (TEM)<br><br>Cortical electrical activity (EEG; 5 rats/group)<br><br>Serum exosome identification and exosome protein expression (4 rats/group)<br><br>Morphology analysis (TEM) and proteomic analysis	Hippocampal damage was observed, number of exosomes was decreased and 145 exosomal proteins were differentially expressed in exposed animals.<br><br>Conclusion: Microwave exposure might damage the hippocampus.<br><br>(Effect found)	Data on exposure to 1.5 GHz exposure and co-exposure to 1.5 GHz + 4.3 GHz were not relevant for the review and therefore not extracted.

Authors, study design	Study groups	Exposure (Assessment method)	Endpoints	Results Conclusion according to authors (Effect categorization according to authors)	Remarks
**CNS, cognitive functions, and behavior**					
Wang et al. 2023<br><br>EA<br><br>Study quality: 2nd tier	Wistar rats (male, 6-8 weeks)<br><br>Exposure group 1: n=17 (10 mW/cm²), exposure group 2: n=17 (30 mW/cm²), sham exposure group: n=17	Horn antenna, 9.375 GHz<br><br>3 conditions: sham exposure, exposure group 1 (PD 10 mW/cm², SAR 3.375 W/kg (whole-body)), exposure group 2 (PD 30 mW/cm², SAR 10.125 W/kg (whole-body)) (calculation/simulation)<br><br>Whole body exposure<br><br>Exposure duration: 10 min<br><br>Remarks: restrained rats were exposed with incident direction of the microwave from the dorsal side to the ventral side on a rotating platform	Spatial memory (Morris water maze; 12 rats/group)<br><br>Brain network topological organization (rs-fMRI; 5 rats/group)	No significant effects found.<br><br>Conclusion: No significant changes in the spatial memory or topological organization of the brain network were observed in rats subjected to 9.375 GHz exposure.<br><br>(No effect found)	Data on exposure to 1.5 GHz were not relevant for the review and therefore not extracted.

Authors, study design	Study groups	Exposure (Assessment method)	Endpoints	Results<br>Conclusion according to authors<br>(Effect categorization according to authors)	Remarks
**CNS, cognitive functions, and behavior**					
Zhu et al. 2021<br><br>EA<br><br>Study quality: 2nd tier	Wistar rats (male, 8 weeks)<br><br>Exposure group: n=35, sham exposure group: n=35	Horn antenna, 4.3 GHz, PW (rate 200 pps, width 500 ns)<br><br>2 conditions: sham exposure and exposure (PD 10 mW/cm², SAR 3.3 W/kg (average)) (measurement, calculation/simulation)<br><br>Whole body exposure<br><br>Exposure duration: 6 min<br><br>Remarks: rat cages rotating during exposure; 35 rats were exposed in two batches simultaneously (housed individually in separate compartments); rectal temperature of 5 rats controlled before and after exposure	Effects on the brain: brain activity (EEG), hippocampal morphology, Nissl body content (n=5 on days 1, 7, 14, and 28 after exposure), enzyme activities of lactate dehydrogenase and succinate dehydrogenase (on day 1 and 7 after exposure)<br><br>Learning and spatial memory (on days 1, 2, 3, 7, 14, and 28 after exposure) (Morris water maze)	The exposure group showed significantly prolonged averaged escape latencies in the water maze test at 1 day after exposure. At 7 days after exposure, hippocampal tissue from the exposure group showed varying degrees of damage. Additionally, a significant increase in the count of deeply stained neuron nucleus and significant increases in neuron's postsynaptic density thickness were found.<br><br>Conclusion: Microwave exposure induced cognitive impairment and hippocampal tissue damage, but the damage might be reversible at 28 days after the exposure.<br><br>(Effect found)	Data of additional experiments on 1.5 GHz exposure and co-exposures not extracted.

Table B. Extracted data of studies on immune system.

Authors, study design	Study groups	Exposure (Assessment method)	Endpoints	Results Conclusion according to authors (Effect categorization according to authors)	Remarks
**Immune system**					
Fesenko et al. 1999<br><br>EA<br><br>Study quality: 2nd tier	NMRI mice (male, 8-10 weeks)<br><br>Exposure group 1: n=45, exposure group 2: n=60, control group: n=20, cage control: n=105, sham exposure group: n=20	Horn antenna, 10 GHz, 8.15–18 GHz, swept frequency (direct and reversed sweep times were 1.0 s and 16 ms, respectively)<br><br>5 conditions: sham exposure, cage control, control group and 2 exposure groups (PD 1 µW/cm² (spatial average), PD 0.2-1.6 µW/cm² (range), SAR 2-5 mW/kg): group 1 (10 GHz) and group 2 (swept frequency 8.15–18 GHz) (measurement)<br><br>Whole body exposure<br><br>Exposure duration: 0.5 h-7 days	Cytotoxic activity: TNF secretion in peritoneal macrophages and in T cells (directly after 0.5, 2, 3, 5, 8, 10, 24, 72, 120 and 168 h of exposure, and 24 and 72 after exposure for 24 h)<br><br>Splenic T cell proliferation (directly, 24 h and 72 h after exposure for 24 h)	10 GHz exposure with a duration of at least 3 h induced an increase of TNF secretion with a maximum after 5 h of exposure in macrophages while exposure for 7 days led to an inhibition of TNF production (no statistics). Exposure to swept frequency 8.15–18 GHz for 24 hours increased the TNF production in macrophages and T cells for up to 72 hours after the termination of exposure.<br><br>Conclusion: Weak microwave exposure for 3 h–3 days induced an activation of TNF production in macrophages and T cells.<br><br>(Effect found)	

Authors, study design	Study groups	Exposure (Assessment method)	Endpoints	Results Conclusion according to authors (Effect categorization according to authors)	Remarks
**Immune system**					
Liddle et al. 1980<br><br>EA<br><br>Study quality: 1st tier	CD-1 mice (female, 28-35 days)<br><br>Exposure group 1: n=107 (?; previously immunized mice challenged with S pneumoniae, type III infection after exposure)<br>cage control: n=4, sham exposure group: n=106 (?)	Horn antenna, 9 GHz, PW (rate 970-1000, width 1.0 µs)<br><br>3 conditions: cage control, sham exposure and exposure (PD 10 mW/cm² (average), PD 10 W/cm² (peak), SAR 3.3 mW/g or 4.7 mW/g (depending on used model)<br>(measurement, calculation/simulation)<br><br>Exposure duration: 2 h/day for 5 days	Circulating antibody titers<br><br>Hematology: RBC and WBC counts, hemoglobin, hematocrit, lymphocytes, differential counts<br><br>Mortality/survival time	Antibody titers were higher in exposed mice.<br><br>Conclusion: Exposed mice showed a stimulation of the circulating antibody response.<br><br>(Effect found)	
Liddle et al. 1986<br><br>EA<br><br>Study quality: 1st tier	CD-1 mice (female, 28-35 days)<br>Exposure group 1: n=44 (previously immunized mice challenged with S pneumoniae, type III infection after exposure),<br>sham exposure group: n=44	Horn antenna, 9 GHz, PW (rate 970-1000, width 1.0 µs)<br><br>2 conditions: sham exposure and exposure (PD 1.0 mW/cm² (average), PD 1 W/cm² (peak), SAR 0.47 W/kg)<br>(measurement, calculation/simulation)<br><br>Exposure duration: 2h/day for 5 days	Circulating antibody titers<br><br>Hematology: RBC and WBC counts, hematocrit, hemoglobin, differential counts<br><br>Mortality/survival	No significant differences were detected between the groups.<br><br>Conclusion: Exposure did not change the circulating antibody response of immunized mice.<br><br>(No effect found)	

Authors, study design	Study groups	Exposure (Assessment method)	Endpoints	Results Conclusion according to authors (Effect categorization according to authors)	Remarks
**Immune system**					
Novoselova et al. 1999<br><br>EA<br><br>Study quality: 2nd tier	NMRI mice (male, 8-10 weeks)<br><br>Exposure group: n=20 (?), cage control: n=20 (?), sham exposure group: n=20 (?)	Horn antenna, 8.15–18 GHz, swept frequency (direct and reversed sweep times were 1.0 s and 16 ms, respectively)<br><br>3 conditions: sham exposure, cage control, and exposure (PD 1 µW/cm² (spatial average), PD 0.2-1.6 µW/cm² (range), SAR 2-5 mW/kg) (measurement)<br><br>Whole body exposure<br><br>Exposure duration: 5 hours	Stimulation of the immune system: TNF production in peritoneal macrophage and T cells (0, 24, 48, and 72 h after exposure)<br><br>Splenic T cell proliferation (0, 24, and 72 h after exposure)	Exposure led to an increase of TNF production in macrophages and T cells directly and 24 hours after termination of exposure.<br><br>Conclusion: Exposure to low-power microwaves stimulated the immune reaction of macrophages and T cells.<br><br>(Effect found)	Groups (co-)exposed to EMF and antioxidants feeding were not relevant for review and therefore not extracted

Authors, study design	Study groups	Exposure (Assessment method)	Endpoints	Results Conclusion according to authors (Effect categorization according to authors)	Remarks
**Immune system**					
Novoselova et al. 2004<br><br>EA<br><br>Study quality: 1st tier	NMRI mice (male, 8-10 weeks)<br><br>exposure group 1: n=65 (tumor-bearing mice)<br><br>exposure group 2: n=15 (healthy mice)<br><br>sham exposure group: n=65	Rectangular horn-antenna, 8.15-18 GHz, swept frequency (direct and reversed sweep times were 1.0 s and 16 ms, respectively)<br><br>2 conditions: sham exposure and 2 exposure groups (PD 1 µW/cm² (spatial average), PD 0.4-1.6 µW/cm² (range), SAR 2-5 mW/kg) (measurement)<br><br>Exposure duration: 1.5 h/day for up to 30 days (starting on day 1 after transplantation of carcinoma cells)<br><br>Remarks: animal cancer model was produced by subcutaneous injection of Ehrlich ascite carcinoma cells in one hind limb	TNF production in peritoneal macrophage and T cells (on days 7, 14 and 30 after transplantation of carcinoma cells)<br><br>Tumor size<br><br>Survival time	TNF production was increased in healthy and tumor-bearing mice after 7, 14 and 30 days of exposure. Exposure increased survival in tumor-bearing mice and tumor sizes were decreased from day 14 to 30 of exposure.<br><br>Conclusion: Exposure to 8.15-18 GHz increased survival time and decreased tumor growth rate mediated by TNF production.<br><br>(Effect found)	Groups co-exposed to EMF and a diet with antioxidants were not relevant for review and therefore not extracted

Authors, study design	Study groups	Exposure (Assessment method)	Endpoints	Results Conclusion according to authors (Effect categorization according to authors)	Remarks
**Immune system**					
Novoselova et al. 2017<br><br>EA<br><br>Study quality: 2nd tier	Balb/C mice (male, 3 months)<br><br>Exposure group: n=5, control group: n=5, sham exposure group: n=5	Rectangular horn antenna, 8.15–18 GHz, sweep frequency (direct and reversed sweep times were 1.0 s and 16 ms, respectively)<br><br>3 conditions: control, sham exposure and exposure (PD 0.4 µW/cm²-1.6 µW/cm², 1 µW/cm² (average)<br>(measurement)<br><br>Whole body exposure<br><br>Exposure duration: 1 h	Blood cytokine profile (levels of interleukin (IL)-1, IL-6, IL-10, IL-17, TNF- α, IFN-γ)<br><br>Spleen lymphocyte heat shock and signal cascade protein expression (HSP70, HSP90α, NF-ϰB, SAPK/JNK, IRF-3, p38 MAPK, and TLR4)	Exposure induced an increase in the plasma cytokine values of IL-6, TNF-α, and IFN-γ, the HSP90-α and HSP72 expressions as well as an activation of the NF-kB, p38 MAPK, and the TLR 4 pathways in splenic lymphocytes.<br><br>Conclusion: Exposure to 8.15-18 GHz microwaves might modulate the immune system in mice.<br><br>(Effect found)	Groups (co-)exposed to toluene were not relevant for review and therefore not extracted
Rifat et al. 2013<br><br>EA<br><br>Study quality: 2nd tier	Swiss albino mice (male, 6-8 weeks)<br><br>Exposure group: n=10<br>sham exposure group: n=10	Horn antenna, 10 GHz<br><br>2 conditions: sham exposure and exposure (PD 0.25 mW/cm², SAR 0.1790 W/kg)<br>(measurement, calculation/simulation)<br><br>Whole body exposure<br><br>Exposure duration: 2h/day for 30 consecutive days	Biochemical changes of the spleen: levels of lipid peroxidation, glutathione and total protein<br><br>Histopathological changes of spleen<br><br>Spleen (and body?) weights	Spleen weight, total protein and glutathione levels were decreased while lipid peroxidation was increased in exposed animals. Moreover, exposed mice showed congestion and changes in the architecture of spleen (e.g., trabeculae were not clearly defined) as well as fibrosis of pulp with enlarged, irregularly shaped sinusoids and giant cells (no statistics).<br><br>Conclusion: Exposure led to histopathological and biochemical alterations in spleen of mice.<br><br>(Effect found)	Groups with co-exposure to *Prunus Avium* extract were not relevant for review and therefore not extracted

Authors, study design	Study groups	Exposure (Assessment method)	Endpoints	Results Conclusion according to authors (Effect categorization according to authors)	Remarks
**Immune system**					
Shao and Chiang 1989<br><br>EA<br><br>Study quality: 1st tier	Kunming mice, infant (4-5 weeks), young (9-10 weeks), adult (over 16 weeks)<br>Mice immunized by sheep red blood cells (SRBC)<br>Young mice<br>Exposure groups 1-7: n=10 each, sham exposure groups 1-6: n=10 each<br>Infant mice<br>exposure group 8: n≥10, sham exposure group 7: n=10<br>Adult mice<br>exposure group 9: n≥10, sham exposure group 8: n=10	Horn antenna, 3 GHz, PW (rate 933 Hz, width 1.2 µs)<br><br>17 conditions: 8 sham exposure groups and 9 exposure groups: group 1 (PD 1 mW/cm²), groups 2, 8, 9 (PD 5 mW/cm²), groups 3, 5, 6, 7 (PD 7 mW/cm²), group 4 (PD 12 mW/cm²) (measurement)<br><br>Whole body exposure<br><br>Exposure duration: 60 min/day for 2 days<br><br>Remarks: animals were restrained	Immune reaction: immunization with sheep red blood cells (10 animals/group at 7, 8, 9 and 22 days after exposure; groups 1-4 and 8-9 only after 8 days, measurement of hemagglutinin titer)	An increase in immune reaction was observed 8 days after exposure at 5, 7 and 12 mW/cm². Day 8 after exposure at 7 mW/cm² was the day of maximum increase in immune reaction. Afterwards, the reaction declined and reached the level of sham exposure on day 22 after exposure. Infant mice did not show a reaction to exposure to 5 mW/cm² but young and adult mice showed an increase in immune reaction.<br><br>Conclusion: Exposure stimulated immune functions in mice for short periods. The immune system of older mice was more susceptible.<br><br>(Effect found)	

Authors, study design	Study groups	Exposure (Assessment method)	Endpoints	Results<br>Conclusion according to authors<br>(Effect categorization according to authors)	Remarks
**Immune system**					
Veyret et al. 1991<br><br>EA<br><br>Study quality: 1st tier	Balb/C mice (male, 6 weeks)<br><br>Mice immunized by sheep red blood cells (SRBC)<br>14 different exposure groups: n=12 each<br><br>Mice immunized by glutaric-anhydride conjugated bovine serum albumin (GA-BSA)<br>11 different exposure groups: n=10 each<br><br>25 sham exposure groups (same numbers as exposure groups): n=10 or 12 each	Coaxial antenna, 9.4 GHz ± 0.01 GHz, PW (width 1 µs, rate 1,000 pps, 5 ns rise time) with and without AM differentially modulated at 14-41 MHz<br><br>50 conditions: 25 sham exposure groups and 25 exposure groups (PD 30 ± 2 µW/cm² (RMS), SAR ~0.015 W/kg): PW with and without AM differentially modulated at 14-41 MHz (measurement, calculation/simulation)<br><br>Whole body exposure<br><br>Exposure duration: 10 h/day for 5 days	Antibody response: plaque-forming-cell assay (SRBC groups), titration of IgM and IgG antibodies (GA-BSA groups)	In SRBC groups, there was a decrease in the number of plaques in exposures with AM at 14, 36, or 41 MHz, and an increase at 21 or 32 MHz. In GA-BSA groups, there was an increase in the IgM titers in exposure with PW modulation alone. With the addition of AM modulation, there was a decrease in antibody titers at 14, 36, and 41 MHz, and an increase at 21 and 32 MHz.<br><br>Conclusion: Low power, PW microwaves can affect antibody responses in mice if the pulsed component of the carrier is impressed with AM.<br><br>(Effect found)	

Table C. Extracted data of studies on thermoregulation and heat-related effects.

Authors, study design	Study groups	Exposure (Assessment method)	Endpoints	Results Conclusion according to authors (Effect categorization according to authors)	Remarks
**Thermoregulation and heat-related effects**					
Blick et al. 1997<br><br>HCT<br><br>Study quality: 2nd tier	Volunteers (male, 45.2±6.0 years)<br><br>Exposure condition 1: n=15,<br>exposure condition 2: n=15,<br>control condition: n=15<br><br>same subjects used for every condition	Different horn antennas, 7.5 GHz and 10.0 GHz<br><br>>3 conditions: control condition and 2 exposure conditions (different not further described intensities): condition 1 (7.5 GHz) and condition 2 (10.0 GHz)<br>(measurement, calibration)<br><br>Exposure of the middle of the uncovered back (327 cm²)<br><br>Exposure duration: 20 single 10 s-stimuli for each frequency (at 1 min intervals)<br><br>Remarks: subjects were exposed in the E orientation (long axis of subject parallel to E-vector)	Warmth sensation in skin: detection thresholds (staircase procedure)	The detection threshold was 19.6 ± 2.9 mW/cm² at 10 GHz and 19.5 ± 2.9 mW/cm² at 7.5 GHz.<br><br>Conclusion: Sensitivity increased with frequency throughout the range of microwave frequencies (from 2.45 GHz to 94 GHz) tested.<br><br>(Effect found)	Additional data on exposure to 2.45 GHz, 35 GHz, 94 GHz and infrared irradiation not relevant for this review and therefore not extracted.<br><br>No further described catch (control) trials were included in exposure trials

Authors, study design	Study groups	Exposure (Assessment method)	Endpoints	Results Conclusion according to authors (Effect categorization according to authors)	Remarks
**Thermoregulation and heat-related effects**					
Frei et al. 1989<br><br>EA<br><br>Study quality: 1st tier	Sprague-Dawley rats (female, age not stated)<br><br>Exposure condition 1: n=6,<br>exposure condition 2: n=6,<br>exposure condition 3: n=6,<br>exposure condition 4: n=6<br><br>The same 6 animals were used for every condition.	Antenna (CW), horn antenna (PW), 9.3 GHz, CW, PW (width 2 µs, rate 500 pps)<br><br>4 conditions: condition 1 (PD 30 mW/cm², SAR 9.3 W/kg (CW)), condition 2 (PD 30 mW/cm², SAR 9.3 W/kg (PW)), condition 3 (PD 60 mW/cm², SAR 18.6 W/kg (CW)), condition 4 (PD 60 mW/cm², SAR 18.6 W/kg (PW)) (measurement, calculation/simulation)<br><br>Whole body exposure<br><br>Exposure duration: until colonic temperature increased by 1°C from 38.5°C to 39.5°C (between approx. 13-41 min for one cycle, according to Figure)<br><br>Remarks: exposure was conducted to cyclically increase colonic temperature from 38.5 to 39.5°C; 8 x 1°C cycles in each animal anesthetized rats were exposed in H-orientation (left lateral exposure, long axis of animal parallel to the magnetic field)	Effects on thermoregulation: colonic, tympanic, subcutaneous temperature<br><br>Effects on cardiovascular system: heart rate, ECG, blood pressure, respiratory rate	The time required to achieve a 1°C colonic temperature increase varied inversely with the average power density used during exposure (at 30 mW/cm² approximately three times longer than at 60 mW/cm²). At both exposure levels, heart rate increased significantly during exposure and returned to baseline when exposure was discontinued. The increase of subcutaneous and tympanic temperatures exceeded the increase of colonic temperature.<br><br>Conclusion: No differences in effects between CW and PW on the investigated parameters.<br><br>(Effect found)	

Authors, study design	Study groups	Exposure (Assessment method)	Endpoints	Results<br>Conclusion according to authors<br>(Effect categorization according to authors)	Remarks
**Thermoregulation and heat-related effects**					
Ijima et al. 2023a<br><br>EA<br><br>Study quality: 2nd tier	Sprague-Dawley rats, male<br>(male, 8-9 weeks)<br><br>Exposure groups 1-4: n=6 to 15 rats (subgroup of 55 animals),<br><br>sham exposure group: n=6 to 15 rats (subgroup of 55 animals),	Patch antenna, 26.5 GHz, CW<br><br>5 conditions: sham exposure (0 W/m²) and 4 exposure groups: group 1 (APD 125 W/m²), group 2 (APD 250 W/m²), group 3 (APD 370 W/m²), group 4 (APD 500 W/m²)<br>(measurement, calculation/simulation)<br><br>Exposure of dorsal skin<br><br>Exposure duration: 18 min<br><br>Remarks: dorsal hair of rats shorn; anaesthetized rats were restrained and exposed individually; abdomen placed on a heating pad (39.5°C)	Effects on thermoregulation (rectal and dorsal skin temperature; target site, 1 cm, and 2 cm away from target)	Dorsal skin temperature increased by approx. 11.3 °C at 500 W/m², but the rectal temperature increased by only 0.6 °C, indicating highly localized effects. A significant correlation was observed between APD and skin temperature rise. An increase in rectal temperature was observed only in the 370 W/m² and 500 W/m² exposure groups. A temperature rise of less than 5 °C (4.32°C) was estimated in the skin exposed to 200 W/m² of APD.<br><br>Conclusion: The data suggest that the operational threshold for the millimeter wave exposure guidelines is valid under the present experimental conditions using rats.<br><br>(Effect found)	

Authors, study design	Study groups	Exposure<br>(Assessment method)	Endpoints	Results<br>Conclusion according to authors<br>(Effect categorization according to authors)	Remarks
**Thermoregulation and heat-related effects**					
Ijima et al. 2023b<br><br>EA<br><br>Study quality: 1st tier	Sprague-Dawley rats (male, 8-9 weeks)<br><br>exposure group 1: n=11,<br>exposure group 2: n=12,<br>sham exposure group: n=8	Lens antenna (horn antenna and dielectric lens), 28.0 GHz, CW<br><br>3 conditions: sham exposure and 2 exposure groups: group 1 (APD 122 W/m², SAR 3.7 W/kg (whole body)), group 2 (APD 237 W/cm², SAR 7.2 W/kg (whole body)) (measurement, calculation/simulation)<br><br>Whole body exposure/exposure of shorn dorsal side<br><br>Exposure duration: 40 min<br><br>Remarks: dorsal hair of rats shorn; rats restrained and exposed individually	Effects on thermoregulation (rectal temperature, dorsal and tail skin temperature, dorsal and tail skin blood flow)	Intensity-dependent temperature increases were observed in the dorsal skin, the rectum, and the tail skin. Skin blood flow was altered in the tail (but not in the dorsum), accompanied by an increase in rectal temperature and resulting in an increase in tail skin temperature. It was estimated that 4.6 W/kg and 38 W/kg of whole-body averaged SAR could induce 1°C and 5°C of rectal temperature increase, respectively.<br><br>Conclusion: Exposure drove thermoregulation to transport and dissipate heat generated on the exposed body surface. The thermoregulatory systems can be activated due to rectal temperature changes.<br><br>(Effect found)	

Authors, study design	Study groups	Exposure (Assessment method)	Endpoints	Results<br>Conclusion according to authors<br>(Effect categorization according to authors)	Remarks
**Thermoregulation and heat-related effects**					
Jauchem et al. 1984<br><br>EA<br><br>Study quality: 2nd tier	Sprague-Dawley rats (female, age not stated)<br><br>Exposure conditions 1: n=7,<br>exposure conditions 2: n=7,<br>exposure conditions 3: n=7,<br>exposure conditions 4: n=7	Antenna, 5.6 GHz, CW, PW (width 1 or 2 µs, rate 250 or 500 pps)<br><br>4 conditions: condition 1 (PD 30 mW/cm², SAR 6 W/kg (CW)), condition 2 (PD 30 mW/cm², SAR 6 W/kg (PW)), condition 3 (PD 60 mW/cm², SAR 12 W/kg (CW)), condition 4 (PD 60 mW/cm², SAR 12 W/kg (PW))<br>(measurement, calculation/simulation)<br><br>Whole body exposure<br><br>Exposure duration: up to approx. 210 min. for 8 x 1°C-cycles (derived from a Figure); for one cycle at 60 mW/cm² approx. 6.3 ± 0.3 min and at 30 mW/cm² approx. 14.9 ± 1.2 min)<br><br>Exposure remarks: exposure was conducted to cyclically increase colonic temperature from 38.5 to 39.5°C; 8 x 1°C cycles in each animal; anesthetized rats were exposed in H-orientation	Effects on cardiovascular system: heart rate, ECG, blood pressure, respiratory rate<br><br>Effects on thermoregulation: colonic, tympanic, subcutaneous temperature	Exposure to 60 mW/cm² resulted in a significant increase in heart rate compared to the 30 mW/cm² exposure groups. The average time for colonic temperature to increase 1°C was over twice as long in animals exposed to 30 mW/cm² as in those exposed to 60 mW/cm². There was an inverse relationship between the time required for a 1°C rise in colonic temperature and the change in heart rate.<br><br>Conclusion: Heart rate changes during exposure to 5.6 GHz exposure were related to the average PD applied, and thus to the rate of change in temperature, and not simply to the absolute change in temperature.<br><br>(Effect found)	

Table D. Extracted data of studies on male fertility.

Authors, study design	Study groups	Exposure (Assessment method)	Endpoints	Results Conclusion according to authors (Effect categorization according to authors)	Remarks
**Male fertility**					
Kumar et al. 2011<br><br>EA<br><br>Study quality: 2nd tier	Wistar rats (male, 70 days)<br><br>exposure group: n=6, sham exposure group: n=6	Horn antenna, 10 GHz<br><br>2 conditions: sham exposure and exposure (PD 0.21 mW/cm², SAR 0.014 W/kg) (measurement, calculation/simulation)<br><br>Whole body exposure<br><br>Exposure duration: 2 h/day for 45 days	Male fertility: histone kinase activity, total reactive oxygen species levels, spermatozoa cell cycle analysis (in sperm samples)	Histone kinase activity decreased while the total reactive oxygen species and apoptosis increased in exposed rats. In cell cycle analysis, S and G2/M phase were significantly lower.<br><br>Conclusion: Exposure showed a significant effect on the reproductive pattern in male rats.<br><br>(Effect found)	
Kumar et al. 2012<br><br>EA<br><br>Study quality: 2nd tier	Wistar albino rats (male, 70 days)<br><br>exposure group: n=6, sham exposure group: n=6	Horn antenna, 10 GHz<br><br>2 conditions: sham exposure, exposure (PD 0.21 mW/cm², SAR 0.014 W/kg) (measurement, calculation/simulation)<br><br>Whole body exposure<br><br>Exposure duration: 2 h/day for 45 days	Biochemical changes: malondialdehyde in sperm, melatonin concentration and creatine kinase in serum<br><br>Complete blood count: hemoglobin, leukocytes, neutrophils, lymphocytes, platelets, red blood cells	Malondialdehyde and creatine kinase activity were increased in exposed animals while the melatonin concentration was decreased. Hemoglobin level as well as count of leukocytes, neutrophils, lymphocytes, platelets, and red blood cells was also decreased in exposed animals.<br><br>Conclusion: Exposure could adversely affect male fertility mediated by oxidative stress.<br><br>(Effect found)	

Authors, study design	Study groups	Exposure (Assessment method)	Endpoints	Results Conclusion according to authors (Effect categorization according to authors)	Remarks
**Male fertility**					
Kumar et al. 2013<br><br>EA<br><br>Study quality: 2nd tier	Wistar rats (male, 70 days)<br>Exposure group: n=6, sham exposure group: n=6	Horn antenna, 10 GHz<br><br>2 conditions: sham exposure and exposure (PD 0.21 mW/cm², SAR 0.014 W/kg)<br>(measurement, calculation/simulation)<br><br>Whole body exposure<br><br>Exposure duration: 2 h/day for 45 days	Genotoxicity in blood: micronucleus assay, chromosomal aberrations<br><br>Fertility parameters: DNA strand breaks in spermatozoa, caspase-3 activity, diameter of seminiferous tubules, serum testosterone level, testicular weight	The occurrence of micronuclei, DNA strand breaks and caspase-3 activity (indicating apoptosis) increased in exposed animals while the diameter of seminiferous tubules, testosterone and testicular weight level decreased.<br><br>Conclusion: Exposure affected fertility of rats through DNA damage.<br><br>(Effect found)	Experiments were repeated 3 times.
Xue et al. 2022<br><br>EA<br><br>Study quality: 2nd tier	Sprague-Dawley rats (male, adult)<br><br>Exposure group: n=18, sham exposure group: n=18	Horn antenna, 5.8 GHz<br><br>2 conditions: sham exposure and exposure (PD 74.25 W/m², SAR 1.15 W/kg (whole body), SAR 3.36 W/kg (testis)), (measurement, calculation/simulation)<br><br>Whole body exposure<br><br>Exposure duration: 1 h/day for 30 days	Sperm count and sperm abnormality (13 rats/group)<br><br>Testicular histological structure and apoptosis (caspase-3 expression; 5 rats/group)<br><br>Serum levels of sex hormones (follicle stimulating hormone, luteinizing hormone, testosterone (5 rats/group)<br><br>Testicular secretory function: levels of glial cell line-derived neurotrophic factor, stem cell factor, transferrin in testicular tissue, (5 rats/group)<br><br>General body condition, rectal temperature, relative testis weight	No significant results.<br><br>Conclusion: Exposure had no obvious effect on testicular structure and function of rats.<br><br>(No effect found)	

Table E. Extracted data of studies on fetus and teratogenicity.

**Authors, study design**	**Study groups**	**Exposure (Assessment method)**	**Endpoints**	**Results Conclusion according to authors (Effect categorization according to authors)**	**Remarks**
**Effects on fetus & teratogenicity**					
Jensh 1984a<br><br>EA<br><br>Study quality: 2nd tier	Wistar albino rats (female, pregnant)<br><br>exposure group: n=11 (10 pregnant), control group: n=10, cage control: n=5, sham exposure group: n=10 (8 pregnant)	Horn antenna, 6.0 GHz<br><br>4 conditions: cage control, exposure chamber control, sham exposure, exposure (35 mW/cm², average SAR 7.28 W/kg) (measurement)<br><br>Whole body exposure<br><br>Exposure duration: 8 h/day for 21 days during pregnancy<br><br>Remarks: power density was determined in preliminary experiments to not increase body temperature	Teratologic examinations (2 animals/group): uterine location of each fetus and placenta, fetal and placental weight, fetal viability, mean litter size, number of resorptions and resorption rate, number of abnormalities and abnormality rate<br><br>Maternal parameters: weight and weight gain, organ weight and organ weight/body weight ratios, body weight ratios of brain, liver, kidneys, gonads and muscle, peripheral blood parameters including hematocrit, hemoglobin, and white cell (differential) counts	Fetuses of exposed dams showed growth retardation compared to the control and sham exposure groups. Maternal monocyte count was lower in exposed mothers.<br><br>Conclusion: 6.0 GHz exposure was only slightly teratogenic.<br><br>(Effect found)	

Authors, study design	Study groups	Exposure (Assessment method)	Endpoints	Results<br>Conclusion according to authors<br>(Effect categorization according to authors)	Remarks
**Effects on fetus & teratogenicity**					
Pyrpaso-poulou et al. 2004<br><br>EA<br><br>Study quality: 2nd tier	Wistar rats (female, pregnant)<br><br>Exposure group 1: n=25 (newborns exposed *in utero*), exposure group 2: n=26 (newborns exposed *in utero*)<br><br>sham exposure group: n=20 (newborns; date of sham exposure not specified)	Horn antenna, 9.4 GHz, PW (GSM-like: width 20 µs, rate 50 Hz)<br><br>2 conditions: sham exposure and 2 exposure groups (PD 5 µW/cm², SAR 0.5 µW/kg (max.)): group 1 (exposed during days 1-3 post coitum), group 2 (exposed during days 4-7 post coitum) (measurement, calculation/simulation)<br><br>Whole body exposure<br><br>Exposure duration: 3 to 4 days	Effects on kidney: Expression and localization of bone morphogenetic proteins BMP-4, -7, BMPR-IA, -IB, and -II, histology, macroscopic malformations	Kidney sections of exposed rats occasionally exhibited slight hydropic swelling in the tubular cells (no statistics). BMP-4, BMPR-IA, and -II were differentially expressed after exposure on days 1–3 post coitum and BMP-4 and BMPR-IA after exposure on days 4–7 post coitum. Aberrant expression and localization of these molecules were found at the histological level.<br><br>Conclusion: Exposure interfered with gene expression during early gestation which resulted in aberrations of BMP expression in the newborn rat.<br><br>(Effect found)	

Table F. Extracted data of studies on systemic effects.

Authors, study design	Study groups	Exposure (Assessment method)	Endpoints	Results Conclusion according to authors (Effect categorization according to authors)	Remarks
**Systemic effects**					
Adang et al. 2009<br><br>EA<br><br>Study quality: 2nd tier	Wistar Hanover albino rats (male, 4 months)<br><br>exposure group 1: n=31,<br>sham exposure group: n=31	Horn antennas, 9.70 GHz, CW<br><br>2 conditions: sham exposure and exposure (PD 3.20 W/m² (mean) ± 30%) (measurement)<br><br>Whole body exposure<br><br>Exposure duration: 2 h/day, 7 days/week for up to 21 months	Effects on blood parameters: count of leucocytes, monocytes, erythrocytes, hemoglobin, lymphocytes, neutrophiles, mean corpuscular hemoglobin concentration (after 3, 8, 11, 14, and 18 months of exposure)<br><br>Mortality rate<br><br>Anatomopathological analysis on liver, lungs, kidneys, heart, thymus, bladder, spleen, brain tissue, testes, and gastrointestinal track	Mean corpuscular hemoglobin concentration was increased after 3, 8 and 11 months of exposure. Hemoglobin concentration and erythrocytes count were increased after 3 months of exposure. After 18 months of exposure, the number of neutrophils increased, and the number of lymphocytes decreased. Mortality increased in the exposure group over the whole course of the experiment (observation period up to 3 months after the end of exposure).<br><br>Conclusion: Beside some alterations in blood parameters at different points in time, the main result was a decreased survival after long-term exposure to 9.70 GHz microwaves.<br><br>(Effect found)	Exposure groups with exposures below 3 GHz not extracted; data on pathological changes should be published elsewhere

Authors, study design	Study groups	Exposure (Assessment method)	Endpoints	Results Conclusion according to authors (Effect categorization according to authors)	Remarks
**Systemic effects**					
De Seze et al. 2020<br><br>EA<br><br>Study quality: 2nd tier	Sprague Dawley rats(male, 6 weeks)<br><br>10 GHz<br>Exposure group 1: n=61 (?)<br><br>3.7 GHz<br>exposure group 2: n=12 (?; acute exposure), exposure group 3: n=12 (?; for avoidance test), exposure group 4: n=44 (?; repeated exposure)<br><br>Sham exposure group: n=144 (?)	Horn antenna, 3.7 GHz and 10 GHz, PW (width 1-2.5 ns)<br><br>5 conditions: sham exposure and 4 exposure groups: group 1 (SAR 0.34 W/kg), group 2 (SAR 4.7 W/kg), group 3 (SAR 22 W/kg), group 4 (SAR 0.83 W/kg) (measurement, calculation/simulation)<br><br>Whole body exposure<br><br>Exposure duration: exposure group 1 (10 s every 5 min for 1 hour), exposure group 2 (2 x 8 min), exposure group 3 (14 min), exposure group 4 (2 x 8 min repeated with 10 min interval on 5 days/week for 8 weeks)	Cognitive and sensorimotor functions: beam-walking, rotarod, T-maze, open-field, avoidance test<br><br>Brain inflammation: GFAP expression<br><br>Anatomopathology: histopathology of several organs<br><br>Life span	Rotarod performance was significantly enhanced after repeated exposure. Acute exposure induced an avoidance reflex. Brain inflammation was increased two days after acute exposure and seven days after 10 GHz exposure. The life span was 4 months shorter after repeated exposure and 11 animals exhibited a tumor (no statistics applied).<br><br>Conclusion: High power microwaves can increase cancer prevalence and decrease survival time in rats.<br><br>(Effect found)	
Kössler et al. 1981<br><br>EA<br><br>Study quality: 1st tier	ICR mice (female, 16 weeks)<br><br>Exposure group: n=12, sham exposure group: n=12	Horn antenna, 3.3 GHz<br><br>2 conditions: sham exposure and exposure (PD 2 mW/cm² (average)) (measurement)<br><br>Whole body exposure<br><br>Exposure duration: 6 h/day, 5 days/week for 12 weeks	Behavior: spontaneous activity (every 14 days of exposure) (wheel running test)<br><br>Effects on blood parameters: differential blood count, erythrocyte and leukocyte level, blood sugar, hemoglobin (every 2 or 4 weeks); albumin and globulin level (after 12 weeks)<br><br>Serum enzyme activities: aspartate aminotransferase, creatine phosphokinase (after 12 weeks)	Only aspartate aminotransferase enzyme activity increased in exposed animals.<br><br>Conclusion: The data did not indicate a specific microwave effect.<br><br>(No effect found)	

Table G. Extracted data of studies on other topics.

Authors, study design	Study groups	Exposure (Assessment method)	Endpoints	Results<br>Conclusion according to authors<br>(Effect categorization according to authors)	Remarks
**Other topics**					
Akdag et al. 1999a<br><br>EA<br><br>Study quality: 2nd tier	Wistar-albino rats (female, adult)<br><br>Exposure group 1: n=8, sham exposure group: n=8	Horn antenna, 9.450 GHz, CW<br><br>2 conditions: sham exposure and exposure (PD 2.65 mW/cm², SAR 1.80 W/kg)<br>(measurement)<br><br>Whole body exposure<br><br>Exposure duration: 1h/day for 21 days	Hormone levels: serum concentrations of thyroid stimulating hormone, thyroxin, triiodothyronine, cortisol, growth hormone, follicular stimulating hormone and estradiol<br><br>Rectal temperature	Growth hormone and follicular stimulating hormone decreased while estradiol levels increased in the exposure group. The temperature rise during exposure was larger in exposed animals than in the sham exposure group.<br><br>Conclusion: 9.450 GHz exposure affected hormone levels probably due to thermal stress.<br><br>(Effect found)	Data on co-exposure to microwaves and Vitamin C or E, respectively, were not relevant for review and therefore not extracted.
Bektas et al. 2023<br><br>EA<br><br>Study quality: 2nd tier	Wistar Albino rats (healthy and diabetic (streptozotocin-induced))<br>(male, 8-10 weeks)<br><br>exposure group 1: n=7 (healthy rats), exposure group 2: n=7 (diabetic rats)<br><br>sham exposure group 1: n=7 (healthy rats), sham exposure group 2: n=7 (diabetic rats)	Antenna, 3.5 GHz<br><br>4 conditions: 2 sham exposure groups and 2 exposure groups (28.1 V/m, PD 1.6 W/m², SAR 37 mW/kg (whole-body))<br>(measurement, calculation/simulation)<br><br>Exposure with head in direction of antenna<br><br>Exposure duration: 2 h/day for 30 days<br><br>Remarks: 7 rats were exposed in a carousel with the antenna placed in the center	Effect on biomechanics of bones: bone quality, flexibility, and durability; lateral cortex thickness, medulla width, medial cortex thickness<br><br>Effect on skeletal muscle tissues: oxidative stress parameters (catalase activity, levels of reduced glutathione, malondialdehyde, ischemia-modified albumin)	Exposure had adverse effects on bone biomechanics (decreased Young's modulus, increased maximum displacement) in diabetic and healthy rats. Oxidative stress parameters were also altered by exposure.<br><br>Conclusion: Exposure may have the potential to alter bone quality and structural integrity including muscle oxidative stress parameters in healthy and diabetic rats.<br><br>(Effect found)	

Authors, study design	Study groups	Exposure (Assessment method)	Endpoints	Results Conclusion according to authors (Effect categorization according to authors)	Remarks
**Other topics**					
Hirsch et al. 1977<br><br>EA<br><br>Study quality: 2nd tier	New Zealand albino rabbits<br>(sex not stated, adult)<br><br>Exposure group 1-5: n=5 each,<br>exposure group 6: n=10	Dish antenna, 3.0 GHz, CW<br><br>6 exposure conditions:<br>group 1 (PD 50 mW/cm²),<br>group 2 (100 mW/cm²),<br>group 3 (200 mW/cm²),<br>group 4 (300 mW/cm²),<br>group 5 (400 mW/cm²),<br>group 6 (500 mW/cm²)<br>(measurement, calculation/simulation)<br><br>Exposure of left eye<br><br>Exposure duration: 15 min/day for 30 days<br><br>Remarks: animals anesthetized during exposure; temperature probably > 42°C in strongest exposure groups	Corneal or lenticular abnormalities: morphological examination, histopathological findings (daily during exposure and intermittently for up to 1 year thereafter)	At and above 300 mW/cm², iritis, posterior subcapsular iridescence, and posterior cortical cataracts were found (no statistics).<br><br>Conclusion: Exposure to microwaves ≥ 300 mW/cm² damaged the iris and produce cataract. This happened earlier at higher power levels. It appeared that the cataracts were produced by increased temperature levels.<br><br>(Effect found)	

Authors, study design	Study groups	Exposure (Assessment method)	Endpoints	Results Conclusion according to authors (Effect categorization according to authors)	Remarks
**Other topics**					
Knyazeva et al. 2018<br><br>EA<br><br>Study quality: 2nd tier	Nonlinear laboratory white mice (male, age not stated)<br><br>Exposure group 1: n=6, exposure group 2: n=6, exposure group 3: n=6, exposure group 4: n=6, sham exposure group: n=6	Horn antenna, 10 GHz, PW (4000 pulses/session, width 100 ns)<br><br>5 conditions: sham exposure and 4 exposure groups (PD 1.5 × 10^3 kW/cm² (peak), PD 1.5 × 10^3 mW/cm² (average)): group 1 (pulse repetition rate 8 pps), group 2 (13 pps), group 3 (16 pps), group 4 (22 pps) (measurement)<br><br>Whole body exposure<br><br>Exposure duration: 3 to 11 minutes, depending on the repetition frequency of the pulses (once/day for a 5-day period)<br><br>Remarks: animals probably restraint	Stress: serum corticosterone level	After exposure (13 and 16 pps) the corticosterone level increased compared to the sham control group. Exposure of mice with a frequency of 22 pps resulted in a significant decrease.<br><br>Conclusion: Repeated exposure altered the level of corticosterone in the blood serum depending on the frequency of repetition of microwave pulses.<br><br>(Effect found)	
Kumar et al. 2010<br><br>EA<br><br>Study quality: 2nd tier	Wistar rats (male, 70 days)<br><br>Exposure group: n=6, sham exposure group: n=6	Horn antenna, 10 GHz<br><br>2 conditions: sham exposure and exposure (PD 0.214 mW/cm², SAR 0.014 W/kg) (measurement, calculation/simulation)<br><br>Whole body exposure<br><br>Exposure duration: 2 h/day for 45 days	Oxidative stress in serum (total reactive oxygen species (ROS), enzyme activity of glutathione peroxidase, superoxide dismutase and catalase)<br><br>Genotoxicity in red blood cells (micronucleus assay)	The level of total ROS and micronuclei formation was increased in the exposure group. Enzyme activities of glutathione peroxidase and superoxide dismutase were decreased while catalase activity was increased.<br><br>Conclusion: Microwave radiation may be genotoxic by inducing oxidative stress.<br><br>(Effect found)	Data on exposure to 50 GHz EMF not relevant for review and therefore not extracted.

Authors, study design	Study groups	Exposure<br>(Assessment method)	Endpoints	Results<br>Conclusion according to authors<br>(Effect categorization according to authors)	Remarks
**Other topics**					
Schrot and Hawkins 1974<br><br>EA<br><br>Study quality: 1st tier	Wistar rats<br>(male, age not stated)<br><br>exposure group 1: n=7 to 13,<br>exposure group 2: n=9 to 11,<br>exposure group 3: n=10 to 13,<br>exposure group 4: n=7 to 14	Ellipsoidal antenna, 3.0 GHz, CW<br><br>16 conditions: combinations of different power densities (0.35-2.6 W/cm²) x durations (30-240 s), (measurement, calibration)<br><br>Head exposure<br><br>Exposure duration: 30, 60, 120 and 240 s<br><br>Remarks: animals were exposed individually with head facing the antenna and aligned with the longitudinal axis of the antenna	Mortality: determination of LD50 (lethal dose 50%)	At each exposure duration, the percentage of mortality increased with increasing power density. Increasing duration had the opposite effect on lethal power density, which decreased by 85% from 30 to 240 s duration. In addition, body weight was found to be a factor in determining mortality, with the heavier animals being more vulnerable to the effects of exposure. The authors found lethal power density to vary between 2.36 W/cm² and 0.42 W/cm² for exposure durations between 30 s and 240 s.<br><br>No further conclusion available.<br><br>(Effect found)	Probably, the animals were killed by heat. We have nevertheless included the article because LD50 is an internationally accepted parameter in toxicology. Most data are presented in W-min/cm² in the publication and were calculated as W/cm² by the review authors for better comparison.

Authors, study design	Study groups	Exposure (Assessment method)	Endpoints	Results<br>Conclusion according to authors<br>(Effect categorization according to authors)	Remarks
**Other topics**					
Sisodia et al. 2013<br><br>EA<br><br>Study quality: 2nd tier	Swiss albino mice (male, 6-8 weeks)<br><br>Exposure group 1: n=10,<br>sham exposure group: n=10	Pyramidal horn antenna, 10 GHz<br><br>2 conditions: sham exposure and exposure (PD 0.25 mW/cm², SAR 0.1790 W/kg) (measurement, calculation/simulation)<br><br>Exposure duration: 2 hours/day for 30 days	Hematological parameters: count of total hemoglobin, red blood cells, total and differential white blood cells, mean corpuscular hemoglobin concentration, mean corpuscular volume, erythrocyte sedimentation rate, packed cell volume and platelets<br><br>Biochemical blood parameters: levels of glucose, total protein, total cholesterol, and reduced glutathione; enzyme activities of alkaline phosphatase, acid phosphatase and lipid peroxidase<br><br>Blood smear histopathology	Hemoglobin, monocytes, packed cell volume, red blood cells, mean corpuscular hemoglobin concentration, blood glucose, acid phosphatase activity, and reduced glutathione decreased, whereas lymphocytes, erythrocyte sedimentation rate, mean corpuscular volume, cholesterol, alkaline phosphatase activity, and lipid peroxidation activity increased in the exposure group. Histopathological alterations in blood cells were also observed.<br><br>Conclusion: Several hematological, biochemical, and histopathological parameters were sensitive to exposure to 10 GHz microwaves.<br><br>(Effect found)	Data on co-exposure to *Prunus avium* extract and microwaves were not relevant for review and therefore not extracted.

Authors, study design	Study groups	Exposure (Assessment method)	Endpoints	Results Conclusion according to authors (Effect categorization according to authors)	Remarks
**Other topics**					
Verma et al. 2021<br><br>EA<br><br>Study quality: 2nd tier	Sprague-Dawley rats (hair on back was removed) (male, age not stated)<br><br>Exposure group 1: n=10,<br>exposure group 2: n=10,<br>sham exposure group: n=10	Horn antenna, 10.0 GHz, CW<br><br>3 conditions: sham exposure and 2 exposure groups: group 1 (PD 5 mW/cm² (5.23 ± 0.25 mW/cm²), SAR 0.5 ± 0.02 W/kg (whole body)), group 2 (PD 10 mW/cm² (10.01 ± 0.15 mW/cm²), SAR 1.0±0.03 W/kg (whole body)) (measurement, calculation/simulation)<br><br>Whole body exposure<br><br>Exposure duration: 3 h/day for 30 days<br><br>Remarks: rats were exposed in individual cages (n=5) placed together vertically below the antenna	Dorsal skin temperature<br><br><u>5 rats/group</u><br>Oxidative stress in skin (total ROS, lipid peroxidation (LPO), advanced oxidized protein product (AOPP), superoxide dismutase (SOD), catalase levels<br><br>Effects on energy metabolism in skin (enzyme activities of hexokinase (HK), lactate dehydrogenase (LDH), citrate synthase (CS), glucose-6-phospahte dehydrogenase (G6PD), glucose transporter (GLUT1))<br><br><u>5 rats/group</u><br>Effects on (1) inflammation, (2) chaperones and (3) cell survival signaling mediators in the skin: (1) tumor necrosis factor-alpha (TNF-alpha), interleukin-1beta (IL-1beta), NF-kappaB, nitric oxide synthase 2 (NOS2/iNOS), cyclooxygenase-2 (COX-2), 4-hydroxynonenal (4-HNE) adduct; (2) heat shock proteins (HSP70, HSP90, HSP27, GRP78, HSF-1); (3) caspase-3, p-Akt, T-Akt, HIF-1alpha, Na+-K+ ATPase, p-p38, p-p53, BCL2<br><br>Histopathology, collagen and extracellular matrix (hydroxyproline and hexosamine content)	Skin surface temperature was increased by 1.8 ± 0.38°C after exposure to 10 mW/cm² compared to sham exposed animals. Exposure to 10 mW/cm² led to oxidative stress (increased levels of ROS, 4-HNE, LPO, AOPP, SOD and catalase), inflammatory responses (increased levels of NF-kappaB, iNOS/NOS2, COX-2) and metabolic alterations (increased levels of HK, LDH, G6PD, decrease of CS) compared to controls. Additionally, an alteration (up- and downregulation) in expression of markers associated with cell survival (Akt/PKB) and HSP27/p38MAPK-related stress-response signaling cascade was observed. Qualitative histopathological analysis revealed a mild cytoarchitectural alteration in epidermal layer and slight aggregation of leukocytes in 10 mW/cm²-exposed rat skin.<br><br>Conclusion: Exposure led to significant alterations in molecular markers associated with adaptive stress-response in rat skin.<br><br>(Effect found)	
