# Supplementary material for: Physiological and health-related effects of 5G-relevant radiofrequency fields: A systematic review of experimental studies in humans and mammals
Source: PLoS One. 2026 Nov 4;21(11):e0361104. doi: 10.1371/journal.pone.0361104 (PMC13734471; doi:10.1371/journal.pone.0361104)
Supplement: Supplementary file 16 [file pone.0361104.s016.pdf]

S2 Table. Extracted study data (3rd tier studies).

A list of references for all 3rd tier studies can be found in S16 Reference List.

Abbreviations: AM = Amplitude-modulated, CW = continuous wave, EA = experimental animal study, ECG = electrocardiography, EEG = electroencephalography, fMRi = functional magnetic resonance imaging, HCT = human controlled trial, LD50 = median lethal dose, MRI = magnetic resonance imaging, PD = power density, pps = pulses per second, PW = pulsed wave, REAC = radio electric asymmetric conveyer, RT-PCR = real-time reverse transcription polymerase chain reaction, SAR = specific absorption rate, TEM = transmission electron microscopy

Authors, study design	Study groups	Exposure (Assessment method)	Endpoints	Results Conclusion according to authors (Effect categorization according to authors)	Remarks
**CNS, cognitive functions, and behavior**					
Baranski 1972a<br><br>EA<br><br>Study quality: 3rd tier	Guinea pigs and rabbits<br><br>Exposure groups 1-6: subgroups of 180 guinea pigs<br><br>exposure group 7 and 8: subgroups of 30 rabbits<br><br>control group: 30 guinea pigs and 20 rabbits	no details provided on exposure source and set-up, 3.0 GHz, CW and PW<br><br>9 conditions: control and 8 exposure groups: group 1 (3.5 mW/cm², PW, chronic exposure), group 2 (3.5 mW/cm², CW, chronic exposure), group 3 (25 mW/cm², CW, single exposure), group 4 (3.5 mW/cm², CW, single exposure), group 5 (25 mW/cm², PW, single exposure), group 6 (3.5 mW/cm², PW, single exposure), group 7 (5 mW/cm², PW, chronic exposure), group 8 (5 mW/cm², CW, chronic exposure)<br>(no data provided on exposure validation)<br><br>Exposure duration: 3 h (single exposure) and 3 h per day for 3 months (chronic exposure)	Histological effects on the brain (e.g., lesions, metachromasia, spherical metachromatic bodies)<br><br>Enzyme activities in the brain (succinic acid dehydrogenase, cytochrome oxidase, acetylcholinesterase)	Chronic exposure: Appearance of morphologic lesions was indicative of metabolic disturbances in myelin sheaths. A proliferative reaction of glial cells was also encountered (no statistics, only qualitative data provided).<br>According to the text, acetylcholinesterase activity and succinic acid dehydrogenase activity decreased (but contradictory data in the appropriate tables).<br>Single exposure: After exposure to 25 mW/cm², typical thermal lesions were present (although the authors declare that the exposure conditions were athermal).<br>Acetylcholinesterase activity was decreased after PW exposure. Succinic acid dehydrogenase activity was decreased after PW exposure and increased after CW (25 mW/cm²) exposure.<br><br>Conclusion: Chronic exposure may lead to morphologic lesions in the brain and decreased acetylcholinesterase and succinic acid dehydrogenase activity, more profound for PW than for CW.<br><br>(Effect found)	

Authors, study design	Study groups	Exposure (Assessment method)	Endpoints	Results<br>Conclusion according to authors<br>(Effect categorization according to authors)	Remarks
**CNS, cognitive functions, and behavior**					
Baranski and Edelwejn 1967<br><br>EA<br><br>Study quality: 3rd tier	Rabbits (with screw electrodes implanted into the skull)<br><br>Exposure group 1: n=10 (experiment 1)<br><br>exposure group 2: n=10 (experiment 1)<br><br>exposure group 3: n=10 (experiment 1)<br><br>exposure group 4: subgroups of 40 (exact number unknown; experiment 2)<br><br>exposure group 5: subgroups of 40 (exact number unknown; experiment 2)<br><br>control group: controls are mentioned, but no details provided	High frequency generator (CW exposure) and generator (PW) (for 3 GHz); appropriate auxillary unit, parabolic and horn antenna (for 10 GHz)<br>3 GHz and 10 GHz, CW, PW<br><br>6 conditions: control and 5 exposure groups: group 1: 3 GHz PW, group 2: 3 GHz CW, group 3: 10 GHz PW, groups 1-3: 5 mW/cm² (or 7 mW/cm², contradictory data)),<br><br>group 4: 3 GHz (5-30 mW/cm²; increasing every 15 min. by 5 mW/cm²; PW and CW),<br><br>group 5: 10 GHz (5 and 10 mW/cm²; increasing every 15 min. by 5 mW/cm²; PW),<br>(no data provided on exposure validation)<br><br>Exposure duration: experiment 1: 3 h/day for 60 days; experiment 2: 1 single exposure (2-6 x 15 min., i.e., 30-90 min.)	Bioelectric activity from different brain areas (experiment 1: before, during (after 30 days (or after 28 or 49 days,- contradictory statements) and after ending the exposure; experiment 2: every 15 min. with increasing power density and 24 h after the end of exposure (EEG)<br><br>Morphological examination of the brain (at different time points)<br><br>Rectal temperature; temperature on the surface of the cerebral cortex and in the subcutaneous tissue of the head	Chronic exposure with 3 GHz microwaves (7 mW/cm² power density) produced both functional and morphological changes, their intensity being greater with PW than with CW (only descriptive qualitative data of single rabbits).<br><br>Conclusion: Chronic 3 GHz exposure did not raise the temperature, but produced both functional and morphological changes, their intensity being greater when pulse and not continuous modulation was applied (experiment 1).<br><br>(Effect found)	

Authors, study design	Study groups	Exposure (Assessment method)	Endpoints	Results Conclusion according to authors (Effect categorization according to authors)	Remarks
**CNS, cognitive functions, and behavior**					
Bertharion et al. 1971<br><br>EA<br><br>Study quality: 3rd tier	White rats<br><br>Exposure group: n=15, control group: n=15	No data provided on exposure source, 3.0 GHz, PW (width and rate not provided)<br><br>2 conditions: control and exposure (PD 5±1 mW/cm²)<br>(no data provided on exposure validation)<br><br>Whole body exposure<br><br>Exposure duration: 1, 2, 3, 4, 5, 7 days, 1 and 5 months	Brain activity (electro-cortico-graphic recording collected by different electrode derivations after drilling the skull)	Cortico-graphic recordings were modified under exposure from day 4 of exposure and the recordings returned to normal after exposure cessation (no statistics provided).<br><br>Conclusion: Pulsed microwave exposure may have specific effects on the central nervous system.<br><br>(Effect found)	Correspondence article with preliminary results
De Lorge 1984<br><br>EA<br><br>Study quality: 3rd tier	Rhesus monkeys<br><br>Exposure group (different conditions): n=5,<br>sham exposure group: n=5<br><br>The same 5 animals were used in every group.	Horn antenna, 5.8 GHz, PW (rate 662 pps, width 0.5 or 2 µs)<br><br>Several conditions: sham exposure and exposure to PD 11 – 150 mW/cm² (exact number of conditions not provided)<br>(measurement, calculation/simulation)<br><br>Whole body exposure<br><br>Exposure duration: 60 min/session; typically, 3 sessions at each power density and each monkey<br><br>Remarks: animals restrained in a chair	Operant behavior: observing-response task/vigilance task (response rate)<br><br>Colonic temperature changes	The threshold PD of disrupted response rate was 140 mW/cm². A PD of 150 mW/cm² also produced minor burns on the faces of 3 of the 5 monkeys. These PD was associated with reliable increases in colonic temperatures above sham-exposure levels of approximately 1 °C (no statistics for all above results).<br><br>Conclusion: Exposure to 5.8 GHz microwaves of 140 mW/cm² or more changed the behavior of rhesus monkeys. Colonic temperature was the best predictor of behavioral disruption.<br><br>(Effect found)	conditions exposed to 255 MHz and 1.3 GHz were outside the reviews frequency range and therefore not extracted

Authors, study design	Study groups	Exposure (Assessment method)	Endpoints	Results Conclusion according to authors (Effect categorization according to authors)	Remarks
**CNS, cognitive functions, and behavior**					
Liu et al. 2022<br><br>EA<br><br>Study quality: 3rd tier	Sprague-Dawley rats<br><br>Exposure group 1: n=20,<br>control group: n=20	Horn antenna, 9.37 GHz, PW (width 0.6 µs, rate 1.875 kHz)<br><br>2 conditions: sham exposure and exposure (PD 30 mW/cm², SAR 6.08 W/kg)<br>(measurement, calculation/simulation)<br><br>Whole body exposure<br><br>Exposure duration: 2 min<br><br>Remarks: rats were fixed in a glass box, radiation table was rotated to assure whole body exposure	Learning and memory (Morris water maze test, 9 rats/group)<br><br>Cell proliferation and cell differentiation of hippocampal stem cells, brain histology (5-bromo-20-deoxyuridine assay)	Exposure caused learning and memory deficits in rats.<br>Different morphological changes were observed (significance not provided) and the numbers of TUNEL-positive cells were significantly increased in both the cortex and hippocampus of exposed rats.<br>Cell proliferation in cultured hippocampal cells of exposed animals was increased.<br><br>Conclusion: The learning and memory deficits were due to structural changes observed in the hippocampus after microwave exposure.<br><br>(Effect found)	The aim of the study was to investigate the effects of 20-Hydroxyecdysone in animals exposed to microwaves. Only results of microwave exposure were extracted.

Authors, study design	Study groups	Exposure (Assessment method)	Endpoints	Results Conclusion according to authors (Effect categorization according to authors)	Remarks
**CNS, cognitive functions, and behavior**					
Olazaran et al. 2013<br><br>HCT<br><br>Study quality: 3rd tier	Patients with probable Alzheimer's disease (male, female, 84.7 years)<br><br>Exposure group: n=17, sham exposure group: n=14	REAC-Emitter, 10.5 GHz, PW (single pulse)<br><br>2 conditions: sham exposure and exposure (SAR 7 µW/kg) (no data provided on exposure validation)<br><br>Exposure duration: a single 250 ms burst<br><br>Remarks: the REAC pulse was applied by touching the tip of the metallic REAC probe to a specific area of the ear	Motor measures: Axial Movement Test, Rating Scale for Gait Evaluation in Cognitive Deterioration, Steps × Seconds Test<br><br>Cognitive measure: Severe Mini-Mental State Examination<br><br>Activities of daily living performance: Barthel Index<br><br>Behavior disturbance measure: Neuropsychiatric Inventory<br><br>Safety measures: record of falls, infections, emergency room visits, hospitalizations, and any other potentially relevant events	Directly after exposure, axial movement was transitorily disturbed in exposed patients. However, after 1-3 weeks exposed patients showed an improvement in their capacity to walk (unadjusted post-hoc analysis).<br><br>Conclusion: The results hold some promise for a role of REAC in the treatment of motor deterioration in Alzheimer's disease.<br><br>(Effect found)	
Paulraj and Behari 2012b<br><br>EA<br><br>Study quality: 3rd tier	Wistar rats<br><br>Exposure group: n=6 in each category (not really clear what is meant by category), sham exposure group: n=6 (exact number not provided)	Horn antenna, 16.5 GHz, AM<br><br>2 conditions: sham exposure and exposure (PD 1.0 mW/cm², SAR 2.01 W/kg) (measurement, calculation/simulation, calibration)<br><br>Whole body exposure<br><br>Exposure duration: 2 h/day, 6 days/week for 35 days<br><br>Remarks: 2 rats were exposed simultaneously	Enzyme activity of calcium dependent protein kinase C in the brain<br><br>Brain morphological changes (liquid scintillation counting; electron microscopy; MRI)	Exposure caused significant decreases in the enzyme activity of protein kinase C (in whole brain and hippocampus) and an increase in glial cell population.<br><br>Conclusion: Chronic exposure to a low-level microwave radiation affected developing brain of rats.<br><br>(Effect found)	

Authors, study design	Study groups	Exposure<br>(Assessment method)	Endpoints	Results<br>Conclusion according to authors<br>(Effect categorization according to authors)	Remarks
**CNS, cognitive functions, and behavior**					
Pu et al. 1997<br><br>EA<br><br>Study quality: 3rd tier	LACA mice (implanted with electrodes)<br><br>Exposure group: n=12 (electrodes implanted on day 6 of exposure)<br><br>exposure group 2: n=5 or 12 (contradictory data; electrodes implanted before exposure)<br><br>exposure group 3: n=5 or 12 (contradictory data; electrodes implanted after exposure)<br><br>sham exposure group: n=12	Antenna, 3.0 GHz<br><br>2 conditions: sham exposure and exposure group (PD 5 mW/cm^2) (no data provided on exposure validation)<br><br>Whole body exposure<br><br>Exposure duration: 1 h/day for 7 days<br><br>Remarks: body (long axis) parallel to the field	Brain activity (customized EEG with implanted electrodes)<br><br>Effects on mitochondrial marker enzymes in the brain (succinate dehydrogenase and ATP level)	Exposure induced a significant decrease in brain activity. ATP and succinate dehydrogenase levels in the exposure group were significantly decreased.<br><br>Conclusion: The data suggested that microwave exposure may have an effect on brain activity.<br><br>(Effect found)	Exposure groups 2 and 3 were additional experiments to compare electrode implantation time

Authors, study design	Study groups	Exposure (Assessment method)	Endpoints	Results Conclusion according to authors (Effect categorization according to authors)	Remarks
**CNS, cognitive functions, and behavior**					
Rinaldi et al. 2014<br><br>HCT<br><br>Study quality: 3rd tier	Healthy volunteers (or patients? (contradictory data) (male, female, 19-54 years (mean: 32.4 years))<br><br>Exposure group: n=20, sham exposure group: n=13	Radio-electric asymmetric conveyer (REAC) (medical device), 5.8 GHz, single pulse<br><br>2 conditions: sham exposure and exposure (SAR 7 µW/kg, 7 µA/cm²) (150 cm from the emitter)<br>(no data provided on exposure validation)<br><br>Whole body exposure<br><br>Exposure duration: 250 ms	Brain activity (while performing a motor task) (fMRI)<br><br>Changes in functional dysmetria	Exposure group showed cerebellar and ponto-mesencephalic activation components that disappeared in the second fMRI scan, while these activation components persisted in the sham exposure group (level of statistical significance not shown).<br><br>Conclusion: Exposure could lead to lasting effects on brain activity modification.<br><br>(Effect found)	

Authors, study design	Study groups	Exposure (Assessment method)	Endpoints	Results<br>Conclusion according to authors<br>(Effect categorization according to authors)	Remarks
**CNS, cognitive functions, and behavior**					
Roberti et al. 1975<br><br>EA<br><br>Study quality: 3rd tier	Albino Wistar rats<br><br>Exposure group 1: n=15,<br>exposure group 2: n=12,<br>exposure group 3: n=6,<br>exposure group 4: n=5<br><br>4 sham exposure groups: 5 to 15 animals (equal numbers as in respective exposure groups)	Horn antenna, 3.0 GHz and 10.7 GHz, CW, PW (duty cycle 1:1000, width 1.3 µs, rate 1.3 ms)<br><br>8 conditions: 4 sham exposure groups and 4 exposure groups: group 1 (10.7 GHz, CW, PD 0.6-0.9 mW/cm²), group 2 (3.0 GHz, CW, PD 0.5-1.0 mW/cm²), group 3 (3.0 GHz, PW, PD 1.5-2.0 mW/cm²), group 4 (3.0 GHz, PW, PD 24-26 mW/cm²) (measurement)<br><br>Whole body exposure<br><br>Exposure duration: continuously for 185 h or 408 h (only group 4) for 17 days<br><br>Exposure remarks: 5-6 rats/cage, rats were kept in darkness during exposure	Effects on behavior: motor activity, runway performance (only in group 4) (registration of movement in a static electric field and running on an electrified grid floor)	No effects were found.<br><br>Conclusion: No deleterious effects of the microwave exposure have been detected.<br><br>(No effect found)	Preliminary study

Authors, study design	Study groups	Exposure (Assessment method)	Endpoints	Results Conclusion according to authors (Effect categorization according to authors)	Remarks
**CNS, cognitive functions, and behavior**					
Servantie et al. 1975<br><br>EA<br><br>Study quality: 3rd tier	Albino rats<br><br>Exposure group 1: n=4, exposure group 2: n=4, exposure,<br>sham exposure group: n=10	Generator, 3.0 GHz, PW (rate 525 and 570 pps, width 1 µs)<br><br>3 conditions: sham exposure and 2 exposure groups: group 1 (PD 5 mW/cm², 525 pps), group 2 (PD 5 mW/cm², 570 pps)<br>(no data provided on exposure validation)<br><br>Whole body exposure<br><br>Exposure duration: 10 days	Brain activity (effects on electrocorticogram)	Exposure yielded a frequency of the occipital electrocorticogram synchronous to the pulse repetition rate and this frequency persisted for few hours after exposure termination (no statistics).<br><br>Conclusion: Exposure to pulsed microwave may lead to a synchronization of cortical neurons in rats.<br><br>(Effect found)	

Authors, study design	Study groups	Exposure (Assessment method)	Endpoints	Results Conclusion according to authors (Effect categorization according to authors)	Remarks
**CNS, cognitive functions, and behavior**					
Sharma et al. 2014b<br><br>EA<br><br>Study quality: 3rd tier	Swiss albino mice<br><br>Exposure group 1: n=12,<br>sham exposure group: n=12	Horn antenna, 10.0 GHz<br><br>2 conditions: sham exposure and exposure (PD 0.25 mW/cm² (max.), SAR 0.179 W/kg) (measurement, calculation/simulation)<br><br>Head exposure<br><br>Exposure duration: 2 h/day for 30 days<br><br>Remarks: 2 mice exposed simultaneously (restrained) with their heads facing the antenna; antenna was kept in H (magnetic field) plane configuration (electric field perpendicular to the ground); field was almost uniform	Learning and spatial memory (6 mice/groups) (Morris water maze test)<br><br>Brain protein concentration (6 mice/group 48 h following exposure and 6 mice/group after water maze test)	Microwave exposure affected spatial learning and memory and induced a decrease in protein content in whole brain of mice after 48 h of exposure termination and after completion of the water maze test.<br><br>Conclusion: Microwave exposure caused decrements in the ability of mice to learn the memory task; this may be due to simultaneous decrease in protein levels in the brain of mice.<br><br>(Effect found)	

Authors, study design	Study groups	Exposure (Assessment method)	Endpoints	Results Conclusion according to authors (Effect categorization according to authors)	Remarks
**CNS, cognitive functions, and behavior**					
Sharma et al. 2017b<br><br>EA<br><br>Study quality: 3rd tier	Swiss albino mice<br><br>Exposure group 1: n=30,<br>sham exposure group: n=30	Horn antenna, 10.0 GHz<br><br>2 conditions: sham exposure and exposure (PD 0.25 mW/cm² (max.), SAR 0.1790 W/kg)) (measurement, calculation/simulation)<br><br>Head exposure<br><br>Exposure duration: 2 h/day for 15 days<br><br>Remarks: animals were lightly restrained with their heads facing the antenna; antenna was kept in H (magnetic field) plane configuration, where electric field was perpendicular to the ground surface; field was almost uniform	Effects on developing brain (brain weight; biochemical parameters (lipid peroxidation, glutathione, superoxide dismutase, catalase, protein level); histopathology (6 mice/group immediately and 6 mice/group 2 weeks after exposure)<br><br>Spatial memory and learning (at 2 weeks after exposure, 6 mice/group) (Morris water maze)<br><br>Body weight (6 mice/group immediately and 6 mice/group 2 weeks after exposure)	A reduction in body weight (except for 6 weeks of age) and brain weight was observed in mice for both immediately and 2 weeks after exposure. Levels of lipid peroxidation (increase), glutathione (decrease), protein level (decrease), catalase (increase), and superoxide dismutase (decrease) were also significantly altered immediately and 2 weeks after exposure. Exposure significantly affected spatial memory. A significant alteration in histopathological parameters was observed in CA1 region of the hippocampus, cerebral cortex, and ansiform lobule of cerebellum.<br><br>Conclusion: The brain of 2 weeks aged mice was very sensitive to microwave exposure in terms of morphometric, biochemical, behavioral, and histopathological parameters observed immediately after exposure and during follow-up study at 6 weeks of age (i.e., 2 weeks after exposure termination).<br><br>(Effect found)	Experiments were repeated thrice (but no further details provided)

Authors, study design	Study groups	Exposure (Assessment method)	Endpoints	Results Conclusion according to authors (Effect categorization according to authors)	Remarks
**CNS, cognitive functions, and behavior**					
Shtemberg et al. 2001<br><br>EA<br><br>Study quality: 3rd tier	Wistar rats<br><br>Exposure group: 3 subgroups of 10-18 rats (3 groups of rats with different higher nervous activity characteristics)<br><br>sham exposure group: 3 subgroups of 10-18 rats (3 groups of rats with different higher nervous activity characteristics)<br><br>(exact number not provided for exposure/sham exposure group)	Horn antenna, 4.2 GHz, modulated with quasichaotic signals at 20–20000 Hz<br><br>2 conditions: sham exposure and exposure (PD 15 µW/cm², 4.5 mJ/kg or 15 mJ/kg (contradictory data))<br>(no data provided on exposure validation)<br><br>Whole body exposure<br><br>Exposure duration: 1 h<br><br>Remarks: animals were unrestrained	Neurochemical effects on the brain (level of monoamines in the motor area of the cortex (noradrenaline, dopamine, adrenaline, serotonin, 5-hydroxyindoleacetic acid)<br><br>Effects on behavior: conditioned reflex activity (e.g., number of conditioned-reflex (avoidances) and non-conditioned-reflex (escapes) reactions, numbers of complete failures to perform the reflex, escape reaction time)	Exposure stimulated the acquisition of a motor-defensive conditioned reflex in rats characterized by a high level of excitability, slightly delayed acquisition in rats with a markedly inhibitory type of behavior, and selectively suppressed motor restlessness in rats characterized by unstable behavior. Rats characterized by a high level of excitability showed a significant increase in dopamine and serotonin content after exposure. Rats of the group with unstable behavior produced a decrease in the 5-hydroxyindoleacetic acid content following exposure.<br><br>Conclusion: Low-intensity exposure to microwaves produced changes in the state of the monoaminergic mediator systems. The nature of these processes depended to a significant extent on the typological characteristics of the animals.<br><br>(Effect found)	The 3 groups of rats comprised 1. rats characterized by a high level of excitability, 2. rats with a markedly inhibitory type of behavior and 3. rats characterized by unstable behavior.

Authors, study design	Study groups	Exposure<br>(Assessment method)	Endpoints	Results<br>Conclusion according to authors<br>(Effect categorization according to authors)	Remarks
**CNS, cognitive functions, and behavior**					
Teng et al. 1996<br><br>EA, cross-over study<br><br>Study quality: 3rd tier	New Zealand albino rabbits<br><br>Exposure group 1: n=1 or 2? (exact number of rabbits not provided)<br><br>exposure group 2: n=1 or 2? (exact number of rabbits not provided)<br><br>exposure group 3: n=2? (exact number of rabbits not provided)<br><br>exposure group 4: n=2? (exact number of rabbits not provided)	Implanted micro-antenna, 4.2 GHz, PW (width 200 ms or 3 s, rate 6 s), CW<br><br>5 conditions: no exposure and 4 exposure groups; group 1: PW (pulse width 200 ms, repetition period 6 s for 3 min), group 2: PW (pulse width 3 s, repetition period 6 s for 15 min), group 3: CW for 30 s, group 4: CW for 16 min (calculation/simulation, calibration)<br><br>Spinal cord exposure (the asymmetrical dipole was implanted into in the extradural cavity along the spinal cord of the animal)<br><br>Exposure duration: 30 s - 16 min	Brain activity: somatosensory evoked potentials (electrical stimulus applied at the peripheral nervous system (back paw); electrode in the cortex)	After long period of exposure (all groups except exposure group 1) the amplitude and latency of somatosensory evoked potentials were changed, and this effect was reversible. Computations showed a temperature increase for at least 3°C of the spinal cord for exposure groups 2-4.<br><br>Conclusion: Exposure decreased the latency and amplitude of the somatosensory evoked potentials. The effect was of thermal origin.<br><br>(Effect found)	cross-over design, i.e., responses under exposure were compared to responses without exposure

Authors, study design	Study groups	Exposure (Assessment method)	Endpoints	Results Conclusion according to authors (Effect categorization according to authors)	Remarks
**CNS, cognitive functions, and behavior**					
Thomas et al. 1975<br><br>EA<br><br>Study quality: 3rd tier	NMRI:O[SD] albino rats (Sprague-Dawley derived)<br><br>Exposure group 1: n=4, exposure group 2: n=4, exposure group 3: n=4, exposure group 4: n=4, sham exposure group: n=4<br><br>The same 4 animals were used in every group.	Horn antenna, 9.60 GHz, PW (width 1 µs, frequency 500 Hz)<br><br>5 conditions: sham exposure and 4 exposure conditions: PD 1 (?), 5, 10 and 15 mW/cm^2 (measurement)<br><br>Whole body exposure<br><br>Exposure duration: 30 min/session (unknown number of sessions)<br><br>Remarks: animals probably restrained	Behavioral changes: respond to multiple schedules of reinforcements (food) in conditioned behavior task: differential reinforcement of low rate (DLR) and fixed-ratio (FR) schedules; number of time-out responses	The low rates of responding produced by the DRL schedule increased after exposure, and the high rates of responding produced by the FR schedule decreased after exposure (with exception of the 1 mW/cm^2). The number of responses that occurred during the time-out periods increased after exposure.<br><br>Conclusion: Low level microwave exposure provoked behavioral changes in the different tests.<br><br>(Effect found)	Two additional exposure groups (2450 and 2860 MHz) were irrelevant for review and therefore not extracted

Authors, study design	Study groups	Exposure (Assessment method)	Endpoints	Results Conclusion according to authors (Effect categorization according to authors)	Remarks
**CNS, cognitive functions, and behavior**					
Thuroczy et al. 1994<br><br>EA<br><br>Study quality: 3rd tier	Hybrid F1-rats (anaesthetized and with implanted EEG electrodes)<br><br>Exposure group 1: n=5, Exposure group 2: up to n=5, exposure group 3: n=5, exposure group 4: up to n=5, exposure group 5: n=4, exposure group 6: n=4, exposure group 7: up to n=5, exposure group 8: n=4, sham exposure group 1: up to n=5, sham exposure group 2: n=5<br><br>Not clear if the same or separate animals were used for the different exposure groups and for the sham exposure as well as for 2.45 GHz- and 4 GHz-exposure.	TE-cell waveguide, 4 GHz, CW, AM (16 Hz)<br><br>Up to 10 conditions (depending on endpoint): sham exposure group 1 (CW, 0 mW/g), sham exposure group 2 (AM, 0 mW/g) and 8 exposure groups: group 1 (AM, SAR 8.4 mW/g (average in the cortex)), group 2 (CW, SAR 8.4 mW/g), group 3 (AM, SAR 16.8 mW/g), group 4 (CW, SAR 16.8 mW/g), group 5 (CW, SAR 25.2 mW/g, group 6 (CW, SAR 33.4 mW/g), group 7 (CW, SAR 42 mW/g), group 8 (CW, 56 mW/g)<br><br>(measurement, calculation/simulation)<br><br>Head exposure<br><br>Exposure duration: 30 min	Effects on brain: brain activity (EEG; groups 1, 3, 7), cerebral blood flow (rheoencephalogram (REG); groups 2, 4), brain tissue DC impedance (groups 1, 2, 3, 4) and brain temperature (groups 2, 4, 5, 6, 7, 8)<br><br>Effects on the cardiovascular system (ECG; groups 1, 2, 3, 4, 7)	CW exposure at 42 mW/g caused a significant increase of the power of EEG delta waves simultaneously with the REG amplitude (however, data for REG amplitude at 42 mW/g not provided) as an index of cerebral blood flow. AM exposure (8.4 mW/g) was associated with increased power of EEG alpha and beta waves but changes in the cerebral blood flow were not observed (REG amplitude data not shown for AM exposure). CW exposure at 8.4 mW/g and 16.8 mW/g increased the cerebral blood flow but did not change EEG spectra.<br><br>Conclusion: No changes in the brain activity were observed at low level exposure. However, at thermal level exposure, effects were detected.<br><br>(Effect found)	Only data for 4 GHz exposure extracted, but additional data for 2.45 GHz are available in the publication<br><br>Effects caused by SAR values above 16.8 mW/g were considered as thermal

Authors, study design	Study groups	Exposure (Assessment method)	Endpoints	Results Conclusion according to authors (Effect categorization according to authors)	Remarks
**CNS, cognitive functions, and behavior**					
Van Eeghem et al. 2017<br><br>EA<br><br>Study quality: 3rd tier	NMRI mice<br><br>Exposure group 1: n=14 (2 Hz AM), exposure group 2: n=14 (8 Hz AM), sham exposure group 1: n=14 (2 Hz AM), sham exposure group 2: n=15 (8 Hz AM)	Horn antenna, 10.0 GHz, AM (2 and 8 Hz)<br><br>4 conditions: 2 sham exposure groups, 2 exposure groups (2 Hz AM and 8 Hz AM; SAR 0.3 W/kg) (calculation/simulation)<br><br>Whole body exposure<br><br>Exposure duration: 24 h/day for 6 days<br><br>Remarks: brain temperature increased by 0.23°C during exposure (investigated in 3 animals)	Brain neurochemical changes: levels of dopamine, 3,4-dihydroxyphenylacetic acid and glutamate (immediately or 4 weeks after exposure)<br><br>Locomotor activity, anxiety, depressive-like behaviour, spatial working memory (immediately or 4 weeks after exposure) (Open Field test, Rotarod test, Forced Swim test, Y-maze test)	8 Hz AM significantly reduced locomotor activity immediately after exposure which normalized after 4 weeks.<br><br>Conclusion: Exposure to low-intensity 8 Hz AM microwaves can alter the locomotor activity of mice.<br><br>(Effect found)	
Walani et al. 2014<br><br>EA<br><br>Study quality: 3rd tier	Swiss albino mice<br><br>Exposure group: n=?<br>sham exposure group: n=?	Horn antenna, 10 GHz, CW<br><br>2 conditions: sham exposure and exposure (PD 0.25 mW/cm² (peak), SAR 0.1790 W/kg) (measurement, calculation/simulation)<br><br>Head exposure<br><br>Exposure duration: 2 h/day for 30 days<br><br>Remarks: animals were probably immobilized as they were faced towards antenna	Biochemical and anti-oxidative parameters in brain/cerebellum at 1-30 days follow-up after exposure (levels of lipid peroxidation, protein, reduced glutathione, superoxide dismutase and catalase)	Lipid peroxidation and catalase levels increased while protein, reduced glutathione and superoxide dismutase levels were reduced at all follow-up time points in exposed animals' brain/cerebellum.<br><br>Conclusion: Free radicals causing oxidative stress may be responsible for altered biochemical levels of the mice brain after microwave exposure.<br><br>(Effect found)	Not clear if whole brain or cerebellum was investigated (contradictory specifications).

Authors, study design	Study groups	Exposure (Assessment method)	Endpoints	Results Conclusion according to authors (Effect categorization according to authors)	Remarks
**CNS, cognitive functions, and behavior**					
Wang et al. 2023b<br><br>EA<br><br>Study quality: 3rd tier	Wistar rats<br><br>Exposure group: n=?, sham exposure group: n=?	Horn antenna, 9.375 GHz<br><br>2 conditions: sham exposure and exposure (10 mW/cm²)<br>(no data provided on exposure validation)<br><br>Whole body exposure<br><br>Exposure duration: 6 min/day, 5 days/week for 6 weeks	Spatial learning and memory (Morris water maze test; 12 rats/group)<br><br>Hippocampal microstructure (5 rats/group)<br><br>Hippocampal ultrastructure; dendritic spine density and maturation ratio analysis of neurons in the hippocampus (3 rats/group)<br><br>quantitative proteomic analysis of hippocampal and serum exosomes (5 rats/group)<br><br>EEG (5 rats/group)	Spatial reference learning and memory abilities as well as the hippocampal structure were impaired after exposure. There were 406 and 49 differentially expressed proteins in hippocampus and in serum exosomes, respectively.<br><br>Conclusion: Long-term microwave exposure could lead to spatial learning and memory impairment, structural damage to the hippocampus, and differential expression of hippocampal tissue and serum exosomes as well as different proteins.<br><br>(Effect found)	Data on exposure to 2.856 GHz were not relevant for the review and therefore not extracted.

Authors, study design	Study groups	Exposure (Assessment method)	Endpoints	Results<br>Conclusion according to authors<br>(Effect categorization according to authors)	Remarks
**CNS, cognitive functions, and behavior**					
Yang et al. 2022<br><br>EA<br><br>Study quality: 3rd tier	Guinea pigs<br><br>Exposure group 1: n=10 (2 W/kg), exposure group 2: n=10 (4 W/kg), exposure group 3: n=10 (10 W/kg), sham exposure group: n=10	Horn antenna, 3.5 GHz, CW<br><br>4 conditions: sham exposure and 3 exposure groups (SAR 2, 4, and 10 W/kg)<br>(measurement, calculation/simulation, calibration)<br><br>Whole body exposure<br><br>Exposure duration: 72 h	Auditory brainstem response thresholds<br><br>Anxiety-like behavior (Open Field test)<br><br>Malondialdehyde level and antioxidant enzyme activity (glutathione peroxidase, superoxide dismutase, catalase)<br><br>Ultrastructure of the auditory cortex (TEM)<br><br>Cytochrome-c relocalization, caspase-9, and cleaved caspase-3 expression in the auditory cortex	The level of MDA increased, while the antioxidant enzyme activities decreased. Ultrastructural changes in auditory cortex (e.g., swollen mitochondria and layered myelin sheaths) were observed (no statistics). Cytochrome-c relocalization, caspase-9 and cleaved caspase-3 activation were detected in exposed groups (no statistics).<br><br>Conclusion: The results suggest that oxidative stress is an important mechanism which can induce ultrastructural damage to the auditory cortex and cell apoptosis. Oxidative stress, apoptosis induction and ultrastructural damage increase in a SAR-dependent manner.<br><br>(Effect found)	

Authors, study design	Study groups	Exposure (Assessment method)	Endpoints	Results Conclusion according to authors (Effect categorization according to authors)	Remarks
**CNS, cognitive functions, and behavior**					
Zhang et al. 2015<br><br>EA<br><br>Study quality: 3rd tier	CD1 mice (pregnant)<br><br>Exposure group: n=?, control group: n=?, sham exposure group: n=?	Exposure source not provided, 9.417 GHz<br><br>3 conditions: control, sham exposure and exposure (SAR 2.0 W/kg)<br>(no data provided on exposure validation)<br><br>Whole body exposure<br><br>Exposure duration: 12 h/day during pregnancy days 3.5–18	Behavior and cognition in offspring: anxiety-related behavior (Open Field test, Elevated-plus maze), depression-related behavior (tail suspension test, forced swimming test), learning and memory (Morris water maze)	Exposed mice showed less movement in the center of an Open Field test and in an open arm of the Elevated-plus maze suggesting increased anxiety-related behavior. Moreover, exposed mice demonstrated reduced immobility in tail suspension and forced swim test, which suggested that the mice had decreased depression-related behavior. In the Morris water maze, only male offspring demonstrated decreased learning and memory, suggesting gender-dependent effects.<br><br>Conclusion: Prenatal exposure of mice led to neurobehavioral alterations/disorders and showed gender-dependent effects on learning and memory deficits.<br><br>(Effect found)	12 pregnant rats were divided into 3 groups (groups sizes not stated); number of pups used for tests only partially provided in results (n=12 or 8/group)

Authors, study design	Study groups	Exposure (Assessment method)	Endpoints	Results Conclusion according to authors (Effect categorization according to authors)	Remarks
**Immune system**					
Baranski 1972b<br><br>EA<br><br>Study quality: 3rd tier	Guinea pigs<br><br>Exposure group 1: n=50,<br>exposure group 2: n=50,<br>control group: n=50	Exposure source not provided, 3 GHz, PW, CW<br><br>3 conditions: control group and 2 exposure groups (PD 3.5 mW/cm^2): group 1 (PW) and group 2 (CW)<br><br>Exposure duration: 3 h/day for 3 months	Morphological changes, cell number, mitotic index (5 animals/group), cell maturation, ^{3}H-thymidine incorporation (10 animals/group) of peripheral blood cells and blood cells in bone marrow, lymph nodes and spleen (e.g., lymphocytes, granulocytes)	There was an increase in absolute number of lymphocytes in peripheral blood as well as an increase in cells incorporation ^{3}H-thymidine in lymph nodes and spleen of both exposure groups. Histological observations suggested a shift of lymphocyte population into the direction of less differentiated forms and changes in lymph node structure, especially after PW exposure (no statistics).<br><br>Conclusion: Exposure to 3 GHz microwaves induced lymphopoesis, based on an increased lymphocyte count in the peripheral blood and a shift of lymphocytic population into the direction of less differentiated forms in the blood forming organs.<br><br>(Effect found)	

Authors, study design	Study groups	Exposure (Assessment method)	Endpoints	Results Conclusion according to authors (Effect categorization according to authors)	Remarks
**Immune system**					
Dasdag et al. 2001<br><br>EA<br><br>Study quality: 3rd tier	Albino Wistar rats<br><br>Exposure group: n=8, sham exposure group: n=8	Exposure source not provided, 9.45 GHz<br><br>2 conditions: sham exposure and exposure (PD 2.65 mW/cm², SAR 1.80 W/kg)<br>(measurement)<br><br>Whole body exposure<br><br>Exposure duration: 1 h/day for 21 days	Phagocytic activity of peritoneal and alveolar macrophages<br><br>Rectal temperature	Rectal temperature increase was higher in the exposure group compared to the sham exposure group.<br><br>Conclusion: Microwave exposure increased the body temperature but not the phagocytic activity.<br><br>(No effect found)	Groups (co-)exposed to EMF and vitamins E or a magnetic field were not relevant for review and therefore not extracted
Glushkova et al. 2007<br><br>EA<br><br>Study quality: 3rd tier	NMRI mice<br><br>exposure group: n=?, control group: n=?	Horn antenna, 8.15-18 GHz, swept frequency (direct and reversed sweep times were 1.0 s and 16 ms, respectively)<br><br>2 conditions: sham exposure and exposure (PD 0.2-1.6 µW/cm², 1 µW/cm² (spatial average), SAR 2-5 mW/kg)<br>(measurement)<br><br>Whole body exposure<br><br>Exposure duration: 1 h/day for 10 days	Indices of immune status: counts of thymocytes, macrophages, splenocytes, production of TNFα, interleukins 1α, 1β, 2, 6, and 10, IFNγ, NO, and expression Hsp 27, 72, and 90α	Exposure increased numbers of thymocytes, macrophages, splenocytes as well as TNF, IL 6 and HSp 72, but decreased NO and IFN.<br><br>Conclusion: No conclusion (only conclusion for co-exposure stated)<br><br>(Effect found)	groups co-exposed to EMF and acute intoxication with *E. coli* were not relevant for the review and were therefore not extracted

Authors, study design	Study groups	Exposure (Assessment method)	Endpoints	Results Conclusion according to authors (Effect categorization according to authors)	Remarks
**Immune system**					
Szmigielski et al. 1975<br><br>EA<br><br>Study quality: 3rd tier	Rabbits<br><br>exposure group 1: n=5,<br>exposure group 2: n=5, control group: n=5	Conical antenna, 3 GHz<br><br>3 conditions: control and 2 exposure groups (PD 3 mW/cm²): group 1 (6 h/day for 6 weeks), group 2 (6 h/day for 3 months)<br>(no data provided on exposure validation)<br><br>Whole body exposure<br><br>Exposure duration: 6 h/day for 6 weeks or 3 months<br><br>Remarks: after exposure, animals were infected with *Staphylococcus aureus*	Granulopoiesis (before, 4, 6, 10, and 14 days after infection): Leukocyte and granulocyte number in peripheral blood, bone marrow picture, reserve pool mobilization after staphylococcal alpha-toxin injection, lysozyme activity in blood serum, lysis of resuspended lyophylized *Micrococcus lysodeikticus*	Exposed rabbits showed a decreased leukocytosis and granulocytosis 10 and 14 days after infection. These changes were accompanied by a decline in the bone marrow reserve pool and lysozyme activity during the entire observation period. Effects were in general more pronounced in rabbits exposed for 3 months compared to 6 weeks (no statistics for all results).<br><br>Conclusion: Exposure to low level microwaves for several weeks might result in decreased production of mature granulocytes in the bone marrow.<br><br>(Effect found)	
Szmigielski et al. 1977b<br><br>EA<br><br>Study quality: 3rd tier	Tumor bearing CFW mice<br><br>exposure group: n=36,<br><br>sham exposure group: n=48	Exposure source not provided, 3 GHz<br><br>2 conditions: sham exposure and exposure (PD 40 mW/cm² ± 10%) (measurement)<br><br>Whole body exposure<br><br>Exposure duration: 2 h/day for 15 days	Tumor growth: tumor mass, number of regressed tumors, incorporation of ^{3}H-thymidine and ^{3}H-uridine	Exposure resulted in decreased tumor mass and a higher number of regressed tumors. This was accompanied by a reduced incorporation of ^{3}H-thymidine (no statistics).<br><br>Conclusion: Microwave exposure led to an inhibition of tumor growth.<br><br>(Effect found)	Groups (co-)exposed to EMF and Colcemide and/or Streptolysin S were not relevant for review and therefore not extracted

Authors, study design	Study groups	Exposure (Assessment method)	Endpoints	Results Conclusion according to authors (Effect categorization according to authors)	Remarks
**Immune system**					
Szmigielski et al. 1977a<br><br>EA<br><br>Study quality: 3rd tier	NMRI mice<br><br><u>HSV-1 infected mice:</u><br>exposure groups 1-4: n=18-30<br><br><u>Vaccinia infected mice:</u><br>exposure groups 5-12: n=?<br><br>Control group 1 (HSV-1): n=32, control group 2 (vaccinia): n=?	Conical antenna, 3 GHz, CW<br><br>14 conditions: 2 control groups and 12 exposure groups (PD 40 mW/cm² ± 10%); different exposure durations starting at different days before/after infection (measurement)<br><br>Whole body exposure<br><br>Exposure duration: 2 hours/day for 5-15 days (up to 7 days before and up to 12 days after virus infection)	Mortality (in animals infected with HSV-1 virus)<br><br>Number of pox tail lesions (in animals infected with vaccinia virus)	Hyperthermia through microwave exposure after HSV-1 infection resulted in a decrease of the mortality rate (HSV-1) and number of tail lesions (vaccinia).<br><br>Conclusion: Microwave hyperthermia resulted in a marked inhibition of virus multiplication immediately after infection. Hyperthermia before infection or initiated several days after virus infection showed little or no effect.<br><br>(Effect found)	*In vitro* experiments irrelevant for review and therefore not extracted<br><br>Maybe temperatures > 42°C reached

Authors, study design	Study groups	Exposure (Assessment method)	Endpoints	Results Conclusion according to authors (Effect categorization according to authors)	Remarks
**Immune system**					
Yao et al. 2022<br><br>EA<br><br>Study quality: 3rd tier	Wistar rats<br><br>Exposure group 1: n=20 (?)<br>sham exposure group: n=20 (?)	Horn antenna, 9.3 GHz, PW<br><br>2 conditions: sham exposure and exposure (PD 10 mW/cm²(average))<br>(no data provided on exposure validation)<br><br>Whole body exposure<br><br>Exposure duration: 6 min	Histopathology of thymus and spleen (6 h and on days 7, 14 and 28 after exposure), ultrastructure of spleen (on day 7 after exposure)<br><br>Count of white blood cells, lymphocytes and neutrophils in peripheral blood; differential count of lymphocytes (B, T, CD4+ and CD8+) in peripheral blood (6 h and on days 7 and 28 after exposure)<br><br>Contents of cytokines and chemokines related to the proliferation, maturation and activation of B lymphocytes and T lymphocytes (not further specified) in the serum (6 h and on day 7 after exposure)<br><br>Transcriptome and proteome analysis of peripheral blood and spleen (on day 7 after exposure), gene expressions of *Ahcy, Atic, Dnaja1, Il17ra, Kiaa0408L, Mal, Nxf1, Pglyrp1, Sept1, Sowahb, Was* and *Zbtb1*	7 and 14 days after exposure, rats showed injuries in thymus and spleen, which disappeared 28 days after exposure. The percentage of B lymphocytes was increased 6 h and 7 days after exposure while T lymphocytes were decreased 7 days after exposure and returned to baseline 28 days after exposure in blood. Concentrations of cytokines IL-1α, IL-1β and IL-4 were increased after 6 h but were restored to baseline level 7 days after exposure. Moreover, T-cell related cytokines TNF-α and IL-12 were increased 6 h after exposure. Transcriptomic and proteomic analysis showed many differentially expressed genes and proteins related to metabolism and immune-related signaling pathways in blood and spleen.<br><br>Conclusion: Exposure might cause reversible injuries in thymus and spleen and activate immune cells in the peripheral blood by upregulating mRNA and protein expression, as well as cytokine release.<br><br>(Effect found)	Data of exposure to 2.8 GHz and combined exposure to 2.8 and 9.3 GHz were not relevant for the review and therefore not extracted

Authors, study design	Study groups	Exposure (Assessment method)	Endpoints	Results Conclusion according to authors (Effect categorization according to authors)	Remarks
**Immune system**					
Zhao et al. 2022<br><br>EA<br><br>Study quality: 3rd tier	Wistar rats<br><br>Exposure group: n=25 (?),<br>sham exposure group: n=25 (?)	Horn antenna, 4.3 GHz<br><br>2 conditions: sham exposure and exposure (PD 10 mW/cm² (average) (measurement)<br><br>Whole body exposure<br><br>Exposure duration: not stated<br><br>Remarks: rats restrained in fan-shaped boxes	Counts of immune cells in peripheral blood (white blood cells, lymphocytes (+subtypes), neutrophils; after 6 h and on days 7 and 14)<br><br>Secretion of cytokines in peripheral blood (interleukin (IL)-1α, IL-4, IL-6, IL-10, IL-17A and IFN-γ; after 6 h and on day 7)<br><br>Histopathological analysis of thymus, spleen tissue and bone marrow (after 6 h and on days 7, 14 and 28)<br><br>Ultra-structural analysis of spleen (on day 7)<br><br>Further analysis of spleen and peripheral blood (transcriptomics, proteomics, RT-PCR, mass spectrometry) (on day 7)	For up to 7 days, significant histopathological (e.g., congestion) and ultrastructural changes (e.g., swollen mitochondria) were detected in thymus and spleen and spleen alone, respectively (no statistics).<br>Additionally, the white blood cells and lymphocytes in peripheral blood were decreased at 6 h after exposure and increased on day 14 after exposure. B lymphocytes were increased while the T lymphocytes were decreased at 6 h after exposure. All these effects gradually returned to baseline 14 days after exposure. Results of transcriptomic and proteomic analysis showed that microwaves differentially expressed numerous genes and proteins associated with immune regulation and cellular metabolism in peripheral blood and in the spleen.<br><br>Conclusion: Exposure to 4.3 GHz microwaves could cause immune suppression in rats.<br><br>(Effect found)	Further groups with (co-)exposure to a 1.5 GHz EMF were not relevant for review and therefore not extracted

Authors, study design	Study groups	Exposure (Assessment method)	Endpoints	Results<br>Conclusion according to authors<br>(Effect categorization according to authors)	Remarks
**Reproduction & Teratogenicity**					
Akdag et al. 1999b<br><br>EA<br><br>Study quality: 3rd tier	Sprague-Dawley rats<br><br>Exposure group 1: n=10,<br>exposure group 2: n=10,<br>exposure group 3: n=10,<br>exposure group 4: n=10<br><br>Sham exposure group 1: n=10,<br>sham exposure group 2: n=10,<br>sham exposure group 3: n=10,<br>sham exposure group 4: n=10	Horn antenna, 9.450 GHz, CW<br><br>8 conditions: 4 sham exposure groups (same durations as exposure groups) and 4 exposure groups (PD 2.65 mW/cm², electric field strength 100 V/m, SAR 1.80 W/kg (mean whole body)): group 1 (13 days exposure), group 2 (26 days), group 3 (39 days), group 4 (52 days) (measurement, calculation/simulation)<br><br>Whole body exposure<br><br>Exposure duration: 1 h/day for 13, 26, 39, and 52 days<br><br>Remarks: exposure durations corresponded to 1, 2, 3, and 4 cycles of the seminiferous epithelium, respectively	Left epididymal sperm count, percentage of normal/abnormal sperm, masses of right and left testes and epididymes, seminal vesicles, and prostate, histology of right testis and epididymis<br><br>Rectal temperature	Sperm counts were reduced after 52 days of exposure. Normal spermatocyte percentage decreased after 26, 39, and 52 days of exposure while abnormal spermatocyte percentages increased for the same exposure periods. Masses of few single organs were different after 26, 39, and 52 days of exposure. Histopathological abnormalities were found in testes (e.g., necrosis) and epididymes (e.g., edema) in all exposure groups (no statistics). Rectal temperatures of rats after exposure were found to be lower after 26 and 39 days of exposure compared to the sham exposure.<br><br>Conclusion: Sperm count and morphology as well as weight and morphology of testis and epididymes were affected by microwave exposure depending on duration of exposure.<br><br>(Effect found)	

Authors, study design	Study groups	Exposure (Assessment method)	Endpoints	Results Conclusion according to authors (Effect categorization according to authors)	Remarks
**Reproduction & Teratogenicity**					
Chiang and Yao 1987<br><br>EA<br><br>Study quality: 3rd tier	Kunming mice (pregnant dams and offspring)<br><br>Pregnant mice<br>exposure group 1: n=? (subgroup of n=60)<br><br>Offspring<br>exposure group 2: n=? (half of the offspring of exposed dams),<br>exposure group 3: n=? (half of the offspring of sham-exposed dams)<br><br>Sham exposure<br>group 1: n=? (subgroup of n=60 dams),<br>group 2: n=? (half of the offspring of exposed dams),<br>group 3: n=? (half of the offspring of sham-exposed dams)	No details provided on exposure source, 3.0 GHz, PW (rate 937 pps, width 1.2 µs)<br><br>6 conditions: 3 sham exposure and 3 exposure groups: group 1 (PD 8 mW/cm², SAR 3.0-3.5 mW/g), groups 2-3 (PD 1 mW/cm²) (measurement)<br><br>Whole body exposure<br><br>Exposure duration: 5 h/day during pregnancy and from day 3-20 after birth<br><br>Remarks: pregnant mice exposed with their bodies parallel to the field; no core temperature rise in the mice after microwave exposure	Enzyme levels in the brain: mitochondrial marker enzyme activities (succinate dehydrogenase, catecholamine, monoamine oxidase levels)<br><br>Enzyme levels in the liver (succinate dehydrogenase level)	Succinate dehydrogenase levels in the hypothalamus and liver were reduced by either pre- or post-natal microwave exposure. The same pattern appeared with hypothalamic catecholamine and monoamine oxidase.<br><br>Conclusion: The data indicated that low intensity microwave exposure can induce subtle alterations in enzyme activities/levels in offspring of mice.<br><br>(Effect found)	Short Communication

Authors, study design	Study groups	Exposure (Assessment method)	Endpoints	Results Conclusion according to authors (Effect categorization according to authors)	Remarks
**Reproduction & Teratogenicity**					
Gunn et al. 1961<br><br>EA<br><br>Study quality: 3rd tier	Wistar rats<br><br>exposure group: n=30,<br>co-exposure group: n=30<br>control group: number not specified	Horn antenna, 24.0 GHz, PW (duty cycle 0.0006)<br><br>3 conditions: control group, exposure, and co-exposure group (+ hormone administration)<br>(PD 0.250 W/cm²)<br>(calculation/simulation)<br><br>Exposure of scrotal area<br><br>Exposure duration: 5 minutes<br><br>Remarks: animals anesthetized	Pituitary-testis-prostate endocrine chain: Zn65 uptake in dorsolateral prostate, histopathology of prostate and testes	Up to 13 days following the exposure, the prostate Zn65 uptake was decreased (statistically significant) and mild to moderate histological changes could be detected in the testes (no statistics). Administration of either chorionic gonadotrophin or testosterone restored the prostate Zn65 uptake to control level.<br><br>Conclusion: Exposure resulted in mild testicular damage with decreased androgen levels, probably caused by insufficient luteinizing hormone output by the pituitary.<br><br>(Effect found)	Exposure groups with exposures for 10 and 15 min were not relevant because of temperature increase >42 °C and therefore not extracted

Authors, study design	Study groups	Exposure (Assessment method)	Endpoints	Results Conclusion according to authors (Effect categorization according to authors)	Remarks
**Reproduction & Teratogenicity**					
Jensh 1984b<br><br>EA<br><br>Study quality: 3rd tier	Wistar albino rats (pregnant)<br><br>exposure group 1: n=11,<br>control group: n=10,<br>cage control: n=5, sham exposure group: n=10	Horn antenna, 6.0 GHz<br><br>4 conditions: cage control, exposure chamber control, sham exposure, exposure (PD 35 mW/cm², SAR 7.28 W/kg (average)) (measurement)<br><br>Whole body exposure<br><br>Exposure duration: 8 h/day for 21 days during pregnancy<br><br>Remarks: power density was determined in preliminary experiments to not increase body temperature	Blood analysis, weight and histology of ovaries, liver, kidneys, and brain (P generation after rebreeding and F1a at age of 100 days)<br><br>Teratologic evaluation (F1b, F2 and F2 offspring): number of fetuses, neonates, abnormal neonates<br><br>Body weight (weekly, F1a)<br><br>Fertility (F1a females bred with exposed or non-exposed F1a males at age of 90-120 days): fertility rate and litter size<br><br>Behavioral tests (F1a): Physical feature-eye opening, neonatal reflex tests (surface righting, negative geotaxis, auditory startle, visual placing, air righting), adult behavioral and cognitive tests (at age of 60-90 days): open field, activity wheel, swimming, hanging, avoidance test, water T-maze test	Exposed dams and F1b fetuses weighed less than the sham exposure. Exposed F1a fetuses opened their eyes earlier and weighed less at several days postnatally than the sham exposure. Exposed F1a females showed decreased achievement in water T-maze, differences in the avoidance test and postnatal growth to the fifth week compared to sham. *In utero* exposed pregnant F1a females showed a decreased weight gain and litter size when mated with *in utero* exposed F1a males compared to sham exposed F1a mated with sham exposed F1a males. Several organ/body weights and blood parameters were different in *in utero* exposed F1a animals compared to sham exposed animals.<br><br>Conclusion: 6.0 GHz *in utero* exposure may result in subtle long-term neurophysiologic alterations.<br><br>(Effect found)	P generation: exposed dams,<br>F1a generation: offspring exposed *in utero*,<br>F1b generation: offspring from further pregnancy of P generation,<br>F2 generation: F1a offspring

Authors, study design	Study groups	Exposure (Assessment method)	Endpoints	Results Conclusion according to authors (Effect categorization according to authors)	Remarks
**Reproduction & Teratogenicity**					
Manikowska et al. 1979<br><br>EA<br><br>Study quality: 3rd tier	Balb/c mice<br><br>Exposure group 1: n=4, exposure group 2: n=4, exposure group 3: n=4, exposure group 4: n=4, control group: n=7	9.4 GHz, PW (width 0.5 µs, rate 1000 Hz)<br><br>5 conditions: control group and 4 exposure groups (PD 0.1, 0.5, 1.0 and 10.0 mW/cm²)<br>(no data provided on exposure validation)<br><br>Exposure duration: 1 h/day for 2 weeks (5 days a week)	Meiosis in spermatocytes and spermatogonia from Seminiferous tubules: number of meiotic metaphases (M1 and M2), spermatogonial metaphases (MG) and number and structure of chromosomes in M1 metaphase	All metaphase counts were reduced after exposure to 0.1 mW/cm² and the M2 count was reduced following exposure to 0.5 mW/cm². The numbers of translocations (metaphases with quadri- or hexavalents) were increased after 0.1, 1.0 and 10.0 mW/cm² exposures while the number of metaphases with univalents was increased in all exposure groups.<br><br>Conclusion: Exposure to 9.4 GHz pulsed microwaves disturbed meiosis.<br><br>(Effect found)	

Authors, study design	Study groups	Exposure (Assessment method)	Endpoints	Results Conclusion according to authors (Effect categorization according to authors)	Remarks
**Reproduction & Teratogenicity**					
Sharma et al. 2017a<br><br>EA<br><br>Study quality: 3rd tier	Swiss albino mice (*in utero*)<br><br>Exposure group 1: n=6 dams (number of pups not provided),<br>exposure group 2: n=6 dams (number of pups not provided),<br>sham exposure group 1: n=6 (number of pups not provided,<br>sham exposure group 2: n=6 (number of pups not provided)	Pyramidal horn antenna, 10.0 GHz<br><br>4 conditions: 2 sham exposure groups and 2 exposure groups (PD 0.25 mW/cm² (max.), SAR 0.179 W/kg): group 1 (exposure from day 0.25), group 2 (exposure from day 11.25)<br>(measurement, calculation/simulation)<br><br>Whole body exposure (*in utero*)<br><br>Exposure duration: 2 h/day from 0.25 and 11.25 day of gestation until birth (20 days (exposure/sham exposure groups 1) and 8 days (exposure/sham exposure groups 2))<br><br>Remarks: dams were placed towards the midline of the antenna and were lightly restrained with heads facing antenna	Effects on developing brain (at the age of 3 weeks of pups): brain weight, biochemical changes (glutathione assay, lipid peroxidation assay, total protein level (6 pups/group)), histopathology (6 pups/group)<br><br>Effects on pregnancy outcome: litter size, body weight of litters, crown-rump length of litters	The data showed a significant reduction in the brain weight (except exposure group 2) and body weight of the exposed groups as well as an increased level of lipid peroxidation, decreased level of glutathione and protein. Moreover, changes in cytoarchitecture of hippocampus and cerebellum of the brain and a reduction in Purkinje cell numbers were observed after microwave exposure.<br><br>Conclusion: The degree of severity of damage in neonatal mice brain was higher when exposure started from 0.25 day of gestation compared to 11.25 days of gestation.<br><br>(Effect found)	

Authors, study design	Study groups	Exposure (Assessment method)	Endpoints	Results Conclusion according to authors (Effect categorization according to authors)	Remarks
**Reproduction & Teratogenicity**					
Varma and Traboulay 1975<br><br>EA<br><br>Study quality: 3rd tier	Swiss mice<br><br>Exposure group: number not specified, sham exposure group: n=8	3.0 GHz, continuous pulse (?)<br><br>2 conditions: sham exposure and exposure (PD 50 mW/cm²)<br>(no data provided on exposure validation)<br><br>Exposure of scrotum<br><br>Exposure duration: 20 min<br><br>Remarks: animals anesthetized	Testicular histology	Mild disintegration of nuclear material in lumens (no statistics).<br><br>Conclusion: Exposure to the 3 GHz field showed only minimal injuries.<br><br>(Effect found)	Data on exposure to 1.7 GHz was not relevant for review and therefore not extracted

Authors, study design	Study groups	Exposure (Assessment method)	Endpoints	Results<br>Conclusion according to authors<br>(Effect categorization according to authors)	Remarks
**Effects on eyes**					
Akdag et al. 2002<br><br>EA<br><br>Study quality: 3rd tier	Wistar albino rats<br><br>exposure group: n=8, sham exposure group: n=8	Exposure source not provided, 9.45 GHz<br><br>2 conditions: sham exposure and exposure (PD 2.65 mW/cm², SAR 1.8 W/kg (whole body))<br>(no data provided on exposure validation)<br><br>Whole body exposure<br><br>Exposure duration: 1 h/day for 21 days<br><br>Remarks: animal's long axis of the body placed parallel to the electric field	Effects on eyes: daily microscopical investigation; histopathological changes and oxidative stress (malondialdehyde level) (at the end of exposure)<br><br>Body temperature	Conjunctiva: sero-hemorrhagic dark brown secretion was observed in exposed group.<br>Lens fibers: vacuolization, pleomorphism, pyknosis and increasing of cell layers were found in exposed group.<br>Cornea: cellular loss, shape and size differences among cells and lack of polarity were found in exposed animals.<br>Retina: slight edema, congestion, desquamation in epithelial cells, separation between layers in some areas, and alteration in pigment cells were determined in exposed rats (no statistics provided).<br>Malondialdehyde levels increased in the exposure group. Rectal temperature before and after exposure was different compared to control group.<br><br>Conclusion: Microwave exposure caused histopathologic alternations and lipid peroxidation in rat eyes.<br><br>(Effect found)	Data on co-exposure to microwaves and vitamin E resp. vitamin C were not extracted.

Authors, study design	Study groups	Exposure (Assessment method)	Endpoints	Results Conclusion according to authors (Effect categorization according to authors)	Remarks
**Effects on eyes**					
Appleton et al. 1975<br><br>EA<br><br>Study quality: 3rd tier	New Zealand albino rabbits<br><br>Exposure groups 1-7: n=3 each,<br>exposure group 8: n=6,<br>control group: n=3,	Dish antenna, 3.0 GHz, CW<br><br>9 conditions: control group and 8 exposure groups: group 1 (PD 100 mW/cm² for 15 min), group 2 (PD 100 mW/cm² for 30 min), group 3 (200 mW/cm² for 15 min), group 4 (200 mW/cm² for 30 min), group 5 (300 mW/cm² for 15 min), group 6 (300 mW/cm² for 30 min), group 7 (400 mW/cm² for 15 min), group 8 (500 mW/cm² for 15 min) (measurement, calculation/simulation)<br><br>Exposure of left eye<br><br>Exposure duration: 15 min and 30 min<br><br>Remarks: animals anesthetized during exposure	Ocular changes: acute changes (hyperemia of lids and conjunctiva, miosis and anterior chamber flare, engorgement of iris vessels, periorbital cutaneous burn), long-term ocular changes (lens changes, cataract; examinations over a one-year period)	Acute changes were found in animals exposed to 300-500 mW/cm² for 15 min which were reversible after 24 h. All animals died during exposure to 300 mW/cm² for 30 min, and 3 of 6 animals immediately died after exposure to 500 mW/cm² for 15 min. Severe stress was observed in surviving animals (no statistics applied).<br><br>Conclusion: Exposure up to 200 mW/cm² induced no effect and ≥ 300 mW/cm² induced reversible acute ocular effects.<br><br>(Effect found)	

Authors, study design	Study groups	Exposure (Assessment method)	Endpoints	Results Conclusion according to authors (Effect categorization according to authors)	Remarks
**Effects on eyes**					
Crouzier et al. 2014<br><br>EA<br><br>Study quality: 3rd tier	New Zealand rabbits<br><br>exposure group 1: n=15 (+ surgery),<br>exposure group 2: n=15 (+ sham surgery),<br>sham exposure group 1: n=15 (+ surgery),<br>sham exposure group 2: n=15 (+ sham surgery)	Horn antenna, 9.71 GHz, PW (width 9000 ns, 1% duty cycle)<br><br>4 conditions: 2 sham exposure groups (surgery and sham surgery) and 2 exposure groups (PD 50 W/m², SAR 6.35 W/kg): group 1 (surgery), group 2 (sham surgery) (measurement, calculation/simulation)<br><br>Exposure duration: 1 h/day, 3 days/week for 5 months<br><br>Remarks: exposure mimicked chronic occupational exposure to radar	Healing process of operated eyes: clinical examination (at 3 and 6 months after surgery), histological examination (cornea), lens transparency, lens metabolism, lipid composition (lens and cornea), oxidative stress in plasma and vitreous and aqueous humor	No effect found.<br><br>Conclusion: No substantial deleterious effect was identified on eye healing or eye structure under exposure conditions.<br><br>(No effect found)	The rabbits were either treated with LASIK keratotomy or had a sham surgery one month prior exposure.

**Authors, study design**	**Study groups**	**Exposure (Assessment method)**	**Endpoints**	**Results Conclusion according to authors (Effect categorization according to authors)**	**Remarks**
**Effects on eyes**					
Paulsson et al. 1979<br><br>EA<br><br>Study quality: 3rd tier	Pigmented rabbits (black and white Dutch breed)<br><br>exposure group 1: n=13 (?),<br>exposure group 2: n=9 (?),<br>exposure group 3: n=8 (?),<br>exposure group 4: n=2 (?),<br>control group: n=22 (?)	Horn antenna, 3.1 GHz, PW (width 1.4 µs and rate 300 Hz)<br><br>5 conditions: control group and 4 exposure groups (PD 550 W/m²(estimated mean) +/- 20% with pulse peak intensity of 1.3 x 10^6 W/m²):<br>group 1 (exposure for 1 h/day, 3 d/week up to about 50 h),<br>group 2 (1.5 h),<br>group 3 (1 h followed by a drug inducing 70-100 % blood-pressure increase),<br>group 4 (1 h, killed after 3.5 months)<br>(measurement, calculation/simulation)<br><br>Head exposure (retina)<br><br>Exposure duration: 1 h, 1.5 h and 1 h/day, 3 days/week for about 50 h<br><br>Remarks: not anesthetized, therefore probably restrained (not specified)	Ocular changes: retinal morphology, retinal histopathological changes<br><br>Blood tissue barrier studies: blood-brain barrier changes, blood-retina barrier changes<br><br>General observations: weight, appearance, motility, pupillary reflex, lens appearance	Electron microscopic examination showed distinct degenerative changes in the retina of the repeatedly exposed animals (exposure group 1) and mild changes in animals killed after 3.5 months (exposure group 4) (no statistics).<br><br>Conclusion: Ultrastructural changes can occur in rabbit retina due to repeated pulsed 550 W/m² at 3.1 GHz.<br><br>(Effect found)	

**Authors, study design**	**Study groups**	**Exposure (Assessment method)**	**Endpoints**	**Results Conclusion according to authors (Effect categorization according to authors)**	**Remarks**
**Blood parameters**					
Baranski 1971<br><br>EA<br><br>Study quality: 3rd tier	Guinea pigs, rabbits<br><br><u>Guinea pigs</u><br>exposure group 1: n=50,<br>exposure group 2: n=50,<br>control group 1: n=50<br><br><u>Rabbits</u><br>exposure group 3: n=50,<br>exposure group 4: n=50,<br>control group 2: n=50	3 GHz, CW and PW<br><br>6 conditions: 2 control groups and 4 exposure groups (PD 3.5 mW/cm²) groups 1 and 3 (CW), groups 2 and 4 (PW)<br>(measurement)<br><br>Exposure duration: 3 hours/day for up to 4 months<br><br>Remarks: animals immobilized	Blood parameters: red blood cell count (only Guinea pigs), white blood cell count, differential count (immediately, 1, 2, 3, and 4 weeks after exposure for 3 months)<br><br>Microscopic examination of peripheral blood, bone marrow, spleen and lymph nodes (only in Guinea pigs)<br><br>Mitotic indices (only in Guinea pigs directly after 3 and 4 months of exposure and 1 one month after exposure for 3 months)	After 3 months of exposure, white blood cell count increased in guinea pigs and rabbits through lymphocytosis. Nuclear structure and mitosis showed abnormalities in erythroblastic cells in the bone marrow and in lymphoid cells, in lymph nodes and spleen. There was a marked depression in pro-erythroblasts and basophilic erythroblasts, and the maturation curve was shifted to poly-a and orthochromatic erythroblasts (stronger after PW than after CW).<br><br>Conclusion: A marked stimulation of the lymphocytic system and disturbances in erythropoiesis were found.<br><br>(Effect found)	
Deich-mann et al. 1964<br><br>EA<br><br>Study quality: 3rd tier	CFN albino rats<br><br>exposure group: n=10, sham exposure group: n=10	Horn antenna, 24.0 GHz, PW (duty cycle 0.0006)<br><br>2 conditions: sham exposure and exposure (PD 20 mW/cm²)<br>(calculation/simulation)<br><br>Whole body exposure<br><br>Exposure duration: 10 min<br><br>Remarks: animals restrained	Hemopoietic effects (numbers of erythrocytes, total leukocytes, polymorphonuclear leukocytes, lymphocytes)	Exposure induced a decrease in the leukocyte count.<br><br>(Effect found)	Data of experiments lacking an adequate control group (experiments 1, 2 and 4 in the publication) were not extracted.

Authors, study design	Study groups	Exposure (Assessment method)	Endpoints	Results Conclusion according to authors (Effect categorization according to authors)	Remarks
**Blood parameters**					
McAfee et al. 1985<br><br>EA<br><br>Study quality: 3rd tier	Rhesus monkeys<br><br>Exposure group 1: n=6 (unclear if 6 animals in total or per exposure condition, animals of exposure group also used as sham exposure group in some experiments),<br><br>exposure group 2: n=6 (unclear if 6 animals in total or per exposure condition),<br><br>sham exposure group: n=6	Horn antenna (radar), 9.31 GHz, PW (width 0.5 µs, rate 1050 pps (150 mW/cm² condition) or 2300 pps (300 mW/cm² condition))<br><br>3 conditions: sham exposure and 2 exposure groups: group 1 (PD 150 mW/cm², SAR 20 mW/g (average)), group 2 (PD 300 mW/cm², SAR 40 mW/g) (average))<br>(measurement, calculation/simulation)<br><br>Exposure of face and eyes<br><br>Exposure duration: up to 15 minutes (accumulated) per session; total of 169 ± 11 min or 317 ± 5.1 min (exposure group 1); total of 428 ± 39 min (exposure group 2)	Effects on blood parameters (e.g., red blood cells, hemoglobin, hematocrit, protein, albumin, globulin)	Only inconsistent and transient effects were found.<br><br>(No effect found)	Monkeys were trained to expose their faces and eyes to microwave via an operant behavior task.

Authors, study design	Study groups	Exposure<br>(Assessment method)	Endpoints	Results<br>Conclusion according to authors<br>(Effect categorization according to authors)	Remarks
**Heart & CVS**					
Drobakhin et al. 2020<br><br>EA<br><br>Study quality: 3rd tier	Rats (partly thyroidectomized as hypothyroidism model)<br><br>Exposure group 1: n=21 (normal),<br>exposure group 2: n=21 (thyroidectomized),<br>exposure group 3: n=21 (normal),<br>exposure group 4: n=21 (thyroidectomized),<br>control group 1: n=21 (normal),<br>control group 2: n=21 (thyroidectomized)	Horn antenna, 10.0 GHz<br><br>6 conditions: 2 control groups and 4 exposure groups (PD 1,1 mW/m²): groups 1+2 (45 min), groups 3+4 (120 min)<br>(no data provided on exposure validation)<br><br>Whole body exposure<br><br>Exposure duration: 45 min or 120 min/day for 10 days	Morphological changes of the myocardium of the heart (only in animals after thyroidectomy?)<br><br>Peripheral blood parameter changes (on 11th day; only in healthy animals?)	After 120 min of exposure, an increase in stab and segmented neutrophils, monocytes, the appearance of young forms of leukocytes, erythrocytopenia, anemia and lymphocytopenia was found. In the myocardium of the hypothyroidism model, destructive changes and processes of muscle fiber degeneration were observed.<br>(No statistics provided)<br><br>Conclusion: Exposure for 45 min showed a regenerative compensatory restructuring of the structural components of the heart wall in thyroidectomized animals, whereas exposure for 120 min showed an increase in destructive and degenerative processes.<br><br>(Effect found)	Data probably also published in parts by Drobakhin (2021)

Authors, study design	Study groups	Exposure (Assessment method)	Endpoints	Results Conclusion according to authors (Effect categorization according to authors)	Remarks
**Heart & CVS**					
Drobakhin et al. 2021<br><br>EA<br><br>Study quality: 3rd tier	Rats (partly thyroidectomized as hypothyroidism model)<br><br>Exposure group 1: n=21 (normal),<br>exposure group 2: n=21 (thyroidectomized),<br>exposure group 3: n=21 (normal),<br>exposure group 4: n=21 (thyroidectomized),<br>control group 1: n=21 (normal),<br>control group 2: n=21 (thyroidectomized)	Horn antenna, 10.0 GHz<br><br>6 conditions: 2 control groups and 4 exposure groups (PD 1,1 mW/m²): groups 1+2 (45 min), groups 3+4 (120 min)<br>(no data provided on exposure validation)<br><br>Whole body exposure<br><br>Exposure duration: 45 min or 120 min (per day?) for 10 days	Effect on heart morphogenesis after thyroidectomy and normal conditions (CD34 expression, ultrastructural changes of the myocardium, morphological structure, wall thickness and vessels	Group 1: Increase in the relative volume of the endothelium in the myocardium as indicated by CD34 accumulation.<br>Group 2: Compensatory restructuring of the energy apparatus of the contractile cardiomyocytes of the left ventricle and increase of CD34.<br>Group 4: Destruction of subsarcolemal and paranuclear organelles, edema and degradation of intermyofibrillar mitochondria; development of destructive-degenerative processes in the mitochondrial apparatus of left ventricular cardiomyocytes, deepening of damage to intermitochondrial contacts. CD34 decreased.<br>(No statistics provided)<br><br>Conclusion: Morphogenetic transformations were highly active in the myocardium of the heart at exposures of 45 minutes (both groups) resulting in a reparative-regenerative effect, whereas after 120 min exposure (+ thyroidectomized) an increase in degenerative and destructive processes in the myocardium of the heart wall was observed.<br><br>(Effect found)	Data probably also published in parts by Drobakhin (2020)

Authors, study design	Study groups	Exposure (Assessment method)	Endpoints	Results<br>Conclusion according to authors<br>(Effect categorization according to authors)	Remarks
**Heart & CVS**					
Yin et al. 2021<br><br>EA<br><br>Study quality: 3rd tier	Wistar rats<br><br>Exposure group 1: n=20,<br>exposure group 2: n=20,<br>control group: n=20	Exposure source not provided, 9.375 GHz<br><br>3 conditions: control group and 2 exposure groups (PD 5 mW/cm² and 10 mW/cm²)<br>(no data provided on exposure validation)<br><br>Whole body exposure<br><br>Exposure duration: 6 min<br><br>Remarks: Rats were restrained with rats rotating during exposure	Effects on structure and function of the heart: effects on structure of myocardial tissue (on day 7 after exposure; 5 rats/group); effects on ultrastructure of myocardial tissue (on day 7 after exposure; 3 rats/group); ECG (at 6 h, on days 7, 14, and 28 after exposure; 5 rats/group)<br><br>Protein expression and enzyme activities in the heart: connexin43 expression and distribution (on day 7 after exposure; 5 rats/group), aspartate transferase, creatine kinase, lactate dehydrogenase (at 6 h, on days 7, 14, and 28 after exposure; 5 rats/group)	At 6 h after exposure the heart rate increased (both exposure groups) and on day 7 after exposure the P wave amplitude decreased (5 mW/cm² group) and the R wave amplitude increased (both groups); at 6 h after exposure myocardial enzyme activities of aspartate transferase and creatine kinase increased in 10 mW/cm² group and enzyme activity of , lactate dehydrogenase increased in 5 mW/cm² group; , lactate dehydrogenase was also increased on days 7 and 14 after exposure in 10 mW/cm² group; ultrastructure of cardiac tissue was damaged in 10 mW/cm² group, e.g., mitochondrial cavitation occurred and intercalary disk structure was broken (no statistics provided). The expression of connexin43 in myocardial tissue was decreased in 10 mW/cm² group.<br><br>Conclusion: The mechanism of abnormal electrical conduction in the heart of exposed rats might be related to the decreased expression of connexin43.<br><br>(Effect found)	Data on 2.856 GHz exposure and co-exposure not relevant and therefore not extracted.

Authors, study design	Study groups	Exposure (Assessment method)	Endpoints	Results Conclusion according to authors (Effect categorization according to authors)	Remarks
**Effects on skin**					
Gostyu-khina et al. 2022<br><br>EA<br><br>Study quality: 3rd tier	Wistar rats<br><br>Exposure group 1: n=10,<br>exposure group 2: n=10,<br>cage control: n=10	Horn antenna, 10.0 GHz, PW (width 100 ns, rate 8 pps)<br><br>3 conditions: cage control and 2 exposure groups: group 1 (PD 140 W/cm² (peak)), group 2 (1500 W/cm² (peak)) (measurement, calibration)<br><br>Exposure of burn wound on the back<br><br>Exposure duration: 8 min per day (4000 pulses per session) for 4 days after formation of wound<br><br>Remarks: animal's body (except the wound) was covered with a radio-absorbing material during exposure; temperature of wound area increased by 0.03–0.05°C (140 W/cm²) and 0.13°C (1500 W/cm²) during exposure	Regeneration of burn wounds: measurement of damaged skin area (on days 1, 5, 12, 14, 19, 24 and 28 after formation of wound), inflammatory process of burn wounds (hematological analysis; on day 1, 5, 12 and 19 after formation of wound)	From the 19th day of the experiment, a significant decrease in the wound area was found in exposed groups with complete healing of all burns at the 28th (140 W/cm²) or 30th (1500 W/cm²) day of the experiment. 140 W/cm² exposure was more effective than 1500 W/cm² exposure.<br><br>Conclusion: Exposure of burn wounds to nanosecond repetitive pulsed microwave radiation contributed to acceleration of their regeneration.<br><br>(Effect found)	Thermal wound was created by applying a metal rod (diameter 2 cm) heated up to 100°C

Authors, study design	Study groups	Exposure (Assessment method)	Endpoints	Results Conclusion according to authors (Effect categorization according to authors)	Remarks
**Effects on skin**					
Sheikh et al. 2013<br><br>EA<br><br>Study quality: 3rd tier	db/db mouse (model of diabetic wound healing)<br><br>Exposure group: n=5, control group: n=5	2 antennae, 7.5 GHz<br><br>2 conditions: control and exposure (electric field strength 200 V/m) (no data provided on exposure validation)<br><br>Exposure of wound on the back<br><br>Exposure duration: 1 h/day for 7 days<br><br>Remarks: 2 antennae approx. 5 mm away from the wounds; animals anaesthetized during exposure	Diabetic wound healing: vascular endothelial growth factor (VEGF) expression in the wounds	Exposure resulted in increased VEGF protein levels in wound tissue.<br><br>Conclusion: VEGF accumulation in the wound may result in increased wound vascularization and improved healing.<br><br>(Effect found)	Additional data on in vitro experiments (including 60 Hz exposure) not relevant for this review and therefore not extracted.<br><br>Extracted *in vivo* experiments were preliminary data used to validate the *in vitro* experiments in the current study.

Authors, study design	Study groups	Exposure (Assessment method)	Endpoints	Results Conclusion according to authors (Effect categorization according to authors)	Remarks
**Multiple parameters & other parameters**					
Cain and Rissmann 1978<br><br>EA, HCT<br><br>Study quality: 3rd tier	cat, dog, chinchilla, healthy humans (male and female)<br><br>Cats<br>exposure group 1: n=1 (pre-experiment; alive and dead (as control)), exposure group 2: n=2<br><br>Beagle dog<br>exposure group: n=1<br><br>Chinchillas<br>exposure group: n=2<br><br>Humans<br>exposure group: n=8	Horn antenna, 3.0 GHz, PW (rate 0.5 Hz, width 5, 10 or 15 µs)<br><br>Several exposure conditions: combination of different peak power densities and pulse durations (no further details provided) (measurement, calculation/simulation)<br><br>Head exposure<br><br>Exposure duration: approx. 50 x 10 µs wide pulses (pre-experiment); approx. 100 x 5, 10 or 15 µs wide pulses each (main experiment)<br><br>Remarks: animals: spinal axis parallel to the electric field; humans: faced away from the antenna	Effects on auditory system: determination of hearing threshold parameter (auditory evoked response) (audiometric testing (for humans); electrical recording via scalp electrodes (for non-human species) or implanted glass micropipettes (for the cats in the pre-experiment)	The energy density per pulse needed to evoke auditory responses in cats ranged from 8.7 µJ/cm² to 14 µJ/cm². Chinchilla thresholds ranged from 7.5 µJ/cm² to 20 µJ/cm². The average of the thresholds for the beagle dog was 5 µJ/cm². The average threshold which could be heard by human subjects was 10.6 µJ/cm² (3.4-17.5 µJ/cm²). However, 3 out of the 8 subjects could not hear pulses below 20 µs at maximum peak power settings.<br><br>Conclusion: For the pulse widths investigated, the threshold energy density per pulse for the animals remained relatively constant as pulse width and peak power were varied. The inability of some of the human subjects to hear short microwave pulses was correlated with hearing losses above 8 kHz.<br><br>(Effect found)	Additional experiments performed with acoustic stimuli (for comparison), but data not extracted for the current review

Authors, study design	Study groups	Exposure (Assessment method)	Endpoints	Results Conclusion according to authors (Effect categorization according to authors)	Remarks
**Multiple parameters & other parameters**					
Deich-mann et al. 1959<br><br>EA<br><br>Study quality: 3rd tier	Albino Sprague-Dawley rats and mice (Swiss and C57)<br><br><u>Rats</u><br>n=2 to 11 (at least 24 different conditions)<br><br><u>Mice</u><br>n=10 (at least 5 different conditions)<br><br>Probably further conditions tested but not transparently presented	Horn antenna, 24.0 GHz, PW (duty cycle 0.0006)<br><br><u>Rats</u><br>12 whole-body conditions (PD 0.02 - 0.15 W/cm² and near-zone), head exposure (0.26 W/cm²), dorsal surface of the head (0.019 - 0.3 W/cm²), lumbar exposure (0.26 W/cm²), scrotal exposure (0.02 - 0.3 W/cm²), right side exposure (0.075, 0.08 W/cm²)<br><br><u>Mice</u><br>5 whole-body conditions (0.037 - 0.17 W/cm²)<br><br>(measurement, calculation/simulation)<br><br>Exposure of whole body, head, or lumbar/abdominal/scrotal region (in dependence of the experiment)<br><br>Exposure duration: single continuous exposure (rats up to 480 min (minimum), mice up to 282/140 min (mean/minimum))<br><br>Remarks: animals were anesthetized or restrained in Plexiglas cages and exposed individually	Local and systemic effects: rectal, subcutaneous, and internal organ temperatures; behavior; effects on skin (e.g., burns) and other organs (e.g., lung, muscle, liver, spleen, kidney, hemopoietic system (e.g., number of erythrocytes, leukocytes, and hemoglobin content)	5 min. of exposure (rat) to 0.02 W/cm² induced slight congestion of the lungs, a subcutaneous scrotal tissue temperature of 35°C, and a rise in temperature of 1°C at the dura of the frontal lobe of an intact skull.<br>7 h of exposure (rat) to 0.024 W/cm² caused a marked leukocytopenia and an increase in the number of erythrocytes. The severity of pathological changes decreased with decreasing power density. (Most data presented in descriptive manner in the text only without statistics)<br><br>Conclusion: Acute exposure to microwave is capable of inducing marked local and systemic effects in rats and mice.<br><br>(Effect found)	Data on infrared exposure, on exposure of chicks, on intermittent exposure (lack of comparison group) and on lethality/survival (temperature increase >42°C) not relevant for this review and therefore not extracted

Authors, study design	Study groups	Exposure (Assessment method)	Endpoints	Results Conclusion according to authors (Effect categorization according to authors)	Remarks
**Multiple parameters & other parameters**					
Fontani et al. 2011<br><br>HCT<br><br>Study quality: 3rd tier	Patients (taking pain medication) with pain and physical problems and medically unexplained symptoms, male and female<br><br>Exposure group: n=688 (age 41.8 years (mean))<br><br>control group: n=200, (age 47.7 years (mean))	Radioelectric asymmetric conveyer (REAC) (medical device), 10.5 GHz, PW (7 pulses of 500 ms)<br><br>2 conditions: control group and exposure (SAR 7mW/kg)<br>(no data provided on exposure validation)<br><br>Whole body exposure<br><br>Exposure duration: 18 sessions on alternate days; 7 pulses of 500 ms<br><br>Remarks: exposure applied by touching the metallic tip of the REAC probe onto seven specific reflex auricular points	Symptoms of pain, physical problems, and psychological stress (Psychological Stress Measure test)	There was a significant reduction in scores measuring subjective perceptions of stress for REAC-treated patients and the number of treated subjects reporting symptoms of stress-related pain and physical problems was significantly decreased.<br><br>Conclusion: REAC treatment appeared to reduce subjective perceptions of stress, particularly on the pain and physical problems cluster.<br><br>(Effect found)	

**Authors, study design**	**Study groups**	**Exposure (Assessment method)**	**Endpoints**	**Results<br>Conclusion according to authors<br>(Effect categorization according to authors)**	**Remarks**
**Multiple parameters & other parameters**					
Mikołaj-czyk et al. 1991<br><br>EA<br><br>Study quality: 3rd tier	IMP-Dak rats<br><br>Exposure group: n=11, sham exposure group: n=11	Horn antenna (maritime radar system), 9.5 GHz, PW (width 0.5 µs, rate 1000 pps)<br><br>2 conditions: sham exposure and exposure (SAR 10.5 W/kg) (measurement, calculation/simulation)<br><br>Whole body exposure<br><br>Exposure duration: 30 min<br><br>Remarks: unrestrained rats were exposed in pairs	Effects on thermoregulation (colonic temperature)<br><br>Organ weight, conductivity, and water content (lobe of the submaxillary salivary gland, brain cortex, peripheral blood, and left kidney)	Exposed rats showed a temperature rise of 2.65°C, a significant weight augmentation of the submaxillary salivary gland accompanied by a large increase in conductivity and water content. Additionally, a decreased conductivity was found in the kidney of exposed rats.<br><br>Conclusion: There were some differences in conductivity and water content in the submaxillary salivary gland and kidney in exposed rats.<br><br>(Effect found)	Additional data on 27.12 MHz, 2880 MHz, or heat (40°C) exposure not relevant for this review and therefore not extracted; conditions of all exposures were predetermined to cause an increase in colonic temperature of 2-2.5°C within 30 min
Moussa 2009<br><br>EA<br><br>Study quality: 3rd tier	Swiss albino or Wister (Wistar?) albino rats (contradictory data)<br><br>Exposure group 1-3: n=10,<br>control group: n=10	Wave guide, 3.5 GHz or 10 GHz (contradictory data), modulated (no further data provided)<br><br>4 conditions: control group and 3 exposure groups (SAR 1.0 W/kg (whole body): group 1 (exposure for 4 weeks), group 2 (8 weeks), group 3 (8 weeks + 50 days post exposure observation)<br>(measurement, calculation/simulation)<br><br>Whole body exposure<br><br>Exposure duration: 4 or 8 weeks	Effects on liver and kidney (levels of alanine aminotransferase, aspartate aminotransferase, alkaline phosphatase, albumin, plasma creatinine, urea and total protein)<br><br>Oxidative stress parameters (levels of malondialdehyde and reduced glutathione)<br><br>Effects on hemoglobin (electrical conductivity and intrinsic viscosity)	All investigated liver enzymes and kidney parameters were increased compared with the control (according to table 1, only in the two 8 weeks-exposure groups, contradictory results). Malondialdehyde increased while glutathione concentration decreased. The electrical conductivity and intrinsic viscosity of hemoglobin were increased.<br><br>Conclusion: The exposure affected the oxidative state of the liver, kidney, and hemoglobin.<br><br>(Effect found)	

Authors, study design	Study groups	Exposure (Assessment method)	Endpoints	Results Conclusion according to authors (Effect categorization according to authors)	Remarks
**Multiple parameters & other parameters**					
Prausnitz and Süsskind 1962<br><br>EA<br><br>Study quality: 3rd tier	Swiss albino mice<br><br>Exposure group: n=200,<br>sham exposure group: n=100	Horn antenna, 9.27 GHz, PW (width 2 µs, rate 500 pps)<br><br>2 conditions: sham exposure and exposure (PD 0.1 W/cm²) (measurement, calculation/simulation, calibration)<br><br>Whole body exposure<br><br>Exposure duration: 4.5 min (half of LD50 determined in pre-tests) per day, 5 days/week for 59 weeks<br><br>Remarks: 10 animals exposed simultaneously in a circular, compartmentalized cage which rotated at 1 rpm; exposure applied from below; exposure produced an average body temperature rise of 3.3°C (determined in 5 mice)	Mortality<br><br>Histopathology of different organs (e.g., liver, spleen, kidneys, lungs, testes; 10/5, 20/10, 67/19 exposed/sham exposed animals at 7, 16 or 19 months, resp.)<br><br>Effects on red and white blood counts (in 10% of the animals after 4 months of exposure)<br><br>Glucose level in urine<br><br>Effects on body weight	Testicular degeneration was found in 40% of the dead exposed mice and in 8% of the controls. Cancer of white cells was found in 35% of exposed mice and in 10% of controls. In third series animals, 21% of exposed mice and 5% of controls showed testicular damage (some degree of atrophy of tubules with corresponding diminution in spermatogenesis) and 38% of exposed mouse testes weighed less than 90 mg, whereas this was true of only 5% of controls. (No statistics applied)<br><br>Conclusion: Testicular degenerations were found in exposed mice that died without being sacrificed. Testicular damage was strongly suggested in the final sacrifice series.<br><br>(Effect found)	3 sacrifice series were carried out 7, 16, and 19 months after the start of exposure (second series performed within 1 month and third series was performed 4 months after final exposure).

Authors, study design	Study groups	Exposure (Assessment method)	Endpoints	Results Conclusion according to authors (Effect categorization according to authors)	Remarks
**Multiple parameters & other parameters**					
Ray and Behari 1990<br><br>EA<br><br>Study quality: 3rd tier	Albino rats<br><br>Exposure group: n=18, sham exposure group: n=18	Horn antenna, 7.5 GHz, PW (1.0 kHz square wave modulation, width 0.6 ms)<br><br>2 conditions: sham exposure and exposure (PD 0.6 mW/cm², SAR 0.0317 W/kg (average)) (measurement, calculation/simulation, calibration)<br><br>Whole body exposure<br><br>Exposure duration: 3 h/day for 60 days<br><br>Remarks: 9 rats were exposed simultaneously, each in a prespecified cage compartment	Consumption of food and water (daily), body weight (every 10 days)<br><br>Hematologic parameters: hemoglobin level, erythrocyte sedimentation rate, count of red blood cells, white blood cells and differential counts of neutrophils, eosinophils, lymphocytes, and monocytes (after exposure)<br><br>Weights of lungs, heart, spleen, testis, kidney, liver, skull, brain, ovary (after exposure)	Exposed animals tended to eat and drink less and thus showed a smaller gain in body weight (for body weight statistical significance not shown). White blood cell and eosinophil count was increased, while the weights of spleen, kidney, brain, and ovary were reduced in exposed animals.<br><br>Conclusion: A nonspecific stress response due to microwave exposure may be responsible for the observed physiological changes.<br><br>(Effect found)	

Authors, study design	Study groups	Exposure (Assessment method)	Endpoints	Results Conclusion according to authors (Effect categorization according to authors)	Remarks
**Multiple parameters & other parameters**					
Rifat and Sisodia 2017<br><br>EA<br><br>Study quality: 3rd tier	Swiss albino mice<br><br>Exposure group: n=10, sham exposure group: n=10	Horn antenna, 10.0 GHz, PW<br><br>2 conditions: sham exposure and exposure (PD 0.25 mW/cm² (max), SAR 0.179 W/kg) (measurement, calculation/simulation)<br><br>Whole body exposure<br><br>Exposure duration: 2 h/day for 30 days<br><br>Remarks: 2 mice exposed simultaneously; lightly restrained	Oxidative stress parameters in intestine, liver, spleen, testes (lipid peroxidation (malondialdehyde level), glutathione and protein levels)<br><br>Body weight	Lipid peroxidation was increased, and glutathione and protein levels were decreased in all organs investigated after exposure.<br><br>Conclusion: Exposure to microwaves caused significant alterations in lipid peroxidation, glutathione, and protein levels of different organs of the mice.<br><br>(Effect found)	Data on co-exposure group (microwaves and plant extract) not extracted.
