# Supplementary material for: Physiological and health-related effects of 5G-relevant radiofrequency fields: A systematic review of experimental studies in humans and mammals
Source: PLoS One. 2026 Nov 4;21(11):e0361104. doi: 10.1371/journal.pone.0361104 (PMC13734471; doi:10.1371/journal.pone.0361104)
Supplement: Supplementary file 17 [file pone.0361104.s017.pdf]

Peer-Review Report for Manuscript PONE-D-25-16037

Title: Physiological and health-related effects of 5G-relevant radiofrequency fields: A systematic review of experimental studies in humans and mammals"

Overall Evaluation

The document presents a thorough and well-planned review of studies on 5Grelevant RFEMF (3–30 GHz) in humans and mammals. The topic is relevant to current events, important for society, and significant in science. The authors follow PRISMA guidelines, register their research protocol, and correctly use the OHAT risk of bias tools.

However, some areas need more explanation, strengthening, or changes to make them clearer, more reproducible, and easier to understand. The main limitations concern how we handle heterogeneity, the decisions we make about evidence synthesis, the clarity of the presentations, and how we interpret the findings.

1. How would your conclusions change if exposure classification relative to ICNIRP limits were removed?

2. Can you provide a more detailed rationale for excluding cross-sectional studies?

3. Could you summarize the main methodological weaknesses in a single table?

3. What are the most realistic exposure scenarios for humans at 3–30 GHz, and how do they compare to the experimental conditions?

5. Please prepare a clean, journal-ready reviewer form, a point-by-point revision plan, or a rewritten conclusion section based on the manuscript.

Major Comments

1. Evidence synthesis criteria need clearer justification

The manuscript frequently states that evidence synthesis was not possible due to heterogeneity, small numbers, or risk of bias. However, the decision rules for when synthesis is or is not performed are scattered and not consistently justified.

Provide a clear, unified decision framework early in the Methods section.

Consider adding a flowchart or table summarizing synthesis criteria.

2. Interpretation of "inadequate evidence" should be more nuanced

The conclusion states that current scientific data are "inadequate to draw any conclusions." However, the review *does* identify:

Moderate evidence for cytokine secretion effects

Low evidence for hippocampal structural changes

Consistent thermal effects at high exposures

The conclusion should distinguish between:

No evidence

Insufficient evidence

Evidence of no effect

Evidence of possible effect

This distinction is important for public health communication.

3. Exposure classification relative to ICNIRP limits is uncertain

The authors acknowledge that applying human exposure limits to animals is problematic. However, the manuscript still draws conclusions based on "above/below limit values."

Provide a stronger methodological justification or

Add a sensitivity analysis showing how results change if classification is removed.

4. Lack of epidemiological studies deserves deeper discussion

The review found zero epidemiological studies meeting criteria. This is a major finding but is under-discussed.

Explain why epidemiological evidence is absent.

Discuss feasibility challenges (exposure assessment, confounding, latency).

Clarify implications for future research.

5. Risk-of-bias results need clearer visualization

The text describes risk-of-bias patterns, but readers would benefit from:

A summary figure (e.g., heatmap or bar chart) showing domain-level ratings.

A table highlighting the most common methodological weaknesses.

6. Some sections are overly long and descriptive

The Results section contains extensive study-by-study descriptions.

Consider condensing these into tables and focusing the text on patterns, not details.

Move long descriptions to Supplementary Data.

Minor Comments

1. Clarify terminology

Terms like “effect,” “no effect,” “adverse,” and “beneficial” are used inconsistently. Define these terms explicitly.

2. Improve consistency in reporting exposure metrics

Some studies report SAR, others PD, others APD. Provide a standardized conversion table or explain why harmonization is not possible.

3. Add a PRISMA flow diagram figure number

The text references “Fig. 1” but the figure is not visible in the excerpt. Ensure it is included.

4. Improve readability of long paragraphs

Several paragraphs exceed one page in length. Breaking them up will improve clarity.

5. Check for minor typographical issues

A few small inconsistencies appear (spacing, capitalization, missing commas).

4. Strengths of the Manuscript

Extensive literature search across PubMed, Web of Science, and EMF-Portal.

Protocol registration (PROSPERO) ensures transparency.

Use of OHAT risk-of-bias tool, appropriate for toxicology-related evidence.

Clear identification of research gaps, especially for high-frequency 5G bands.

Balanced tone, avoiding alarmism or unwarranted reassurance.

5. Recommendation

Major Revision

The manuscript is strong and valuable, but the issues above—especially regarding evidence synthesis criteria, interpretation, and clarity—should be addressed before publication.
