# Supplementary material for: Physiological and health-related effects of 5G-relevant radiofrequency fields: A systematic review of experimental studies in humans and mammals
Source: PLoS One. 2026 Nov 4;21(11):e0361104. doi: 10.1371/journal.pone.0361104 (PMC13734471; doi:10.1371/journal.pone.0361104)
Supplement: Supplementary file 19 [file pone.0361104.s020.docx]

We thank the editor and reviewers for their time to evaluate our revised manuscript and that they rated it as potentially acceptable for publication. We respond separately to each point raised by the reviewer.

**Academic editor comment:**

**The manuscript is greatly improved, but I would suggest you to address the minor point raised by Reviewer 3 before having it accepted for publication.**

We would like to express our appreciation to the editor for this overall assessment and are delighted to address the minor point raised by Reviewer#3 below.

Please note that we have corrected one of the references [24] from Qin et al. **2022** to Qin et al. **2024**. We have therefore uploaded the updated version of each of the following files as well:

- Fig2.tif
- S10_Tables
- S12_Exposure
- S16_Reference_List

**Comments Reviewer #2:**

**The authors have addressed all major and minor concerns. Specifically, the clear justification for evidence synthesis criteria, improved visualization of risk-of-bias data and the discussion regarding the absence of epidemiological studies now appropriate and well-reasoned.**

We would like to thank the reviewer for taking the time to review our revision, and for his overall assessment.

**Comments Reviewer #3:**

**I was not one of the original reviewers, but I have examined their reviews and consider that the authors have adequately responded. This is an experienced group in SRs in the health effects of EMF and the paper is very good.**

Thank you for this positive feedback, which is much appreciated.

**One suggestion: put in a statement that few if any of the papers used actual 5G waveforms (which use a particular form of pulse modulation). I have seen numerous misstatements by authors that they were investigating "effects of 5G" whereas they were using CW of GSM modulated energy for exposure.**

The reviewer is right. In some cases, study authors claim to have investigated the “effects of 5G”, but this was not actually the case (e.g., [117-119]). We now provide detailed commentary on this issue, e.g., lines 738-742 (chapter “Newly published articles since the last systematic search”):

*17 of the 28 relevant articles, including all human studies, explicitly stated that they investigated 5G exposure. However, not all of them used a 5G modulation scheme for the applied RF signal, e.g., three studies conducted by the same research group investigated GSM-modulated 3.5 GHz exposure, which is not representative of 5G exposure [117–119].*

Additionally, we have modified the section of the 'Review Limitations' chapter to clarify that only a small proportion of the studies included in our systematic review explicitly involved 5G exposure.

Lines 702-707

*Only five of the 50 studies included in the evidence synthesis explicitly stated that they were investigating 5G exposure [24,50,60,61,98]. Although the RF signals used in these studies were at frequencies used in 5G networks, they did not represent typical 5G modulation patterns but were either continuous wave or (GSM-like) pulse modulated. The remaining studies either used therapeutic applications, high-power microwaves, radar exposures or investigated the effects of microwave exposure without focusing on any specific application. Therefore, it is important to note that the (…)*
