# Supplementary material for: Physiological and health-related effects of 5G-relevant radiofrequency fields: A systematic review of experimental studies in humans and mammals
Source: PLoS One. 2026 Nov 4;21(11):e0361104. doi: 10.1371/journal.pone.0361104 (PMC13734471; doi:10.1371/journal.pone.0361104)
Supplement: Peer review history

# Peer review: Decision Letter 0

**DOI:** 10.1371/journal.pone.0361104.r001

**Authors:** Hesham Zakaly

9 Mar 2026

Dear Dr. Driessen,

Thank you for submitting your manuscript to PLOS ONE. After careful consideration, we feel that it has merit but does not fully meet PLOS ONE’s publication criteria as it currently stands. Therefore, we invite you to submit a revised version of the manuscript that addresses the points raised during the review process.

Please submit your revised manuscript by Apr 23 2026 11:59PM. If you will need more time than this to complete your revisions, please reply to this message or contact the journal office at plosone@plos.org. When you're ready to submit your revision, log on to https://www.editorialmanager.com/pone/ and select the 'Submissions Needing Revision' folder to locate your manuscript file.

- A letter that responds to each point raised by the academic editor and reviewer(s). You should upload this letter as a separate file labeled 'Response to Reviewers'.
- A marked-up copy of your manuscript that highlights changes made to the original version. You should upload this as a separate file labeled 'Revised Manuscript with Track Changes'.
- An unmarked version of your revised paper without tracked changes. You should upload this as a separate file labeled 'Manuscript'.

If you would like to make changes to your financial disclosure, please include your updated statement in your cover letter. Guidelines for resubmitting your figure files are available below the reviewer comments at the end of this letter.

If applicable, we recommend that you deposit your laboratory protocols in protocols.io to enhance the reproducibility of your results. Protocols.io assigns your protocol its own identifier (DOI) so that it can be cited independently in the future. For instructions see: https://journals.plos.org/plosone/s/submission-guidelines#loc-laboratory-protocols. Additionally, PLOS ONE offers an option for publishing peer-reviewed Lab Protocol articles, which describe protocols hosted on protocols.io. Read more information on sharing protocols at https://plos.org/protocols?utm_medium=editorial-email&utm_source=authorletters&utm_campaign=protocols.

We look forward to receiving your revised manuscript.

Kind regards,

Hesham M.H. Zakaly, Ph.D.

Academic Editor

PLOS One

**Journal Requirements:**

When submitting your revision, we need you to address these additional requirements.

1. Please ensure that your manuscript meets PLOS ONE's style requirements, including those for file naming. The PLOS ONE style templates can be found at

https://journals.plos.org/plosone/s/file?id=wjVg/PLOSOne_formatting_sample_main_body.pdf and

https://journals.plos.org/plosone/s/file?id=ba62/PLOSOne_formatting_sample_title_authors_affiliations.pdf

2. Please include your tables as part of your main manuscript and remove the individual files. Please note that supplementary tables (should remain/ be uploaded) as separate "supporting information" files.

3. Thank you for stating the following in the Financial Disclosure section:

“LB, DD, and TE received funding by the Ministry of Economic Affairs, Industry, Climate Action and Energy of the State of North Rhine-Westphalia, Germany, in the framework of the 5G.NRW-Förderwettbewerb (https://5g.nrw/best-practices/foerderwettbewerb5g-nrw/). The funder had no role in the study design, data collection and analysis, decision to publish, or preparation of the manuscript.”

We note that one or more of the authors are employed by a commercial company: Electromagnetic Compatibility & Optics, Seibersdorf Laboratories

1. Please provide an amended Funding Statement declaring this commercial affiliation, as well as a statement regarding the Role of Funders in your study. If the funding organization did not play a role in the study design, data collection and analysis, decision to publish, or preparation of the manuscript and only provided financial support in the form of authors' salaries and/or research materials, please review your statements relating to the author contributions, and ensure you have specifically and accurately indicated the role(s) that these authors had in your study. You can update author roles in the Author Contributions section of the online submission form.

Please also include the following statement within your amended Funding Statement.

“The funder provided support in the form of salaries for authors [insert relevant initials], but did not have any additional role in the study design, data collection and analysis, decision to publish, or preparation of the manuscript. The specific roles of these authors are articulated in the ‘author contributions’ section.”

If your commercial affiliation did play a role in your study, please state and explain this role within your updated Funding Statement.

2. Please also provide an updated Competing Interests Statement declaring this commercial affiliation along with any other relevant declarations relating to employment, consultancy, patents, products in development, or marketed products, etc.

Within your Competing Interests Statement, please confirm that this commercial affiliation does not alter your adherence to all PLOS ONE policies on sharing data and materials by including the following statement: "This does not alter our adherence to  PLOS ONE policies on sharing data and materials.” (as detailed online in our guide for authors http://journals.plos.org/plosone/s/competing-interests) . If this adherence statement is not accurate and  there are restrictions on sharing of data and/or materials, please state these. Please note that we cannot proceed with consideration of your article until this information has been declared.

Please include both an updated Funding Statement and Competing Interests Statement in your cover letter. We will change the online submission form on your behalf.

4. Please include your tables as part of your main manuscript and remove the individual files. Please note that supplementary tables (should remain/ be uploaded) as separate "supporting information" files.

5. Please include captions for your Supporting Information files at the end of your manuscript, and update any in-text citations to match accordingly. Please see our Supporting Information guidelines for more information: http://journals.plos.org/plosone/s/supporting-information.

6. If the reviewer comments include a recommendation to cite specific previously published works, please review and evaluate these publications to determine whether they are relevant and should be cited. There is no requirement to cite these works unless the editor has indicated otherwise.

[Note: HTML markup is below. Please do not edit.]

Reviewers' comments:

Reviewer's Responses to Questions

**Comments to the Author**

1. Is the manuscript technically sound, and do the data support the conclusions?

Reviewer #1: Partly

Reviewer #2: Yes

**********

2. Has the statistical analysis been performed appropriately and rigorously?

Reviewer #1: N/A

Reviewer #2: Yes

**********

3. Have the authors made all data underlying the findings in their manuscript fully available?

The PLOS Data policy

Reviewer #1: Yes

Reviewer #2: Yes

**********

4. Is the manuscript presented in an intelligible fashion and written in standard English?

Reviewer #1: Yes

Reviewer #2: Yes

**********

Reviewer #1: 1:

The review is not up to date on the recent literature. For example, on:

Bodin R, Seewooruttun C, Corona A, Delanaud S, Pelletier A, Villégier AS. Sex-dependent impact of perinatal 5G electromagnetic field exposure in the adolescent rat behavior. Environ Sci Pollut Res Int. 2023 Nov;30(53):113704-113717.

2:

In the Background “covid” seems to extrapolate beyond the topic while, more accuracy regarding the actual environmental exposures of the population with 5G frequencies is necessary. It is worth mentioning which frequencies are introduced in our public or occupancy environments and which are not introduced yet.

3:

The methods aimed first to select 5G and non5G EMF research. What was the limitation for not directly focusing on 5G frequencies with appropriate key words ?

4:

EMF effects may be linked to absorbed energy levels. It seems necessary to further mention and more deeply assess the exposure levels (specific absorption rate or surface power density, …) and durations (acute, chronical, intermittent, in min or hours, …) for each study, as well as considering the age of the rodent models.

5:

Minor point: “obeserved”

“In five studies, effects were obeserved at exposures above and below the limits”

Reviewer #2: Overall Evaluation.

The document presents a thorough and well-planned review of studies on 5Grelevant RFEMF (3–

30 GHz) in humans and mammals. The topic is relevant to current events, important for society,

and significant in science. The authors follow PRISMA guidelines, register their research protocol,

and correctly use the OHAT risk of bias tools.

However, some areas need more explanation, strengthening, or changes to make them clearer,

more reproducible, and easier to understand. The main limitations concern how we handle

heterogeneity, the decisions we make about evidence synthesis, the clarity of the presentations,

and how we interpret the findings.

1. How would your conclusions change if exposure classification relative to ICNIRP limits were

removed?

2. Can you provide a more detailed rationale for excluding cross-sectional studies?

3. Could you summarize the main methodological weaknesses in a single table?

3. What are the most realistic exposure scenarios for humans at 3–30 GHz, and how do they

compare to the experimental conditions?

5. Please prepare a clean, journal-ready reviewer form, a point-by-point revision plan, or a

rewritten conclusion section based on the manuscript.

For details please see the attached PDF file......

**********

what does this mean?). If published, this will include your full peer review and any attached files.

If you choose “no”, your identity will remain anonymous but your review may still be made public.

**Do you want your identity to be public for this peer review?**  For information about this choice, including consent withdrawal, please see our Privacy Policy

Reviewer #1: No

Reviewer #2: **Yes:**  Md. Ariful Islam Arif

**********

[NOTE: If reviewer comments were submitted as an attachment file, they will be attached to this email and accessible via the submission site. Please log into your account, locate the manuscript record, and check for the action link "View Attachments". If this link does not appear, there are no attachment files.]

To ensure your figures meet our technical requirements, please review our figure guidelines: https://journals.plos.org/plosone/s/figures

You may also use PLOS’s free figure tool, NAAS, to help you prepare publication quality figures: https://journals.plos.org/plosone/s/figures#loc-tools-for-figure-preparation.

NAAS will assess whether your figures meet our technical requirements by comparing each figure against our figure specifications.

Attachment Submitted filename: Reviewers Comments.pdf

# Author comment: Author response to Decision Letter 1

**DOI:** 10.1371/journal.pone.0361104.r002

24 Apr 2026

Please see Response to Reviewers.docx

We thank the editor and reviewers for their critical and valuable comments and recommendations. We responded separately to each point and all page and line references in the manuscript refer to the manuscript without tracked changes if not mentioned otherwise.

Journal Requirements/Academic editor:

When submitting your revision, we need you to address these additional requirements.

1. Please ensure that your manuscript meets PLOS ONE's style requirements, including those for file naming. The PLOS ONE style templates can be found at

https://journals.plos.org/plosone/s/file?id=wjVg/PLOSOne_formatting_sample_main_body.pdf and

https://journals.plos.org/plosone/s/file?id=ba62/PLOSOne_formatting_sample_title_authors_affiliations.pdf

We thank the editor for this information. We applied the PLOS ONE style requirements accordingly.

2. Please include your tables as part of your main manuscript and remove the individual files. Please note that supplementary tables (should remain/ be uploaded) as separate "supporting information" files.

Done.

3. Thank you for stating the following in the Financial Disclosure section:

“LB, DD, and TE received funding by the Ministry of Economic Affairs, Industry, Climate Action and Energy of the State of North Rhine-Westphalia, Germany, in the framework of the 5G.NRW-Förderwettbewerb (https://5g.nrw/best-practices/foerderwettbewerb5g-nrw/). The funder had no role in the study design, data collection and analysis, decision to publish, or preparation of the manuscript.”

We note that one or more of the authors are employed by a commercial company: Electromagnetic Compatibility & Optics, Seibersdorf Laboratories

1. Please provide an amended Funding Statement declaring this commercial affiliation, as well as a statement regarding the Role of Funders in your study. If the funding organization did not play a role in the study design, data collection and analysis, decision to publish, or preparation of the manuscript and only provided financial support in the form of authors' salaries and/or research materials, please review your statements relating to the author contributions, and ensure you have specifically and accurately indicated the role(s) that these authors had in your study. You can update author roles in the Author Contributions section of the online submission form.

Please also include the following statement within your amended Funding Statement.

“The funder provided support in the form of salaries for authors [insert relevant initials], but did not have any additional role in the study design, data collection and analysis, decision to publish, or preparation of the manuscript. The specific roles of these authors are articulated in the ‘author contributions’ section.”

If your commercial affiliation did play a role in your study, please state and explain this role within your updated Funding Statement.

Thank you for pointing out that the financial disclosure section was not sufficiently outlined for two of the authors (GS and PS). We have now amended the following Funding Statement for these authors:

PS and GS are employees of Seibersdorf Laboratories, Department EMC & Photonics, a commercial company. Seibersdorf Laboratories provided support in the form of salaries for PS and GS, but did not have any additional role in the study design, data collection and analysis, decision to publish, or preparation of the manuscript. The specific roles of these authors are articulated in the ‘author contributions’ section.

Additionally, we have amended the funding statement for the other authors in line with your recommendation.

“The funder provided support in the form of salaries for authors [LB, DD, and TE], but did not have any additional role in the study design, data collection and analysis, decision to publish, or preparation of the manuscript. The specific roles of these authors are articulated in the ‘author contributions’ section.”

2. Please also provide an updated Competing Interests Statement declaring this commercial affiliation along with any other relevant declarations relating to employment, consultancy, patents, products in development, or marketed products, etc.

Within your Competing Interests Statement, please confirm that this commercial affiliation does not alter your adherence to all PLOS ONE policies on sharing data and materials by including the following statement: "This does not alter our adherence to PLOS ONE policies on sharing data and materials.” (as detailed online in our guide for authors http://journals.plos.org/plosone/s/competing-interests). If this adherence statement is not accurate and there are restrictions on sharing of data and/or materials, please state these. Please note that we cannot proceed with consideration of your article until this information has been declared.

We now provide the following updated Competing Interests Statement for Seibersdorf Laboratories:

Seibersdorf Laboratories is a commercial company operating independent ISO 17025-certified testing laboratories and is also active in independent research and consulting. The fact PS and GS are affiliated with this company does not alter our adherence to PLOS ONE policies on sharing data and materials.

Please include both an updated Funding Statement and Competing Interests Statement in your cover letter. We will change the online submission form on your behalf.

Done. We have included the statements in the cover letter.

4. Please include your tables as part of your main manuscript and remove the individual files. Please note that supplementary tables (should remain/ be uploaded) as separate "supporting information" files.

Done.

5. Please include captions for your Supporting Information files at the end of your manuscript, and update any in-text citations to match accordingly. Please see our Supporting Information guidelines for more information: http://journals.plos.org/plosone/s/supporting-information.

Done.

6. If the reviewer comments include a recommendation to cite specific previously published works, please review and evaluate these publications to determine whether they are relevant and should be cited. There is no requirement to cite

these works unless the editor has indicated otherwise.

OK, thank you for this hint.

To ensure your figures meet our technical requirements, please review our figure guidelines: https://journals.plos.org/plosone/s/figures

You may also use PLOS’s free figure tool, NAAS, to help you prepare publication quality figures: https://journals.plos.org/plosone/s/figures#loc-tools-for-figure-preparation. NAAS will assess whether your figures meet our technical requirements by comparing each figure against our figure specifications.

Done. Thank you for the advice. We have checked and optimized all our figures by using the NAAS figure tool.

Comments Reviewer #1:

1:

The review is not up to date on the recent literature. For example, on:

Bodin R, Seewooruttun C, Corona A, Delanaud S, Pelletier A, Villégier AS. Sex-dependent impact of perinatal 5G electromagnetic field exposure in the adolescent rat behavior. Environ Sci Pollut Res Int. 2023 Nov;30(53):113704-113717.

The reviewer is right. There was a long interval between the extraction and appraisal period of already included studies (until October 2024), manuscript submission (April 2025), and revision (March 2026). Additionally, numerous new studies have been published in the meantime because of research initiatives following the introduction of 5G technology. Therefore, we have gladly updated our search in the EMF-Portal, which resulted in 28 new articles. These new articles (including the one you mentioned, which was found as part of our updated search) have been summarized in a new chapter entitled 'Newly published articles since the last systematic search' (pages 32-33, lines 716–755) and are listed in Supporting Information S15 Reference List.

2:

In the Background “covid” seems to extrapolate beyond the topic while, more accuracy regarding the actual environmental exposures of the population with 5G frequencies is necessary. It is worth mentioning which frequencies are introduced in our public or occupancy environments and which are not introduced yet.

We thank the reviewer for their critical remark that we did not sufficiently outline which frequencies are already introduced in public and occupational environments and which are not.

We have added the relevant information to the Introduction:

Page 4, lines 81-89

Although 5G also uses frequency bands below 3 GHz, the vast majority of 5G applications and services are presently using the frequency range 3.4–3.8 GHz, particularly in urban environments. However, future 5G applications are going to use higher frequencies up to the millimeter-wave range (> 30 GHz). Mobile millimeter-wave applications in 5G public networks are presently commercialized in a few countries only (e.g., USA, Japan, Australia), mainly using the 26–28 GHz frequency range. In Europe this is still not the case and 5G data communication in the 26–28 GHz band is presently taking place on a relatively small scale for fixed wireless access (as alternative to glass-fiber internet access) and in private networks, typically set up in fully automated smart factories.

3:

The methods aimed first to select 5G and non5G EMF research. What was the limitation for not directly focusing on 5G frequencies with appropriate key words?

We are not quite sure if we have understood this comment correctly. We have carefully checked the wording of our methods concerning the eligibility criterion for the exposure condition in both the manuscript and the PROSPERO protocol. The methods state that we included studies that investigated exposure to radiofrequency electromagnetic fields in the 3–30 GHz frequency range (protocol), or 5G-relevant RF EMF (3–30 GHz) at any exposure level (manuscript). Thus, during the eligibility process, no priority was given to studies with real 5G exposure over those without. The frequency was the decisive factor. Had we focused solely on real 5G exposure, the validity of our systematic review would have been extremely limited due to the fact that 5G technology has only recently been introduced and 5G-related research projects have only just started (thus, only a few adequate publications were expected). Therefore, the aim of our review was to include all studies investigating the 3–30 GHz frequency range (regardless of application) in order to derive as much knowledge as possible concerning the potential biological effects of 5G technology.

4:

EMF effects may be linked to absorbed energy levels. It seems necessary to further mention and more deeply assess the exposure levels (specific absorption rate or surface power density, …) and durations (acute, chronical, intermittent, in min or hours, …) for each study, as well as considering the age of the rodent models.

All exposure-related data (e.g., specific absorption rate, power density) and durations for each study are listed in Tables A to G of S10 Table (see Supporting Information). Additionally, we have now added information about the sex and age of the animals in these tables. Please note, however, that the details on exposure metrics differed substantially between studies and that we could extract only the data the authors provided in the studies. In S12 Exposure Table, we converted the exposure metrics (where necessary and possible) and compared them to the international limits for each study to give the reader an idea how it compares to everyday exposure. Additionally, if we had any doubts regarding the exposure metrics in single studies, we have commented on it separately. In order to provide a more detailed explanation of the complex relationship between external exposure to electromagnetic fields (EMFs) and the amount of power absorbed internally in general, as well as the associated uncertainties, we have expanded the 'Description of Exposure' section (pages 5-6, lines 108-128).

5:

Minor point: “obeserved”

“In five studies, effects were obeserved at exposures above and below the limits”

Done. Thank you for the hint.

Comments Reviewer 2

Peer-Review Report for Manuscript PONE-D-25-16037

Title: Physiological and health-related effects of 5G-relevant radiofrequency fields: A systematic review of experimental studies in humans and mammals”

Overall Evaluation

The document presents a thorough and well-planned review of studies on 5Grelevant RFEMF (3–30 GHz) in humans and mammals. The topic is relevant to current events, important for society, and significant in science. The authors follow PRISMA guidelines, register their research protocol, and correctly use the OHAT risk of bias tools.

We would like to express our appreciation to the reviewer for this overall assessment.

However, some areas need more explanation, strengthening, or changes to make them clearer, more reproducible, and easier to understand. The main limitations concern how we handle heterogeneity, the decisions we make about evidence synthesis, the clarity of the presentations, and how we interpret the findings.

1. How would your conclusions change if exposure classification relative to ICNIRP limits were removed?

Removing our statements and comparisons to ICNIRP limits would not change our overall conclusion. Our conclusions on each outcome are based solely on our overall confidence in the synthesis of evidence. The process for conducting evidence synthesis was in accordance with international systematic review guidelines and detailed in the PROSPERO protocol and in the method section.

We only included the comparison with the ICNIRP limits as additional information for the following reason: The thermal effects of RF EMF are generally accepted, well-documented, and can be harmful at certain levels of intensity. Our systematic review also included studies in which exposure exceeded the limits, and many observed effects were likely due to thermal effects. We believe it is important to indicate to readers which studies this applies to, and to help them understand that such exposures are unlikely in everyday life. Otherwise, the reader could misinterpret potential harmful effects of studies with exposures above the limits.

However, we now only make statements relating to the ICNIRP limits for endpoints for which an overall evidence synthesis could be conducted. In contrast, we have removed any statements relating to the ICNIRP limits for other endpoints, where it was not possible to make a general statement anyway.

2. Can you provide a more detailed rationale for excluding cross-sectional studies?

We are pleased to provide the following additional information on our reasons for excluding cross-sectional studies to the section “Amendments compared to protocol“ (pages 14-15, lines 301-307):

Moreover, cross-sectional studies were excluded due to the lack of temporality and the consequential difficulty in proving causal effects, i.e., the simultaneous assessment of exposure and outcome makes a causal effect difficult to establish. Further, any reported effects may be due to factors other than exposure to RF EMF, and cross-sectional studies do not control for potential confounding factors. The general limitations of cross-sectional studies are also reflected by OHAT classifying the certainty of evidence as “low” for cross-sectional studies in the initial confidence rating based on study design [28].

3. Could you summarize the main methodological weaknesses in a single table?

We have summarized the main methodological weaknesses in an appropriate figure/table (see excerpt below). In the previous version of our manuscript, this figure was included in the supplementary data (formerly Supplementary Data 10). For 1st and 2nd tier studies, we now have moved it to the main manuscript (Fig 2) to highlight the importance of this topic. A similar table/figure for 3rd tier studies is still in the Supporting Information (S9 Risk of Bias Figure).

4. What are the most realistic exposure scenarios for humans at 3–30 GHz, and how do they compare to the experimental conditions?

We are thankful to the reviewer for this remark. Concerning the most realistic exposure scenarios for humans, we expanded our section ‘Description of exposure‘ (page 5, lines 98-107) appropriately:

The main sources of 5G exposure are mobile phones, mobile phone base stations of 5G networks, and wearables. Additionally, some types of wireless local area network (WLAN), radar applications (e.g., military radar) [25], and some medical applications [26] use frequencies in this range. The large variety of applications will lead to manifold exposure scenarios in practice. While exposure from base stations and access points usually leads to (widely) uniform far-field exposure, close near-field exposure conditions of different body parts are to be expected when using mobile phones and wearables, depending on which devices and how they are used. Particularly in case of millimeter-wave applications, it must be noted that absorption takes place almost entirely inside the skin and that inner organs remain largely unexposed due to the decreasing penetration depth with increasing frequency.

To address the second remark of the reviewer, whether the most realistic everyday exposure scenarios for humans can be compared to experimental conditions, we expanded our section ‘Review limitations‘ (page 31, lines 709-713) as follows:

Further, the numerous exposure scenarios that a person may encounter in everyday life are not all covered by the included studies. These scenarios may consist of a combination of exposure frequency and type (partial body vs. whole body), resulting in different target organs potentially being impacted. In most studies, whole-body exposure was investigated, whereas only few studies considered localized head exposure.

5. Please prepare a clean, journal-ready reviewer form, a point-by-point revision plan, or a rewritten conclusion section based on the manuscript.

Done. We applied the PLOS ONE style requirements and adhered to the editor's comments regarding the conditions for resubmission. Additionally, we have modified our conclusion to make it more nuanced in the Abstract (page 2, lines 33-38) and the Conclusion (pages 34-35, lines 786-790).

Major Comments

1. Evidence synthesis criteria need clearer justification

The manuscript frequently states that evidence synthesis was not possible due to heterogeneity, small numbers, or risk of bias. However, the decision rules for when synthesis is or is not performed are scattered and not consistently justified.

Provide a clear, unified decision framework early in the Methods section.

We thank the reviewer for this valuable comment. We realized that the conduct of our evidence synthesis was not presented prominently enough. In the previous version of our manuscript, the method used for the evidence synthesis was described in the method section in Supplementary Data 4 and the decision rules for when a synthesis was or was not performed were described at the beginning of the chapter „Summary of results and evidence synthesis“. We have now moved the entire method section including the chapter ‘Evidence synthesis and determination of confidence’ to the main manuscript (pages 13-14, lines 260-292). Additionally, the decision rules for when a synthesis was or was not performed have been moved to the end of the chapter (page 14, lines 293-297):

We did not perform an evidence synthesis if a) only single studies were available for a specific topic, b) ≤ 3 studies were available and in addition, risk of bias was identified in these studies (i.e., 2nd tier studies), or c) the studies lacked a sufficient homogeneity in terms of species, exposure and/or outcomes and, therefore, a meaningful comparison was not possible. If any of these conditions were met, we deemed it impossible to perform a meaningful evidence synthesis.

Consider adding a flowchart or table summarizing synthesis criteria.

Please refer to the Supporting Information (S13 Evidence Synthesis) for a table summarizing our evidence synthesis, including factors that decrease or increase confidence in the evidence for each endpoint (see excerpt below).

2. Interpretation of “inadequate evidence” should be more nuanced

a) The conclusion states that current scientific data are “inadequate to draw any conclusions.” However, the review does identify:

Moderate evidence for cytokine secretion effects

Low evidence for hippocampal structural changes

b) Consistent thermal effects at high exposures

c) The conclusion should distinguish between:

No evidence

Insufficient evidence

Evidence of no effect

Evidence of possible effect

This distinction is important for public health communication.

a) Thank you for pointing out that our overall conclusion was not sufficiently nuanced. We have modified our conclusion in the Abstract (page 2, lines 33-38) and Conclusion (pages 34-35, lines 786-790) as follows:

In summary, for most topics, the current scientific data were inadequate to draw any conclusions about the physiological and health-related effects of radiofrequency electromagnetic fields in the investigated frequency range of 3–30 GHz. However, moderate evidence below the limit values was found for cytokine secretion in mice and low evidence was found for structural changes in the hippocampus of rats above the limit values, when taking the exposure limits presently recommended by the International Commission on Non-Ionizing Radiation Protection as the basis.

b) Unfortunately, we disagree on this point. Thermal effects were only investigated in a few of the studies included in the current review, which does not allow to draw this general conclusion. However, to address this important issue, we included the following statement in our Conclusion (page 35, lines 790-793):

Although thermal effects are the only biological effect of RF EMF at frequencies above 10 MHz that is scientifically accepted, they were hardly investigated systematically in the studies included in this review. Nevertheless, many of the study authors suspect that thermal effects are the main effect in their studies.

c) The confidence in the body of evidence was assessed according to the approach described in the OHAT guidelines, which is based on the GRADE (Grading of Recommendations, Assessment, Development and Evaluation) approach. We defined the rules for all our methods in the review protocol according to the PRISMA-P guidelines and summarized them in the Methods section. Thus, unfortunately, we have to adhere to the protocol and cannot use the wording recommended by the reviewer for the overall evidence. For details of the rules and wording of the overall evidence, please refer to page 14, lines 283–292.

3. Exposure classification relative to ICNIRP limits is uncertain

The authors acknowledge that applying human exposure limits to animals is problematic. However, the manuscript still draws conclusions based on “above/below limit values.”

Provide a stronger methodological justification or

Add a sensitivity analysis showing how results change if classification is removed.

As stated earlier in this letter, removing our statements and comparisons to ICNIRP limits would not change our overall conclusion. We only included the comparison with the ICNIRP limits as additional information. However, we agree that the reasons for doing so were not outlined sufficiently. We have therefore added the following explanations to the chapter ‘Classification of exposure in relation to limit values’ (page 14, lines 317-321):

Thermal effects of RF EMF are generally accepted, well-documented, and can be harmful at certain levels of intensity. In cases where our systematic review includes studies in which exposure exceeds the limits, it is important to indicate, despite all the uncertainties, which studies this applies to. This will help to understand that such exposures are unlikely in everyday life. Otherwise, potential harmful effects of studies with exposures above the limits could be misinterpreted.

4. Lack of epidemiological studies deserves deeper discussion

The review found zero epidemiological studies meeting criteria. This is a major finding but is under-discussed.

Explain why epidemiological evidence is absent.

Discuss feasibility challenges (exposure assessment, confounding, latency).

We now discuss some hypotheses as to why we could not identify any epidemiological studies in our search to the chapter ‘Review limitations’ (page 30, lines 684-690):

Overall, we did not identify any epidemiological studies that fulfilled our eligibility criteria. However, this was to be expected for the following reasons: 1) 5G technology has only recently been introduced; 2) epidemiological studies, especially prospective cohort studies in the field of mobile communications, are usually conducted over a long period of time due to the long latency period of, e.g., brain tumors (the main area of interest) and other health effects; and 3) there are almost no new (or ongoing) projects investigating the health effects of mobile phone exposure in epidemiological studies.

Clarify implications for future research.

We added some implications for future epidemiological studies to the ‘Research needs’ chapter (page 34, lines 766-771):

However, conducting future case-control studies may be difficult as most people already use mobile phones today, which reduces the number of suitable control subjects without exposure. Any potential health-related effects could be elucidated by future cohort studies with improved exposure assessment, i.e. with objective prospective exposure data. Cross-sectional studies with subjective exposure data should be avoided as confidence in the evidence provided is low.

5. Risk-of-bias results need clearer visualization

The text describes risk-of-bias patterns, but readers would benefit from:

A summary figure (e.g., heatmap or bar chart) showing domain-level ratings.

A table highlighting the most common methodological weaknesses.

As stated earlier in this letter, we have summarized the main methodological weaknesses in an appropriate table/figure (see excerpt below). In the previous version of our manuscript, this figure was included in the supplementary data (formerly Supplementary Data 10). For 1st and 2nd tier studies, we now have moved the table/figure to the main manuscript (Fig 2) to highlight the importance of this topic. A similar table/figure for 3rd tier studies is still in the Supporting Information (S9 Risk of Bias Figure).

6. Some sections are overly long and descriptive

The Results section contains extensive study-by-study descriptions.

Consider condensing these into tables and focusing the text on patterns, not details.

Move long descriptions to Supplementary Data.

Thank you for your advice. We condensed and summarized the Results section as far as possible and removed any redundant information that is also presented in the Supporting Information “S10 Tables”.

Minor Comments

1. Clarify terminology

Terms like “effect,” “no effect,” “adverse,” and “beneficial” are used inconsistently. Define these terms explicitly.

We thank the reviewer for this comment. We realized that the terms “effect,” “no effect,” “adverse,” and “beneficial” had not been sufficiently defined, so we added the following definition to the „Description of outcome“ section (page 4-5, lines 86-91):

We define an adverse effect as any undesired harmful effect resulting from exposure. In contrast, beneficial effects are, e.g., desirable therapeutic or positive effects that improve a person’s health following exposure to RF EMF. Additionally, there may be effects that are unclear in terms of their relevance to health, e.g., changes in brain activity that have no further health implications. We define results as “no effect” if there are no differences between the exposed and control groups.

We have also carefully checked and adjusted the manuscript for any inconsistencies in the use of these terms.

2. Improve consistency in reporting exposure metrics

Some studies report SAR, others PD, others APD. Provide a standardized conversion table or explain why harmonization is not possible.

All exposure-related data (e.g., specific absorption rate, power density) for each study are listed in Tables A to G of S10 Tables (see Supporting Information). Please note, however, that the details on exposure metrics differed substantially between studies and that we could extract only the data the authors provided in the studies. To provide, in general, a more detailed explanation of the complex relationship between external exposure to electromagnetic fields (EMFs) and the amount of power absorbed internally, as well as the associated uncertainties, we have expanded the 'Description of Exposure' section (Page 5-6, lines 108-128) as follows:

Exposure to RF EMF induces body/tissue internal electric fields and the energy is absorbed in tissues. This can lead to an increase in tissue temperature and, depending on the level and duration of exposure, cause thermal biological effects. To protect the public from these effects, the most important parameters to limit exposure are the internal electric field strength and the rate of absorption of RF energy (specific absorption rate, SAR). The SAR is directly related to the internal electric field strength via the electric conductivity and the mass density of the tissue. In the 10 MHz–6 GHz range, SAR is the most important exposure parameter. The SAR can be determined numerically and experimentally; however, this requires sophisticated and elaborate methods.

At higher frequencies above 6 GHz, absorption is confined to the surface of the exposed tissue, and the absorbed power density (APD) can be used as an appropriate measure of exposure.

Alternatively, the more easily measurable incident electric and magnetic field strength or the incident power density (PD) at the site of the exposed body can be used as measurable exposure parameters. However, it is important to note that these body/tissue external exposure metrics can in general only be seen as rough approximations for the biologically relevant body/tissue internal exposure metrics. This is due to the coupling of the external fields into the body, which follows highly complex physical phenomena and therefore depends on several factors, e.g., frequency, size, orientation and posture of the exposed body, field polarization, and dielectric properties of body tissues. Hence, it is not possible to provide a generalized conversion table between the body/tissue external and body/tissue internal exposure metrics. The ICNIRP sets limits for both body/tissue internal and body/tissue external quantities, referring to the body/tissue external limits as "reference levels" and the body/tissue internal limits as "basic restrictions" [7].

3. Add a PRISMA flow diagram figure number

The text references “Fig. 1” but the figure is not visible in the excerpt. Ensure it is included.

According to the PLOS ONE Manuscript Body Formatting Guidelines (https://journals.plos.org/plosone/s/file?id=wjVg/PLOSOne_formatting_sample_main_body.pdf), figures should be uploaded separately as individual files. Please refer to the separate file to find Figure 1.

4. Improve readability of long paragraphs

Several paragraphs exceed one page in length. Breaking them up will improve clarity.

With the support of a native speaker, we have checked the manuscript for readability and optimized it where necessary. Thank you for your advice.

5. Check for minor typographical issues

A few small inconsistencies appear (spacing, capitalization, missing commas).

We have thoroughly checked and adjusted the manuscript for any typos and other inconsistencies.

4. Strengths of the Manuscript

Extensive literature search across PubMed, Web of Science, and EMF-Portal.

Protocol registration (PROSPERO) ensures transparency.

Use of OHAT risk-of-bias tool, appropriate for toxicology-related evidence.

Clear identification of research gaps, especially for high-frequency 5G bands.

Balanced tone, avoiding alarmism or unwarranted reassurance.

We express our appreciation to the reviewer for this overall assessment of our manuscript.

5. Recommendation

Major Revision

The manuscript is strong and valuable, but the issues above—especially regarding evidence synthesis criteria, interpretation, and clarity—should be addressed before publication.

We would like to thank the reviewer for their comprehensive evaluation of our manuscript, and we hope that we have addressed all the issues raised by the reviewer adequately.

Attachment Submitted filename: Response to Reviewers.docx

# Peer review: Decision Letter 1

**DOI:** 10.1371/journal.pone.0361104.r003

**Authors:** Olga Zeni

9 Sep 2026

Dear Dr. Driessen,

Thank you for submitting your manuscript to PLOS One. After careful consideration, we feel that it has merit but does not fully meet PLOS One’s publication criteria as it currently stands. Therefore, we invite you to submit a revised version of the manuscript that addresses the points raised during the review process.

Please submit your revised manuscript by Oct 24 2026 11:59PM. If you will need more time than this to complete your revisions, please reply to this message or contact the journal office at plosone@plos.org. When you're ready to submit your revision, log on to https://www.editorialmanager.com/pone/ and select the 'Submissions Needing Revision' folder to locate your manuscript file.

- A letter that responds to each point raised by the academic editor and reviewer(s). You should upload this letter as a separate file labeled 'Response to Reviewers'.
- A marked-up copy of your manuscript that highlights changes made to the original version. You should upload this as a separate file labeled 'Revised Manuscript with Track Changes'.
- An unmarked version of your revised paper without tracked changes. You should upload this as a separate file labeled 'Manuscript'.

If you would like to make changes to your financial disclosure, please include your updated statement in your cover letter. Guidelines for resubmitting your figure files are available below the reviewer comments at the end of this letter.

If applicable, we recommend that you deposit your laboratory protocols in protocols.io to enhance the reproducibility of your results. Protocols.io assigns your protocol its own identifier (DOI) so that it can be cited independently in the future. For instructions see: https://journals.plos.org/plosone/s/submission-guidelines#loc-laboratory-protocols. Additionally, PLOS One offers an option for publishing peer-reviewed Lab Protocol articles, which describe protocols hosted on protocols.io. Read more information on sharing protocols at https://plos.org/protocols?utm_medium=editorial-email&utm_source=authorletters&utm_campaign=protocols.

As the corresponding author, your ORCID iD is verified in the submission system and will appear in the published article. PLOS supports the use of ORCID, and we encourage all coauthors to register for an ORCID iD and use it as well. Please encourage your coauthors to verify their ORCID iD within the submission system before final acceptance, as unverified ORCID iDs will not appear in the published article. *Only*  the individual author can complete the verification step; PLOS staff *cannot*  verify ORCID iDs on behalf of authors.

We look forward to receiving your revised manuscript.

Kind regards,

Olga Zeni

Academic Editor

PLOS One

Journal Requirements:

If the reviewer comments include a recommendation to cite specific previously published works, please review and evaluate these publications to determine whether they are relevant and should be cited. There is no requirement to cite these works unless the editor has indicated otherwise.

Please review your reference list to ensure that it is complete and correct. If you have cited papers that have been retracted, please include the rationale for doing so in the manuscript text, or remove these references and replace them with relevant current references. Any changes to the reference list should be mentioned in the rebuttal letter that accompanies your revised manuscript. If you need to cite a retracted article, indicate the article’s retracted status in the References list and also include a citation and full reference for the retraction notice.

Additional Editor Comments :

Dear Authors,

The manuscript is greatly improved, but I would suggest you to address the minor point raised by Reviewer 3 before having it accepted for publication.

[Note: HTML markup is below. Please do not edit.]

Reviewers' comments:

Reviewer's Responses to Questions

**Comments to the Author**

Reviewer #2: All comments have been addressed

Reviewer #3: All comments have been addressed

**********

2. Is the manuscript technically sound, and do the data support the conclusions?

Reviewer #2: Yes

Reviewer #3: Yes

**********

3. Has the statistical analysis been performed appropriately and rigorously?

Reviewer #2: Yes

Reviewer #3: Yes

**********

4. Have the authors made all data underlying the findings in their manuscript fully available?

The PLOS Data policy

Reviewer #2: Yes

Reviewer #3: Yes

**********

5. Is the manuscript presented in an intelligible fashion and written in standard English?

Reviewer #2: Yes

Reviewer #3: Yes

**********

Reviewer #2: The authors have addressed all major and minor concerns. Specifically, the clear justification for evidence synthesis criteria, improved visualization of risk-of-bias data and the discussion regarding the absence of epidemiological studies now appropriate and well-reasoned

Reviewer #3: I was not one of the original reviewers, but I have examined their reviews and consider that the authors have adequately responded. This is an experienced group in SRs in the health effects of EMF and the paper is very good.

One suggestion: put in a statement that few if any of the papers used actual 5G waveforms (which use a particular form of pulse modulation). I have seen numerous misstatements by authors that they were investigating "effects of 5G" whereas they were using CW of GSM modulated energy for exposure..

**********

what does this mean?). If published, this will include your full peer review and any attached files.

If you choose “no”, your identity will remain anonymous but your review may still be made public.

**Do you want your identity to be public for this peer review?**  For information about this choice, including consent withdrawal, please see our Privacy Policy

Reviewer #2: **Yes:**  Md. Ariful Islam Arif

Reviewer #3: No

**********

[NOTE: If reviewer comments were submitted as an attachment file, they will be attached to this email and accessible via the submission site. Please log into your account, locate the manuscript record, and check for the action link "View Attachments". If this link does not appear, there are no attachment files.]

To ensure your figures meet our technical requirements, please review our figure guidelines: https://journals.plos.org/plosone/s/figures

You may also use PLOS’s free figure tool, NAAS, to help you prepare publication quality figures: https://journals.plos.org/plosone/s/figures#loc-tools-for-figure-preparation.

NAAS will assess whether your figures meet our technical requirements by comparing each figure against our figure specifications.

# Author comment: Author response to Decision Letter 2

**DOI:** 10.1371/journal.pone.0361104.r004

1 Oct 2026

We thank the editor and reviewers for their time to evaluate our revised manuscript and that they rated it as potentially acceptable for publication. We respond separately to each point raised by the reviewer.

Academic editor comment:

The manuscript is greatly improved, but I would suggest you to address the minor point raised by Reviewer 3 before having it accepted for publication.

We would like to express our appreciation to the editor for this overall assessment and are delighted to address the minor point raised by Reviewer#3 below.

Please note that we have corrected one of the references [24] from Qin et al. 2022 to Qin et al. 2024. We have therefore uploaded the updated version of each of the following files as well:

• Fig2.tif

• S10_Tables

• S12_Exposure

• S16_Reference_List

Comments Reviewer #2:

The authors have addressed all major and minor concerns. Specifically, the clear justification for evidence synthesis criteria, improved visualization of risk-of-bias data and the discussion regarding the absence of epidemiological studies now appropriate and well-reasoned.

We would like to thank the reviewer for taking the time to review our revision, and for his overall assessment.

Comments Reviewer #3:

I was not one of the original reviewers, but I have examined their reviews and consider that the authors have adequately responded. This is an experienced group in SRs in the health effects of EMF and the paper is very good.

Thank you for this positive feedback, which is much appreciated.

One suggestion: put in a statement that few if any of the papers used actual 5G waveforms (which use a particular form of pulse modulation). I have seen numerous misstatements by authors that they were investigating "effects of 5G" whereas they were using CW of GSM modulated energy for exposure.

The reviewer is right. In some cases, study authors claim to have investigated the “effects of 5G”, but this was not actually the case (e.g., [117-119]). We now provide detailed commentary on this issue, e.g., lines 738-742 (chapter “Newly published articles since the last systematic search”):

17 of the 28 relevant articles, including all human studies, explicitly stated that they investigated 5G exposure. However, not all of them used a 5G modulation scheme for the applied RF signal, e.g., three studies conducted by the same research group investigated GSM-modulated 3.5 GHz exposure, which is not representative of 5G exposure [117–119].

Additionally, we have modified the section of the 'Review Limitations' chapter to clarify that only a small proportion of the studies included in our systematic review explicitly involved 5G exposure.

Lines 702-707

Only five of the 50 studies included in the evidence synthesis explicitly stated that they were investigating 5G exposure [24,50,60,61,98]. Although the RF signals used in these studies were at frequencies used in 5G networks, they did not represent typical 5G modulation patterns but were either continuous wave or (GSM-like) pulse modulated. The remaining studies either used therapeutic applications, high-power microwaves, radar exposures or investigated the effects of microwave exposure without focusing on any specific application. Therefore, it is important to note that the (…)

Attachment Submitted filename: Response_to_Reviewers_auresp_2.docx

# Editor report: Decision Letter 2

**DOI:** 10.1371/journal.pone.0361104.r005

**Authors:** Olga Zeni

7 Oct 2026

Physiological and health-related effects of 5G-relevant radiofrequency fields: A systematic review of experimental studies in humans and mammals

PONE-D-25-16037R2

Dear Dr. Sarah Driessen,

We’re pleased to inform you that your manuscript has been judged scientifically suitable for publication and will be formally accepted for publication once it meets all outstanding technical requirements.

Within one week, you’ll receive an e-mail detailing the required amendments. When these have been addressed, you’ll receive a formal acceptance letter and your manuscript will be scheduled for publication.

An invoice will be generated when your article is formally accepted. Please note, if your institution has a publishing partnership with PLOS and your article meets the relevant criteria, all or part of your publication costs will be covered. Please make sure your user information is up-to-date by logging into Editorial Manager at Editorial Manager® and clicking the ‘Update My Information' link at the top of the page. For questions related to billing, please contact billing support.

If your institution or institutions have a press office, please notify them about your upcoming paper to help maximize its impact. If they’ll be preparing press materials, please inform our press team as soon as possible -- no later than 48 hours after receiving the formal acceptance. Your manuscript will remain under strict press embargo until 2 pm Eastern Time on the date of publication. For more information, please contact onepress@plos.org.

Kind regards,

Olga Zeni

Academic Editor

PLOS One

Additional Editor Comments (optional):

Reviewers' comments:

# Editor report: Acceptance letter

**DOI:** 10.1371/journal.pone.0361104.r006

**Authors:** Olga Zeni

PONE-D-25-16037R2

PLOS One

Dear Dr. Driessen,

I'm pleased to inform you that your manuscript has been deemed suitable for publication in PLOS One. Congratulations! Your manuscript is now being handed over to our production team.

At this stage, our production department will prepare your paper for publication. This includes ensuring the following:

* All references, tables, and figures are properly cited

* All relevant supporting information is included in the manuscript submission,

* There are no issues that prevent the paper from being properly typeset

You will receive further instructions from the production team, including instructions on how to review your proof when it is ready. Please keep in mind that we are working through a large volume of accepted articles, so please give us a few days to review your paper and let you know the next and final steps.

Lastly, if your institution or institutions have a press office, please let them know about your upcoming paper now to help maximize its impact. If they'll be preparing press materials, please inform our press team within the next 48 hours. Your manuscript will remain under strict press embargo until 2 pm Eastern Time on the date of publication. For more information, please contact onepress@plos.org.

You will receive an invoice from PLOS for your publication fee after your manuscript has reached the completed accept phase. If you receive an email requesting payment before acceptance or for any other service, this may be a phishing scheme. Learn how to identify phishing emails and protect your accounts at https://explore.plos.org/phishing.

If we can help with anything else, please email us at customercare@plos.org.

Thank you for submitting your work to PLOS One and supporting open access.

Kind regards,

PLOS One Editorial Office Staff

on behalf of

Dr. Olga Zeni

Academic Editor

PLOS One
